# Non-Mendelian inheritance of DNA methylation patterns in mice

**DOI:** 10.1038/s41588-026-02604-z

**Published:** 2026-05-20

**Authors:** Adam Davidovich, Danila Cuomo, Hang Su, Sandeep Kambhampati, Qingqing Gong, Alexandra Naron, Rakel Tryggvadottir, Leonard McMillan, Kasper D. Hansen, David W. Threadgill, Andrew P. Feinberg

**Affiliations:** 1 Center for Epigenetics, Johns Hopkins School of Medicine, Baltimore, MD USA; 2 Department of Biomedical Engineering, Johns Hopkins University, Baltimore, MD USA; 3 Department of Cell Biology and Genetics, Texas A&M University, College Station, TX USA; 4 Bioinformatics and Computational Biology Curriculum, University of North Carolina at Chapel Hill, Chapel Hill, NC USA; 5 Program in Biochemistry, Cellular, and Molecular Biology, Johns Hopkins University, Baltimore, MD USA; 6 Interdisciplinary Graduate Program in Genetics and Genomics, Texas A&M University, College Station, TX USA; 7 Department of Biostatistics, Johns Hopkins Bloomberg School of Public Health, Baltimore, MD USA; 8 Department of Genetic Medicine, Johns Hopkins School of Medicine, Baltimore, MD USA; 9 Department of Nutrition, Texas A&M University, College Station, TX USA; 10 Department of Medicine, Johns Hopkins School of Medicine, Baltimore, MD USA; 11 Department of Mental Health, Johns Hopkins Bloomberg School of Public Health, Baltimore, MD USA; 12 Department of Biomedical Engineering, Tel Aviv University, Tel Aviv, Israel

**Keywords:** Epigenomics, Genome informatics

## Abstract

Epigenetic mechanisms such as genomic imprinting demonstrate that molecular inheritance can deviate from typical Mendelian patterns. Despite this, the intergenerational inheritance of DNA methylation remains poorly understood. Here we developed a genome-wide approach to study epigenetic inheritance in mice using long-read nanopore sequencing. Using this approach in both liver and muscle, we found that ~93% of autosomal epigenetic inheritance patterns followed Mendel’s laws, primarily driven by *cis*-acting methylation quantitative trait loci. However, we also identified extensive non-Mendelian inheritance, including emergent epigenetic inheritance patterns, widespread sex-specific DNA methylation patterns localized to the liver, and five seemingly new autosomal and X-linked imprinted genes. Notably, we also report an example of naturally occurring intergenerational paramutation, confirmed over strain-specific transposable elements within *Capn11* and highly likely at *Vps37c*. Overall, an unexpectedly high ~7% of autosomal epigenetic inheritance patterns identified were non-Mendelian, highlighting the importance of epigenetic information in the analysis of inherited traits and disorders.

## Abstract (web summary)

Davidovich et al. investigate allele-specific DNA methylation inheritance patterns in mouse liver and muscle. Most patterns are Mendelian, but ~7% are non-Mendelian, including new imprinted genes and a paramutation at the *Capn11* locus.

## Main

It has been hypothesized that the inheritance of epigenetic traits, such as DNA methylation, may be more prevalent than is currently established^1,2^. This phenomenon could help explain poorly understood concepts in genetics, including incomplete penetrance^3,4^, symptomatic heterozygotes for traits with recessive monogenic inheritance^5^, and complex phenotypes influenced by environmental exposure and transmitted in a non-Mendelian manner^6–10^. Established non-Mendelian patterns of epigenetic inheritance include (1) parent-of-origin-specific DNA methylation patterns established by genomic imprinting^11,12^; (2) polar overdominance, a combined genetic and parent-of-origin effect exemplified by the callipyge locus in sheep^13^; and (3) transvection and paramutation, in which the methylation of an allele is altered due to the presence of its homolog, previously observed only after transgenic manipulation^14–17^. Furthermore, some genomic loci exhibit complex patterns of inheritance, such as the agouti viable yellow allele (*A*^*vy*^), regulated by an intracisternal A particle (IAP), a subclass of endogenous retroviruses (ERV), for which epigenetic inheritance is influenced by parent-of-origin, strain effects and environmental exposures^18,19^. However, these mechanisms of non-Mendelian inheritance remain insufficiently studied in mammals.

**Extended Data Fig. 1:**
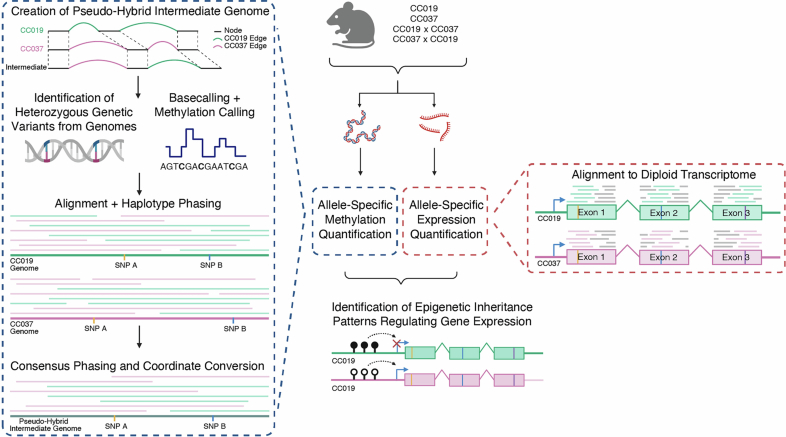
Computational pipeline for the analysis of allele-specific methylation and expression in crosses of inbred mice. ONT sequencing was performed on liver and muscle DNA from the inbred and F1 generations as well as liver DNA from the F2 generation of two CC mouse strains, CC019 and CC037. Matched RNA-seq was also performed on liver RNA from the inbred and F1 generations. ONT sequencing data were aligned and phased twice, once using each of the CC reference genomes. Only consensus phasing decisions, in which the assignment of each read is consistent independent of the choice of reference genome, were used to maximize the accuracy of phased methylation estimates and minimize the introduction of reference bias. Phased methylation data were then mapped to a pseudo-hybrid intermediate genome, comprised of all anchors and each of the longer respective edges of CC019 or CC037 from the Collaborative Cross Graphical Genome. Analysis of methylation in the pseudo-hybrid intermediate genome enables the retention of strain-specific methylation information to maximize the amount of analyzable data without the introduction of additional reference bias. RNA-seq data were analyzed by aligning reads to a diploid transcriptome containing transcript information for both CC019 and CC037. Allele-specific methylation and expression data were then used to identify various forms of epigenetic inheritance patterns and subsequently analyze their effects on the expression of associated genes.

Here we performed a genome-scale analysis of the inheritance of DNA methylation patterns in two tissues, examining the contributions of genotype, parent-of-origin and sex to establish a foundation for understanding the complex relationship between genetics, epigenetics, environment and disease. As there is ambiguity regarding the terminology surrounding intergenerational epigenetic inheritance, we provide a glossary reflecting our usage (Supplementary Information). For this analysis, we treated DNA methylation levels at distinct loci as traits to characterize their inheritance patterns across multiple generations in crosses of inbred mice. We selected two strains from the Collaborative Cross (CC) mouse model, a panel of recombinant inbred strains derived from eight founders, to increase interstrain heterozygosity while minimizing hybrid dysgenesis and regulatory incompatibilities often seen in interspecific and subspecific crosses^20,21^. Using long-read Oxford Nanopore Technologies (ONT) sequencing of these strains and their F1 and F2 crosses, we directly measured both genetic sequence and DNA methylation on the same DNA molecules, enabling haplotype phasing. We developed a tool to map methylation data from these highly divergent CC strains into a single pseudohybrid intermediate genome, enabling rigorous and unbiased allele-specific methylation analysis^22^ (Extended Data Fig. 1). Additionally, to assess the functional consequences of these epigenetic inheritance patterns, we coupled this methylation analysis with measurements of allele-specific expression. Lastly, as DNA methylation is known to be tissue-specific, we analyzed these inheritance patterns genome-wide in two tissues.

Using this approach, we identified at least 522 autosomal examples of non-Mendelian inheritance, including a paramutation event identified in a mammalian genome in the absence of transgenic manipulation, 54 emergent epigenetic inheritance patterns, at least 51 regions under the control of distal *trans*-acting methylation quantitative trait loci (meQTLs), and at least five imprinted genes to our knowledge not previously reported, including one located on the X chromosome. We also identified two additional highly likely examples of paramutation over strain-specific IAP elements. Furthermore, we characterized and mapped many examples of Mendelian inheritance of DNA methylation patterns, including 7,081 regions under the control of *cis*-acting meQTLs.

## Results

### Integrated genetic and epigenetic analysis of the intergenerational inheritance of DNA methylation patterns

**Fig. 1:**
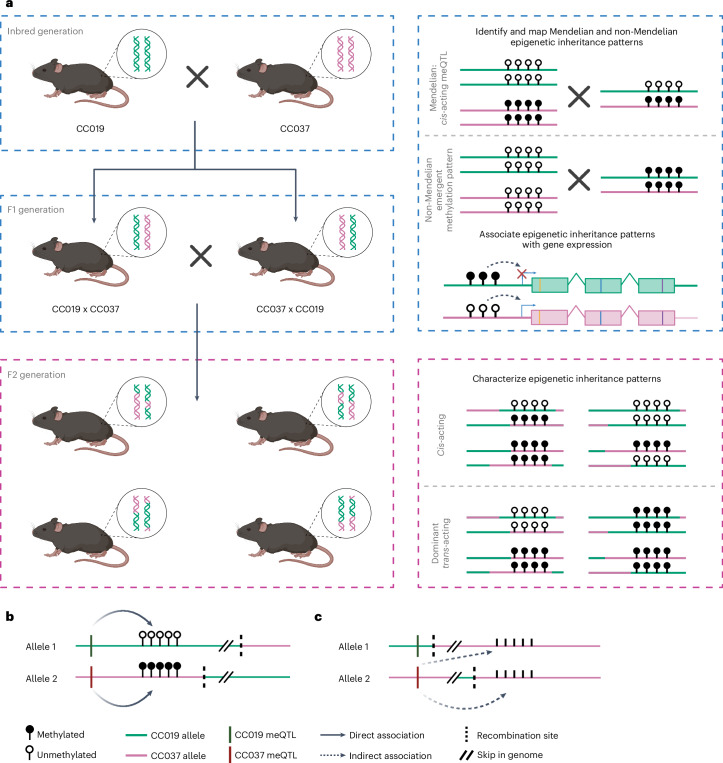
Identification and characterization of Mendelian and non-Mendelian epigenetic inheritance patterns. **a**, Long-read ONT sequencing was performed on DNA from inbred samples of two genetically divergent CC strains and their F1 crosses. Allele-specific methylation patterns were analyzed to identify and map both Mendelian and non-Mendelian patterns of epigenetic inheritance. Matched RNA was also sequenced and used to associate the identified epigenetic inheritance patterns with gene expression. Additionally, F2s from the same CC strains were sequenced to further investigate the genetic and nongenetic factors regulating the identified epigenetic inheritance patterns. **b**, Schematic of a *cis*-acting meQTL in an F2 mouse, indicating that the local haplotype of the DMR will define the identity of the meQTL, as recombination is unlikely to occur between the SNP and the CpGs over which it regulates methylation when they are in close proximity (that is, <50 cm apart). A *cis*-acting regulatory mechanism will establish an F2 population in which the local haplotype of the DMR is associated with the methylation level (for example, CC019 alleles are unmethylated while CC037 alleles are methylated), as shown in **a**. **c**, Schematic of a dominant *trans*-acting meQTL in an F2 mouse, indicating that the local haplotype of the DMR will not necessarily define the identity of the meQTL, as there is a high likelihood that independent assortment of chromosomes or meiotic recombination will occur between the SNP and the CpGs over which it regulates methylation if they are >50 cM apart. A *trans*-acting regulatory mechanism will establish an F2 population in which the local haplotype over the DMR is not associated with the methylation level (that is, there is no clear methylation pattern associated with the CC019 or CC037 alleles), as shown for a dominant *trans*-acting meQTL in **a**. Schematics in **a** created in BioRender; Feinberg, A. https://biorender.com/xobyc15 (2026).

We designed a combined genetic and epigenetic analysis to identify DNA methylation patterns related to genotype, sex, intergenerational inheritance, tissue and parent-of-origin effects (Fig. 1a). This was achieved using genome-wide long-read ONT sequencing of liver and muscle DNA from 26 (15 liver, 11 muscle) male and female mice of two genetically distinct inbred CC strains, CC019/TauUnc and CC037/TauUnc, as well as 34 (22 liver, 12 muscle) of their F1 hybrids in both cross directions. Muscle and liver were profiled in distinct mice. We subsequently performed targeted ONT sequencing of liver DNA from 19 F2 crosses, generated by crossing members of the F1 generation, to further analyze candidate epigenetic inheritance patterns identified using the inbred and F1 datasets. Analysis of the F2 generation is essential to distinguish epigenetic inheritance patterns mediated by *cis*-acting and *trans*-acting regulatory factors, as described in Fig. 1b,c. Sample information and sequencing statistics for all ONT samples are provided in Supplementary Table 1.

Within the CC019 and CC037 reference genomes, there are ~19 million CpGs, of which ~4.8 million overlap regions identical by descent (IBD), that is, lacking genetic variation between the two strains. Using our experimental and computational approach, we analyzed ~12 million autosomal CpGs and ~350,000 X-chromosomal CpGs in both tissues, accounting for ~90% and ~85% of the CpGs present in both strains, mappable to the intermediate genome and within non-IBD regions.

To assess the functional implications of these methylation patterns, we sequenced liver RNA from a subset of the inbred and F1 samples that were also profiled using ONT sequencing. Outputs were evaluated for allele specific and total expression in the context of each identified epigenetic inheritance pattern, as detailed in the Methods. Only 9.5% of genes (4,092/42,880 genetic elements considered; 3,956/19,655 protein-coding genes) were analyzable, that is, they contained transcribed polymorphisms that differ between CC019 and CC037 with expression levels exceeding a minimum threshold, thereby limiting allele-specific expression comparisons to a subset of genes.

We defined and searched for 12 distinct patterns of epigenetic inheritance. Representations of each epigenetic inheritance pattern identified on the autosomes, as well as the number of regions exhibiting each pattern, are shown in Fig. 2. A comprehensive list of these regions is included in Supplementary Tables 2 and 3, and the top ten regions for each pattern are shown in Supplementary Figs. 1–24. Each pattern is discussed in detail below and in Supplementary Notes.

**Fig. 2:**
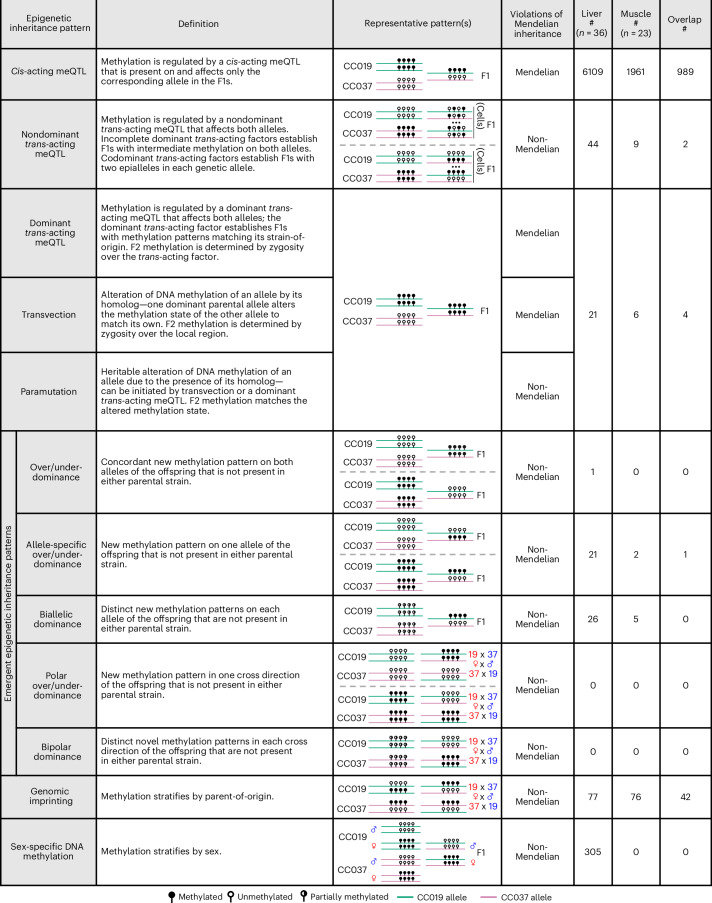
Autosomal epigenetic inheritance patterns. Definitions, representative patterns and violations to Mendelian inheritance of each autosomal epigenetic inheritance pattern identified, as well as the number of each pattern identified in both the liver and muscle and the number common to both tissues.

### *Cis*-acting meQTLs (pattern 1)

**Fig. 3:**
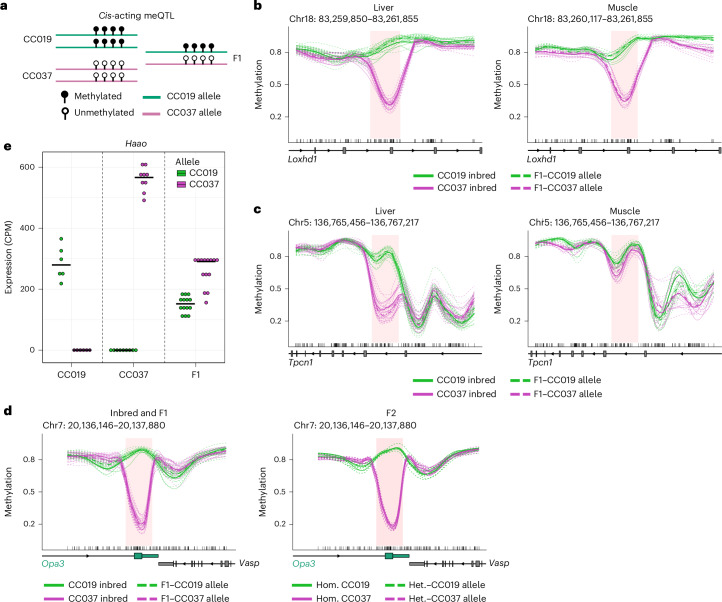
*Cis*-acting meQTLs. **a**, Generic representation of methylation in the context of a *cis*-acting meQTL in inbred CC019 and CC037 samples and their F1 crosses. **b**, C*is*-acting meQTL identified in both the liver (left) and muscle (right) over *Loxhd1*. **c**, Methylation pattern of the tissue-specific *cis*-acting meQTL identified over *Tpcn1* in the liver (left) and not in the muscle (right). **d**, *Cis*-acting meQTL identified in the liver over *Vasp* and *Opa3*, shown for inbred and F1 samples (left) and F2 samples (right). **e**, Allele-specific expression of *Haao* in the liver, exhibiting regulation by a *cis*-acting eQTL and overlapping a *cis*-acting meQTL in both the liver and muscle. Expression was analyzed in 6 CC019 inbred, 9 CC037 inbred and 14 F1 mice. For the inbred samples (CC019 and CC037), the expression levels of the absent alleles are provided as technical controls confirming accurate allelic assignment. For the methylation plots shown in **b**–**d**, bold lines represent coverage-weighted mean methylation of the respective group and CpG sites included in the final analysis are denoted by tick marks on the *x* axis. CPM, counts per million; hom., homozygous; het., heterozygous.

Among the observed epigenetic inheritance patterns, the most prevalent in both tissues were regions of the genome in which methylation stratifies by genotype and allele (Fig. 3a). These methylation patterns are established by *cis*-acting meQTLs, defined as genetic variants that regulate DNA methylation levels of proximal CpGs located on the same allele^23^. In total, 7,081 genomic regions were identified that follow this pattern in at least one tissue, accounting for ~93% of autosomal intergenerational epigenetic inheritance patterns (Figs. 2 and 3b,c, Supplementary Figs. 1 and 2, and Supplementary Tables 2 and 3). We find that 16% and 50% of the *cis*-acting meQTLs identified in liver and muscle, respectively, are present in both tissues. Examples of tissue-independent and tissue-specific *cis*-acting meQTLs are shown in Fig. 3b,c.

To confirm that these differentially methylated regions (DMRs) are controlled by *cis*-acting regulatory elements, we performed targeted ONT sequencing on 20 candidate regions in F2 mice. As expected, all 20 regions exhibit methylation levels that segregate with the local haplotype (Fig. 3d and Supplementary Data 1), thereby validating that these methylation patterns are established by *cis*-acting meQTLs.

To assess the effects of these *cis*-acting meQTLs on gene expression, we examined genes whose promoters or enhancers overlap with, or are located within 100 kb of, the corresponding DMR. Specifically, we searched for genes with allelic imbalance in the F1s which mirrors the expression difference observed between the two parental strains (Fig. 3e) with no limitation on which allele is more highly expressed. Allele-specific expression could be assessed for only a subset of these genes (2,610/13,013; Supplementary Table 4). We identified 700 genes showing *cis*-acting regulation of both expression and DNA methylation, suggesting that many *cis*-acting meQTLs may also function as expression quantitative trait loci (Supplementary Tables 4 and 5).

### Nondominant *trans*-acting meQTLs (pattern 2)

**Fig. 4:**
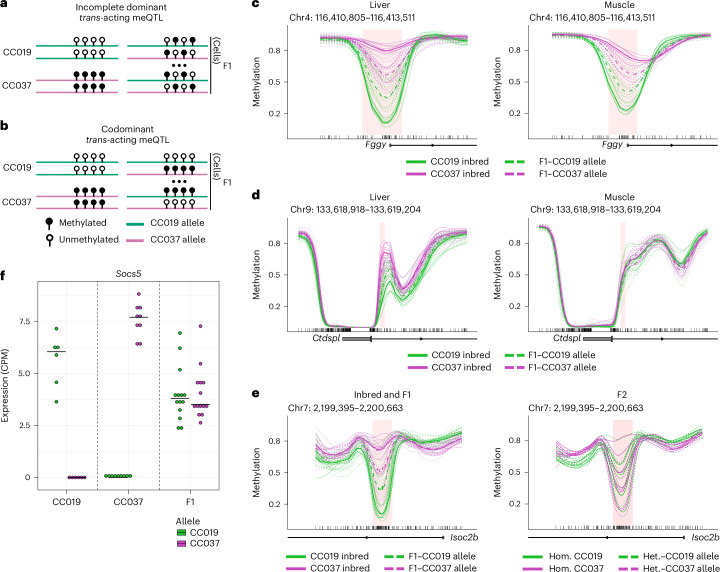
Nondominant *trans*-acting meQTLs. **a**, Generic representation of methylation in the context of an incomplete dominant *trans*-acting meQTL in inbred CC019 and CC037 samples and their F1 crosses. **b**, Generic representation of methylation in the context of a codominant *trans*-acting meQTL. Methylation patterns in **a** and **b** indicate that read-level methylation analyses are required to distinguish incomplete dominant from codominant *trans*-acting meQTLs, as their methylation patterns will appear identical at the sample level. **c**, Nondominant *trans*-acting meQTL identified in both the liver (left) and the muscle (right) over *Fggy*. **d**, Tissue-specific nondominant *trans*-acting meQTL identified over *Ctdspl* in the liver (left) and not in the muscle (right). **e**, Nondominant *trans*-acting meQTL identified in the liver over *Isoc2b*, shown for inbred and F1 samples (left) and F2 samples (right). **f**, Allele-specific expression of *Socs5* in the liver, exhibiting regulation by a tissue-specific nondominant *trans*-acting eQTL identified in the liver. Expression was analyzed in 6 CC019 inbred, 9 CC037 inbred and 14 F1 mice. For the inbred samples (CC019 and CC037), the expression levels of the absent alleles are provided as technical controls confirming accurate allelic assignment. For the methylation plots shown in **c**–**e**, bold lines represent coverage-weighted mean methylation of the respective group and CpG sites included in the final analysis are denoted by tick marks on the *x* axis.

We also identified regions in which methylation stratifies by genotype only in the inbred samples, whereas both alleles of the F1s exhibit intermediate methylation levels (Fig. 4a,b). These patterns are likely caused by *trans*-acting meQTLs, defined as genetic variants that influence methylation levels of distal CpGs and can regulate both alleles. We identified 51 genomic regions that exhibit this methylation pattern in at least one tissue (Figs. 2 and 4c, Supplementary Figs. 3 and 4, and Supplementary Tables 2 and 3). While there were fewer nondominant *trans*-acting meQTLs identified in the muscle (*n* = 9) than in the liver (*n* = 44), only 22% (2/9) of the muscle nondominant *trans*-acting meQTLs were also observed in the liver (Fig. 4c,d), suggesting that these nondominant *trans*-acting meQTLs may be more tissue-specific than their *cis*-acting counterparts.

**Extended Data Fig. 2:**
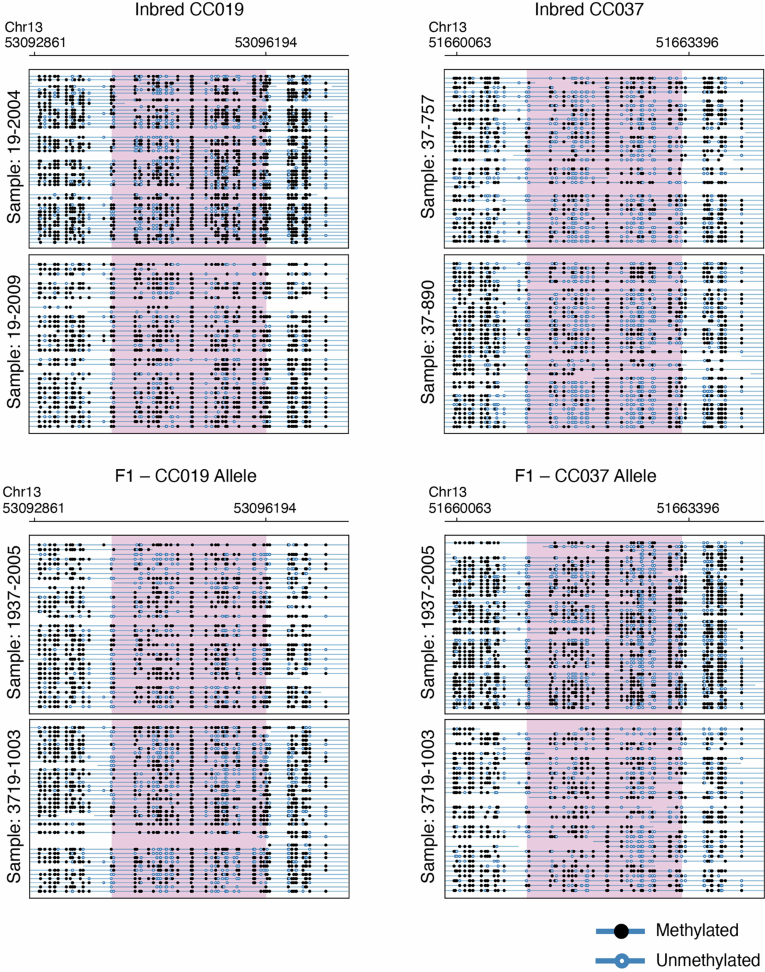
Read-level methylation of an incomplete dominant *trans*-acting meQTL in the liver. Read-level methylation of a non-dominant *trans*-acting meQTL identified in the liver, which indicates incomplete dominance due to the lack of two clear epialleles. All reads overlapping this region are shown for two representative replicates per group—inbred CC019, inbred CC037, F1–CC019 allele and F1–CC037 allele.

**Extended Data Fig. 3:**
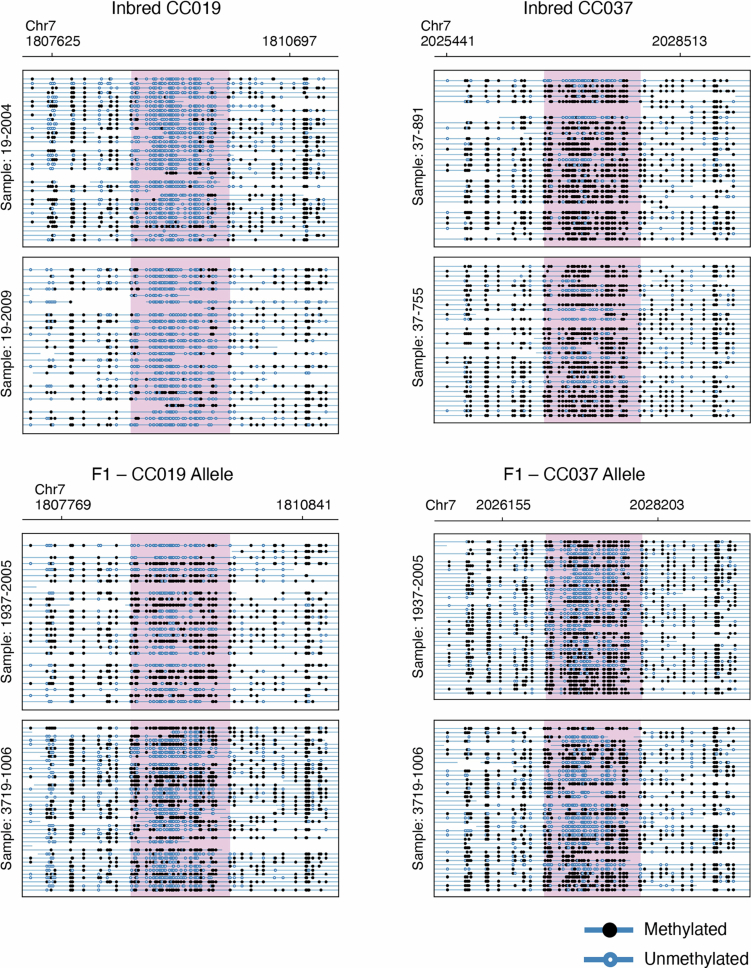
Read-level methylation of a codominant *trans*-acting meQTL in the liver. Read-level methylation of a non-dominant *trans*-acting meQTL identified in the liver, which indicates codominance due to the presence of two clear epi-alleles within each genetic allele of the F1 generation, matching the methylation of the inbred parental strains. All reads overlapping this region are shown for two representative replicates per group—inbred CC019, inbred CC037, F1–CC019 allele and F1–CC037 allele.

Nondominant *trans*-acting meQTLs comprise two distinct subpatterns—incomplete dominance and codominance. While each of these subpatterns establishes identical methylation patterns at the sample level, they can be distinguished using long-read sequencing data by further assessing the variability of DNA methylation at both the cellular and CpG levels. In regions regulated by an incomplete dominant *trans*-acting meQTL, each sequenced strand of DNA from both parental alleles of the F1 crosses exhibit intermediate methylation levels which are between the two inbred parental strains, indicating CpG-level methylation variability within each cell (Fig. 4a and Extended Data Fig. 2). Conversely, in regions regulated by codominant *trans*-acting meQTLs, each phased F1 alleles exhibits two clear epialleles, groups of reads that are either fully methylated or fully unmethylated, indicating variability at the cellular level (Fig. 4b and Extended Data Fig. 3). Thus, methylation differences between the inbred strains and F1 crosses in these regions reflect shifts in the distributions of these epialleles.

Five candidate regions regulated by nondominant *trans-*acting meQTLs were chosen for subsequent targeted analysis in the F2s. Three of these regions exhibited no clear relationship between local haplotype and observed methylation states (Fig. 4e and Supplementary Data 1), further supporting regulation by *trans*-acting genetic variants. The remaining two DMRs exhibited genotype-specific and allele-specific methylation patterns in the F2 generation that were smaller than observed in the inbred generation and roughly the same as in the F1 generation (Supplementary Data 1).

We subsequently analyzed allele-specific and total expression levels of genes associated with these DMRs via direct promoter/enhancer overlap or by genomic proximity to identify instances in which expression levels are consistent with regulation by nondominant *trans*-acting meQTLs. These genes are expected to exhibit expression levels that stratify by genotype in inbred samples but lack significant allelic imbalance in the F1s. We identified nine such instances (Fig. 4f and Supplementary Tables 4 and 5), suggesting that these nondominant *trans*-acting meQTLs may also function as expression quantitative trait loci (eQTL), thereby providing an additional link between these regulatory mechanisms.

### Dominant *trans*-acting meQTLs, transvection and paramutation (patterns 3–5)

**Fig. 5:**
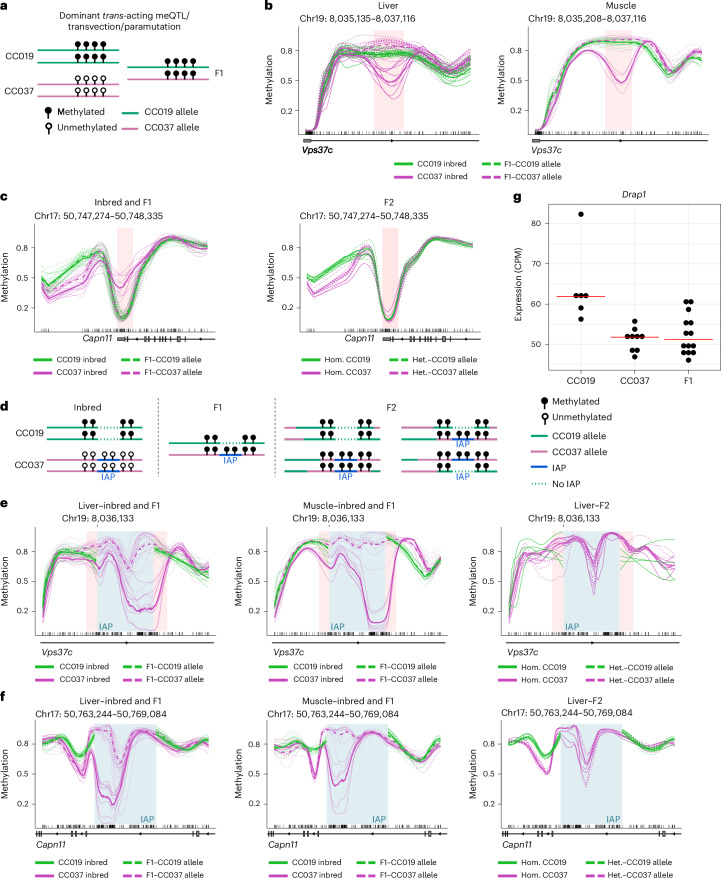
Dominant *trans*-acting meQTLs, transvection and paramutation. **a**, Generic representation of methylation in the context of a dominant *trans*-acting meQTL, transvection or paramutation in inbred CC019 and CC037 samples and their F1 crosses. **b**, Dominant *trans*-acting meQTL/transvection/paramutation methylation pattern identified in both the liver (left) and muscle (right) over *Vps37c*. **c**, Paramutation methylation pattern identified in the liver over *Capn11*, shown for inbred and F1 samples (left) and F2 samples (right). **d**, Generic representation of methylation in the context of paramutation driven by the presence of a strain-specific IAP element in the inbred (left), F1 (middle) and F2 (right) generations. **e**, Methylation over the CC037-specific *Vps37c* IAP is shown for the inbred and F1 samples of the liver (left), inbred and F1 samples of the muscle (center), and F2 samples of the liver (left). For the liver F2s, only those samples/alleles with an average coverage of at least 1× over the region shown are included. The IAP is highlighted in blue and the original DMR (identified without inclusion of the IAP in the reference genome) is highlighted in red. As these plots no longer conform to the original pseudohybrid intermediate coordinate system due to the addition of the IAP, a single anchor coordinate indicating the start of the IAP is shown. **f**, Methylation over the CC037-specific *Capn11* IAP shown for the inbred and F1 samples of the liver (left), inbred and F1 samples of muscle (center), and F2 samples of the liver (left). The IAP is highlighted in blue. **g**, Total expression of *Drap1* in the liver, exhibiting a dominant *trans*-acting eQTL/transvection/paramutation expression pattern and proximal to a dominant *trans*-acting meQTL/transvection/paramutation methylation pattern in the liver. Expression was analyzed in 6 CC019 inbred, 9 CC037 inbred and 14 F1 mice. For the methylation plots shown in **b**, **c**, **e** and **f**, bold lines represent coverage-weighted mean methylation of the respective groups and CpG sites included in the final analysis are denoted by tick marks on the *x* axis.

Our analysis further revealed epigenetic inheritance patterns in which methylation stratifies by genotype in the inbred samples, while methylation on both alleles of the F1s resembles only one of the parental strains (Fig. 5a,b). We identified 23 such regions present in at least one tissue (Fig. 2, Supplementary Figs. 5 and 6, and Supplementary Tables 2 and 3). Of these, four were identified in both tissues, representing 17% and 67% of the regions identified in the liver and muscle, respectively. Included among these four tissue-independent DMRs is a region overlapping the gene *Vps37c* (Fig. 5b), involved in the vesicular sorting of endocytic cargo^24^ and subject to further investigation below.

**Extended Data Fig. 4:**
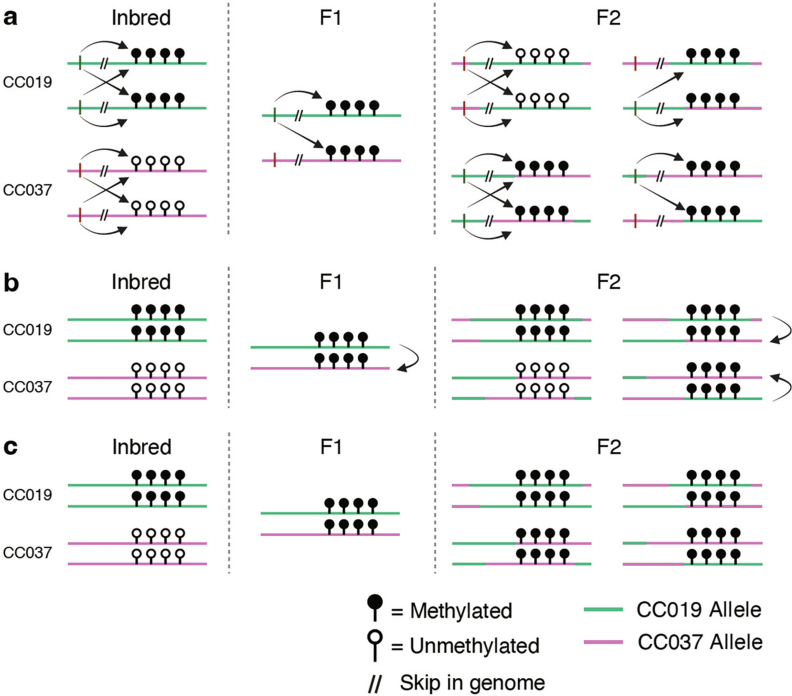
Schematics for dominant *trans*-acting meQTLs, transvection and paramutation in the F2 generation. **a**, Generic representation of methylation in the context of a dominant *trans*-acting meQTL in the inbred (left), F1 (middle) and F2 (right) generations. **b**, Generic representation of methylation in the context of transvection in the inbred (left), F1 (middle) and F2 (right) generations. **c**, Generic representation of methylation in the context of paramutation in the inbred (left), F1 (middle) and F2 (right) generations. Methylation patterns in **a**–**c** indicate that sequencing of the F2 generation is required to distinguish dominant *trans*-acting meQTLs, transvection and paramutation.

This pattern may conceivably arise from three distinct underlying mechanisms—dominant *trans*-acting meQTLs, transvection or paramutation (Supplementary Notes 1 and 2), and additional data from the F2 generation are required to distinguish these potential mechanisms (Extended Data Fig. 4).

**Extended Data Fig. 5:**
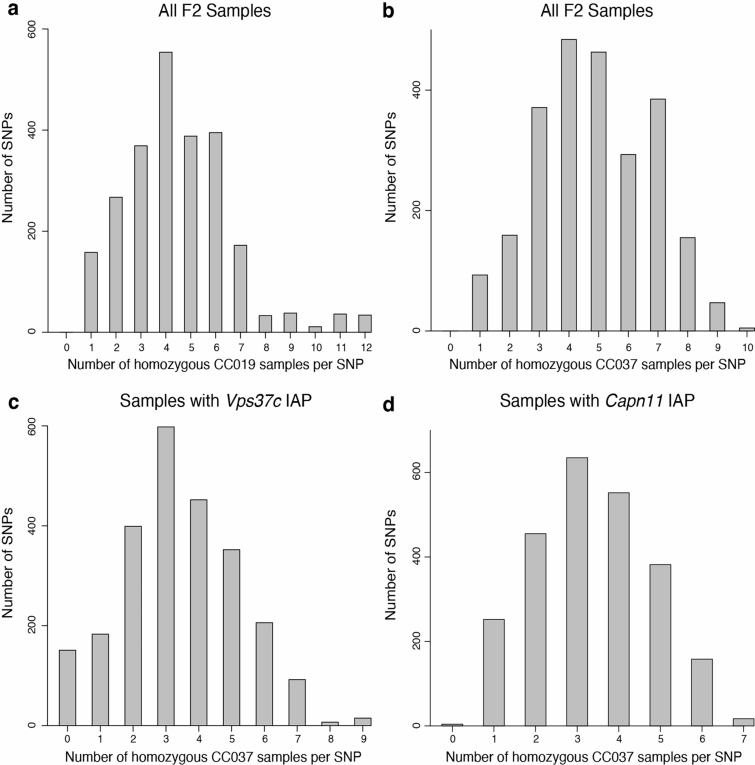
F2 SNP genotyping histograms. Histograms of genome-wide SNP genotyping for: **a**, all 19 sequenced F2 samples, which are homozygous for SNPs contributed by the CC019 strain; **b**, all 19 sequenced F2 samples, which are homozygous for SNPs contributed by the CC037 strain; **c**, 14 sequenced F2 samples which have at least one copy of the *Vps37c* IAP and are homozygous for SNPs contributed by the CC037 strain; **d**, 12 sequenced F2 samples, which have at least one copy of the *Capn11* IAP and are homozygous for SNPs contributed by the CC037 strain.

To differentiate genomic regions regulated by these three mechanisms, we profiled a set of three candidate regions in the F2 generation using targeted ONT sequencing on liver DNA from 19 F2 mice selected from a set of 58 based on their genotyping such that, at each region in the genome, there is at least one sample which is homozygous for each of the two parental alleles—CC019 and CC037 (Extended Data Fig. 5a,b). This ensures that, if the methylation of a candidate region is driven by a dominant *trans*-acting meQTL, at least one sample will be homozygous for the nondominant allele and will therefore exhibit the nondominant methylation pattern, allowing distinction from paramutation.

**Extended Data Fig. 6:**
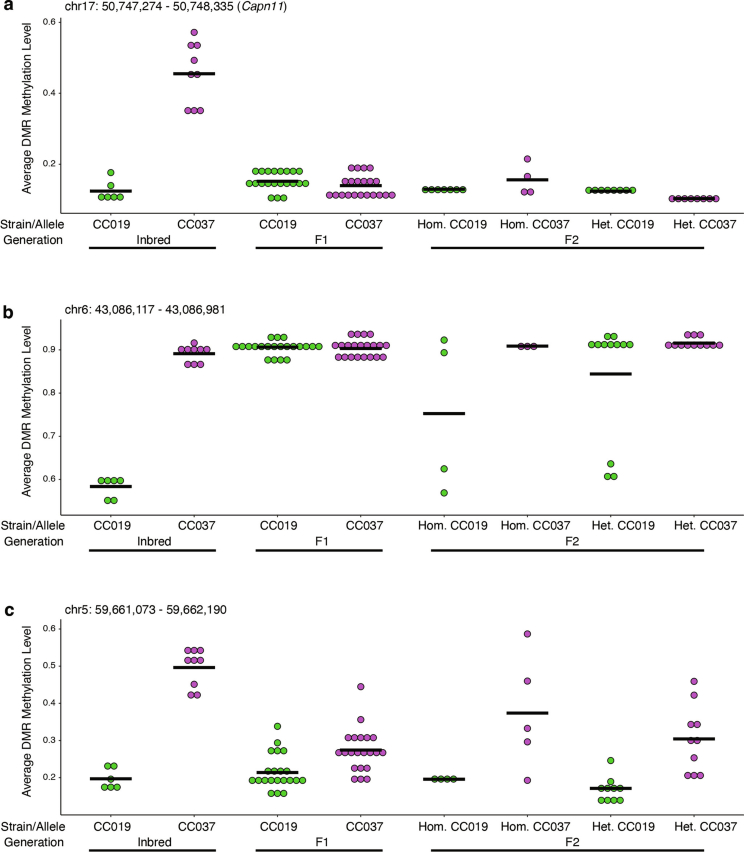
Intergenerational DNA methylation patterns of three candidate dominant *trans*-acting meQTL/transvection/paramutation regions. **a**–**c**, Average smoothed methylation in the liver over the three candidate dominant *trans*-acting meQTL/transvection/paramutation regions targeted in the F2 generation, of which only **a** is confirmed as a paramutation region, and **b** and **c** are unclassified due to the heterogeneity of methylation in the F2 generation. Data are shown across the inbred, F1 and F2 generations and split by generation and strain/allele. Only samples/alleles with at least 0.5× coverage are shown. Methylation was analyzed in 6 CC019 inbred, 9 CC037 inbred, 21 F1 and 19 F2 mice.

We observed that the candidate region overlapping the gene *Capn11* exhibits only one methylation pattern in the F2s, matching that of both F1 alleles (Fig. 5c, Extended Data Fig. 6, Supplementary Fig. 25 and Supplementary Data 1). This indicates that this region exhibits intergenerational paramutation, representing a form of non-Mendelian epigenetic inheritance observed across generations previously reported in engineered and transgenic mice. Notably, *Capn11* is a calcium-dependent protease that is predominantly expressed in the testis during later stages of meiosis in both mice and humans^25–27^, and decreased expression in humans has been associated with infertility and azoospermia^28^.

**Extended Data Fig. 7:**
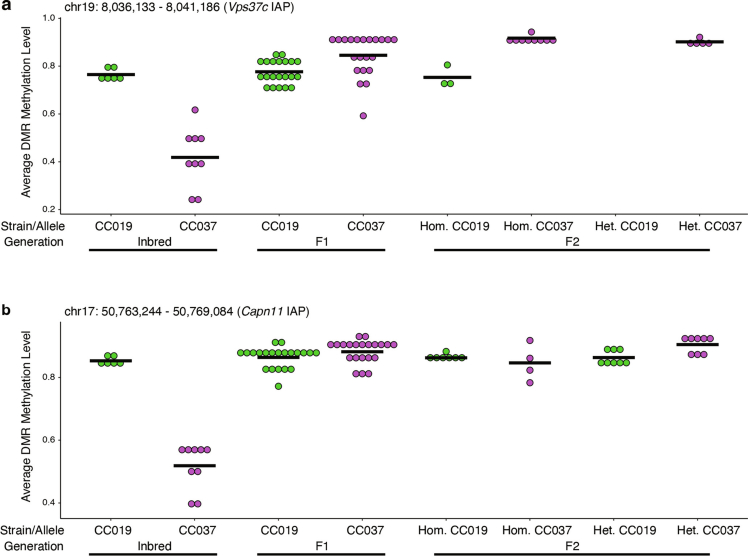
Intergenerational DNA methylation patterns of two highly likely paramutation regions. Average smoothed methylation in the liver across the two highly likely paramutation regions directly overlapping strain-specific IAPs. Data are shown across the inbred, F1 and F2 generations and split by generation and strain/allele. Only samples/alleles with at least 0.5× coverage are shown (explaining the absence of the CC019 allele in heterozygous samples in **a**). Methylation is shown for the CC019 alleles from 500 bp upstream and downstream of the IAPs, as these alleles lack the IAP. Methylation was analyzed in 6 CC019 inbred, 9 CC037 inbred, 21 F1, and 14 and 12 F2 mice for **a** and **b**, respectively.

Additionally, within the aforementioned DMR overlapping *Vps37c* (Fig. 5b), we identified a ~5-kb insertion inside the DMR relative to the CC037 reference genome. Investigation of this insertion using the Dfam database^29^ reveals that it is a transposable element (TE) belonging to the IAP family of ERVs and is present only in the CC037 strain. We suspected that this DMR may be an additional example of intergenerational paramutation, in which the highly methylated pattern observed in the CC037 alleles of the F1 generation in both liver and muscle would be subsequently inherited in all CC037 alleles of the F2 generation. While this region was not included in the F2 sequencing target regions, there exists another IAP ~13 kb downstream of the *Capn11* paramutation region^30^, which was included as a target region. The *Capn11* IAP shows sufficiently high sequence homology to the *Vps37c* IAP such that the CC037 allele was captured by adaptive sampling with enough frequency for a regional methylation analysis (average coverage of the CC037 allele with the IAP, ~8×; average coverage of the CC019 allele without the IAP, ~0.5×). As such, we reanalyzed methylation of the CC037 samples and alleles in this region using a new CC037 reference genome that includes the IAP sequence. Methylation over the *Vps37c* IAP is much lower for the inbred CC037 samples than the CC037 alleles of the heterozygous F1s in both the muscle and liver (Fig. 5d,e and Extended Data Fig. 7a). Furthermore, we observe a methylation pattern in all CC037 alleles of the F2 generation that more closely matches the F1–CC037 alleles than the inbred CC037 samples, independent of the zygosity of the F2 samples in this region, suggesting that this region is highly likely to be an additional example of intergenerational paramutation. However, as not all 19 F2 mice contain at least one copy of this IAP, there remains the possibility that this methylation pattern is driven by a dominant *trans*-acting meQTL (Supplementary Notes 1 and 2).

Given this observation, we further analyzed DNA methylation at an IAP ~13 kb downstream of the *Capn11* DMR, which is also present only in the CC037 genome. Methylation at this IAP shows a pattern similar to the *Vps37c* IAP, with inbred CC037 samples exhibiting lower methylation than CC037 alleles of the F1 generation (Fig. 5f and Extended Data Fig. 7b). Furthermore, all CC037 alleles in the F2 generation exhibit a methylation pattern nearly identical to that of the F1–CC037 alleles, strongly suggesting this is another example of intergenerational paramutation (Supplementary Notes 1 and 2).

To investigate the link between these methylation patterns and gene expression, we analyzed genes associated with these DMRs via direct promoter or enhancer overlap or genomic proximity to identify those genes whose total F1 expression levels match that of only one parental strain. This analysis revealed 14 such genes (Fig. 5g and Supplementary Tables 4 and 5), suggesting a potential role for these methylation patterns in the regulation of gene expression.

### Emergent epigenetic inheritance patterns (patterns 6–10)

**Fig. 6:**
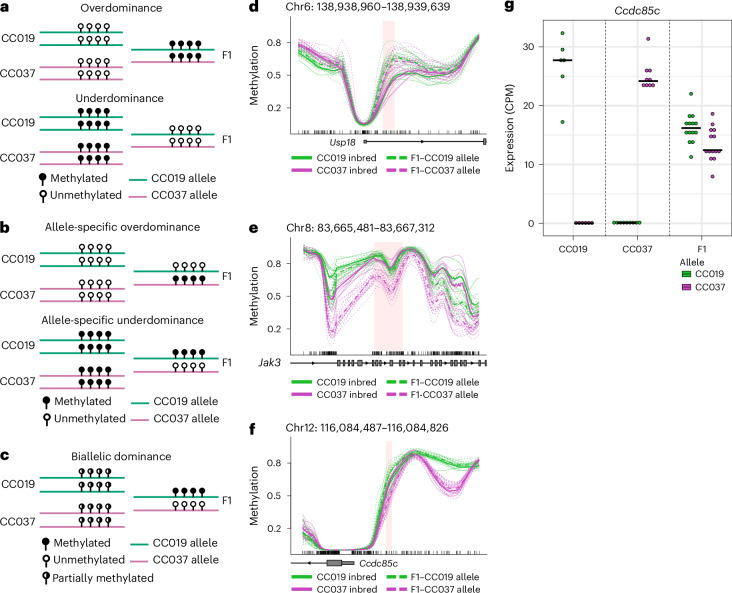
Emergent epigenetic inheritance patterns. **a**, Generic representation of methylation in the context of overdominance and underdominance in inbred CC019 and CC037 samples and their F1 crosses. **b**, Generic representation of methylation in the context of allele-specific overdominance and allele-specific underdominance in inbred CC019 and CC037 samples and their F1 crosses. **c**, Generic representation of methylation in the context of biallelic dominance in inbred CC019 and CC037 samples and their F1 crosses. **d**, Overdominant methylation pattern identified in the liver over *Usp18*. **e**, Allele-specific underdominant methylation pattern identified in the liver over *Jak3*. **f**, Biallelic dominant methylation pattern identified in the liver over *Ccdc85c*. **g**, Allele-specific expression of *Ccdc85c* in the liver, exhibiting a biallelic dominant expression pattern. The promoter of *Ccdc85c* overlaps the biallelic dominant methylation pattern shown in **f**. Expression was analyzed in 6 CC019 inbred, 9 CC037 inbred and 14 F1 mice. For the inbred samples (CC019 and CC037), the expression levels of the absent alleles are provided as technical controls confirming accurate allelic assignment. For the methylation plots shown in **d**–**f**, bold lines represent coverage-weighted mean methylation of the respective groups and CpG sites included in the final analysis are denoted by tick marks on the *x* axis.

We also searched for five distinct sets of emergent epigenetic inheritance patterns, in which at least one allele of the F1 generation exhibits a new methylation pattern not observed in either of the parental strains (for example, Fig. 6a–c). This analysis revealed 54 instances of such emergent epigenetic inheritance patterns present in at least one tissue (Fig. 2 and Supplementary Tables 2 and 3), of which one was overdominant (Fig. 6d and Supplementary Fig. 7), 22 were allele-specific overdominant or allele-specific underdominant (Fig. 6e and Supplementary Figs. 8 and 9), and 31 were biallelic dominant (Fig. 6f and Supplementary Figs. 10 and 11). This represents a substantial increase over what has been previously reported in mammals in the literature—seven examples identified in mice^31–33^ in addition to the callipyge locus in sheep^13^. Notably, only a single emergent epigenetic inheritance pattern is found in both liver and muscle, suggesting that these patterns are primarily regulated in a tissue-specific manner.

To further explore the functional role of these emergent epigenetic inheritance patterns, we analyzed the expression of genes associated with these DMRs via promoter or enhancer overlap or genomic proximity and identified 19 that exhibit expression patterns consistent with biallelic dominance (Fig. 6g and Supplementary Tables 4 and 5).

### Genomic imprinting (pattern 11)

**Fig. 7:**
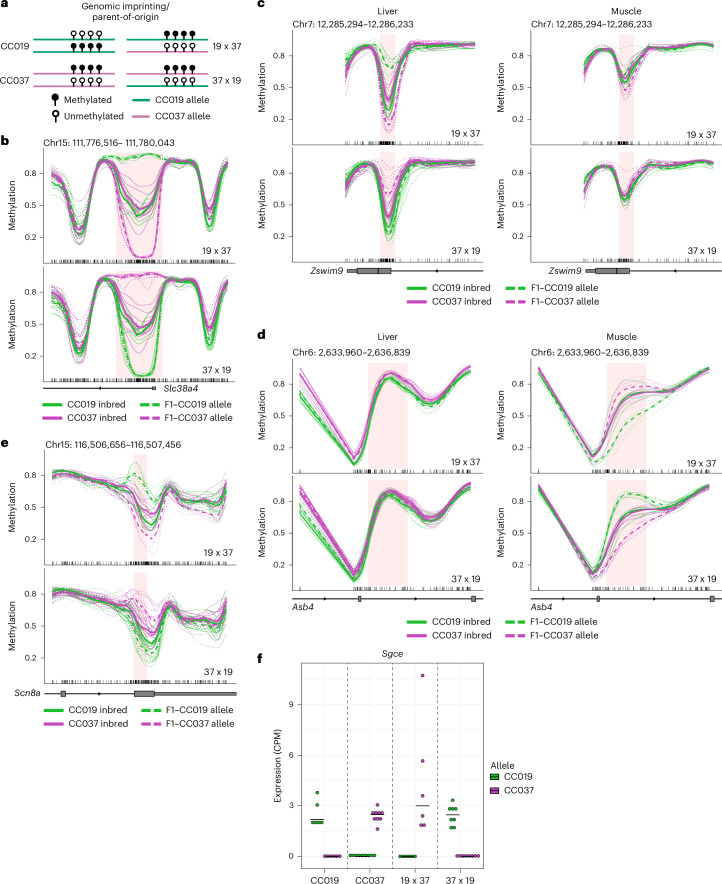
Genomic imprinting. **a**, Generic representation of methylation in the context of genomic imprinting in inbred CC019 and CC037 samples and their F1 crosses in both cross directions (denoted as maternal strain x paternal strain). **b**, Imprinted methylation pattern identified over the promoter of *Slc38a4* in the liver. Inbred samples are shown on both plots and F1 samples have been split by cross direction, CC019 x CC037 (top) and CC037 x CC019 (bottom). **c**, Parent-of-origin-specific methylation pattern found exclusively in liver, identified over *Zswim9*, shown in both the liver (left) and muscle (right). Inbred samples are shown on both plots and F1 samples have been split by cross direction, CC019 x CC037 (top) and CC037 x CC019 (bottom). **d**, Parent-of-origin-specific methylation pattern found exclusively within the muscle identified over *Asb4*, shown in both the liver (left) and muscle (right). Inbred samples are shown on both plots and F1 samples have been split by cross direction, CC019 x CC037 (top) and CC037 x CC019 (bottom). **e**, Novel parent-of-origin-specific methylation pattern identified over *Scn8a* in the liver. Inbred samples are shown on both plots and F1 samples have been split by cross direction, CC019 x CC037 (top) and CC037 x CC019 (bottom). **f**, Allele-specific expression of *Sgce* in the liver, exhibiting a parent-of-origin-specific expression pattern and overlapping a parent-of-origin-specific methylation pattern. Expression was analyzed in six CC019 inbred, nine CC037 inbred, six CC019 x CC037 F1 and eight CC037 x CC019 F1 mice. For the inbred samples (CC019 and CC037), the expression levels of the absent alleles are provided as technical controls confirming accurate allelic assignment. For the methylation plots shown in **b**–**e**, bold lines represent coverage-weighted mean methylation of the respective groups and CpG sites included in the final analysis are denoted by tick marks on the *x* axis.

Parent-of-origin-specific methylation, or genomic imprinting (Fig. 7a), is the most thoroughly studied example of non-Mendelian inheritance of DNA methylation in mammals. We identified 111 autosomal regions exhibiting parent-of-origin-specific methylation patterns in at least one tissue (Figs. 2 and 7b, Supplementary Figs. 12 and 13, and Supplementary Tables 2 and 3). We also identified a single region with parent-of-origin-specific methylation on the X chromosome in females, which is discussed further alongside the other X-chromosomal patterns. We compared this list to a curated set of known DNA methylation-regulated imprinting control regions (ICRs)^34^ to evaluate our analysis approach on known regions. Of the 13 analyzable ICRs (non-IBD, mappable), we detected imprinted methylation patterns in at least one tissue within 1 kb of all 13 regions.

In addition, we identified parent-of-origin-specific DMRs overlapping four autosomal genes that, to our knowledge, have not previously been identified as imprinted—*Scn8a*, *Pcdhb4*, *Fry* (identified in the liver) and *Socs5* (identified in the muscle). Furthermore, we identified parent-of-origin-specific methylation near four autosomal genes, which are variably reported as imprinted in the literature—*Zswim9* (ref. ^35^), *Nav2* (ref. ^36^), *Casc1* (ref. ^35^) and *Cntnap1* (refs. ^37,38^). Methylation over these imprinted genes is shown in Supplementary Fig. 26. Additionally, although not statistically significant, we observe a parent-of-origin-specific methylation pattern in muscle over *Scn8a*, which was identified in the liver, as well as a parent-of-origin-specific methylation pattern in the liver over *Socs5*, which was identified in the muscle (Supplementary Fig. 27).

Our analysis also revealed parent-of-origin-specific expression in two genes associated with these parent-of-origin-specific DMRs via promoter or enhancer overlap or genomic proximity, including the known imprinted gene *Sgce* (Fig. 7 and Supplementary Tables 4 and 5). However, another well-documented imprinted gene, *Slc38a4*, did not exhibit parent-of-origin-specific expression (Supplementary Fig. 28) despite clear parent-of-origin-specific methylation over its promoter (Fig. 7b). This finding, previously reported in adult mouse liver^39^, indicates that methylation over an ICR, while necessary to establish imprinting of certain genes, may be insufficient to cause parent-of-origin-specific expression in all cases. This suggests that additional regulatory mechanisms are involved in imprinting.

### Sex-specific DNA methylation (pattern 12)

**Fig. 8:**
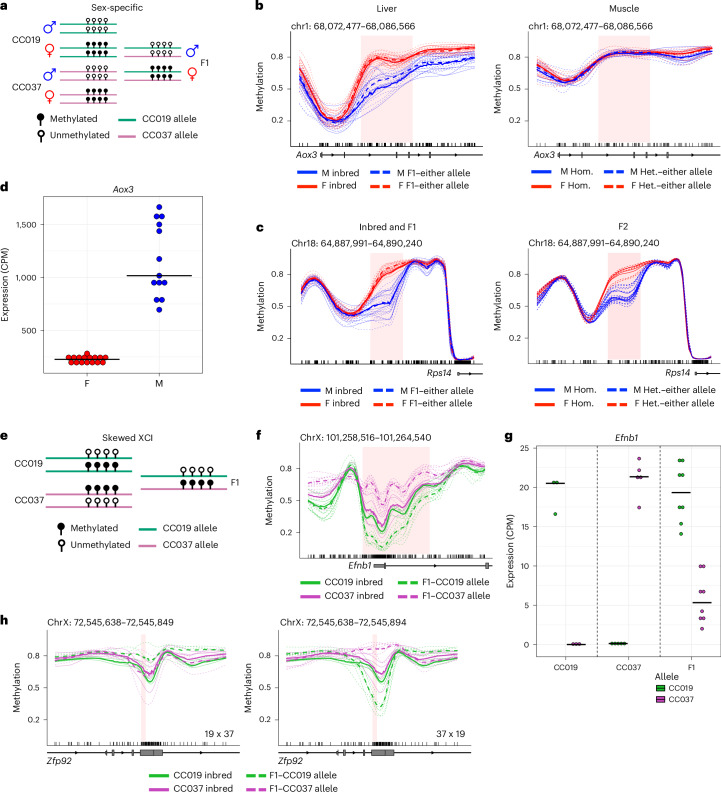
Sex-specific DNA methylation and X-chromosomal epigenetic inheritance patterns. **a**, Generic representation of sex-specific methylation in male and female inbred samples and F1 crosses. **b**, Sex-specific methylation pattern found exclusively within the liver identified over *Aox3*, shown in both the liver (left) and muscle (right). **c**, Sex-specific DMR identified in the liver upstream of *Rps14*, shown for inbred and F1 samples (left) and F2 samples (right). **d**, Total expression of *Aox3* in the liver, split by sex, exhibiting a sex-specific expression pattern and overlapping the sex-specific methylation pattern shown in **b**. Expression was analyzed in 16 female and 13 male mice. **e**, Generic representation of methylation in the context of skewed XCI in inbred CC019 and CC037 samples and their F1 crosses. **f**, Skewed XCI methylation pattern identified in the liver over the promoter of *Efnb1*. **g**, Allele-specific expression of *Efnb1* in the liver, exhibiting skewed XCI and overlapping the skewed XCI methylation pattern shown in **b**. Expression was analyzed in 6 CC019 inbred, 9 CC037 inbred and 14 F1 mice. For the inbred samples (CC019 and CC037), the expression levels of the absent alleles are provided as technical controls confirming accurate allelic assignment. **h**, New parent-of-origin-specific methylation pattern identified in the liver over *Zfp92*. Inbred samples are shown on both plots and F1 samples have been split by cross direction, CC019 x CC037 (left) and CC037 x CC019 (right). For the methylation plots shown in **b**, **c**, **f** and **h**, bold lines represent coverage-weighted mean methylation of the respective group and CpG sites included in the final analysis are denoted by tick marks on the *x* axis. M, male; F, female.

We also identified 305 autosomal regions exhibiting sex-specific methylation patterns (Figs. 2 and 8a,b, Supplementary Fig. 14, and Supplementary Tables 2 and 3). Notably, methylation differences in all but one region (304; 99.7%) show female hypermethylation relative to males. All 305 sex-specific patterns were detected in the liver, whereas none were observed in muscle. Sex-specific DNA methylation and gene expression patterns in the liver have been reported in both humans^40,41^ and mice^42^ and, as such, were expected to be less abundant in muscle.

To validate the sex specificity of these methylation patterns, we examined 20 candidate regions in the F2 generation. Sex-specific methylation is established in a manner independent of the identity of local and distal autosomal genetic variants, although an effect of X-dosage or Y chromosome cannot be ruled out. As anticipated, all 20 regions exhibited methylation patterns that segregated by sex in the F2s (Fig. 8c and Supplementary Data 1), confirming a sex-dependent regulatory mechanism.

Our analysis also identified 233 genes associated with these sex-specific DMRs that exhibit concordant sex-biased gene expression (Fig. 8d and Supplementary Tables 4 and 5). Notably, 182 genes (78.1%) showed higher expression in males, consistent with the observed methylation patterns.

### X-chromosomal epigenetic inheritance patterns

DMR finding was performed separately for the autosomes and the X chromosome. Due to expected differences between the male and female X-chromosomal methylation patterns resulting from X chromosome inactivation (XCI), two separate analyses were performed for the X chromosome—one using only female samples and the other using only the maternal alleles of male samples. XCI is a dosage compensation mechanism in which females inactivate one copy of the X chromosome in each cell. In mice, XCI has been shown to have a genetic contribution that maps to a region (QTL) on the X chromosome, within which local genetics contribute to the determination of which parental copy of the X chromosome is inactivated^43,44^. This can lead to skewed XCI, in which one X-chromosomal allele is preferentially inactivated and, thus, more highly methylated than the other allele (Fig. 8e).

For both liver and muscle, we identified few DMRs on the maternal alleles of the male samples (Supplementary Figs. 15–18 and Supplementary Tables 6 and 7), whereas we found many DMRs in the female samples, the majority of which were categorized as skewed XCI (Supplementary Figs. 19–24 and Supplementary Tables 8 and 9). Of the 218 and 226 regions indicating skewed XCI in liver and muscle, respectively, 209 (95.9% and 92.5% for liver and muscle) exhibit hypermethylation of the CC037 allele relative to CC019 (Fig. 8f, Supplementary Figs. 22 and 23, and Supplementary Tables 8 and 9). This indicates skewing of XCI toward the CC037 allele in crosses of these strains, consistent with the expected preferential inactivation of the CC037 allele relative to the CC019 allele.

**Extended Data Fig. 8:**
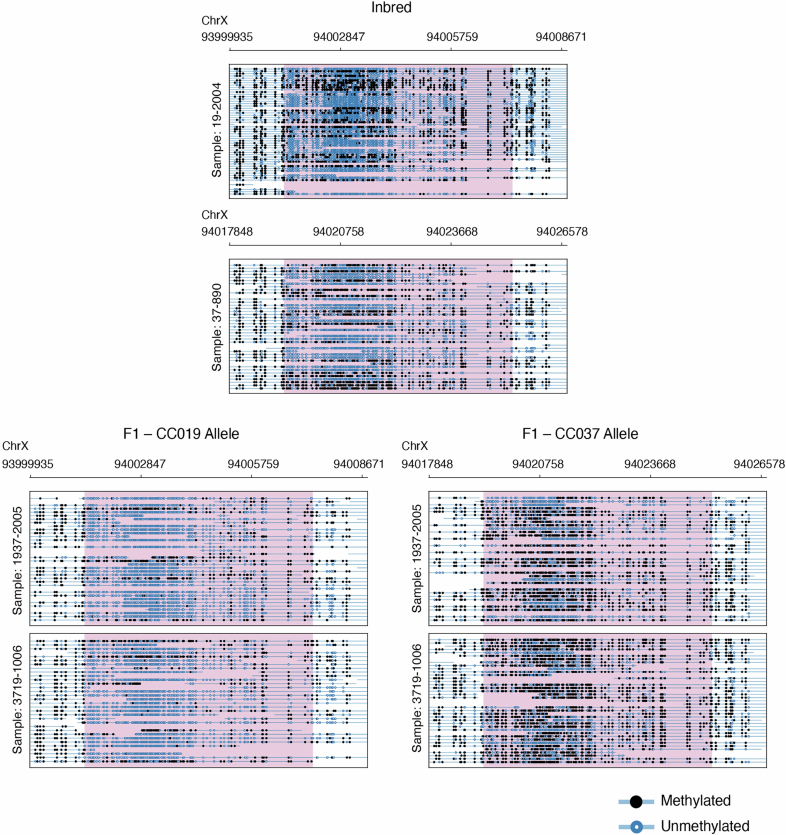
Read-level skewed XCI. Read-level methylation of a region exhibiting skewed XCI in the liver, which indicates the presence of both active and inactive alleles in the inbred samples, while showing a strong preference for the inactive X chromosome in the CC037 alleles and a strong preference for the active X chromosome in the CC019 alleles of the F1 generation. All reads overlapping this region are shown for two representative replicates per group—inbred, F1–CC019 allele and F1–CC037 allele.

**Extended Data Fig. 9:**
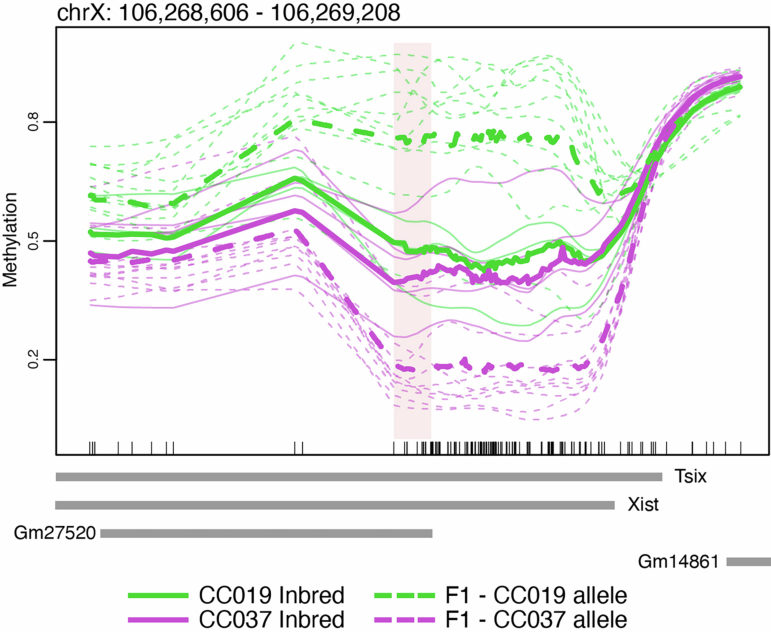
*Xist* skewed XCI. DMR is categorized as skewed XCI from the female X chromosome comparison in the liver, which overlaps the genes *Xist* and *Tsix*. Bold lines represent the coverage-weighted mean methylation of the respective group and CpG sites included in the final analysis are denoted by tick marks on the x-axis.

This pattern of skewed XCI is evident in the read-level methylation data, in which the CC037 allele of female F1 samples typically exhibits a much higher proportion of the inactive methylation pattern (hypermethylated) than the CC019 allele (Extended Data Fig. 8). Additionally, we observe hypermethylation of the active CC019 allele over *Xist* and its antisense RNA *Tsix* (Extended Data Fig. 9), key regulators of XCI known to be expressed exclusively from the inactive X chromosome^45^. Expression analysis of genes associated with these DMRs revealed that all 40 genes showing expression patterns consistent with skewed XCI exhibit higher expression from the CC019 allele, further supporting the preferential inactivation of the CC037 X-chromosomal allele (Fig. 8g and Supplementary Tables 4 and 5).

Our analysis also identified a previously unreported region with parent-of-origin-specific methylation on the X chromosome in female liver samples. This region overlaps a CpG island within the gene *Zfp92* (Fig. 8h and Supplementary Figs. 24 and 26), which is involved in the suppression of TEs^30^ and implicated in X-linked intellectual disability^46,47^ and nonobstructive azoospermia^48^. Analyses of disorders including Turner syndrome and autism spectrum disorder have suggested the existence of an imprinted locus on the X chromosome affecting cognitive function^49,50^, although such a locus has not previously been identified. Furthermore, while not statistically significant, we also observe parent-of-origin-specific methylation in this region within female muscle samples (Supplementary Fig. 27).

## Discussion

Here we show that the intergenerational inheritance of DNA methylation patterns exhibits extensive complexity beyond what is currently recognized in mammals. While our analysis identified these DNA methylation patterns via a comparison of only two mouse strains, they are observed genome-wide in the crosses of two recombinant-inbred CC strains, which are themselves mosaics of eight distinct founder strains. As such, these patterns are not limited to alleles of a specific pair of strains. Additionally, there are likely additional loci in the mouse genome that exhibit these complex epigenetic inheritance patterns but could not be discovered using the allele combinations of only two CC strains. Additionally, despite pairs of CC strains having a smaller fraction of IBD genome than classical laboratory strains^51,52^, we were unable to phase methylation across almost one-third of the genome, which likely contains additional loci exhibiting these complex inheritance patterns. Moreover, the present study analyzed these epigenetic inheritance patterns in two tissues and under stable environmental conditions; consideration of additional tissues and environments is likely to increase the abundance of these complex inheritance patterns.

Notably, each of the regions we identified as exhibiting intergenerational paramutation has associations with TEs of the IAP family, with two of the three regions directly overlapping IAP elements. This suggests that the methylation-based repression of these IAPs may be mechanistically linked with the observed paramutation events. IAPs, the subclass of ERV associated with the *agouti* locus^19^, are subject to complex epigenetic regulation. DNA methylation of IAP elements can be influenced not only by genetic effects but also by parent-of-origin and environmental exposures^18^. Previous studies have further identified an IAP under the regulation of a polymorphic cluster of dominant *trans*-acting KRAB zinc finger proteins^53^. Furthermore, IAPs can be protected from the genome-wide demethylation events that occur during gametogenesis and embryogenesis^54^. Here we present the examples of IAPs associated with paramutation in naturally occurring mammalian genomes, a form of non-Mendelian epigenetic inheritance observed across generations previously reported in engineered and transgenic mice.

Similar allele-specific epigenomic studies conducted in human families and populations will help clarify the conservation of these epigenetic inheritance patterns across species as well as their impact on phenotypes and disorders. To date, a comprehensive analysis of epigenetic inheritance in humans has not been performed, largely due to difficulties controlling confounding factors such as environmental exposures and age, as well as the greater genetic variation in human populations compared with model organisms. Additionally, the combination of our allele-specific analysis with phenotypic measurements may improve our understanding of the link among genetics, epigenetics and phenotype. The use of allele-specific expression and DNA methylation has long been recognized as promising by us and others^55–57^, but it has not been clear how to perform this type of analysis at the genome scale. As we have now shown that epigenetic variants can be comprehensively phased across the genome, this study opens the door to allele-specific epigenome-wide association studies, in which phased allele-specific DNA methylation patterns are associated with a phenotype or disorder of interest. This method could identify new phenotype-associated genes missed by traditional genome-wide and epigenome-wide association studies (Supplementary Fig. 29), which independently consider only genetic or epigenetic trait associations, respectively.

Finally, the large number of non-Mendelian epigenetic inheritance patterns identified here has potentially important implications in evolution. In particular, emergent epigenetic inheritance patterns can generate diversity in the absence of mutation and may contribute to phenomena such as hybrid vigor and hybrid dysgenesis, as well as the emergence of other new traits within hybrid strains. Furthermore, these emergent epigenetic inheritance patterns may provide a mechanism supporting genetic assimilation—a key hypothesis proposed by Waddington, not yet substantiated—in which the environment introduces an epigenetic perturbation that eventually becomes genetically fixed, providing a survival advantage. Notably, 42 of the 54 autosomal emergent epigenetic inheritance patterns (78%) exhibited de novo methylation in at least one F1 allele. As methylated cytosines are mutagenic to C → T transitions, with mutation rates up to threefold higher than unmethylated bases^58^, the ‘genetic fixation’ aspect of Waddington’s hypothesis might involve the acquisition of mutated bases at alleles acquiring this de novo methylation, thereby preventing reversion to the original phenotype. This model is particularly intriguing in the context of IAPs associated with some non-Mendelian epigenetic inheritance patterns, as IAPs are a known source of new mutations in the mouse genome^59^ and their transposition is modulated by their epigenetic state^60^.

## Methods

### Experimental procedures

All experiments were performed in accordance with and approved by the Texas A&M University Institutional Animal Care and Use Committee (protocol 2022-0273).

#### Animal, housing and genotyping

Collaborative Cross^61^ lines CC019/TauUnc and CC037/TauUnc were sourced from the Systems Genetics Core Facility at the University of North Carolina at Chapel Hill and underwent breeding and maintenance at Texas A&M University. F1 mice were generated by crossing CC019/TauUnc females with CC037/TauUnc males (CC019 x CC037) and CC037/TauUnc females with CC019/TauUnc males (CC037 x CC019). The F1 mice were subsequently intercrossed to produce three distinct F2 populations—((CC019 x CC037) x (CC019 x CC037)), ((CC019 x CC037) x (CC037 x CC019)) and ((CC037 x CC019) x (CC037 x CC019)). For details, see Supplementary Methods. Genotyping was performed using the MiniMUGA genotyping array (Neogen)^62^.

From the 58 genotyped F2 mice, we selected a subset of 19 samples for targeted ONT sequencing which satisfied two conditions as follows: (1) for each of the targeted candidate dominant *trans*-acting meQTL/transvection/paramutation regions, there were at least five heterozygous samples and three homozygous samples for each parental allele, CC019 and CC037, as determined by the SNPs immediately flanking each region; and (2) for each region in the genome, there was at least one sample homozygous for each parental strain (Extended Data Fig. 5a,b). The second condition ensured that, for each genomic region, we sequenced at least one F2 homozygous for CC019 and at least one F2 homozygous for CC037. This allows us to distinguish paramutation from dominant *trans*-acting meQTLs in the F2 generation. To perform this selection, we filtered the 11,125 SNPs on the MiniMUGA genotyping array to include only those that could be mapped to the intermediate genome, were successfully genotyped across all 19 selected F2s and were homozygous and divergent between the CC019 and CC037 parental strains. Three additional SNPs were removed upon manual inspection, resulting in 2,455 SNPs for analysis.

ONT sequencing was performed on liver DNA from 6 CC019 inbred samples (3 males and 3 females), 9 CC037 inbred samples (4 males and 5 females), 9 CC019 x CC037 F1 crosses (4 males and 5 females), 13 CC037 x CC019 F1 crosses (6 males and 7 females) and 19 F2 crosses (12 males and 7 females), as well as muscle DNA from 5 CC019 inbred samples (2 males and 3 females), 6 CC037 inbred samples (3 males and 3 females), 6 CC019 x CC037 F1 crosses (3 males and 3 females) and 6 CC037 x CC019 F1 crosses (3 males and 3 females). One female CC019 x CC037 F1 liver sample was removed from subsequent methylation and expression analyses as phasing of the X chromosome revealed only one parental allele, indicating monosomy of the X chromosome (X0). Liver and muscle were chosen due to their relatively high degree of cellular homogeneity, with hepatocytes representing ~60% of the cells and ~80% of the total mass of the liver^63^ and type I and II myofibers accounting for ~70% of the nuclei in the muscle^64^.

Matched RNA-seq was performed on liver RNA from six CC019 inbred (three males and three females), nine CC037 inbred (four males and five females), nine CC019 x CC037 F1 crosses (four males and five females) and nine CC037 x CC019 F1 crosses (four males and five females). Three additional samples were removed from subsequent expression analysis—one male CC019 x CC037 was removed due to incorrect sequencing read depth (~360 million compared to 50 million targeted), one female CC019 x CC037 F1 sample was removed as an outlier (total expression >3 s.d. from the mean in 6,927/12,894 expressed genes) and one male CC037 x CC019 F1 sample was removed due to poor mapping (68.8%) to the diploid transcriptome.

#### High-molecular-weight DNA extraction

This was performed using ~20 mg of flash-frozen median liver lobe with the Nanobind Tissue Kit (PacBio, SKU 102-302-100) and ~35 mg of flash-frozen right femoral muscle using the Nanobind PanDNA Kit (PacBio, SKU 103-260-000). Homogenization was performed using the TissueRuptor II (Qiagen, 9002755) at maximum speed for 10 s.

#### DNA library preparation and ONT sequencing

Whole-genome ONT sequencing of the liver samples was performed in two batches. For batch 1 of the liver samples, 2 μg of high molecular weight (HMW) DNA diluted to 70 μl with nuclease-free water was sheared to ~10 kb using the Megaruptor 2 (Diagenode, B06010002) with an additional AMPure XP (Beckman Coulter, A63881) cleanup. Samples were eluted into a final volume of 50 μl of nuclease-free water. All remaining DNA after shearing, cleanup and quantification was used as input for library preparation. Libraries were prepared using the SQK-LSK109 ligation sequencing kit (ONT).

For batch 2 of the liver samples, 3.25 μg of HMW DNA at a concentration of 50 ng μl^−1^ was sheared using the Megaruptor 3 (Diagenode, B06010003) at speed 29, resulting in N50s in the range of 20–30 kb. All remaining DNA after shearing and quantification was used as input for library preparation using the SQK-LSK110 ligation sequencing kit (ONT) with the following changes which improve the recovery of long reads: SPRIselect beads (Beckman Coulter, B23318) instead of AMPure XP beads; the duration of all incubation and elution steps performed at room temperature or 37 °C were tripled; during DNA repair and end-prep, thermocycler incubation was performed at 20 °C for 10 min and then 65 °C for 10 min; and LFB was used during adapter ligation and cleanup.

For muscle samples, library preparation and sequencing were performed following the same protocol as batch 2 of the liver samples, with the following changes: libraries were prepared using the SQK-LSK114 ligation sequencing kit (ONT) with the same long-read recovery adjustments as in batch 2 of the liver samples, ONT sequencing was performed using R10.4.1 flow cells, and flow cell priming and reloading were performed using the Flow Cell Priming Kit V14 (EXP-FLP004) and Sequencing Auxiliary Vials V14 (EXP-AUX003).

Details of ONT sequencing are provided in Supplementary Methods. As liver ONT sequencing was performed in two distinct batches, a check for potential batch-specific DMRs was performed (described below). The median coverage across both alleles for the whole-genome ONT sequencing of the inbred and F1 generations was 24× (range = 8× to 41×), while the median coverage across both alleles over the target regions in the F2 generation was 68× (range = 56× to 94×). Details of targeted ONT sequencing of the F2 samples are provided in Supplementary Methods.

RNA extraction, library preparation and sequencing are described in Supplementary Methods.

### Statistics and reproducibility

No statistical method was used to predetermine sample size. One expression sample was excluded due to low coverage; the remaining samples passed quality control. Experiments were not randomized. Investigators were not blinded to allocation during experiments and outcome assessment.

### Computational procedures

#### Overall summary of methylation processing and analysis

To process and analyze our sequencing data, we developed a computational pipeline optimized for phasing and characterization of allele-specific methylation in genetically divergent mouse strains (Extended Data Fig. 1). Interstrain analyses of sequencing data can be severely hampered by strain and reference biases^22^. We therefore performed alignment and phasing relative to strain-specific genomes and extracted only consensus phasing decisions that are consistent irrespective of the chosen reference genome. Methylation data were then mapped to a pseudohybrid intermediate genome that incorporates genetic variation from both the CC019 and CC037 genomes, for subsequent analysis. The use of the intermediate genome mitigates bias arising from the choice of a single reference genome while maintaining strain-specific methylation information, which has been shown to increase statistical power in interstrain comparisons of DNA methylation in isogenic mouse models^22^.

Briefly, ONT sequencing data for all samples were aligned and phased against both the CC019 and CC037 genomes and only consensus phasing decisions, reads for which the allelic assignment was consistent independent of the reference genome, were used. Allele-specific methylation information was extracted from these reads and converted into a common coordinate system for analysis. DMR finding was run using data from the inbred and F1 generations to identify genomic regions in which DNA methylation exhibits at least 1 of the 12 intergenerational epigenetic inheritance patterns analyzed here. DMR finding was performed separately for liver and muscle, and separately within each tissue for the autosomes, X chromosomes in female samples and maternal X-chromosomal alleles in male samples. After identifying this set of genome-wide significant DMRs, the DMRs were filtered for quality control and post hoc categorized to best match them to an inheritance pattern using methylation differences between the relevant groups. A subset of these DMRs was targeted for sequencing in the F2 generation to further validate and determine the effects of *cis-*acting and *trans*-acting genetic variants on these methylation patterns.

#### ONT sequencing processing

As ONT sequencing of the liver and muscle samples was performed using different versions of flow cells and library kits, the data were processed using different pipelines. Liver samples, sequenced on R9.4.1 flow cells using the LSK109/LSK110 ligation sequencing kits, were basecalled with Guppy and methylation was called using Nanopolish. Canonical and modified basecalling for muscle samples, sequenced on R10.4.1 flow cells, was performed using Dorado and methylation information was subsequently extracted using modkit. The use of multiple techniques for basecalling and methylation calling across these tissues allows for a limited degree of validation that the identified patterns do not arise due to technical artifacts introduced by these processes. Furthermore, methylation calling (liver) and methylation extraction (muscle) were performed using the reference genome of the strain/allele being considered.

#### Collaborative Cross Graphical Genome

Genome sequence and anchor information for CC019 and CC037 were extracted from the Collaborative Cross Graphical Genome^65^ (v2.0) using its API.

#### Intermediate genome and coordinate conversion

The use of multiple strain-specific reference genomes necessitates mapping of genomic feature coordinates across genomes. Furthermore, the ability to map coordinates from mm10 to our strain-specific reference genomes is crucial to handling annotations. To further minimize the introduction of reference bias into our data, we performed all methylation analyses in a pseudohybrid intermediate genome created from the CC019 and CC037 reference genomes, rather than picking one of these two genomes as a basis for the analysis. This intermediate genome was created by constructing the maximal-length sequences from the alignment between CC019 and CC037 for each edge. This intermediate genome includes all CC019-specific and CC037-specific insertions represented and any coordinate that exists in either strain-specific genome necessarily exists in the intermediate genome. To do this, we developed a suite of custom tools for the creation of the pseudohybrid intermediate genome and for mapping between the coordinate systems.

#### Identification of IBD regions

IBD regions between CC019 and CC037 were defined as those that cannot be phased using 25 kb reads. Within the CC019 and CC037 genomes, there are 19,258,931 and 19,417,691 CpGs, respectively. Of these, 17,911,438 and 17,969,433 can be mapped into the intermediate genome and 4,773,219 and 4,778,508 overlap IBD regions. Additionally, of the 16,966,618 common CpGs that can be mapped to the intermediate genome, 4,718,665 (27.8%) overlap IBD regions, leaving 11,873,848 autosomal and 374,105 X-chromosomal common, non-IBD CpGs that can be mapped to the intermediate genome.

#### DNA methylation analysis

For the inbred and F1 liver dataset, a single female CC019 x CC037 F1 sample was removed from subsequent methylation and expression analyses as phased ONT reads of the X chromosome were assigned to only one allele and the coverage ratio of the X chromosome to the autosomes was roughly half that of other female samples, indicating that this sample was X0 and thus only contains one copy of the X chromosome. This phenomenon has been reported in inbred mice^66^.

Methylation data were processed in R (v4.3.1) using the ‘bsseq’^67^ package (v1.38.0). Methylation values were smoothed using the BSmooth function from ‘bsseq’. This function smooths methylation data for each sample individually to decrease methylation variance and facilitate the identification of DMRs, which are more commonly associated with functionally relevant methylation changes than single-CpG differences^67^. Furthermore, methylation smoothing allows for a better tradeoff between coverage and sample size^68^.

Smoothed methylation data were subsequently subset into three groups—autosomal, X chromosome female samples and X chromosome maternal allele of male samples, as proper analysis of the X chromosome necessitates a more in-depth consideration of sex imbalance due to XCI and because male samples only have maternal X chromosomes. Within each of these groups, the BS objects were subset to include only those CpGs that are covered in at least 75% of the CC019 inbred samples and CC019 phased F1 alleles, as well as in at least 75% of the CC037 inbred samples and CC037 phased F1 alleles. This effectively removes strain-specific CpGs from the analysis, although the methylation values of strain-specific CpGs have been used to impute methylation values over strain-agnostic CpGs during smoothing.

The following number of CpGs were analyzed for each of the conditions: liver autosomal, 11,501,000; muscle autosomal, 11,903,947; liver X chromosome female, 341,941; muscle X chromosome female, 352457; liver X chromosome male maternal allele, 324,311 and muscle X chromosome male maternal allele, 346,487.

DMR finding was performed using an F-statistic test, which allows a genome-wide search for multiple distinct DNA methylation patterns using a single test. This identifies genomic regions in which one or more of these contrasts is different from zero while controlling the family-wise error rate. Following the identification of DMRs, we do post hoc categorization, described below. We considered 13 different contrasts (Supplementary Methods).

We performed a genome-wide search using a single F-statistic on the autosomes, which simultaneously tests for these 13 contrasts. We supplemented this search with an additional search across the X chromosome for female samples (excluding one contrast) and an additional search across the X chromosome for the maternal allele of male samples (excluding five contrasts). Briefly, our approach identifies DMRs passing the *F*-statistic cutoff and then performs permutation testing to adjust for multiple testing and control the family-wise error rate.

The three lists of output DMRs and smoothed BS objects were used to calculate smoothed methylation differences between groups defined in the contrast matrices used for testing, as well as in eight additional contrasts. These groupwise methylation differences were used to assign each statistically significant DMR to a category. The defined categories are mutually exclusive and, as such, each DMR is assigned to only one. However, because these methylation patterns do not necessarily function independently of one another, additional categories were defined that exhibit characteristics of multiple inheritance patterns, as well as one for those DMRs that cannot be clearly categorized. DMRs assigned to these additional categories were not considered in subsequent analyses. After categorization, a final filter step was implemented to ensure that sufficient raw methylation differences were present over identified DMRs.

Whole-genome ONT sequencing of the inbred and F1 liver samples was completed in two batches with differing library preparation kits (batch 1, LSK109; batch 2, LSK110) and target read lengths (batch 1, 10 kb; batch 2, 20–30 kb), factors which may influence alignment, phasing and base and methylation calling. Although each batch includes inbred samples from each strain as well as heterozygous F1 crosses, ensuring that none of the autosomal methylation comparisons are fully confounded by batch, we searched our autosomal DMRs to identify regions that may still be influenced by batch effects. Those DMRs for which the relevant methylation difference without the inclusion of the batch 1 samples is less than half of the relevant methylation difference with all samples included are noted as potentially influenced by sequencing batch. Of the 6,970 autosomal DMRs identified in the liver, only 29 (0.4%) fall into this category (Supplementary Table 2). This additional check was not performed for the X chromosome analyses as all male inbred CC019 samples originated from batch 1.

#### Analysis of methylation over the *Vps37c* and *Capn11* IAPs

Analysis of methylation over each of these CC037-specific IAPs requires additional reprocessing, as the *Vps37c* IAP is not present in the CC037 reference genome and coordinates within the *Capn11* IAP—although present within the CC037 reference genome—cannot be mapped into the intermediate genome, as they reside within an edge that is longer than 3 kb. Additionally, due to the high degree of sequence similarity among these IAPs, MiniMap2 assigns the sections of these reads that directly overlap the IAPs missing from the reference genome as supplementary alignments and incorrectly places them over an IAP that is present within the reference genome, in this case, the *Capn11* IAP. Therefore, our analysis of these regions is customized to the challenges involved in each region and is detailed in Supplementary Methods.

#### RNA-seq preprocessing

RNA sequencing reads were processed using a modified version of the SEESAW pipeline^69^. One male CC037 x CC019 F1 sample exhibited poor mapping to the diploid transcriptome (68.8% mapping rate) and was removed from subsequent expression analysis.

#### Expression analysis

Allelic expression values were calculated using the ‘fishpond’^70^ package (v2.8.0). For each sample, allelic counts were converted to counts per million. For subsequent allelic analyses, an additional level of filtering was applied, using allelic counts from the inbred parental strains, to identify genes with accurate allelic assignments. For the analysis of XCI-associated genes, the above filtering steps were performed on female samples only. Owing largely to the limited number of transcribed polymorphisms between the two strains analyzed, we were able to analyze allele-specific expression for only 9.5% of genes in the genome.

To gauge the effect of batch on the RNA-seq data, principal component analysis plots showing the allele-specific expression of autosomal genes with correct allelic assignment and expression above the aforementioned cutoff for the six inbred samples which were included in both RNA-seq batches, split by both batch and allele, are shown in Supplementary Fig. 30. Clear clustering of these samples by sample and allele, rather than batch, suggests that any batch effect is smaller than the biological differences between samples of different strains.

The allelic counts of the filtered genes, using only inbred samples from a single batch, were analyzed with the ‘swish’ function from the ‘fishpond’ package using one of two methods as follows: (1) the default global allelic imbalance analysis or (2) a differential allelic imbalance analysis. One female CC019 x CC037 F1 sample had a total expression count per million >3 s.d. from the mean across 6,927 of 12,894 expressed genes and was therefore removed from subsequent expression analyses as an outlier.

#### Mouse Genome Informatics gene-disease associations

Gene-disease associations were extracted from the following Mouse Genome Informatics databases: Associations of Mouse Genes with DO Diseases and Mouse Models of Human Disease by Human Gene^71^. Associations are described in Supplementary Notes and Supplementary Tables 11 and 12.

### Reporting summary

Further information on research design is available in the Nature Portfolio Reporting Summary linked to this article.

## Online content

Any methods, additional references, Nature Portfolio reporting summaries, source data, extended data, supplementary information, acknowledgements, peer review information; details of author contributions and competing interests; and statements of data and code availability are available at 10.1038/s41588-026-02604-z.

## Supplementary information

Supplementary Information Supplementary Methods, Notes 1 and 2, Discussion, Glossary, and Figs. 1–30.

Reporting Summary

Supplementary Tables 1–4 and 6–10 Supplementary Table 1: ONT sequencing information. Supplementary Table 2: Liver autosomal DMRs. Supplementary Table 3: Muscle autosomal DMRs; Supplementary Table 4: Liver gene expression summary. Supplementary Table 6: Liver chrX male DMR information. Supplementary Table 7: Muscle chrX male DMR information. Supplementary Table 8: Liver chrX female DMR information. Supplementary Table 9: Muscle chrX female DMR information. Supplementary Table 10: F2 target regions.

Supplementary Table 5 Liver gene expression.

Supplementary Table 11 Liver DMR-associated gene-phenotype relationships.

Supplementary Table 12 Muscle DMR-associated gene-phenotype relationships.

Supplementary Data 1 F2 candidate region methylation. Liver methylation from the F2 generation over the dominant *trans*-acting meQTL/transvection/paramutation DMRs, *cis*-acting meQTL DMRs, nondominant *trans*-acting meQTL DMRs, and sex-specific DMRs chosen for targeted analysis in the F2s. Bold lines represent coverage-weighted mean methylation of the respective group and CpG sites included in the final analysis are denoted by tick marks on the *x*-axis.

## Data Availability

All ONT and RNA-seq FASTQ files have been deposited in the NIH Sequence Read Archive (BioProject: PRJNA1107693). Methylation frequency files, Guppy sequencing summary files, Salmon quantification files and allele-specific gene counts have been deposited in the NCBI Gene Expression Omnibus with accession GSE266668. F2 genotyping data have been deposited in Zenodo and are available at 10.5281/zenodo.17148323 (ref. ^72^).

## Code availability

The processing and analysis code used in this paper is available on Zenodo at 10.5281/zenodo.17727457 (ref. ^73^).
