# Supplementary material for: Non-Mendelian inheritance of DNA methylation patterns in mice
Source: Nat Genet. 2026 May 20;58(6):1409–22. doi: 10.1038/s41588-026-02604-z (PMC13263155; doi:10.1038/s41588-026-02604-z)
Supplement: Supplementary file 1 — Supplementary Methods, Notes 1 and 2, Discussion, Glossary, and Figs. 1–30. [file 41588_2026_2604_MOESM1_ESM.pdf]

nature genetics

Supplementary information https://doi.org/10.1038/s41588-026-02604-z

Non-Mendelian inheritance of DNA methylation patterns in mice

In the format provided by the authors and unedited

Contents

Supplementary Methods 2
Experimental Procedures . . . 2
Statistics & Reproducibility . . . 5
Computational Procedures . . . 5

Supplementary Note: On various intergenerational epigenetic inheritance patterns 24

Supplementary Note: Additional Results 27

Supplementary Discussion 31

References 32

Glossary of Terms 37

Supplementary Data 40

Supplementary Figures 41

Supplementary Methods

The following represents the complete methods for the manuscript. Due to space constraints, the Online Methods are an abridged version of this material

Experimental Procedures

All experiments were performed in accordance and approval by Texas A&M University Institution of Animal Care and Use Committee (IACUC 2022-0273).

Animal, housing, and genotyping

Collaborative Cross [1] lines CC019/TauUnc and CC037/TauUnc were sourced from the Systems Genetics Core Facility at the University of North Carolina at Chapel Hill and underwent breeding and maintenance at Texas A&M University. F1 mice were generated by crossing CC019/TauUnc females with CC037/TauUnc males (CC019 x CC037) and CC037/TauUnc females with CC019/TauUnc males (CC037 x CC019). The F1 mice were subsequently intercrossed to produce three distinct F2 populations: [(CC019 x CC037) x (CC019 x CC037)], [(CC019 x CC037) x (CC037 x CC019)], and [(CC037 x CC019) x (CC037 x CC019)]. These Collaborative Cross strains do not carry the agouti viable yellow (Avy) allele.

All mice, including parental lines, F1 progeny, and the F2 offspring, were housed at a temperature of $22 \pm 2°C$ under 12h light/12h dark cycle with unrestricted access to food and water, and were euthanized at approximately 4 months ($\pm$1 month) of age, with the exception of seven inbred mice from which muscle samples were sequenced which were euthanized at approximately 2.5 months of age. Liver biopsies were obtained from mice post-euthanasia and were used for genotyping the parental CC lines, F1, and F2 populations with the MiniMUGA genotyping array (Neogen, MI) [2]. Sex was assigned using morphological characteristics and confirmed by genotyping. The remaining liver tissue was flash-frozen in liquid nitrogen prior to processing for ONT and RNA sequencing (RNA-seq). Right femoral muscle was also collected from mice post-euthanasia, rinsed with 1x PBS, and flash-frozen in liquid nitrogen prior to processing for ONT sequencing.

From the 58 genotyped F2 mice, we selected a subset of 19 samples for targeted ONT sequencing which satisfied the following two conditions: (1) for each of the targeted candidate dominant trans-acting meQTL/transvection/paramutation regions, there were at least five heterozygous samples and three homozygous samples for each parental allele, CC019 and CC037, as determined by the SNPs immediately flanking each region; and (2) for each region in the genome, there was at least one sample homozygous for each parental strain (Extended Data Fig. 5a-b). The second condition ensured that, for each region in the genome, we sequenced at least one F2 which is homozygous CC019 and at least one F2 which is homozygous CC037. This allows us to distinguish paramutation from dominant trans-acting meQTLs in the F2 generation. To perform this selection, we filtered the 11,125 SNPs on the MiniMUGA genotyping array to include only those that could be mapped into the intermediate genome, were successfully genotyped in all 19 selected F2s, and were homozygous and divergent between the CC019 and CC037 parental strains. Three additional SNPs were removed upon manual inspection, resulting in 2,455 SNPs for analysis.

ONT sequencing was performed on liver DNA from 6 CC019 inbred samples (3 males and 3 females), 9 CC037 inbred samples (4 males and 5 females), 9 CC019xCC037 F1 crosses (4 males and 5 females), 13 CC037xCC019 F1 crosses (6 males and 7 females), and 19 F2 crosses (12 males and 7 females) as well as muscle DNA from 5 CC019 inbred samples (2 males and 3 females, 6 CC037

inbred samples (3 males and 3 females), 6 CC019xCC037 F1 crosses (3 males and 3 females), and 6 CC037xCC019 F1 crosses (3 males and 3 females). One female CC019xCC037 F1 liver sample was removed from subsequent methylation and expression analyses as phasing of the X chromosome revealed only one parental allele, indicating monosomy of the X chromosome (X0). Liver and muscle were chosen due to their relatively high degree of cellular homogeneity, with hepatocytes representing 60% of the cells and 80% of the total mass of the liver23 and type I and II myofibers accounting for 70% of the nuclei in the muscle24.

Matched RNA-seq was performed on liver RNA from 6 CC019 inbred (3 males and 3 females), 9 CC037 inbred (4 males and 5 females), 9 CC019xCC037 F1 crosses (4 males and 5 females), and 9 CC037xCC019 F1 crosses (4 males and 5 females). Three additional samples were removed from subsequent expression analysis: one male CC019xCC037 was removed due to incorrect sequencing read depth (360M compared to 50M targeted), one female CC019xCC037 F1 sample was removed as an outlier (total expression of the outlier was more than 3 standard deviations away from the mean in 6,927/12,894 expressed genes), and one male CC037xCC019 F1 sample was removed due to poor mapping to the diploid transcriptome (68.8% mapping rate).

High molecular weight DNA extraction

High molecular weight (HMW) DNA was extracted from 20mg of flash-frozen median liver lobe using the Nanobind Tissue Kit (PacBio SKU 102-302-100) and 35mg of flash-frozen right femoral muscle using the Nanobind PanDNA Kit (PacBio SKU 103-260-000) following to the manufacturer's instructions. Homogenization was performed using the TissueRuptor II (Qiagen Cat. 9002755) at maximum speed for 10s.

DNA library preparation and ONT sequencing

Whole-genome ONT sequencing of the liver samples was performed in two batches. For batch 1 of the liver samples, $2\mu g$ of HMW DNA diluted to $70\mu L$ with nuclease-free water was sheared to $\sim 10kb$ using the Megaruptor 2 (Diagenode Cat. B06010002) with an additional AMPure XP (Beckman Coulter Cat. A63881) cleanup. Samples were eluted into a final volume of $50\mu L$ of nuclease-free water. All remaining DNA after shearing, cleanup, and quantification was used as input for library preparation. Libraries were prepared using the SQK-LSK109 Ligation Sequencing Kit (ONT) following the manufacturer's instructions. ONT sequencing was performed using a PromethION 24 and R9.4.1 flow cells (ONT Cat. FLO-PRO002). Sequencing was run for 72 hours. Batch 1 contains samples from all relevant groups except the 19x37 F1 cross direction.

For batch 2 of the liver samples, $3.25\mu g$ of HMW DNA at a concentration of 50 $ng/\mu L$ was sheared using the Megaruptor 3 (Diagenode Cat. B06010003) at speed 29 resulting in N50s in the range of 20-30kb. All remaining DNA after shearing and quantification was used as input for library preparation. Libraries were prepared using the SQK-LSK110 Ligation Sequencing Kit (ONT) following the manufacturer's instructions with the following changes which improve the recovery of long reads: SPRIselect beads (Beckman Coulter Cat. B23318) were used instead of AMPure XP beads; the duration of all incubation and elution steps performed at RT or $37°C$ were tripled; during DNA repair and end-prep, thermocycler incubation was performed at $20°C$ for 10 minutes and then $65°C$ for 10 minutes; and LFB was used during adapter ligation and clean-up. ONT sequencing was performed using a PromethION 24 and R9.4.1 flow cells. Sequencing was stopped manually at the end of the life of the flow cells, which were washed and reloaded once or twice depending on the amount of library prepared, flushing when 20% of total pores were remaining.

Flow cell washing, priming, and reloading were performed using the following kits: Flow Cell Wash Kit (EXP-WSH004), Flow Cell Priming Kit (EXP-FLP002), and Sequencing Auxiliary Vials (EXP-AUX002). To reach a coverage sufficient for a phased analysis of DNA methylation, ONT sequencing data for the same samples were pooled across different flow cells, where relevant (Supplementary Table 1). Samples within each sequencing run were randomized while ensuring an even distribution of each group. Additionally, as liver ONT sequencing was performed in two distinct batches with differing read lengths and ligation sequencing kit chemistries, a check for potential batch-specific DMRs was performed, as discussed in the DNA methylation analysis section of the Methods, below.

For the muscle samples, library preparation and sequencing were performed following the same protocol as batch 2 of the liver samples with the following changes: libraries were prepared using the SQK-LSK114 Ligation Sequencing Kit (ONT) following the manufacturer's instructions with the same long read recovery adjustments as used in batch 2 of the liver samples, ONT sequencing was performed using R10.4.1 flow cells, and flow cell priming and reloading were performed using the following kits: Flow Cell Priming Kit V14 (EXP-FLP004) and Sequencing Auxiliary Vials V14 (EXP-AUX003).

For the targeted ONT sequencing of the F2 samples, two BED files were created containing the chromosome, start, and end coordinates of each of the selected DMRs to be targeted, one in the CC019 reference coordinate system and the other in the CC037 reference coordinate system. Included in the target panel were three dominant trans-acting meQTL/ transvection/paramutation DMRs, 20 cis-acting meQTL DMRs, 5 non-dominant trans-acting meQTL DMRs, 20 sex-specific DMRs, 5 imprinted DMRs, 10 skewed XCI DMRs, and 21 regions which were included based on a preliminary analysis but are no longer of interest and have thus been removed from subsequent analysis. An additional 500kb of padding was added to the start and end coordinates of each of the dominant trans-acting meQTL/transvection/paramutation regions to be targeted and 100kb of padding was added to the start and end coordinates of the remaining target regions. The final coordinates used for adaptive sampling are provided in Supplementary Table 10. Strain-specific BED files containing these coordinates were then used to subset the CC019 and CC037 reference genome FASTA files to just the sequence contained within the padded DMRs using the bedtools [3] (v2.30.0) getfasta function. These subset FASTA files were then combined to make a single multi-FASTA with the sequence of each of the padded target regions from both the CC019 and CC037 reference genome in order to minimize sequencing bias which may have been introduced into adaptive sampling by the use of just one reference genome.

Library preparation and sequencing of the F2 samples were performed as described in batch 2 of the liver sequencing above with the following exception: adaptive sampling was activated for sequencing and set to enrich for the multi-FASTA file containing the desired target regions.

The median coverage across both alleles for the whole-genome ONT sequencing of the inbred and F1 generations was 24x (ranging from 8x to 41x), while the median coverage across both alleles over the target regions in the F2 generation was 68x (ranging from 56x to 94x).

RNA extraction

RNA was extracted from ∼10-20mg of flash-frozen median liver lobe using the RNeasy Mini kit (Qiagen Cat. 74104) following the manufacturer's instructions with on-column DNase digestion. Disruption and homogenization were performed using the TissueRuptor II (Qiagen Cat. 9002755) at maximum speed for 10s.

RNA library preparation and sequencing

Strand-specific mRNA libraries were generated using the NEBNext Ultra II Directional RNA Library Prep Kit for Illumina (New England BioLabs #E7760), and mRNA isolation was performed using the Poly(A) mRNA Magnetic Isolation Module (New England BioLabs #E7490). Library preparation followed the manufacturer's protocol (Version 2.2, 05/19). A $1\mu g$ input was used, and samples were fragmented for 15 minutes to achieve an RNA insert size of approximately 200 bp. The subsequent PCR cycling conditions were as follows: 98°C for 30 seconds followed by 8 cycles of 98°C for 10 seconds and 65°C for 75 seconds, with a final step at 65°C for 5 minutes. The stranded mRNA libraries were sequenced on an Illumina NovaSeq 6000 using v1.5 chemistry with an S4 flow cell, generating 150 bp paired-end dual-indexed reads. The mRNA sequencing depth was 50 million reads per sample. RNA was sequenced in two batches, with all inbred samples included in one and all F1 samples included in the other. Six inbred samples were included in both batches to allow for an analysis of batch effects. Principal component analysis (PCA) plots of these six samples across suggested little-to-no effect of batch on expression (see expression analysis and Supplementary Fig. 30). A single CC019xCC037 sample was sequenced to a read depth of 363.6 million reads (50M targeted) and was excluded from subsequent expression analysis.

Statistics & Reproducibility

No statistical method was used to predetermine sample size. One expression sample was excluded due to low coverage; the rest of the samples passed quality control. The experiments were not randomized. The Investigators were not blinded to allocation during experiments and outcome assessment.

Computational Procedures

Collaborative Cross Graphical Genomes

Genome sequence and anchor information for CC019 and CC037 were extracted from the Collaborative Cross Graphical Genome [4] (CCGG; v2.0) utilizing its API. The genome sequence was written into a separate FASTA file for each strain and the anchor information for each strain was written into a separate CSV with the following columns: source anchor, chromosome, outgoing edge, founder on that edge, source anchor coordinate - GRCm38, source anchor coordinate - GRCm39, and source anchor coordinate – respective strain (i.e. CC019 for the CC019 anchors or CC037 for the CC037 anchors).

Overall summary of methylation processing and analysis

To process and analyze our sequencing data, we developed a computational pipeline optimized for the phasing and characterization of allele-specific methylation in genetically divergent mouse strains (Extended Data Fig. 1). Inter-strain analyses of sequencing data can be severely hampered by strain and reference biases22. As such, we designed our computational pipeline to minimize these biases while simultaneously maximizing the amount of data available for analysis. To do so, our pipeline performs alignment and phasing relative to strain-specific genomes and extracts only consensus phasing decisions which are consistent irrespective of the chosen reference genome. Methylation data is then mapped into a pseudo-hybrid intermediate genome, which incorporates genetic variation from both the CC019 and CC037 genomes, for subsequent analysis. The use of the intermediate genome avoids the introduction of bias from the choice of a single reference genome

while maintaining strain-specific methylation information, which has been shown to increase statistical power in inter-strain comparisons of DNA methylation from isogenic mouse models22.

Briefly, ONT sequencing data for all samples was aligned and phased to both the CC019 and CC037 genomes and only reads with consensus phasing decisions, in which the allelic assignment was consistent independent of the reference genome, were utilized. Allele-specific methylation information was subsequently extracted from these reads and converted into a common coordinate system for analysis. DMR finding was then run using data from the inbred and F1 generations to identify regions of the genome in which DNA methylation exhibits at least one of the 12 intergenerational epigenetic inheritance patterns analyzed here. DMR finding was performed separately for liver and muscle, and separately within each tissue for the autosomes, X chromosomes of female samples, and maternal X chromosomal alleles for male samples. After identifying this set of genome-wide significant DMRs, the DMRs were subsequently filtered for quality control and categorized to best match them to an inheritance pattern utilizing methylation differences between the relevant groups. A subset of these DMRs were targeted for sequencing within the F2 generation to further validate/determine the effects of cis and trans-acting genetic variants on these methylation patterns.

ONT sequencing processing

As ONT sequencing for the liver and muscle samples were performed using different versions of flow cells and library preparation kits, they are processed using different pipelines. Liver samples, sequenced using R9.4.1 flow cells and LSK109/LSK110 Ligation Sequencing Kits, were basecalled using Guppy and methylation was called using Nanopolish. Canonical and modified basecalling for muscle samples, sequenced using R10.4.1 flow cells, were performed using Dorado and methylation information was subsequently extracted using modkit. Of note, Nanopolish accounts for supplementary alignments when performing methylation calling whereas modkit excludes these alignments from the methylation extraction. The use of multiple techniques for basecalling and methylation calling across these tissues allows for a limited degree of validation that the identified patterns do not arise due to technical artifacts introduced by these processes. Furthermore, methylation calling (liver) and extraction (muscle) is performed utilizing the reference genome of the strain/allele being considered.

Liver samples were processed using the following pipeline. Basecalling was performed using Guppy (v6.1.2) with the following parameters: `--num_callers 8 --gpu_runners_per_device 8 --chunks_per_runner 1024 --chunk_size 1000 --config dna_r9.4.1_450bps_sup_prom.cfg`, implementing the default Guppy minimum q-score of 10 for super high accuracy basecalling models. The SeqKit [5] (v2.2.0) split2 command with the parameter -p 10 was used to split the FASTQ files passing the minimum q-score cutoff into 10 parts for parallelization of subsequent alignment, phasing, and methylation calling. Alignment was performed using MiniMap2 [6] (v2.24) with the following parameters: `-a -x map-ont`. Alignment of each FASTQ file was performed twice, once relative to the CC019 genome and once relative to the CC037 genome. Heterozygous genetic variants were identified using the nucmer command from MUMmer4 [7] (v4.0.0rc1), converted to a delta file with only SNPs using the show-snps function with the following parameters: `-Clr -I -T`, and subsequently converted to a VCF file using the `my-mummer-2-vcf.py` script with the following parameters: `-n --output-header`. Heterozygous variants were identified once with CC019 as the reference genome and once with CC037 as the reference genome. Phasing information was then added the VCF file to define all heterozygous variants originating from the same parental genome as in phase with one another in the

heterozygous F1 genome. Haplotype phasing of aligned reads was performed using the WhatsHap [8] (v1.6) haplotag function with the following parameter: `--ignore-read-groups`. Haplotype phasing was performed twice for each BAM file, once using each of the VCF files produced using MUMmer4. Consensus haplotype phasing information was extracted by identifying reads for which haplotype assignment was the same using both of the VCF files. Aligned BAM files were split into CC019-phased and CC037-phased files based on the consensus haplotype phasing information, with CC019-phased BAM file information originating from the alignment using CC019 as the reference and with the CC037-phased BAM file information originating from the alignment using CC037 as the reference. Prior to methylation calling, the FASTQ files were indexed using the Nanopolish [9] (v0.14.0) index function using the raw FAST5 files and the sequencing summary file produced by Guppy basecalling. Methylation was called with the Nanopolish call-methylation function once for each set of phased reads using the indexed FASTQ files, phased BAM files, and strain-specific genomes as inputs. Methylation calls were converted to methylation frequency values for each CpG using the Nanopolish `calculate_methylation_frequency.py` script with the following parameters: `-c 2.5 -s`. As Nanopolish groups CpGs within 10bp of each other together during methylation calling, this methylation conversion splits CpGs which are grouped together. Samtools [10] (v1.7) was used for FASTQ and BAM indexing and sorting.

Muscle samples were processed as described in the above pipeline, with the following exceptions: (1) basecalling and methylation calling were performed using Dorado (v0.8.3) using the model `dna_r10.4.1_e8.2_400bps_sup@v5.0.0_5mCG_5hmCG@v2.0.1` with the following parameters: `--modified-bases 5mCG_5hmCG --min-qscore 10`, (2) the resulting unaligned, modified BAM file was then converted to FASTQ format using the samtools (v1.19.2) fastq command with the following parameters to preserve the methylation calls: `-T MM,ML`, (3) alignment was performed using MiniMap2 (v2.28) with the following parameters: `-a -y -x map-ont`, (4) reads with a MAPQ quality score of less than 20 were filtered out after alignment using the samtools view command with the following parameters: `-bSq 20` (note that a MAPQ cutoff of 20 is also used by Nanopolish), (5) haplotype phasing was performed using the WhatsHap (v2.3) haplotag function with the same VCF files produced above using MUMmer4 and the following parameters: `--ignore-read-groups --skip-missing-contigs`, and (6) methylation data was extracted from the final BAM files using the modkit (v0.4.1) pileup command with the following parameters: `--cpg --combine-strands --mod-thresholds m:0.8 --filter-threshold C:0.8` and given the appropriate reference genome. Samtools was also used for FASTQ and BAM indexing, sorting, and merging.

N50 calculations

N50s for each sample were calculated using the Proch-n50 [11] (v1.4.2) tool.

Pairwise alignment of respective CCGG edges

Using the CCGG FASTA and anchor information files, pairwise alignment was performed between all respective edges (those edges bounded by the same anchors) of CC019 and CC037 less than 3kb in length using the pairwise2 function of the Bio python library [12] (v1.78) to create edge-specific CIGAR strings to be used in coordinate mapping. This pairwise alignment was also performed between the respective edges of the mm10 reference genome and both CC019 and CC037 to be used for mapping mm10 coordinates into the CC019 and CC037 coordinate systems and vice-versa.

Coordinate conversion

The use of multiple strain-specific reference genomes necessitates the ability to map particular genome feature coordinates between the genomes. Furthermore, as most reference annotations are exclusively found in the standard mouse reference genome coordinates (e.g. mm10), the ability to map coordinates from mm10 into our strain-specific reference genomes is also crucial. Lastly, to further minimize the introduction of reference bias into our data, we performed all methylation analyses in a pseudo-hybrid intermediate genome created from the CC019 and CC037 reference genomes, rather than picking one of these two genomes in which to perform the analysis. This intermediate genome is created by constructing the maximal length sequences from CC019 and CC037 alignment between each pair of anchors. As such, this intermediate genome has all CC019-specific and CC037-specific insertions represented and any coordinate which exists in either strain-specific genome necessarily exists in the intermediate genome. As such, there is also a necessity to be able to convert all coordinates to and from this intermediate genome.

To do so, we created tools which are capable of mapping coordinates between the CCGG genomes and the intermediate genome, as well as between mm10 and the CCGG genomes. Briefly, these mapping tools utilize the edge-specific CIGAR strings created by the aforementioned pairwise alignment of respective edges of mm10, CC019, and CC037. As the start and end coordinates of each anchor are known for mm10, CC019, CC037, and the intermediate genome, coordinate mapping between any of these coordinate systems can be performed by iterating through each of these CIGAR strings and adding to the anchor position of the respective coordinate system for every insertion and deletion present in the CIGAR string. By doing so, a mapping table can be created with each position in the starting genome and its corollary in the genome to which coordinates are being mapped.

When mapping reference information (e.g. gene, promoter, and enhancer coordinates, etc.) from mm10 into the intermediate coordinate system, the coordinates are first mapped into the CC019 and/or the CC037 coordinate system prior to being mapped into the intermediate coordinate system. When mapping between mm10 and CC019/CC037, there may be coordinates which are present in one genome but not in the others. For this reference information, the nearest existing coordinate in the genome being mapped to is used as the corollary site. This method is also used when mapping the DMR coordinates into mm10 for the creation of Supplementary Tables 2-3 and 6-9. This method is not used for mapping of CpG coordinates prior to methylation analyses.

All coordinates provided in the Supplementary Tables follow BED format, i.e. all ranges are zero-based and half open. For the presentation of coordinates in the Main and Supplementary Figures, all ranges are one-based and closed, following the convention of GenomicRanges as well as genome viewers such as IGV. The mapping of coordinates between coordinate systems and all analyses in R are performed using coordinates which are one-based and closed.

Identification of identical by descent (IBD) regions

IBD regions between CC019 and CC037 were defined as those which cannot be phased using 25kb reads. Briefly, we utilized the heterozygous SNPs between CC019 and CC037 from MUMmer4 and extracted regions in which a 25kb read could not overlap two such heterozygous variants. To do so, we iterate through each SNP in the MUMmer4 VCF files and analyze its position as well as the positions of the three following SNPs relative to each other. For each set of four contiguous SNPs, if the distances between SNP 1 and SNP 3, SNP 2 and SNP 4, and SNP 2 and SNP 3 are all $\geq$ 25kb and the distance between SNP 1 and SNP 4 is $\geq$ 50kb, then the region between SNP 2 and

SNP 3 is defined as IBD. For the first four SNPs of each chromosome, if the position of SNP 2 is $\geq$ 25kb away from SNP 1 and the position of SNP 2 is $\leq$ 25kb away from SNP 3, the region between SNP 1 and SNP 2 is defined as IBD, whereas if the distances between SNP 1 and SNP 3, SNP 2 and SNP 4, and SNP 2 and SNP 3 are all $\geq$ 25kb and the distance between SNP 1 and SNP 4 is $\geq$ 50kb, then the region between SNP 1 and SNP 3 is defined as IBD. Finally, for the final 4 SNPs of each chromosome, if the position of SNP 3 is $\geq$ 25kb away from SNP 4 and the position of SNP 2 is *leq* 25kb away from SNP 3, the region between SNP 3 and SNP 4 is defined as IBD, whereas if the distances between SNP 1 and SNP 3, SNP 2 and SNP 4, and SNP 2 and SNP 3 are all $\geq$ 25kb and the distance between SNP 1 and SNP 4 is $\geq$ 50kb, then the region between SNP 2 and SNP 4 is defined as IBD. This analysis was performed excluding the Y chromosome.

These calculations were performed for both CC019 SNPs relative to the CC037 genome and CC037 SNPs relative to the CC019 genome. IBD coordinates were then converted from the CC reference genome coordinate systems into the intermediate coordinate system and merged to create a unified list of IBD regions using the GenomicRanges [13] (v1.54.1) reduce function. These IBD coordinates were then overlapped with the mapped coordinates of common CpGs present in both the CC019 reference genome and the CC037 reference genome to estimate the percentage of the methylome which is IBD.

Within the CC019 and CC037 genomes, there are 19,258,931 and 19,417,691 CpGs, respectively. Of these, 17,911,438 and 17,969,433 can be mapped into the intermediate genome and 4,773,219 and 4,778,508 overlap IBD regions. Additionally, of the 16,966,618 common CpGs which can be mapped into the intermediate genome, 4,718,665 (27.8%) overlap IBD regions, leaving 11,873,848 autosomal and 374,105 X chromosomal common, non-IBD CpGs which can be mapped into the intermediate genome.

DNA methylation analysis

For the inbred and F1 liver dataset, a single female CC019xCC037 F1 sample was removed from subsequent methylation and expression analyses as phased ONT reads of the X chromosome were assigned to only one allele and the coverage ratio of the X chromosome to the autosomes was roughly half that of other female samples, indicating that this sample was X0 and thus only contains 1 copy of the X chromosome, a phenomenon which has been reported in inbred mice [14].

Methylation data was processed in R (v4.3.1) using the bsseq [15] package (v1.38.0). For liver, coordinate-converted methylation frequency files for each sample were imported into R and converted into BS objects, specifying the chromosome, genomic position mapped into the pseudohybrid intermediate genome, Nanopolish `called_sites_methylated`, and Nanopolish `called_sites` as chr, pos, M, and C, respectively. For muscle, the read.modkit function from the bsseq package (v1.42.0) was utilized to import methylation data from the modkit output files into R (v4.4.0). The resulting BS objects with combined 5mC and 5hmC calls were utilized for subsequent analyses in R (v4.3.1) using the bsseq package (v1.38.0).

Methylation values were smoothed using the BSmooth function from bsseq. This function smooths the methylation data of each sample individually to decrease methylation variance and facilitate the identification of differentially methylated regions, which are more commonly associated with functionally relevant methylation changes than single-CpG differences [15]. Furthermore, methylation smoothing allows for a better tradeoff between coverage and sample size [16]. BSmooth was run using all CpGs present in each sample, including strain-specific CpGs, with the default parameters except the following: maxGap = 100000.

Smoothed methylation data was subsequently subset into three groups: autosomal, X chromosome female samples, and X chromosome maternal allele of male samples, as proper analysis of the X chromosome necessitates a more in-depth consideration of sex imbalance due to X chromosome inactivation and because male samples only have maternal X chromosomes. Within each of these groups, the BS objects are subset to just those CpGs which are covered in at least 75% of the CC019 inbred samples and CC019 phased F1 alleles and 75% of the CC037 inbred samples and CC037 phased F1 alleles. This effectively removes strain-specific CpGs from the analysis, although the methylation values of strain-specific CpGs have been used to impute methylation values over strain-agnostic CpGs during smoothing. CpGs for which the smoothed methylation estimate for at least one sample was 'NA' were also filtered.

The following number of CpGs were analyzed for each of the conditions – liver autosomal: 11,501,000; muscle autosomal: 11,903,947; liver chrX female: 341,941; muscle chrX female: 352457; liver chrX male maternal allele: 324,311; muscle chrX male maternal allele: 346,487. Of these, the following number of CpGs overlapped non-IBD regions of the genome (1) liver autosomal: 10,747,060 (90.5%) of autosomal common non-IBD CpGs; (2) muscle autosomal: 10,784,692 (90.8%) of common non-IBD autosomal CpGs; (3) liver chrX female: 324,579 (86.8%) of common non-IBD chrX CpGs; (4) muscle chrX female: 329,943 (88.2%) of common non-IBD chrX CpGs; (5) liver chrX male maternal allele: 311,733 (83.3%) of common non-IBD chrX CpGs; and (6) muscle chrX male maternal allele: 325,500 (87%) of common non-IBD chrX CpGs.

DMR finding was performed utilizing an F-statistic test, which allows for a genome-wide search for multiple distinct DNA methylation patterns using a single test. This identifies regions of the genome where one or more of these contrasts is different from zero while controlling the family-wise error rate (FWER). Following identification of DMRs, we do post-hoc categorization, described below. We considered the following contrasts:

1. β_{sex} = [inbred females + F1 females either allele] - [inbred males + F1 males either allele]
   (This contrast is only included for the autosomal comparison.)
2. $\beta_{\text{OverUnder\_Dom}}$ = [inbreds] - [F1s either allele]
3. $\beta_{\text{imprinting}}$ = [CC019xCC037 F1 CC019 alleles + CC037xCC019 F1 CC037 alleles] - [CC019xCC037 F1 CC037 alleles + CC037xCC019 F1 CC019 alleles]
   (This contrast is only included for the autosomal and X chromosome female sample comparisons.)
4. β_{cis} = [inbred CC019s + F1 CC019 alleles] - [inbred CC037s + F1 CC037 alleles]
5. β_{trans} = [inbred CC019s] - [inbred CC037s]
6. $\beta_{\text{biallelic}}$ = [F1 CC019 alleles] - [F1 CC037 alleles]
7. $\beta_{\text{AS\_emerg\_19}}$ = [F1 CC019 alleles] - [inbreds + F1 CC037 alleles]
8. $\beta_{\text{AS\_emerg\_37}}$ = [F1 CC037 alleles] - [inbreds + F1 CC019 alleles]
9. $\beta_{\text{paramut\_19}}$ = [Inbred CC019s] - [inbreds CC037s + F1s either allele]
10. $\beta_{\text{paramut\_37}}$ = [Inbred CC037s] - [inbreds CC019s + F1s either allele]
11. $\beta_{\text{polar\_19x37}}$ = [CC019xCC037 F1s] - [inbreds + CC037xCC019 F1s]
    (This contrast is only included for the autosomal and X chromosome female sample comparisons.)

12. $\beta_{\text{polar\_37x19}}$ = [CC037xCC019 F1s] - [inbreds + CC019xCC037 F1s]
(This contrast is only included for the autosomal and X chromosome female sample comparisons.)

13. β_{bipolar} = [CC019xCC037 F1s] - [CC037xCC019 F1s]
(This contrast is only included for the autosomal and X chromosome female sample comparisons.)

We performed a genome-wide search using a single F-statistic on the autosomes, which simultaneously tests for these 13 contrasts. We supplemented this search with an additional search across the X chromosome for female samples (excluding contrast 1) and an additional search across the X chromosome for the maternal allele of male samples (excluding contrasts 1, 3, 11-13). The search was based on the `fstat.pipeline` function from bsseq, which runs the `BSmooth.fstat`, `smoothSds`, `computeStat`, and `dmrFinder` functions sequentially prior to running permutation testing using the `permuteAll`, `getNullDistribution_BSmooth.fstat`, and `getFWER_fstat` functions. Briefly, this pipeline identifies DMRs passing the F-statistic cutoff and then performs permutation testing to adjust for multiple testing and control the FWER. The fstat.pipeline function was modified to accept a k value as input to be used in the `smoothSds` and `getNullDistribution_BSmooth.f` functions. Furthermore, the pipeline has been updated to filter DMRs as well as null DMRs from permutation testing based on the following three criteria:

1. Outlier samples are driven by low coverage
   - All hypermethylated samples with Cook's distance>4*mean(Cook's distances) or all hypomethylated samples with Cook's distance>4*mean(Cook's distances) have an average coverage over the DMR of < 3x.
2. Groups with very low or very high coverage
   - Average coverages of any of the following four groups are <3x or >150x: inbred CC019s, inbred CC037s, CC019 alleles of F1s, and CC037 alleles of F1s.
3. Disproportionate coverages
   - $|\log_2(\text{Coverage}_{\text{inbred 19}}/\text{Coverage}_{\text{inbred 37}})| > 2$
   - $|\log_2(\text{Coverage}_{\text{F1 19}}/\text{Coverage}_{\text{F1 37}})| > 2$
   - $|\log_2(\text{Coverage}_{\text{inbred 19}}/\text{Coverage}_{\text{F1 19}})| > 3$ or
     $|\log 2(\text{Coverage}_{\text{inbred 37}}/\text{Coverage}_{\text{F1 37}})| > 3$
     (Note this higher cutoff adjusts for the expected coverage differences between inbreds and phased F1s.)

The pipeline was run using the following parameters: `nperm` = 1000, `cutoff` = 4.6^2, `k` = 21, `maxGap.sd` = 10^8, `maxGap.dmr` = 1000. The cutoff of 4.6^2 is the recommended F-statistic bsseq cutoff based upon the default T-statistic cutoff of 4.6 [17]. We ran 1000 permutations and computed, for each candidate DMRs, in how many permutations we observe a null DMR anywhere in the genome, which were better than the candidate. As a measure of better DMR we used the areaStat statistic, which is the sum of the CpG-level F-statistics. We expect this procedure to control the FWER. As the cutoff for significance, we selected candidate DMRs which had no better null DMRs in less than or equal to 100 permutations. This translates to an FWER of 101/1001 = 0.101. Following this, we discarded significant DMRs with less than 3 CpGs.

The three lists of output DMRs and smoothed BS objects were then used to calculate smoothed methylation differences between the groups defined in the contrast matrices used for testing as well as eight additional contrasts, all defined below:

1. Δ_{sex} = [inbred females + F1 females either allele] - [inbred males + F1 males either allele] (This difference is only calculated for the autosomal comparison.)
2. $\Delta_{OverUnder\_Dom}$ = [inbreds] - [F1s either allele]
3. $\Delta_{imprinting}$ = [CC019xCC037 F1 CC019 alleles + CC037xCC019 F1 CC037 alleles] - [CC019xCC037 F1 CC037 alleles + CC037xCC019 F1 CC019 alleles]
(This difference is only calculated for the autosomal and X chromosome female sample comparisons.)
4. Δ_{cis} = [inbred CC019s + F1 CC019 alleles] - [inbred CC037s + F1 CC037 alleles]
5. Δ_{trans} = [inbred CC019s] - [inbred CC037s]
6. $\Delta_{biallelic}$ = [F1 CC019 alleles] - [F1 CC037 alleles]
7. $\Delta_{AS\_emerg\_19)}$ = [F1 CC019 alleles] - [inbreds + F1 CC037 alleles]
8. $\Delta_{AS\_emerg\_37}$ = [F1 CC037 alleles] - [inbreds + F1 CC019 alleles]
9. $\Delta_{paramut\_19}$ = [Inbred CC019s] - [inbreds CC037s + F1s either allele]
10. $\Delta_{paramut\_37}$ = [Inbred CC037s] - [inbreds CC019s + F1s either allele]
11. Δ_{polar_19x37} = [CC019xCC037 F1s] - [inbreds + CC037xCC019 F1s]
(This contrast is only included for the autosomal and X chromosome female sample comparisons.)
12. Δ_{polar_37x19} = [CC037xCC019 F1s] - [inbreds + CC019xCC037 F1s]
(This contrast is only included for the autosomal and X chromosome female sample comparisons.)
13. $\Delta_{bipolar}$ = [CC019xCC037 F1s] - [CC037xCC019 F1s]
(This contrast is only included for the autosomal and X chromosome female sample comparisons.)
14. $\Delta_{Inbr\_F1\_19}$ = [inbred CC019s] - [F1 CC019 alleles]
15. $\Delta_{Inbr\_F1\_37}$ = [inbred CC037s] - [F1 CC037 alleles]
16. $\Delta_{Inbr\_19\_F1\_37}$ = [inbred CC019s] - [F1 CC037 alleles]
17. $\Delta_{Inbr\_37\_F1\_19}$ = [inbred CC037s] - [F1 CC019 alleles]
18. $\Delta_{Inbr\_F1\text{-}19x37\_19}$ = [inbred CC019s] - [CC019x CC037 F1 CC019 alleles]
19. $\Delta_{Inbr\_F1\text{-}19x37\_37}$ = [inbred CC037s] - [CC019x CC037 F1 CC037 alleles]
20. $\Delta_{Inbr\_F1\text{-}37x19\_19}$ = [inbred CC019s] - [CC037x CC019 F1 CC019 alleles]
21. $\Delta_{Inbr\_F1\text{-}37x19\_37}$ = [inbred CC037s] - [CC037x CC019 F1 CC037 alleles]

These groupwise methylation differences were then used to assign each statistically significant DMR to its proper category. The defined categories are mutually exclusive and, as such, each DMR

is assigned to only one. However, as these methylation patterns do not necessarily function independently of one another, additional categories have been defined which exhibit characteristics of multiple inheritance patterns, as well as a one for those DMRs which cannot be clearly categorized. DMRs assigned to these additional categories are not considered in subsequent analyses. For the liver data, we have 358 autosomal DMRs in categories 14-19 and 8 DMRs in category 20. For the muscle data, we have 20 autosomal DMRs in categories 14-19 and 0 DMRs in category 20. These numbers are after application of the smoothing filter described below. The maximum absolute categorical difference is defined as Δ_{max}.

1. Sex-specific DMR:
$$\Delta_{\text{max}} = |\Delta_{\text{sex}}| \ \& \ |\Delta_{\text{sex}}| \geq 10\%$$
(This condition is only checked for the autosomal comparison.)

2. Imprinted DMR:
$$\Delta_{\text{max}} = |\Delta_{\text{imprinting}}| \ \& \ |\Delta_{\text{imprinting}}| \geq 10\%$$
(This condition is only checked for the autosomal and X chromosome female sample comparisons.)

3. Overdominant and underdominant emergent:
$$\Delta_{\text{max}} = |\Delta_{\text{OverUnder\_Dom}}| \ \& \ |\Delta_{\text{OverUnder\_Dom}}| \geq 10\%$$
OR
$$\begin{aligned} |\Delta_{\text{OverUnder\_Dom}}| &\geq 10\% \ \& \\ |\Delta_{\text{Inbr\_F1\_19}}| \geq 10\% \ \& \ |\Delta_{\text{Inbr\_F1\_37}}| &\geq 10\% \ \& \\ |\Delta_{\text{Inbr\_37\_F1\_19}}| \geq 10\% \ \& \ |\Delta_{\text{Inbr\_19\_F1\_37}}| &\geq 10\% \ \& \\ \text{sign}(\Delta_{\text{Inbr\_F1\_19}}) = \text{sign}(\Delta_{\text{Inbr\_F1\_37}}) \ &\& \\ \text{sign}(\Delta_{\text{Inbr\_19\_F1\_37}}) = \text{sign}(\Delta_{\text{Inbr\_37\_F1\_19}}&) \end{aligned}$$

4. Polar emergent – CC019xCC037:
$$\begin{aligned} |\Delta_{\text{polar\_19x37}}| \geq 10\% \ \& \ |\Delta_{\text{polar\_37x19}}| &< 10\% \ \& \\ |\Delta_{\text{bipolar}}| \geq 10\% \ \& \ |\Delta_{\text{trans}}| &< 10\% \ \& \\ \text{sign}(\Delta_{\text{Inbr\_F1-19x37\_19}}) = \text{sign}(\Delta_{\text{Inbr\_F1-19x37\_37}}&) \end{aligned}$$
(This condition is only checked for the autosomal and X chromosome female sample comparisons.)

5. Polar emergent – CC037xCC019:
$$\begin{aligned} |\Delta_{\text{polar\_37x19}}| \geq 10\% \ \& \ |\Delta_{\text{polar\_19x37}}| &< 10\% \ \& \\ |\Delta_{\text{bipolar}}| \geq 10\% \ \& \ |\Delta_{\text{trans}}| &< 10\% \ \& \\ \text{sign}(\Delta_{\text{Inbr\_F1-37x19\_19}}) = \text{sign}(\Delta_{\text{Inbr\_F1-37x19\_37}}&) \end{aligned}$$
(This condition is only checked for the autosomal and X chromosome female sample comparisons.)

6. Bipolar emergent:

$$|\Delta_{\text{bipolar}}| \geq 10\% \; \& \; |\Delta_{\text{bipolar}}| \geq |\Delta_{\text{polar\_19x37}}| \; \&$$
$$|\Delta_{\text{bipolar}}| \geq |\Delta_{\text{polar\_37x19}}| \; \& \; |\Delta_{\text{trans}}| < 10\% \; \&$$
$$\text{sign}(\Delta_{\text{Inbr\_F1-19x37\_19}}) = \text{sign}(\Delta_{\text{Inbr\_F1-19x37\_37}}) \; \&$$
$$\text{sign}(\Delta_{\text{Inbr\_F1-37x19\_19}}) = \text{sign}(\Delta_{\text{Inbr\_F1-37x19\_37}})$$

(This condition is only checked for the autosomal and X chromosome female sample comparisons.)

7. Biallelic emergent:

$$|\Delta_{\text{biallelic}}| \geq 10\% \; \& \; |\Delta_{\text{trans}}| < 0.5 * |\Delta_{\text{biallelic}}| \; \&$$
$$|\Delta_{\text{Inbr\_F1\_19}}| \geq 5\% \; \& \; |\Delta_{\text{Inbr\_F1\_37}}| \geq 5\% \; \&$$
$$|\Delta_{\text{Inbr\_19\_F1\_37}}| \geq 5\% \; \& \; |\Delta_{\text{Inbr\_37\_F1\_19}}| \geq 5\% \; \&$$
$$\text{sign}(\Delta_{\text{Inbr\_F1\_19}}) \neq \text{sign}(\Delta_{\text{Inbr\_F1\_37}})$$

8. Allele-specific emergent – CC019:

$$|\Delta_{\text{AS\_emerg\_19}}| \geq 10\% \; \& \; |\Delta_{\text{biallelic}}| \geq 10\% \; \&$$
$$|\Delta_{\text{Inbr\_F1\_19}}| \geq 10\% \; \& \; |\Delta_{\text{Inbr\_37\_F1\_19}}| \geq 10\% \; \&$$
$$|\Delta_{\text{trans}}| < 10\% \; \& \; |\Delta_{\text{Inbr\_F1\_37}}| < 10\% \; \& \; |\Delta_{\text{Inbr\_19\_F1\_37}}| < 10\%$$

9. Allele-specific emergent – CC037:

$$|\Delta_{\text{AS\_emerg\_37}}| \geq 10\% \; \& \; |\Delta_{\text{biallelic}}| \geq 10\% \; \&$$
$$|\Delta_{\text{Inbr\_F1\_37}}| \geq 10\% \; \& \; |\Delta_{\text{Inbr\_19\_F1\_37}}| \geq 10\% \; \&$$
$$|\Delta_{\text{trans}}| < 10\% \; \& \; |\Delta_{\text{Inbr\_F1\_19}}| < 10\% \; \& \; |\Delta_{\text{Inbr\_37\_F1\_19}}| < 10\%$$

10. Dominant trans-acting meQTL | transvection | paramutation – CC019:

$$|\Delta_{\text{paramut\_19}}| \geq 10\% \; \& \; |\Delta_{\text{trans}}| \geq 10\% \; \&$$
$$|\Delta_{\text{Inbr\_F1\_19}}| \geq 10\% \; \& \; |\Delta_{\text{Inbr\_19\_F1\_37}}| \geq 10\% \; \&$$
$$|\Delta_{\text{biallelic}}| < 10\% \; \& \; |\Delta_{\text{Inbr\_F1\_37}}| < 10\% \; \& \; |\Delta_{\text{Inbr\_37\_F1\_19}}| < 10\%$$

11. Dominant trans-acting meQTL | transvection | paramutation – CC037:

$$|\Delta_{\text{paramut\_37}}| \geq 10\% \; \& \; |\Delta_{\text{trans}}| \geq 10\% \; \&$$
$$|\Delta_{\text{Inbr\_F1\_37}}| \geq 10\% \; \& \; |\Delta_{\text{Inbr\_37\_F1\_19}}| \geq 10\% \; \&$$
$$|\Delta_{\text{biallelic}}| < 10\% \; \& \; |\Delta_{\text{Inbr\_F1\_19}}| < 10\% \; \& \; |\Delta_{\text{Inbr\_19\_F1\_37}}| < 10\%$$

12. Non-dominant trans-acting meQTL:

$$|\Delta_{\text{trans}}| \geq 10\% \; \& \; |\Delta_{\text{biallelic}}| < 0.5 * |\Delta_{\text{trans}}| \; \&$$
$$|\Delta_{\text{Inbr\_F1\_19}}| \geq 5\% \; \& \; |\Delta_{\text{Inbr\_F1\_37}}| \geq 5\% \; \&$$
$$|\Delta_{\text{Inbr\_19\_F1\_37}}| \geq 5\% \; \& \; |\Delta_{\text{Inbr\_37\_F1\_19}}| \geq 5\% \; \&$$
$$\text{sign}(\Delta_{\text{Inbr\_F1\_19}}) \neq \text{sign}(\Delta_{\text{Inbr\_F1\_37}})$$

13. cis-acting meQTL:

$$|\Delta_{\text{cis}}| \geq 10\% \ \& \ |\Delta_{\text{trans}}| \geq 10\% \ \& \ |\Delta_{\text{biallelic}}| \geq 10\% \ \& \\ |\Delta_{\text{Inbr\_19\_F1\_37}}| \geq 10\% \ \& \ |\Delta_{\text{Inbr\_37\_F1\_19}}| \geq 10\% \ \& \\ |\Delta_{\text{Inbr\_F1\_19}}| < 10\% \ \& \ |\Delta_{\text{Inbr\_F1\_37}}| < 10\%$$

14. cis-acting meQTL/biallelic emergent:

$$|\Delta_{\text{biallelic}}| > |\Delta_{\text{trans}}| \ \& \ |\Delta_{\text{Inbr\_F1\_19}}| < 10\% \ \& \ |\Delta_{\text{Inbr\_F1\_37}}| < 10\% \ \& \\ \text{sign}(\Delta_{\text{Inbr\_F1\_19}}) \neq \text{sign}(\Delta_{\text{Inbr\_F1\_37}})$$

15. cis-acting meQTL/allele-specific emergent – CC019

$$|\Delta_{\text{biallelic}}| > |\Delta_{\text{trans}}| \ \& \ |\Delta_{\text{Inbr\_F1\_37}}| < 10\% \ \& \\ \text{sign}(\Delta_{\text{Inbr\_F1\_19}}) = \text{sign}(\Delta_{\text{Inbr\_37\_F1\_19}}) \ \& \\ \text{sign}(\Delta_{\text{Inbr\_37\_F1\_19}}) \neq \text{sign}(\Delta_{\text{Inbr\_19\_F1\_37}})$$

16. cis-acting meQTL/allele-specific emergent – CC037

$$|\Delta_{\text{biallelic}}| > |\Delta_{\text{trans}}| \ \& \ |\Delta_{(\text{Inbr\_F1\_19}}| < 10\% \ \& \\ \text{sign}(\Delta_{\text{Inbr\_F1\_37}}) = \text{sign}(\Delta_{\text{Inbr\_19\_F1\_37}}) \ \& \\ \text{sign}(\Delta_{\text{Inbr\_19\_F1\_37}}) \neq \text{sign}(\Delta_{\text{Inbr\_37\_F1\_19}})$$

17. cis-acting meQTL/non-dominant trans-acting meQTL

$$|\Delta_{\text{trans}}| > |\Delta_{\text{biallelic}}| \ \& \ |\Delta_{\text{Inbr\_F1\_19}}|| < 10\% \ \& \ |\Delta_{\text{Inbr\_F1\_37}}| < 10\% \ \& \\ \text{sign}(\Delta_{\text{Inbr\_F1\_19}}) \neq \text{sign}(\Delta_{(\text{Inbr\_F1\_37}})$$

18. cis-acting meQTL/dominant trans-acting meQTL | transvection | paramutation – CC019

$$|\Delta_{\text{trans}}| > |\Delta_{\text{biallelic}}| \ \& \ |\Delta_{(\text{Inbr\_F1\_37}}| < 10\% \ \& \\ \text{sign}(\Delta_{\text{Inbr\_F1\_19}}) = \text{sign}(\Delta_{\text{Inbr\_19\_F1\_37}}) \ \& \\ \text{sign}(\Delta_{\text{Inbr\_37\_F1\_19}}) \neq \text{sign}(\Delta_{\text{Inbr\_19\_F1\_37}})$$

19. cis-acting meQTL/dominant trans-acting meQTL | transvection | paramutation – CC037

$$|\Delta_{\text{trans}}| > |\Delta_{\text{biallelic}}| \ \& \ |\Delta_{\text{Inbr\_F1\_19}}| < 10\% \ \& \\ \text{sign}(\Delta_{\text{Inbr\_F1\_37}}) = \text{sign}(\Delta_{\text{Inbr\_37\_F1\_19}}) \ \& \\ \text{sign}(\Delta_{\text{Inbr\_19\_F1\_37}}) \neq \text{sign}(\Delta_{\text{Inbr\_37\_F1\_19}})$$

20. Uncategorized

All remaining DMRs

After categorization, a final filter was implemented to ensure that sufficient raw methylation differences are present over identified DMRs. To do so, raw methylation differences between the relevant groups for each category, with strain-specific CpGs included, were calculated over each DMR as well as 1kb and 3kb windows upstream and downstream of the DMRs, as smoothing takes into account regional methylation effects. DMRs in which at least one of these windows exhibits a raw methylation difference of at least 10% or half of the smoothed methylation difference and in the same direction as the smoothed methylation difference are retained. As nearby cis-acting meQTLs will create a methylation difference between the relevant groups of DMRs categorized as allele-specific emergent, dominant trans-acting meQTL/transvection/paramutation, non-dominant trans-acting meQTL, or biallelic emergent, the relevant methylation difference for these DMRs was also checked to be greater than Δ_{cis} within the window.

Whole-genome ONT sequencing of the inbred and F1 liver samples was completed in two batches with differing library preparation kits (batch 1: LSK109, batch 2: LSK110) and target read lengths (batch 1: 10kb, batch 2: 20-30kb), factors which may influence alignment, phasing, and base/methylation calling. Although each batch includes inbred samples from each strain as well as heterozygous F1 crosses, ensuring that none of the autosomal methylation comparisons are fully confounded by batch, we searched our autosomal DMRs to identify any which may still be influenced by a batch effect. To do so, smoothed methylation differences between the relevant groups for each category were calculated over each DMR both with and without the samples from batch 1 included. Those DMRs for which the relevant methylation difference without the inclusion of the batch 1 samples is less than half of the relevant methylation difference with all samples included are noted as potentially influenced by sequencing batch. Of the 6,970 autosomal DMRs identified in the liver, only 29 (0.4%) fall into this category. These DMRs are noted in Supplementary Table 2. This additional check was not performed for the chrX analyses as all male inbred CC019 samples originated from batch 1. As such, male maternal allele comparisons relative to the inbred CC019 samples cannot be tested excluding the batch 1 samples.

For the liver F2 dataset, coordinate-converted methylation frequency files for each sample were imported into R and converted into BS objects. Methylation data was split by autosomal and X chromosomal CpGs. The local haplotype of each sample over each of the 84 targeted DMRs was then assigned by comparing the phased coverage of each allele over the adaptive sampling target regions. Samples/regions for which the coverage of the phased CC019 allele is ¿ 75% of the total coverage for the sample over the region are defined as homozygous CC019. Samples/regions for which the coverage of the phased CC037 allele is ¿ 75% of the total coverage for the sample over the region are defined as homozygous CC037. Samples/regions for which the coverage of the phased CC019 and CC037 alleles are both $\leq$ 75% of the total coverage for the sample over the region are defined as heterozygous. Methylation data was then subset to those CpGs within 500kb of the candidate transvection/dominant trans-acting meQTL/paramutation regions, or 100kb of the remaining targeted regions. Smoothing was performed separately on each of these regions with the same parameters used for the inbred and F1 dataset. After smoothing, only the proper alleles were extracted based on the assigned local haplotype: CC019 allele only for homozygous CC019 samples, CC037 allele only for homozygous CC037 samples, and both alleles for heterozygous samples. These samples/alleles were then plotted over the autosomal DMRs, and just the female samples/alleles were plotted over the X chromosomal DMRs. Notably, only a single female F2 sample had heterozygosity over any of the X chromosomal DMRs. As heterozygosity is necessary to identify skewed XCI, these DMRs were removed from subsequent analysis.

Analysis of methylation over the *Vps37c* and *Capn11* IAPs

Analysis of methylation over each of these CC037-specific IAPs requires additional re-processing, as the *Vps37c* IAP is not present in the CC037 reference genome and coordinates within the *Capn11* IAP, which is present within the CC037 reference genome, cannot be mapped into the intermediate genome as they reside within an edge which is longer than 3kb. Furthermore, IGV investigation of reads overlapping the *Capn11* IAP reveals additional supplementary alignments which are highly likely to belong to other IAP elements in the CC037 genome that are not present within the reference sequence. As such, due to the high degree of sequence similarity between these IAPs, MiniMap2 assigns the sections of these reads which directly overlap the IAPs missing from the reference genome as supplementary alignments and incorrectly places them over an IAP which is present within the reference genome, in this case the *Capn11* IAP. These supplementary alignments do not extend past the region containing the IAP.

Within the Integrative Genomics Viewer [18] (IGV; v2.19.1), the sequence of the 5kb insertion present in the CC037-phased reads of the dominant trans-acting meQTL/transvection/paramutation region overlapping the gene *Vps37c* was copied from read `80ce626d-cf13-4e01-bdb9-a78ce5876b04` of the CC037 inbred muscle sample 37-104 to be used as a representative example. The sequence of this insertion was run through the Dfam44 (v3.9) sequence search tool using Mus musculus as the model organism and the Dfam curated threshold cutoff. This query returned six matches, all of which fall into the IAP category. The longest two matches with the lowest E-values (both 0) are of the family IAPEz-int (overlapping 4,299bp of the 5,054bp insertion).

The CC037 reference genome sequence between the two flanking CpGs which can be mapped into the intermediate genome around the *Capn11* IAP region was also run through Dfam using the parameters defined above. This query returned 12 matches of which 6 fall into the IAP category, overlapping 5,274bp of the 5,841bp sequence. The longest two matches with the lowest E-values (both 0) are of the family IAPEz-int (overlapping 4,302bp of the 5,841bp sequence).

To investigate the methylation pattern over the *Vps37c* IAP, which is not present in the original CC037 reference genome and, as such, did not have methylation called by Nanopolish in the liver samples, we created a new version of the CC037 reference genome which includes the IAP by incorporating the entire 5,054bp sequence at the site of the insertion.

We then locally re-processed methylation over both the *Vps37c* and *Capn11* IAPs for all samples for which at least one CC037 allele is present. Genotyping data was utilized to select those F2s which were heterozygous or homozygous for the CC037 allele in this region based upon matching of the nearest upstream and downstream called variants for which CC019 and CC037 differ to the known genotype of CC019 and CC037.

For the inbred, F1, and F2 liver samples, the *Vps37c* IAP re-processing was performed as follows: (1) the samtools view function was used to extract the IDs for all CC037-phased reads which aligned to the site of the original DMR overlapping *Vps37c* with an additional 100kb added upstream and downstream, (2) the seqtk [19] (v1.5-r133) subseq function was used to extract these reads from the basecalled FASTQ files, (3) minimap2 was used to align these reads to the new CC037 reference genome including the new IAP sequence, (4) the nanopolish index and call-methylation functions were used to call the methylation of these newly aligned reads using the new CC037 reference genome with the IAP included, and (5) the Nanopolish provided `calculate_methylation_frequency.py` script was used to convert the methylation calls into methylation frequencies for each CpG site. For the inbred and F1 muscle samples, the *Vps37c* IAP

re-processing was performed as follows: (1) the samtools view function was used to extract the IDs for all CC037-phased reads which aligned to the site of the original DMR overlapping *Vps37c* with an additional 100kb added upstream and downstream, (2) the seqtk subseq function was used to extract these reads from the basecalled and methylation called FASTQ files, (3) minimap2 was used to align these reads to the new CC037 reference genome including the new IAP sequence, (4) the modkit pileup command was used to extract methylation frequencies for each CpG site. Note that the CC019 samples/alleles were not re-processed as this IAP is not present in the CC019 genome.

With the incorporation of the *Vps37c* IAP into the CC037 reference genome, all coordinates after the start of the IAP can no longer be directly mapped into the same pseudo-hybrid intermediate coordinate system which we used for our analysis. As such, we designed a new, local pseudo-hybrid intermediate coordinate system around this IAP in which to analyze its methylation concordantly with CC019 samples/alleles. To do so, the coordinate at which the IAP was inserted into the CC037 reference genome (i.e. its start coordinate) was mapped into the original pseudo-hybrid intermediate coordinate system to determine where in the new intermediate coordinate system the IAP begins (this is the first coordinate at which the two intermediate coordinate systems diverge). CpG coordinates from CC019 samples/alleles which were previously mapped into the original intermediate coordinate system were then mapped into the new intermediate coordinate system as follows: (1) those coordinates which are upstream of the coordinate in which the IAP was inserted (i.e. their linear coordinate in the original intermediate coordinate system is less than the IAP insertion point coordinate) are kept the same, and (2) those coordinates which are downstream of the coordinate in which the IAP was inserted (i.e. their linear coordinate in the original intermediate coordinate system is greater than the IAP insertion point coordinate) are mapped to the sum of their coordinate in the original intermediate coordinate system and the length of the IAP insertion (5,054bp). Note that this ensures that no coordinates of CC019 samples/alleles are located over the IAP itself, as this IAP is not present in the CC019 reference genome. The re-processed CC037 samples/alleles were then mapped into the new intermediate coordinate system utilizing the previous mapping of each respective sample into the original intermediate coordinate system as follows: (1) those coordinates which are upstream of the coordinate in which the IAP was inserted (i.e. their linear coordinate in the original intermediate coordinate system is less than the IAP insertion point coordinate) are kept the same, (2) those coordinates which are downstream of the end coordinate of the IAP in the new intermediate coordinate system (i.e. the sum of their linear coordinate in the original intermediate coordinate system and the length of the IAP is greater than the end coordinate of the IAP in the new intermediate coordinate system) are mapped to the sum of their coordinate in the original intermediate coordinate system and the length of the IAP insertion (5,054bp), and (3) those coordinates which are within the IAP (i.e. all remaining coordinates) are linearly mapped between the final coordinate from upstream of the IAP and the first coordinate from downstream of the IAP by adding the linear distance between each respective coordinate and its preceding coordinate in the new CC037 reference genome with the IAP included to the preceding coordinate in the new intermediate coordinate system. After mapping the coordinates into the new intermediate coordinate system, methylation was smoothed following the same procedure as previously described and plotted separately for the liver inbred and F1 samples, the liver F2 samples, and the muscle inbred and F1 samples. Note that for the F2 samples, a minimum coverage cutoff of 1x was implemented to remove very low-covered samples as this region was not included in the original capture target regions.

Capn11 IAP re-processing was performed as described above with the following exceptions: (1) CC037-phased reads which align to only the IAP region were excluded from analysis to avoid analysis of incorrectly mapped supplementary alignments, taking only reads which overlap the

100kb regions beginning 1kb upstream and downstream of the IAP, (2) a new genome was not created as the *Capn11* IAP is already present within the CC037 reference genome, (3) an IAP length of 5,841bp was used, and (4) mapped coordinates from the original processing pipeline were used for all CpGs which are downstream of the end coordinate of the IAP. As the *Capn11* IAP region was included in the original capture target regions, a minimum coverage cutoff was not implemented.

Note that for each of the re-processing and re-analysis steps above, the same versions of each tool and the same parameters used in the original processing were maintained. Additionally, the IAP coordinates used in the re-processing pipelines are estimates based upon our available sequencing and reference genome data, with the *Vps37c* IAP being considered as the full length of the 5,054bp insertion and the *Capn11* IAP being considered as the full 5,841bp region between the flanking CpGs which can be mapped into the intermediate genome. Lastly, for each of these analyses, methylation over these estimated IAP regions is only considered for the allele which contains the IAP.

Cross-tissue DMR overlapping

All cross-tissue DMR overlaps reported here show the difference between the total number of DMRs identified in at least one tissue (i.e. the sum of the number of liver and muscle DMRs) and the total number of DMRs remaining after reducing this superset of DMRs using the GenomicRanges reduce function. However, all DMR overlapping percentages (shown within-tissue) represent the percentage of DMRs within that tissue which overlap DMRs assigned the same category within the other tissue. This is done to account for small changes in the DMR coordinates between tissues.

DMR gene tracks

Gene tracks for the DMR plots were made using the dmrseq [20] (v1.22.1) dmrPlotAnnotations function with the mm10 annotation from annotatr [21] (v1.28.0).

DMR-gene associations

To identify genes which may be regulated by these DMRs, three levels of DMR-gene associations were defined: (1) promoter overlap, (2) enhancer overlap, and (3) genomic proximity (linear distance between the corresponding gene and DMR). Gene coordinates were taken from strain-specific genome annotation files and mapped into the intermediate coordinate system. Promoters were defined as the regions 3kb upstream and downstream of transcriptional start sites (TSSs), downloaded from version 102 of the `mmusculus_gene_ensembl` mart via BiomaRt [22] (v2.58.0). Enhancer-gene interaction annotations were downloaded from EnhancerAtlas 2.0 [23]. Mus Musculus Liver enhancer annotations were used for DMR-gene associations with liver DMRs and Mus Musculus Limb E14.5 enhancer annotations were used for the muscle DMRs. Enhancers were mapped from mm9 to mm10 using the UCSC LiftOver tool. All TSSs and enhancers were then mapped from mm10 into the intermediate genome coordinate system.

Using these mapped coordinates, DMR-gene associations were defined via the direct overlap of a DMR with defined (1) promoters or (2) enhancers, or (3) the location of a gene within 100kb of a DMR. The level of association is noted for each gene considered (Supplementary Tables 2-3, 5-9, and 11-12).

RNA-seq pre-processing

RNA sequencing reads were processed using a modified version of the SEESAW pipeline [24]. Briefly, two strain-specific transcriptomes for CC019 and CC037 were constructed using the gffread [25] (v0.12.7) function, strain-specific genomes, and strain-specific genome annotation files. The two strain-specific transcriptomes were then concatenated into a single diploid transcriptome, designating the origin strain for each gene in the FASTA definition lines. The diploid transcriptome was indexed using Salmon [26] (v1.9.0) with the following parameter: `--keepDuplicates`. Sequenced reads were mapped to the diploid transcriptome and allelic expression levels were quantified using the Salmon quant function with the following parameters: `-l A --validateMappings --numBootstraps 30`. One male CC037xCC019 F1 sample exhibited poor mapping to the diploid transcriptome (68.8% mapping rate) and, as such, was removed from subsequent expression analysis.

Expression analysis

Allelic expression values were then calculated in R using the fishpond [27] package (v2.8.0). Briefly, a transcript-to-gene mapping file was constructed using the BiomaRt package (v2.58.0) and the `mmusculus_gene_ensembl` mart, extracting the following information for each transcript: `ensembl_transcript_id`, `external_gene_name`, `ensembl_gene_id`, `chromosome_name`, `start_position`, `end_position`. This mapping file was then converted to a GRanges object using the GenomicRanges package (v1.54.1). Salmon quantification output files were then imported using the `importAllelicCounts` function with the mapping file and the following parameter: `format = "wide"`.

For each sample, allelic counts were converted to counts per million (CPM) by dividing by the total sum of the allelic counts for that sample, combined across all genes in the transcript-to-gene mapping file and both alleles, and multiplying by 1e6. Genes were then filtered such that those with a CPM less than 0.5 in at least half of the samples were removed from subsequent analyses. For subsequent allelic analyses, an additional level of filtering was applied in which the allelic counts of inbred parental strains were used to identify genes for which allelic assignments were accurate, using a cutoff of 90% of reads being assigned to the proper allele matching the inbred strain in all inbred samples. For the analysis of XCI-associated genes, the above filtering steps were performed using only the female samples.

In large part due to a lack of transcribed polymorphisms between the two strains analyzed, we are only able to analyze the allele-specific expression of 9.5% of genes in the genome. However, the use of ONT long-read RNA sequencing could improve the ability to quantify allele-specific expression. Between the two selected CC strains, there are 8,024 expressed genes with at least one transcribed polymorphism, whereas we can successfully analyze 4,092 (51.0%).

To gauge the effect of batch on the RNA-seq data, PCA plots showing the allele-specific expression of autosomal genes with correct allelic assignment and expression above the aforementioned cutoff for the six inbred samples which were included in both RNA-seq batches, split by both batch and allele, are shown in Supplementary Fig. 30. Clear clustering of these samples by sample and allele, rather than batch, suggest that the batch effect is much smaller than the biological difference between samples of different strains.

The allelic counts of the filtered genes, using only inbred samples from a single batch, were then analyzed with the swish function from the fishpond package using one of two methods: (1) the

default global allelic imbalance analysis or (2) a differential allelic imbalance analysis. Additional analyses were performed on these filtered genes using total expression data, obtained by summing the allelic counts from both alleles and then converting to CPM. Total expression levels were analyzed using the DESeq2 [28] package (v1.42.0). One female CC019xCC037 F1 sample had a total expression CPM of more than 3 standard deviations away from the mean in 6,927/12,894 expressed genes and, as such, was removed from subsequent expression analyses as an outlier.

To compare methylation and expression, all genes associated with the identified DMRs via direct promoter/enhancer overlap or by genomic proximity were analyzed using a set of tests and conditions specific to each epigenetic inheritance pattern from which the DMR originates, outlined below. Tests for allele-specific overdominance and allele-specific underdominance were not designed as these necessitate a direct comparison in expression between a single phased allele of the F1s and the total expression of the inbreds, which cannot be performed.

Tests:

1. $T_{\text{Strain\_inbred}}$ = DEseq2([total CC019] - [total CC037])
2. $T_{\text{CC019\_F1}}$ = DEseq2([total CC019] - [total F1])
3. $T_{\text{CC037\_F1}}$ = DEseq2([total CC037] - [total F1])
4. T_{Sex} = DEseq2([total female either allele] - [total male either allele])
5. $T_{\text{Allele\_F1}}$ = Swishglobal([F1 CC019 allele] - [F1 CC037 allele])
   Paired comparison
6. $T_{\text{Imprinting}}$ = Swishdifferential(([F1 CC019 allele] - [F1 CC037 allele]))
   Paired differential comparison based on cross direction

For the specified DMR categories listed below, the corresponding tests were used to identify the differentially expressed genes (DEGs), with TRUE being defined as a Benjamini-Hochberg (BH) adjusted p-value ¡ 0.05. BH corrections were performed independently for each test within each associated DMR category:

1. Cis-acting meQTLs

$$T_{\text{Strain\_inbred}} = \text{TRUE}\ \&\ T_{\text{Allele\_F1}} = \text{TRUE}\ \&$$
$$\text{sign}(\log_2 FC(T_{\text{Strain\_inbred}})) = \text{sign}(\log_2 FC(T_{\text{Allele\_F1}}))$$

2. Non-dominant trans-acting meQTLs

$$T_{\text{Strain\_inbred}} = \text{TRUE}\ \&\ T_{\text{Allele\_F1}} = \text{FALSE}$$

3. Dominant trans-acting meQTLs | transvection | paramutation - 19

$$T_{\text{Strain\_inbred}} = \text{TRUE}\ \&$$
$$T_{\text{CC019\_F1}} = \text{TRUE}\ \&\ T_{\text{CC037\_F1}} = \text{FALSE}\ \&$$
$$\text{sign}(\log_2 FC(T_{\text{Strain\_inbred}})) = \text{sign}(\log_2 FC(T_{\text{CC019\_F1}}))$$

4. Dominant trans-acting meQTLs—transvection—paramutation - 37

$$T_{\text{Strain\_inbred}} = \text{TRUE}\ \&$$
$$T_{\text{CC019\_F1}} = \text{FALSE}\ \&\ T_{\text{CC037\_F1}} = \text{TRUE}\ \&$$
$$\text{sign}(\log_2 FC(T_{\text{Strain\_inbred}})) \neq \text{sign}(\log_2 FC(T_{\text{CC037\_F1}}))$$

5. Genomic imprinting

$$T_{\text{Imprinting}} = \text{TRUE} \ \& \\ \text{sign}(\log_2 \frac{\text{"Avg CPM of CC037 allele of 19x37 + 0.01"}}{\text{"Avg CPM of CC019 allele of 19x37 + 0.01"}}) \neq \\ \text{sign}(\log_2 \frac{\text{"Avg CPM of CC037 allele of 37x19 + 0.01"}}{\text{"Avg CPM of CC019 allele of 37x19 + 0.01"}})$$

(A pseudocount of 0.01 is added to ensure that the denominator is not 0)

6. Sex-specific DMRs

$$T_{\text{Sex}} = \text{TRUE}$$

7. Overdominance or underdominance

$$T_{\text{CC037\_F1}} = \text{TRUE} \ \& \ T_{\text{CC019\_F1}} = \text{TRUE} \ \& \\ \text{sign}(\log_2 FC(T_{\text{CC037\_F1}})) = \text{sign}(\log_2 FC(T_{\text{CC019\_F1}}))$$

8. Biallelic dominance

$$T_{\text{Strain\_inbred}} = \text{FALSE} \ \& \ T_{\text{Allele\_F1}} = \text{TRUE}$$

9. Skewed XCI

$$T_{\text{Strain\_inbred}} = \text{FALSE} \ \& \ T_{\text{Allele\_F1}} = \text{TRUE}$$

(This comparison is only performed on female samples)

As the overdominance/underdominance and dominant trans-acting meQTLs/transvection/paramutation comparisons require direct comparisons of expression across batches, the associated DEGs may in part be driven by batch effects.

Transcriptome variant analysis

Heterozygous genetic variants were identified using the nucmer command from MUMmer4 run between the CC019 and CC037 strain-specific transcriptomes, converted to a delta file with SNPs and insertion-deletions (indels) using the `show-snps` function with the following parameters: `-CTlr`, and subsequently converted to a VCF file using the `my-mummer-2-vcf.py` script with the following parameter: `-n`. This process was run once with CC019 as the reference genome and once with CC037 as the reference genome. Genes having at least one transcript with one or more heterozygous transcribed variant were then identified using the transcript-to-gene mapping file and subsequently overlapped with the list of previously defined expressed genes.

MGI gene-disease associations

Gene-disease associations were extracted from the following Mouse Genome Informatics (MGI) databases: Associations of Mouse Genes with DO Diseases and Mouse Models of Human Disease by Human Gene [29]. Associations are described in Supplementary Note and Supplementary Tables 11 and 12.

Read-level methylation plots

Read-level methylation plots were generated for non-dominant trans-acting meQTLs and skewed XCI DMRs using methylartist [30] (v1.2.10). db-nanopolish was run on the Nanopolish methylation call output file for each sample using the following parameter: `-t 2.5`. DMRs were mapped from the intermediate coordinate system into the CC019 and CC037 coordinate systems and subsequently used as the input coordinates (with a 5kb extension) for the methylartist locus function. The CC019 coordinates were used for plotting CC019 inbred and CC019 phased alleles and the CC037 coordinates were used for plotting CC037 inbred and CC037 phased alleles.

Supplementary Note: On various intergenerational epigenetic inheritance patterns

The definitions in the following sections outline the distinct patterns of epigenetic inheritance classified in this analysis and how they are distinguished from one another within the inbred, F1, and F2 generations.

Independent assortment of chromosomes and meiotic recombination in the F1 generation produce the unique genomes of the F2 generation in which the *trans*-acting factors influencing the methylome are inherited independent of the local haplotype (i.e. homozygous CC019 or CC037, or heterozygous CC019/CC037 alleles) of each region over which they influence methylation. Whereas most of these patterns can be distinguished from each other using data from the inbred and F1 generations, three patterns (Dominant *trans*-acting meQTLs, transvection, and paramutation) are indistinguishable in these generations. These three patterns therefore require additional data from the F2 generation to be differentiated from one another (Table 1 and discussion below).

Pattern 1: *Cis*-acting meQTLs

In genomic regions under the control of these *cis*-acting meQTLs, F1 crosses between inbred strains will inherit one allele from each strain, and the CpGs on each allele will exhibit a methylation pattern matching the strain from which the allele was inherited. As such, the *cis*-acting meQTL and its respective methylation pattern are transmitted together from the inbred parental strains to each respective allele of the F1s, creating the observed genotype-specific methylation pattern (Fig. 2a). For this pattern, inbreds and F1 mice are enough to establish the pattern; the F2 mice serve as validation samples.

Pattern 2: Non-dominant *trans*-acting meQTLs

Trans-acting meQTLs can manifest as dominant, in which the presence of a single copy of the dominant allele establishes the dominant methylation pattern on both alleles (Fig. 1c); incomplete dominant, in which individuals heterozygous for the *trans*-acting meQTL exhibit an intermediate methylation level on both alleles (Fig. 4a); or codominant, in which heterozygous individuals exhibit both of the methylation patterns observed in the homozygous individuals on both alleles (Fig. 4b). Within a population of inbred and F1 individuals, incomplete dominant and codominant *trans*-acting meQTLs will each establish intermediate levels of methylation on both alleles of the F1s which fall between the levels observed in the two parental strains, matching pattern 2. Conversely, dominant *trans*-acting meQTLs give rise to pattern 3, discussed further in the next section. Due to the binary nature of DNA methylation at a single CpG in a single cell, intermediate levels of methylation can arise either from variability of methylation at the cellular level or at the CpG level within each cell. Non-dominant (incomplete dominant or codominant) *trans*-acting meQTLs violate Mendelian inheritance, as neither allele is completely dominant nor recessive in its regulation of methylation.

As noted in the Main Text, some DMRs exhibit a genotype- and allele-specific methylation pattern in the F2 generation that is smaller than observed in the inbred generation and roughly the same as in the F1 generation (Supplementary Data 1). This pattern suggests these regions are likely regulated by both *cis*- and non-dominant *trans*-acting meQTLs, and that the methylation differences associated with the homozygous non-dominant *trans*-acting meQTLs in the inbred generation

are removed in both the F1 and F2 generations, while the effects of the *cis*-acting meQTLs are maintained.

Patterns 3-5: Dominant *trans*-acting meQTLs, transvection, and paramutation

Within the inbred and F1 generations, dominant *trans*-acting meQTLs, transvection, and paramutation will all establish the same DNA methylation patterns. As such, data from the F2 generation is required to distinguish patterns established by each of these three mechanisms.

As noted in the Main Text, this pattern can conceivably arise from three distinct underlying mechanisms: dominant *trans*-acting meQTLs, transvection, or paramutation, and additional data from the F2 generation is required to distinguish these potential mechanisms (Extended Data Fig. 4). Briefly, dominant *trans*-acting meQTLs require only a single copy of the dominant allele to establish its associated methylation state on both alleles, while transvection and paramutation involve the alteration of methylation on one allele due to the presence of its homolog. In the case of transvection, this inheritance of this newly established methylation pattern is regulated by the inheritance of its associated *cis*-acting genetic variant, whereas, in the event of paramutation, it is inherited by all members of the following generation independent of their local or distal genetic composition.

Dominant *trans*-acting meQTLs require only a single copy of the dominant genetic variant to establish a particular methylation pattern on both alleles. Consequently, in regions under the control of this regulatory mechanism, inbred strains will exhibit distinct methylation patterns, while both alleles of the heterozygous F1 crosses will inherit the dominant methylation pattern (Extended Data Fig. 4a). In the F2 generation, the methylation over the region will be determined by the zygosity of the individual over the *trans*-acting factor. Dominant *trans*-acting meQTLs do not violate Mendelian inheritance.

In the case of transvection, one allele in a heterozygous individual acquires the methylation state of its dominant homolog [31]. Thus, though the parental strains exhibit distinct methylation patterns, both alleles of the F1s will exhibit a methylation pattern matching that of parental strain from which the dominant homolog originates (Extended Data Fig. 4b). In the F2 generation, the methylation over the region will be determined by the local zygosity of the individual. Transvection events do not violate Mendelian inheritance. Transvection naturally occurs in plants [32], Drosophila [33–36], and fungi [37], and has been observed within transgenic mammalian genomes under certain conditions [38, 39]. However, it has not previously been documented in a non-transgenic mammalian genome, nor has it been mapped genome-wide.

Paramutation involves the alteration of DNA methylation on a paramutable allele due to the presence of its homologous paramutant allele. However, unlike dominant *trans*-acting meQTLs and transvection, this altered methylation state is subsequently heritable independent of the dominant paramutant allele or other *trans*-acting factors [40–42] (Extended Data Fig. 4c). In the F2 generation, methylation over the region will match the altered methylation state in all individuals, regardless of their local or distal genotype. As such, the initiation of paramutation is a hybrid effect, and the heritable methylation pattern can be established by either a transvection event or a dominant *trans*-acting factor. Paramutation violates Mendelian inheritance, as the dominant methylation pattern and associated phenotypes can be inherited even in the absence of the dominant allele. Similar to transvection, paramutation has been previously identified in plants [40–42], Drosophila [40, 43], and transgenic mice [40–42], though it has not been previously observed in non-transgenic mammals nor mapped genome-wide.

Patterns 6-10: Emergent epigenetic inheritance patterns

Emergent epigenetic inheritance patterns can be further categorized into (1) overdominance and underdominance, in which both alleles of heterozygous offspring exhibit higher or lower methylation levels than the inbred parental strains, respectively (Fig. 6a); (2) allele-specific overdominance and allele-specific underdominance, in which just one allele of heterozygous offspring exhibit higher or lower methylation levels than inbred parental strains as well as the other allele of the cross, respectively (Fig. 6b); (3) biallelic dominance, in which one allele of the heterozygous offspring exhibit higher methylation than the inbred parental strains while the other allele exhibits lower methylation (Fig. 6c); (4) polar overdominance and polar underdominance, in which both alleles of one cross direction of the heterozygous offspring exhibit higher or lower methylation levels than inbred parental strains as well as both alleles of the other cross direction; and (5) bipolar dominance, in which both alleles of one cross direction of the heterozygous offspring exhibit higher methylation than the inbred parental strains while both alleles of the other cross direction exhibit lower methylation. Each of these patterns violate Mendelian inheritance, as the offspring inherit methylation states which do not match either parental allele. These patterns can be caused by many distinct regulatory mechanisms and the methylation state in subsequent generations depends on the exact mechanism driving the pattern. For this reason, these patterns were not selected as targets in the F2 generation.

Pattern 11: Genomic imprinting

Genomic imprinting violates Mendelian inheritance, as methylation and associated phenotypes are influenced by the parent-of-origin, rather than solely by the identity of the allele.

Pattern 12: Sex-specific DNA methylation

Sex-specific regulatory effects violate Mendelian inheritance because the methylation pattern and associated phenotypes depend only on the offspring's sex rather than the identity of the allele.

X Chromosomal epigenetic inheritance patterns

Skewed XCI has been previously reported among several of the CC founder strains [44]. Over the QTL identified in mice which regulates XCI, called *Xce*, CC019 originates from WSB/EiJ, which carries the Xce^b allele, while CC037 originates from NOD/ShiLtJ, which carries the Xce^g allele. The Xce^g allele has been shown to be preferentially inactivated when combined with Xce^b allele [44].

Supplementary Note: Additional Results

We note that the overall difference in the number of observed patterns between the liver and muscle is likely, in part, due to the lower sample size of muscle included in this analysis.

Patterns 3-5: Dominant *trans*-acting meQTLs, transvection, and paramutation

Among the remaining two candidate regions profiled in the F2s which were not classified as paramutation, neither exhibited a methylation pattern which indicates clear regulation by a dominant *trans*-acting meQTL or a transvection event (Extended Data Fig. 7, Supplementary Fig. 25, Supplementary Data 1). Rather, these regions exhibit a methylation pattern which cannot be definitively classified into any of these three regulatory mechanisms, as we do not observe the presence of a single methylation pattern across all members the F2 generation (suggesting paramutation), nor an identical pattern in the F2 generation as is observed in the inbred and F1 generations (suggesting transvection), nor a clear lack of association between the local genetics and the methylation over the region (suggesting a dominant *trans*-acting meQTL). As such, it is likely that these regions are under the control of additional complex regulatory mechanisms. The identification of a single confirmed example of paramutation suggests rarity of this intergenerational epigenetic inheritance pattern.

IAPs, as well as many other forms of TEs, are typically repressed via epigenetic mechanisms including DNA methylation [45], suggesting that the presence of the identified methylation pattern may serve to repress the IAP in the CC037 alleles of the heterozygous F1 generation in both liver and muscle, and that this IAP may not be repressed in the inbred CC037 samples for which methylation is significantly lower. Notably, IAPs have been shown to be capable of maintaining their methylation state through the waves of DNA demethylation which occur during gametogenesis and embryogenesis, allowing for this repressive methylation pattern to be maintained in subsequent generations [46].

Regarding the highly likely example of intergenerational paramutation identified over the *Vps37c* IAP, there remains the possibility that this methylation pattern is driven by a dominant *trans*-acting meQTL. This is because methylation over the IAP cannot be analyzed within the five F2 samples which are homozygous for the CC019 allele over this region, and we cannot exclude the possibility that each of the 14 remaining F2s carry at least one copy of a dominant *trans*-acting meQTL originating from the CC019 strain which regulates methylation over this IAP. Based upon the genotyping data of this set of 14 F2 samples, 93.8% (2,304/2,455) of SNPs which differ between CC019 and CC037 and which were successfully genotyped in all 19 selected F2s have at least one sample which is homozygous for the CC037 allele (Extended Data Fig. 5c). As such, we can confidently rule out these regions of the genome, as they are unable to harbor a dominant *trans*-acting meQTL which would exhibit the observed methylation pattern in these samples, further supporting that the methylation in this region is highly likely to be driven by a paramutation event. Similarly, there also remains the possibility that highly likely example of intergenerational paramutation identified over the *Capn11* IAP is driven by a dominant *trans*-acting meQTL for which none of the F2 samples that contain the IAP have the non-dominant allele. Based on the genotyping data of the 12 F2 samples which contain at least one copy of the *Capn11* IAP, 99.8% (2,451/2,455) of SNPs which differ between CC019 and CC037 and which were successfully genotyped in all 19 selected F2s have at least one sample which is homozygous for the CC037 allele (Extended Data Fig. 5d). As such, we can confidently rule out these regions of the genome, providing additional support that the methylation pattern over this IAP is also highly likely to be regulated

by a paramutation event.

A technical point: the coverage of the F2 capture target region of the *Capn11* DMR was an average of $\approx$ 51X per sample/allele due to its proximity to the *Capn11* DMR.

Pattern 11: Genomic imprinting

While $\approx$ 54% of the autosomal imprinted regions identified in each tissue directly overlap one another (54.5% and 53.9% for liver and muscle, respectively), 59 (76.6%) of the imprinted regions identified in liver are within 100kb of an imprinted region identified in the muscle. This indicates that, while these DMRs may not lie over the exact same CpGs in different tissues, they tend to exist in the same regions/domains. However, we observe several exceptions in the form of tissue-specific imprinted genes, including *Zswim9* and *Asb4*, which exhibit parent-of-origin-specific methylation only in the liver and muscle, respectively (Fig. 7c-d).

Association of epigenetic inheritance patterns with inherited diseases

A significant consequence of these non-Mendelian inheritance patterns is that phenotypes associated with the methylation pattern in these regions will not necessarily track with the genotype. To investigate the potential relationship between these epigenetic inheritance patterns and inherited disease, we intersected the set of genes associated with these DMRs via direct promoter/enhancer overlap or by genomic proximity (within 100kb of the DMR) with curated databases of mouse and human disease-gene associations from Mouse Genome Informatics (MGI; see Methods). It is important to note that, because these epigenetic inheritance patterns were identified in a disease-agnostic context, the overlap with these MGI databases does not imply a causal link between the methylation pattern and inherited diseases reported below. These associations are based on relatively broad DMR-gene associations and further functional studies are required to determine if these DMRs fundamentally contribute to these disorders.

Pattern 1: *cis*-acting meQTLs

For the *cis*-acting meQTLs, this analysis identified 2,201 such overlaps, suggesting that many of the genes associated with these *cis*-acting meQTLs may be relevant to human disease (Supplementary Tables 11-12).

Pattern 2: Non-dominant *trans*-acting meQTLs

Among the genes associated with the non-dominant *trans*-acting meQTLs via direct promoter/enhancer overlap or by genomic proximity, 47 have been implicated in the etiology of diseases in the MGI databases (Supplementary Tables 11-12). In the case of non-dominant *trans*-acting meQTLs which contribute to disease phenotypes, incomplete penetrance and variable expressivity may occur as heterozygous individuals with intermediate levels of methylation may not always reach molecular thresholds for disease manifestation or may exhibit more mild symptoms than homozygous individuals or even other heterozygous individuals. As such, these non-dominant *trans*-acting meQTLs may be missed by purely genetic analyses attempting to identify genetic variants contributing to disease. Indeed, several of the associated diseases exhibit these complex forms of inheritance. Among these are trichodentoosseous syndrome, associated with *Dlx3*, which exhibits clinical variability in the absence of genetic heterogeneity for which the authors hypothesize potential epigenetic or environmental contributions to its etiology [47]; Currarino syndrome, associated

with *Mnx1*, which exhibits both variable expressivity as well as reduced penetrance [48, 49]; and nail-patella syndrome, associated with *Lmx1b*, for which the effect of the driving mutation may be influenced by variants on the non-mutant allele acting in trans [50]. For each of these three genes, a DMR identified in the liver exhibiting regulation by a non-dominant *trans*-acting meQTL overlaps the promoter region.

Patterns 3-5: Dominant *trans*-acting meQTLs, transvection, and paramutation

25 genes associated with the dominant *trans*-acting meQTL/transvection/paramutation DMRs via direct promoter/enhancer overlap or by genomic proximity have been implicated in the etiology of diseases in the MGI databases (Supplementary Tables 11-12), several of which exhibit atypical patterns of inheritance. Among these are the variable phenotypes observed in the absence of genetic heterogeneity in Timothy syndrome and common variable immunodeficiency 8, associated with *Cacna1c* (for which a DMR identified in the liver overlaps the promoter) and *Lrba*, respectively. This suggests unexplained genetic or environmental factors associated with these diseases that may be mediated through the epigenome or via other forms of transcriptional regulation [51, 52]. In the investigation of the complex inheritance patterns of these disorders, paramutation, transvection, and dominant *trans*-acting meQTLs were likely not considered, nor was the epigenome sequenced and evaluated as a potential avenue of intergenerational inheritance of the disease phenotype. Given the results reported here, these additional modes of inheritance should be considered in family studies.

Patterns 6-10: Emergent epigenetic inheritance patterns

Emergent epigenetic inheritance patterns which contribute to disease phenotypes are likely to violate typical Mendelian inheritance, as the disease will be associated with the emergent pattern in addition to or in instead of the genetic variant(s) being considered. As such, a higher or lower proportion of individuals may be affected than can be explained purely by Mendelian genetics. Furthermore, as the emergent epigenetic inheritance pattern may be susceptible to additional factors such as environmental exposures, the associated disorders may exhibit a high degree of phenotypic variability. 54 genes associated with these emergent epigenetic inheritance patterns via direct promoter/enhancer overlap or by genomic proximity have been implicated in the etiology of diseases in the MGI databases (Supplementary Tables 11-12) and several of these disorders demonstrate these complex patterns of inheritance. Among these are combined pituitary hormone deficiency-5, associated with *Hesx1*, for which both humans and mice exhibit incomplete penetrance of the heterozygous phenotype [53]; as well as developmental and epileptic encephalopathy 94, associated with *Chd2* for which an allele-specific overdominance DMR identified in the liver overlaps the promoter, which exhibits clinical heterogeneity within and between families with the same mutations [54, 55].

Pattern 11: Genomic imprinting

We identified parent-of-origin-specific methylation patterns overlapping the following genes which, to our knowledge, have not been previously identified as imprinted: *Scn8a* (Fig. 7e), a sodium voltage-gated channel subunit involved in membrane depolarization during the formation of action potentials in neurons; *Pcdhb4*, a protocadherin involved in neuronal cell-cell connections; and *Fry*, a microtubule binding protein involved in maintaining the integrity of mitotic centrosomes (each identified in the liver); as well as *Socs5*, involved in the suppression of cytokine signaling

(identified in the muscle) [56]. Another parent-of-origin-specific methylation pattern was identified over *Cntnap1*, which has been predicted as a potential imprinted gene based on a DNA sequence analysis [57], though this was not validated in subsequent studies performed in the brain [58].

Pattern 12: Sex-specific DNA methylation

198 genes associated with the sex-specific DMRs via direct promoter/enhancer overlap or by genomic proximity have been implicated in the etiology of diseases from the MGI databases (Supplementary Tables 11-12), many of which exhibit sex disparities in their clinical features. For example, skewed sex ratios are observed in type II diabetes mellitus [59] and nephroblastoma [60], associated with *Pik3c2g* and *Pou6f2*, respectively. Furthermore, distinct clinical symptoms are observed between men and women with colorectal cancer [61] as well as inflammatory bowel diseases [62], associated with *Rac1* (for which a sex-specific DMR identified in the liver overlaps an enhancer) and *Ano6*, respectively. Sex-specific regulation of DNA methylation and gene expression have also been implicated in the etiology of several disorders such as non-alcoholic fatty liver disease (NAFLD) [63, 64] and hepatocellular carcinoma (HCC) [65] as well as critical biological processes such as drug metabolism [66, 67]. Given the abundance of these sex-specific methylation patterns, their effect on disease phenotypes and presentation should be considered when examining disorders which exhibit sexual dimorphism.

Supplementary Discussion

Liver and muscle were chosen for analysis in part because these are relatively homogeneous tissues [68, 69] (Methods), which reduces the possibility of cell type confounding. However, it remains possible that differences in cell type compositions could underlie a subset of the reported DMRs. Genetic variants have previously been associated with cell type compositions in blood [70–73], and cell type composition is well appreciated as a possible confounder of molecular phenotypes such as DNA methylation and gene expression. However, within this analysis we rely extensively on allele-specific methylation and expression information in which the two alleles are measured in the same cells, thereby reducing the possible confounding effect of cell type heterogeneity. Additional studies performed with single-cell resolution will enable the identification of potential relationships between these various epigenetic inheritance patterns and differences in cell type heterogeneity across generations.

All samples utilized in this study were obtained from young adult mice between roughly 4-6 months of age. While we cannot definitively rule out minor effects of chronological age, the robustness of the identified patterns across individuals and tissues, combined with the methylation differences associated with each pattern within this narrow range of ages, strongly suggests that age is unlikely to be the primary determinant of these intergenerational epigenetic inheritance patterns.

Numerous mechanisms have been identified which regulate genomic imprinting, including insulators, non-coding RNAs, histone modifications, DNA methylation, and chromatin dynamics [74]. In contrast, little is known regarding the mechanisms driving the other non-Mendelian inheritance patterns discussed here. While IAPs are more commonly found in mice than in humans, they have been identified to be clinically relevant to human health [75, 76], although methylation patterns over these human IAPs have not been previously considered. Other classes of ERVs present in humans have been shown to exhibit methylation-associated repression [77, 78], which may be regulated in a similar manner leading to complex inheritance patterns such as paramutation. Identifying which mechanisms establish the other epigenetic inheritance patterns we describe will require further investigation.

In human populations when compared to model organisms. Furthermore, the increased levels of genetic variation present in human populations, an issue which does not affect model organisms for which genetics and breeding can be tightly controlled, adds significant additional variability and complexity to the analysis. However, an analysis of methylation in human trios identified more than 6.5 million CpGs which exhibit inheritance patterns that are incompatible with Mendel's laws [79]. As such, studies of pedigrees with complex inheritance that do not fit traditional Mendelian patterns should also include epigenetic analyses.

References

[1] The Collaborative Cross, a community resource for the genetic analysis of complex traits. *Nature Genetics* **36**.11 (2004): 1133–1137. DOI: 10.1038/ng1104-1133.

[2] JS Sigmon, MW Blanchard, RS Baric, TA Bell, J Brennan, GA Brockmann, AW Burks, JM Calabrese, KM Caron, RE Cheney, et al. Content and performance of the MiniMUGA genotyping array: a new tool to improve rigor and reproducibility in mouse research. *Genetics* **216**.4 (2020): 905–930. DOI: 10.1534/genetics.120.303596.

[3] AR Quinlan, IM Hall. BEDTools: a flexible suite of utilities for comparing genomic features. *Bioinformatics* **26**.6 (2010): 841–842. DOI: 10.1093/bioinformatics/btq033.

[4] H Su, Z Chen, J Rao, M Najarian, J Shorter, F Pardo Manuel de Villena, L McMillan. The Collaborative Cross Graphical Genome. *bioRxiv* (2019): 858142. DOI: 10.1101/858142.

[5] W Shen, B Sipos, L Zhao. SeqKit2: A Swiss army knife for sequence and alignment processing. *Imeta* **3**.3 (2024): e191. DOI: 10.1002/imt2.191.

[6] H Li. Minimap2: pairwise alignment for nucleotide sequences. *Bioinformatics* **34**.18 (2018). Ed. by I Birol: 3094–3100. DOI: 10.1093/bioinformatics/bty191.

[7] G Marçais, AL Delcher, AM Phillippy, R Coston, SL Salzberg, A Zimin. MUMmer4: A fast and versatile genome alignment system. *PLoS Computational Biology* **14**.1 (2018): e1005944. DOI: 10.1371/journal.pcbi.1005944.

[8] M Martin, M Patterson, S Garg, S O Fischer, N Pisanti, GW Klau, A Schöenhuth, T Marschall. WhatsHap: fast and accurate read-based phasing. *bioRxiv* (2016): 085050. DOI: 10.1101/085050.

[9] JT Simpson, RE Workman, PC Zuzarte, M David, LJ Dursi, W Timp. Detecting DNA cytosine methylation using nanopore sequencing. *Nature Methods* **14**.4 (2017): 407–410. DOI: 10.1038/nmeth.4184.

[10] H Li, B Handsaker, A Wysoker, T Fennell, J Ruan, N Homer, G Marth, G Abecasis, R Durbin. The sequence alignment/map format and SAMtools. *Bioinformatics* **25**.16 (2009): 2078–2079. DOI: 10.1093/bioinformatics/btp352.

[11] A Telatin, P Fariselli, G Birolo. SeqFu: a suite of utilities for the robust and reproducible manipulation of sequence files. *Bioengineering* **8**.5 (2021): 59. DOI: 10.3390/bioengineering8050059.

[12] PJA Cock, T Antao, JT Chang, BA Chapman, CJ Cox, A Dalke, I Friedberg, T Hamelryck, F Kauff, B Wilczynski, MJL de Hoon. Biopython: freely available Python tools for computational molecular biology and bioinformatics. *Bioinformatics* **25**.11 (2009): 1422. DOI: 10.1093/bioinformatics/btp163.

[13] M Lawrence, W Huber, H Pagès, P Aboyoun, M Carlson, R Gentleman, MT Morgan, VJ Carey. Software for computing and annotating genomic ranges. *PLoS Computational Biology* **9**.8 (2013): e1003118. DOI: 10.1371/journal.pcbi.1003118.

[14] K Halter, J Chen, T Priklopil, A Monfort, A Wutz. Cdk8 and Hira mutations trigger X chromosome elimination in naive female hybrid mouse embryonic stem cells. *Chromosome Research* **32**.4 (2024): 12. DOI: 10.1007/s10577-024-09756-w.

[15] KD Hansen, B Langmead, RA Irizarry. BSmooth: from whole genome bisulfite sequencing reads to differentially methylated regions. *Genome Biology* **13**.10 (2012): R83. DOI: 10.1186/gb-2012-13-10-r83.

[16] MJ Ziller, KD Hansen, A Meissner, MJ Aryee. Coverage recommendations for methylation analysis by whole-genome bisulfite sequencing. *Nature Methods* **12**.3 (2015): 230–232. DOI: 10.1038/nmeth.3152.

[17] KD Hansen, W Timp, HC Bravo, S Sabunciyan, B Langmead, OG McDonald, B Wen, H Wu, Y Liu, D Diep, E Briem, K Zhang, RA Irizarry, AP Feinberg. Increased methylation

variation in epigenetic domains across cancer types. *Nature Genetics* **43**.8 (2011): 768–775. DOI: 10.1038/ng.865.

[18] JT Robinson, H Thorvaldsdóttir, W Winckler, M Guttman, ES Lander, G Getz, JP Mesirov. Integrative genomics viewer. *Nature Biotechnology* **29**.1 (2011): 24–26. DOI: 10.1038/nbt.1754.

[19] H Li. *Seqtk: Toolkit for processing sequences in FASTA/Q formats*. https://github.com/lh3/seqtk.

[20] K Korthauer, S Chakraborty, Y Benjamini, RA Irizarry. Detection and accurate false discovery rate control of differentially methylated regions from whole genome bisulfite sequencing. *Biostatistics* **20**.3 (2019): 367–383. DOI: 10.1093/biostatistics/kxy007.

[21] RG Cavalcante, MA Sartor. Annotatr: genomic regions in context. *Bioinformatics* **33**.15 (2017): 2381–2383. DOI: 10.1093/bioinformatics/btx183.

[22] S Durinck, Y Moreau, A Kasprzyk, S Davis, B De Moor, A Brazma, W Huber. BioMart and Bioconductor: a powerful link between biological databases and microarray data analysis. *Bioinformatics* **21**.16 (2005): 3439–3440. DOI: 10.1093/bioinformatics/bti525.

[23] T Gao, J Qian. EnhancerAtlas 2.0: an updated resource with enhancer annotation in 586 tissue/cell types across nine species. *Nucleic Acids Research* **48** (2020): D58–D64.

[24] EY Wu, NP Singh, K Choi, M Zakeri, M Vincent, GA Churchill, CL Ackert-Bicknell, R Patro, MI Love. SEESAW: detecting isoform-level allelic imbalance accounting for inferential uncertainty. *Genome Biology* **24**.1 (2023): 165. DOI: 10.1186/s13059-023-03003-x.

[25] G Pertea, M Pertea. GFF utilities: GffRead and GffCompare. *F1000Research* **9** (2020): 304. DOI: 10.12688/f1000research.23297.2.

[26] R Patro, G Duggal, MI Love, RA Irizarry, C Kingsford. Salmon provides fast and bias-aware quantification of transcript expression. *Nature Methods* **14**.4 (2017): 417–419. DOI: 10.1038/nmeth.4197.

[27] A Zhu, A Srivastava, JG Ibrahim, R Patro, MI Love. Nonparametric expression analysis using inferential replicate counts. *Nucleic Acids Research* **47**.18 (2019): e105–e105. DOI: 10.1093/nar/gkz622.

[28] MI Love, W Huber, S Anders. Moderated estimation of fold change and dispersion for RNA-seq data with DESeq2. *Genome Biology* **15**.12 (2014): 1–21. DOI: 10.1186/s13059-014-0550-8.

[29] JA Blake et al. Mouse Genome Database (MGD): knowledgebase for mouse–human comparative biology. *Nucleic Acids Research* **49** (2021): D981–D987. DOI: 10.1093/nar/gkaa1083.

[30] SW Cheetham, M Kindlova, AD Ewing. Methylartist: tools for visualizing modified bases from nanopore sequence data. *Bioinformatics* **38**.11 (2022): 3109–3112. DOI: 10.1093/bioinformatics/btac292.

[31] T Fukaya, M Levine. Transvection. *Current Biology* **27**.19 (2017): R1047–R1049. DOI: 10.1016/j.cub.2017.08.001.

[32] M Matzke, AJM Matzke, OM Scheid. Inactivation of repeated genes—DNA-DNA interaction? *Homologous Recombination and Gene Silencing in Plants*. Springer Netherlands, 1994: 271–307. DOI: 10.1007/978-94-011-1094-5_12.

[33] R Hopmann, D Duncan, I Duncan. Transvection in the iab-5, 6, 7 region of the bithorax complex of Drosophila: homology independent interactions in trans. *Genetics* **139**.2 (1995): 815–833. DOI: 10.1093/genetics/139.2.815.

[34] IW Duncan. Transvection effects in Drosophila. *Annual Review of Genetics* **36**.1 (2002): 521–556. DOI: 10.1146/annurev.genet.36.060402.100441.

[35] DJ Mellert, JW Truman. Transvection is common throughout the Drosophila genome. *Genetics* **191**.4 (2012): 1129–1141. DOI: 10.1534/genetics.112.140475.

[36] JL Chen, KL Huisinga, MM Viering, SA Ou, Ct Wu, PK Geyer. Enhancer action in trans is permitted throughout the Drosophila genome. *Proceedings of the National Academy of Sciences* **99**.6 (2002): 3723–3728. DOI: 10.1073/pnas.062447999.
[37] R Aramayo, RL Metzenberg. Meiotic transvection in fungi. *Cell* **86**.1 (1996): 103–113. DOI: 10.1016/s0092-8674(00)80081-1.
[38] M Rassoulzadegan, M Magliano, F Cuzin. Transvection effects involving DNA methylation during meiosis in the mouse. *The EMBO Journal* **21**.3 (2002): 440–450. DOI: 10.1093/emboj/21.3.440.
[39] JD Rodriguez, DA Myrick, I Falciatori, MA Christopher, TW Lee, GJ Hannon, DJ Katz. A model for epigenetic inhibition via transvection in the mouse. *Genetics* **207**.1 (2017): 129–138. DOI: 10.1534/genetics.117.201913.
[40] JB Hollick. Paramutation and related phenomena in diverse species. *Nature Reviews Genetics* **18**.1 (2017): 5–23. DOI: 10.1038/nrg.2016.115.
[41] VL Chandler. Paramutation: from maize to mice. *Cell* **128**.4 (2007): 641–645. DOI: 10.1016/j.cell.2007.02.007.
[42] VL Chandler, M Stam. Chromatin conversations: mechanisms and implications of paramutation. *Nature Reviews Genetics* **5**.7 (2004): 532–544. DOI: 10.1038/nrg1378.
[43] A de Vanssay, AL Bougé, A Boivin, C Hermant, L Teysset, V Delmarre, C Antoniewski, S Ronsseray. Paramutation in Drosophila linked to emergence of a piRNA-producing locus. *Nature* **490**.7418 (2012): 112–115. DOI: 10.1038/nature11416.
[44] KY Sun, D Oreper, SA Schoenrock, R McMullan, P Giusti-Rodríguez, P Zhabotynsky, DR Miller, LM Tarantino, F Pardo-Manuel de Villena, W Valdar. Bayesian modeling of skewed X inactivation in genetically diverse mice identifies a novel Xce allele associated with copy number changes. *Genetics* **218**.1 (2021): iyab034. DOI: 10.1093/genetics/iyab034.
[45] Ö Deniz, JM Frost, MR Branco. Regulation of transposable elements by DNA modifications. *Nature Reviews Genetics* **20**.7 (2019): 417–431. DOI: 10.1038/s41576-019-0106-6.
[46] N Lane, W Dean, S Erhardt, P Hajkova, A Surani, J Walter, W Reik. Resistance of IAPs to methylation reprogramming may provide a mechanism for epigenetic inheritance in the mouse. *Genesis* **35**.2 (2003): 88–93. DOI: 10.1002/gene.10168.
[47] JA Price, JT Wright, K Kula, DW Bowden, TC Hart. A common DLX3 gene mutation is responsible for tricho-dento-osseous syndrome in Virginia and North Carolina families. *Journal of Medical Genetics* **35**.10 (1998): 825–828. DOI: 10.1136/jmg.35.10.825.
[48] KW Ashcraft, TM Holder. Hereditary presacral teratoma. *Journal of Pediatric Surgery* **9**.5 (1974): 691–697. DOI: 10.1016/0022-3468(74)90107-9.
[49] IS Kim, Sy Oh, SJ Choi, JH Kim, KH Park, HK Park, JW Kim, CS Ki. Clinical and genetic analysis of HLXB9 gene in Korean patients with Currarino syndrome. *Journal of Human Genetics* **52**.8 (2007): 698–701. DOI: 10.1007/s10038-007-0173-y.
[50] JH Renwick. Nail-patella syndrome: Evidence for modification by alleles at the main locus. *Annals of Human Genetics* **21**.2 (1956): 159–169. DOI: 10.1111/j.1469-1809.1971.tb00278.x.
[51] R Bauer, KW Timothy, A Golden. Update on the Molecular Genetics of Timothy Syndrome. *Frontiers in Pediatrics* **9** (2021): 668546. DOI: 10.3389/fped.2021.668546.
[52] A Alangari, A Alsultan, N Adly, MJ Massaad, IS Kiani, A Aljebreen, E Raddaoui, AK Almomen, S Al-Muhsen, RS Geha, FS Alkuraya. LPS-responsive beige-like anchor (LRBA) gene mutation in a family with inflammatory bowel disease and combined immunodeficiency. *Journal of Allergy and Clinical Immunology* **130**.2 (2012): 481–488. DOI: 10.1016/j.jaci.2012.05.043.

[53] PQ Thomas et al. Heterozygous HESX1 mutations associated with isolated congenital pituitary hypoplasia and septo-optic dysplasia. *Human Molecular Genetics* **10**.1 (2001): 39–45. DOI: 10.1093/hmg/10.1.39.

[54] AK Petersen, H Streff, M Tokita, BL Bostwick. The first reported case of an inherited pathogenic CHD2 variant in a clinically affected mother and daughter. *American Journal of Medical Genetics Part A* **176**.7 (2018): 1667–1669. DOI: 10.1002/ajmg.a.38835.

[55] K Hong, P Bjerregaard, I Gussak, R Brugada. Short QT syndrome and atrial fibrillation caused by mutation in KCNH2. *Journal of Cardiovascular Electrophysiology* **16**.4 (2005): 394–396. DOI: 10.1046/j.1540-8167.2005.40621.x.

[56] G Stelzer, N Rosen, I Plaschkes, S Zimmerman, M Twik, S Fishilevich, TI Stein, R Nudel, I Lieder, Y Mazor, S Kaplan, D Dahary, D Warshawsky, Y Guan-Golan, A Kohn, N Rappaport, M Safran, D Lancet. The GeneCards suite: from gene data mining to disease genome sequence analyses. *Current Protocols in Bioinformatics* **54** (2016): 1.30.1–1.30.33. DOI: 10.1002/cpbi.5.

[57] PP Luedi, AJ Hartemink, RL Jirtle. Genome-wide prediction of imprinted murine genes. *Genome Research* **15** (2005): 875–884. DOI: 10.1101/gr.3303505.

[58] RG Tuskan, S Tsang, Z Sun, J Baer, E Rozenblum, X Wu, DJ Munroe, KM Reilly. Real-time PCR analysis of candidate imprinted genes on mouse chromosome 11 shows balanced expression from the maternal and paternal chromosomes and strain-specific variation in expression levels. *Epigenetics* **3**.1 (2008): 43–50. DOI: 10.4161/epi.3.1.5469.

[59] A Kautzky-Willer, J Harreiter, G Pacini. Sex and gender differences in risk, pathophysiology and complications of type 2 diabetes mellitus. *Endocrine Reviews* **37**.3 (2016): 278–316. DOI: 10.1210/er.2015-1137.

[60] AA Okbah, HA Al-Shamahy. Nephroblastoma (Wilms' Tumor): Sex and Age Distribution and Correlation Rate with Ages, Sex, And Kidney Side in Sana'a City, Yemen. *Archives of Gynaecology and Women Health* **1** (2022). DOI: 10.58489/2836-497X/001.

[61] SE Kim, HY Paik, JE Lee, N Kim, MK Sung. Sex-and gender-specific disparities in colorectal cancer risk. *World Journal of Gastroenterology* **21**.17 (2015): 5167–5175. DOI: 10.3748/wjg.v21.i17.5167.

[62] WA Goodman, IP Erkkila, TT Pizarro. Sex matters: impact on pathogenesis, presentation and treatment of inflammatory bowel disease. *Nature Reviews Gastroenterology & Hepatology* **17**.12 (2020): 740–754. DOI: 10.1038/s41575-020-0354-0.

[63] M Vachher, S Bansal, B Kumar, S Yadav, A Burman. Deciphering the role of aberrant DNA methylation in NAFLD and NASH. *Heliyon* **8**.10 (2022): e11119. DOI: 10.1016/j.heliyon.2022.e11119.

[64] VP Tryndyak, RA Willett, MI Avigan, AJ Sanyal, FA Beland, I Rusyn, IP Pogribny. Non-alcoholic fatty liver disease-associated DNA methylation and gene expression alterations in the livers of Collaborative Cross mice fed an obesogenic high-fat and high-sucrose diet. *Epigenetics* **17**.11 (2022): 1462–1476. DOI: 10.1080/15592294.2022.2043590.

[65] W Ye, S Siwko, RYL Tsai. Sex and race-related DNA methylation changes in hepatocellular carcinoma. *International Journal of Molecular Sciences* **22**.8 (2021): 3820. DOI: 10.3390/ijms22083820.

[66] L Yang, Y Li. Sex differences in the expression of drug-metabolizing and transporter genes in human liver. *Journal of Drug Metabolism & Toxicology* **3**.3 (2012). DOI: 10.4172/2157-7609.1000119.

[67] CG Penaloza, B Estevez, DM Han, M Norouzi, RA Lockshin, Z Zakeri. Sex-dependent regulation of cytochrome P450 family members Cyp1a1, Cyp2e1, and Cyp7b1 by methylation of DNA. *The FASEB Journal* **28**.2 (2014): 966–977. DOI: 10.1096/fj.13-233320.

[68] S Ben-Moshe, S Itzkovitz. Spatial heterogeneity in the mammalian liver. *Nature Reviews Gastroenterology & Hepatology* **16**.7 (2019): 395–410. DOI: 10.1038/s41575-019-0134-x.

[69] Y Lai, I Ramírez-Pardo, J Isern, J An, E Perdiguero, AL Serrano, J Li, E García-Domínguez, J Segalés, P Guo, et al. Multimodal cell atlas of the ageing human skeletal muscle. *Nature* **629**.8010 (2024): 154–164. DOI: 10.1038/s41586-024-07348-6.

[70] CHARGE Consortium Hematology Working Group. Meta-analysis of rare and common exome chip variants identifies S1PR4 and other loci influencing blood cell traits. *Nature Genetics* **48**.8 (2016): 867–896. DOI: 10.1038/ng.3607.

[71] WJ Astle, H Elding, T Jiang, D Allen, D Ruklisa, AL Mann, D Mead, H Bouman, F Riveros-Mckay, MA Kostadima, et al. The allelic landscape of human blood cell trait variation and links to common complex disease. *Cell* **167**.5 (2016): 1415–1429.e19. DOI: 10.1016/j.cell.2016.10.042.

[72] MH Chen, LM Raffield, A Mousas, S Sakaue, JE Huffman, A Moscati, B Trivedi, T Jiang, P Akbari, D Vuckovic, et al. Trans-ethnic and ancestry-specific blood-cell genetics in 746,667 individuals from 5 global populations. *Cell* **182**.5 (2020): 1198–1213. DOI: 10.1016/j.cell.2020.06.045.

[73] AP Reiner, G Lettre, MA Nalls, SK Ganesh, R Mathias, MA Austin, E Dean, S Arepalli, A Britton, Z Chen, et al. Genome-wide association study of white blood cell count in 16,388 African Americans: the continental origins and genetic epidemiology network (COGENT). *PLoS Genetics* **7**.6 (2011): e1002108. DOI: 10.1371/journal.pgen.1002108.

[74] DP Barlow, MS Bartolomei. Genomic imprinting in mammals. *Cold Spring Harbor Perspectives in Biology* **6**.2 (2014): a018382. DOI: 10.1101/cshperspect.a018382.

[75] RF Garry, CD Fermin, DJ Hart, SS Alexander, LA Donehower, H Luo-Zhang. Detection of a human intracisternal A-type retroviral particle antigenically related to HIV. *Science* **250**.4984 (1990): 1127–1129. DOI: 10.1126/science.1701273.

[76] DM Sander, S Szabo, WR Gallaher, JE Deas, JJ Thompson, Y Cao, H Luo-Zhang, LG Liu, I Colmegna, J Koehler, LR Espinoza, SS Alexander, DJ Hart, DM Tom, CD Fermin, JJ Jaspan, PC Kulakosky, SA Tenenbaum, RB Wilson, RF Garry. Involvement of human intracisternal A-type retroviral particles in autoimmunity. *Microscopy Research and Technique* **68**.3–4 (2005): 222–234. DOI: 10.1002/jemt.20234.

[77] P Turelli, N Castro-Diaz, F Marzetta, A Kapopoulou, C Raclot, J Duc, V Tieng, S Quenneville, D Trono. Interplay of TRIM28 and DNA methylation in controlling human endogenous retroelements. *Genome Research* **24**.8 (2014): 1260–1270. DOI: 10.1101/gr.172833.114.

[78] D Reiss, Y Zhang, DL Mager. Widely variable endogenous retroviral methylation levels in human placenta. *Nucleic Acids Research* **35**.14 (2007): 4743–4754. DOI: 10.1093/nar/gkm455.

[79] A Díez-Villanueva, B Martín, F Moratalla-Navarro, FD Morón-Duran, I Galván-Femenía, M Obón-Santacana, A Carreras, R de Cid, MA Peinado, V Moreno. Identification of intergenerational epigenetic inheritance by whole genome DNA methylation analysis in trios. *Scientific Reports* **13**.1 (2023): 21266. DOI: 10.1038/s41598-023-48517-3.

Glossary of Terms

Allele-specific overdominance An emergent epigenetic inheritance pattern in which there is an increase in DNA methylation on one allele of the offspring relative to their parents.

Allele-specific underdominance An emergent epigenetic inheritance pattern in which there is a decrease in DNA methylation on one allele of the offspring relative to their parents.

Biallelic dominance An emergent epigenetic inheritance pattern in which there is an increase in DNA methylation on one allele of the offspring relative to their parents and a decrease in DNA methylation on the other allele.

Bipolar dominance An emergent epigenetic inheritance pattern in which there is an increase in DNA methylation in one cross direction of the offspring relative to their parents and a decrease in DNA methylation in the other cross direction. Bipolar dominance likely involves a combination of genomic imprinting and additional complex regulatory factors.

***Cis*-acting** Factors which can only exert their influence over sites located on the same allele and in close genomic proximity to them.

***Cis*-acting meQTL** An meQTL which acts in *cis*.

Codominant *trans*-acting meQTL A non-dominant *trans*-acting meQTL in which the lack of a dominant variant leads to two distinct epi-alleles within each genetic allele, one matching the methylation pattern of each meQTL.

Cross direction The designation of the maternal and paternal contributions in a genetic cross, represented by the maternal strain followed by the paternal strain (e.g. Maternal Strain A x Paternal Strain B).

Dominant *trans*-acting meQTL A *trans*-acting meQTL for which one of the two alleles dominates over the other, producing a methylation pattern on both alleles which matches that of the dominant meQTL. Methylation patterns in the F2 generation are determined by the zygosity of the sample over the *trans*-acting factor.

Emergent epigenetic inheritance pattern Epigenetic inheritance patterns in which at least one allele of the heterozygous offspring exhibits a novel DNA methylation pattern which is not found in either parental strain. Emergent patterns must involve a *trans*-acting factor, though they may also involve additional *cis*-acting factors.

Epi-allele The presence of two (or more) distinct patterns of DNA methylation at a particular locus. In the literature, this often refers to epigenetic variation between individuals. Here, we primarily use this term to refer to epigenetic variation within individuals, particularly within the same genetic allele.

Epigenetics All factors within a cell which are heritable across cell division, other than the DNA sequence itself. Epigenetics includes DNA methylation, histone post-translational modifications, chromatin accessibility, and broader 3D chromatin structure.

Epigenetic inheritance pattern The inheritance pattern of a particular epigenetic trait between parents and their offspring. Here, we use this term to refer specifically to the intergenerational inheritance of the epigenetic trait.

F1 generation The generation made by crossing two inbred strains. These individuals are heterozygous throughout their entire genome, as meiotic recombination and independent assortment of chromosomes occur across two identical alleles during meiosis in the inbred generation.

F2 generation The generation made by crossing two members of the F1 generation. These individuals will have regions of their genome which are homozygous for each strain and regions which are heterozygous due to meiotic recombination and independent assortment of chromosomes which occurs across the two heterozygous alleles during meiosis in the F1 generation. Each individual within this generation will be genetically distinct from one another.

Genomic imprinting Parent-of-origin-specific DNA methylation, i.e. distinct DNA methylation patterns present on the maternal and paternal alleles. Genomic imprinting can be further influenced by *cis*- or *trans*-acting factors.

Genomic proximity The linear distance (at the level of DNA sequence) between two genomic regions located on the same chromosome.

Genomic region A particular segment of DNA within the genome.

Identical by descent (IBD) Genomic regions in which there exists no genetic divergence between the founder strains being considered. Conventionally, these have no clear size definition. Here, we define them as regions which cannot be phased using read lengths of 25kb.

Incomplete dominant *trans*-acting meQTL A non-dominant *trans*-acting meQTL in which the lack of a dominant variant leads to an intermediate level of methylation across both alleles of heterozygous offspring.

Methylation quantitative trait locus (meQTL) A genetic variant which is capable of influencing DNA methylation over particular CpG sites in the genome.

Non-dominant *trans*-acting meQTL A *trans*-acting meQTL for which neither of the two alleles fully dominates over the other, producing a methylation pattern on both alleles which is a mixture of the patterns associated with each meQTL in heterozygous offspring. This pattern includes both incomplete dominant and codominant *trans*-acting meQTLs.

Overdominance An emergent epigenetic inheritance pattern in which there is an increase in DNA methylation on both alleles of the offspring relative to their parents.

Paramutation The heritable alteration of DNA methylation of a paramutable allele due to the presence of its homologous paramutant allele. Paramutation can be caused by *cis*- or *trans*-acting factors (i.e. initiated by transvection or a dominant *trans*-acting meQTL, respectively) but are ultimately heritable in subsequent generations independent of the dominant (paramutant) allele. Methylation patterns in the F2 generation match the altered methylation state.

Polar overdominance An emergent epigenetic inheritance pattern in which there is an increase in DNA methylation in one cross direction of the offspring relative to their parents. Polar overdominance likely involves a combination of genomic imprinting and additional complex regulatory factors.

Polar underdominance An emergent epigenetic inheritance pattern in which there is a decrease in DNA methylation in one cross direction of the offspring relative to their parents. Polar underdominance likely involves a combination of genomic imprinting and additional complex regulatory factors.

Sex-specific DNA methylation DNA methylation on both alleles is driven by the sex of the individual.

Skewed X chromosome inactivation Preferential inactivation of one X chromosomal allele in heterozygous offspring.

***Trans*-acting** Factors which can exert their influence over sites located anywhere in the genome and on either allele.

***Trans*-acting meQTL** An meQTL which acts in trans. As each of the two alleles' meQTLs can influence methylation over the same CpG sites on either allele, this introduces competition between the genetic variants to establish their associated methylation pattern, leading to several distinct forms of *trans*-acting meQTLs.

Transvection The alteration of DNA methylation of an allele by its homolog, i.e. one dominant parental allele alters the methylation state of the other allele to match its own. Transvection events exhibit characteristics of both *cis*- and *trans*-acting mechanisms insofar as they can influence DNA methylation over CpG sites located on the opposite allele, however they can only do this at the homologous site in the genome and, as such, are bound to influence CpGs in close genomic proximity to the genetic variant. Methylation patterns in the F2 generation are determined by the zygosity of the sample over the local region containing the DMR.

Underdominance An emergent epigenetic inheritance pattern in which there is a decrease in DNA methylation on both alleles of the offspring relative to their parents.

Supplementary Data

Supplementary Data 1. F2 candidate region methylation. Liver methylation from the F2 generation over the dominant *trans*-acting meQTL/transvection/paramutation DMRs, *cis*-acting meQTL DMRs, non-dominant *trans*-acting meQTL DMRs, and sex-specific DMRs chosen for targeted analysis in the F2s. Bold lines represent coverage-weighted mean methylation of the respective group and CpG sites included in the final analysis are denoted by tick marks on the x-axis.

Supplementary Figures

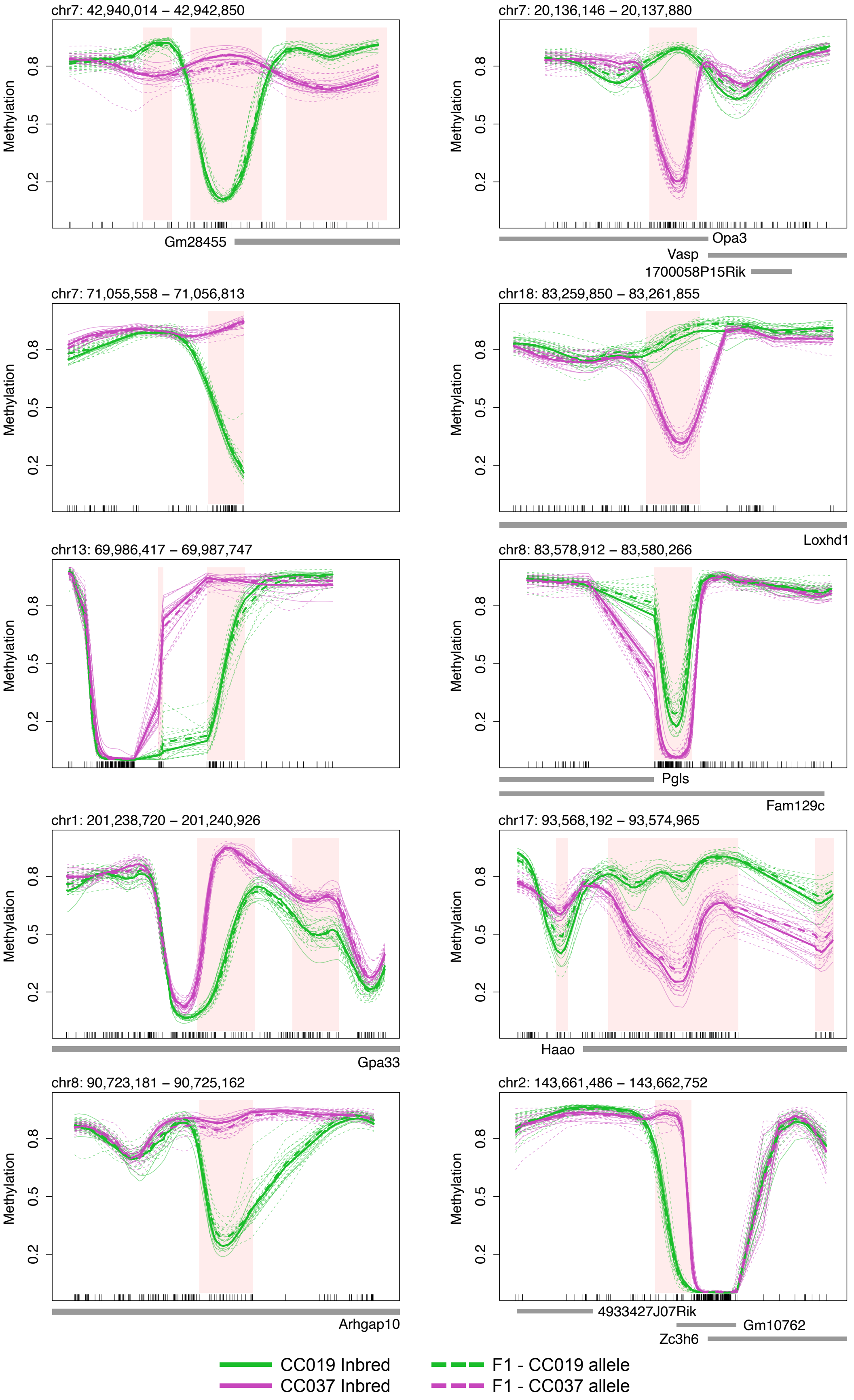

Supplementary Fig. 1. Top liver autosomal *cis*-acting meQTL DMRs. Top 10 liver autosomal DMRs categorized as *cis*-acting meQTLs. Bold lines represent coverage-weighted mean methylation of the respective group and CpG sites included in the final analysis are denoted by tick marks on the x-axis.

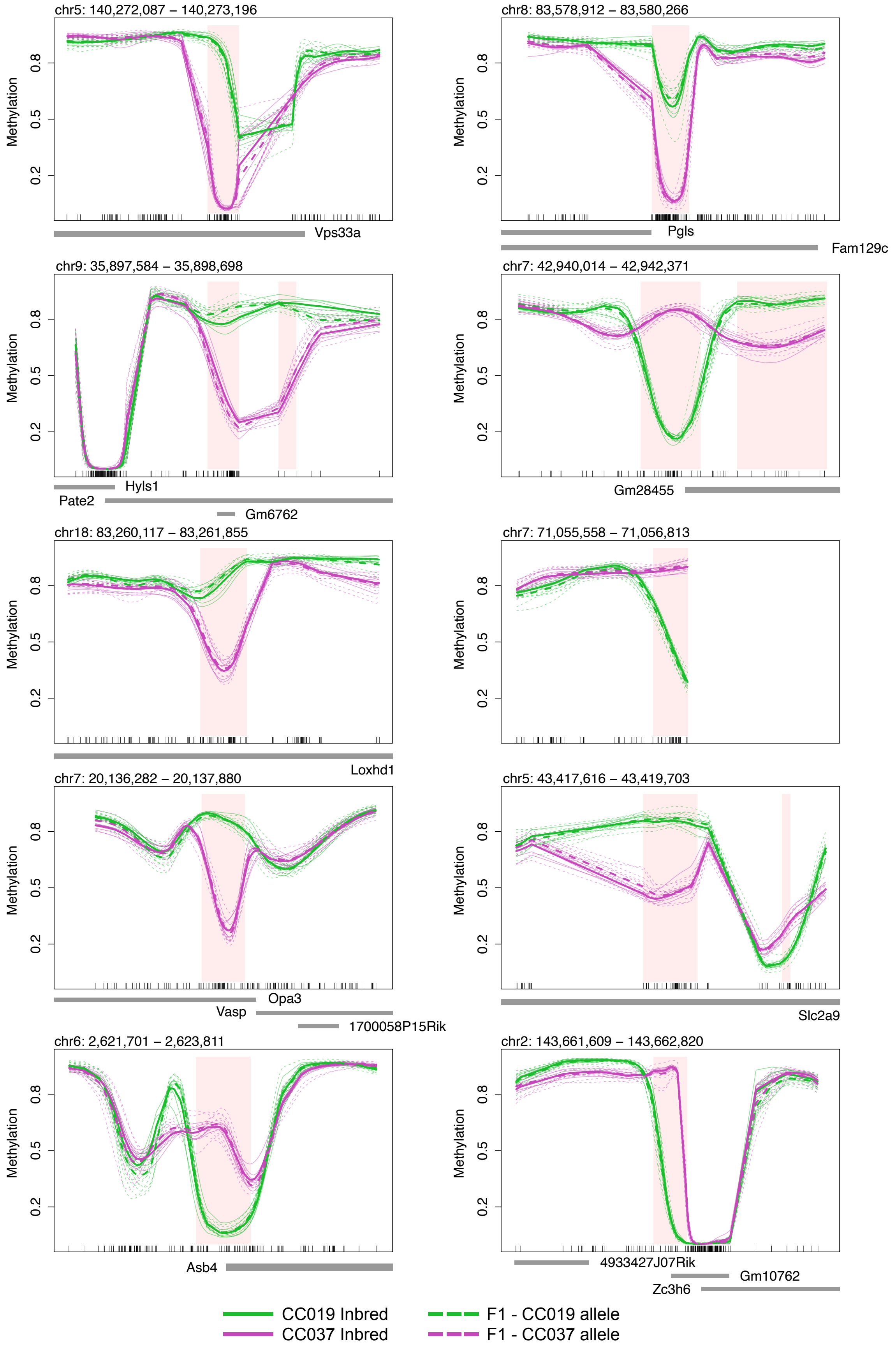

Supplementary Fig. 2. Top muscle autosomal *cis*-acting meQTL DMRs. Top 10 muscle autosomal DMRs categorized as *cis*-acting meQTLs. Bold lines represent coverage-weighted mean methylation of the respective group and CpG sites included in the final analysis are denoted by tick marks on the x-axis.

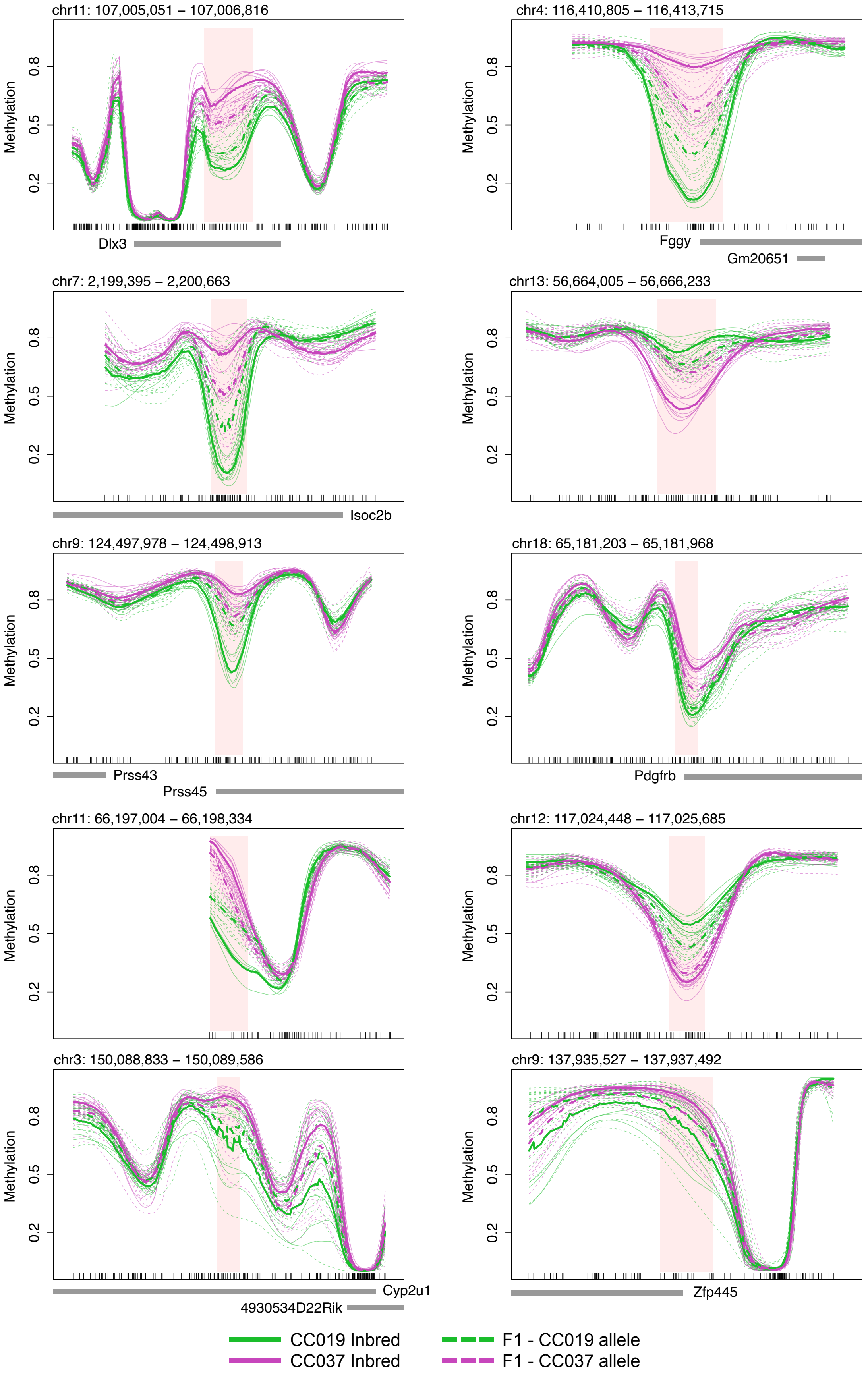

Supplementary Fig. 3. Top liver autosomal non-dominant *trans*-acting meQTL DMRs. Top 10 liver autosomal DMRs categorized as non-dominant *trans*-acting meQTLs. Bold lines represent coverage-weighted mean methylation of the respective group and CpG sites included in the final analysis are denoted by tick marks on the x-axis. Note that the regions overlapping *Cyp2u1* and *Zfp445* exhibit potential batch specificity (as defined in the Methods).

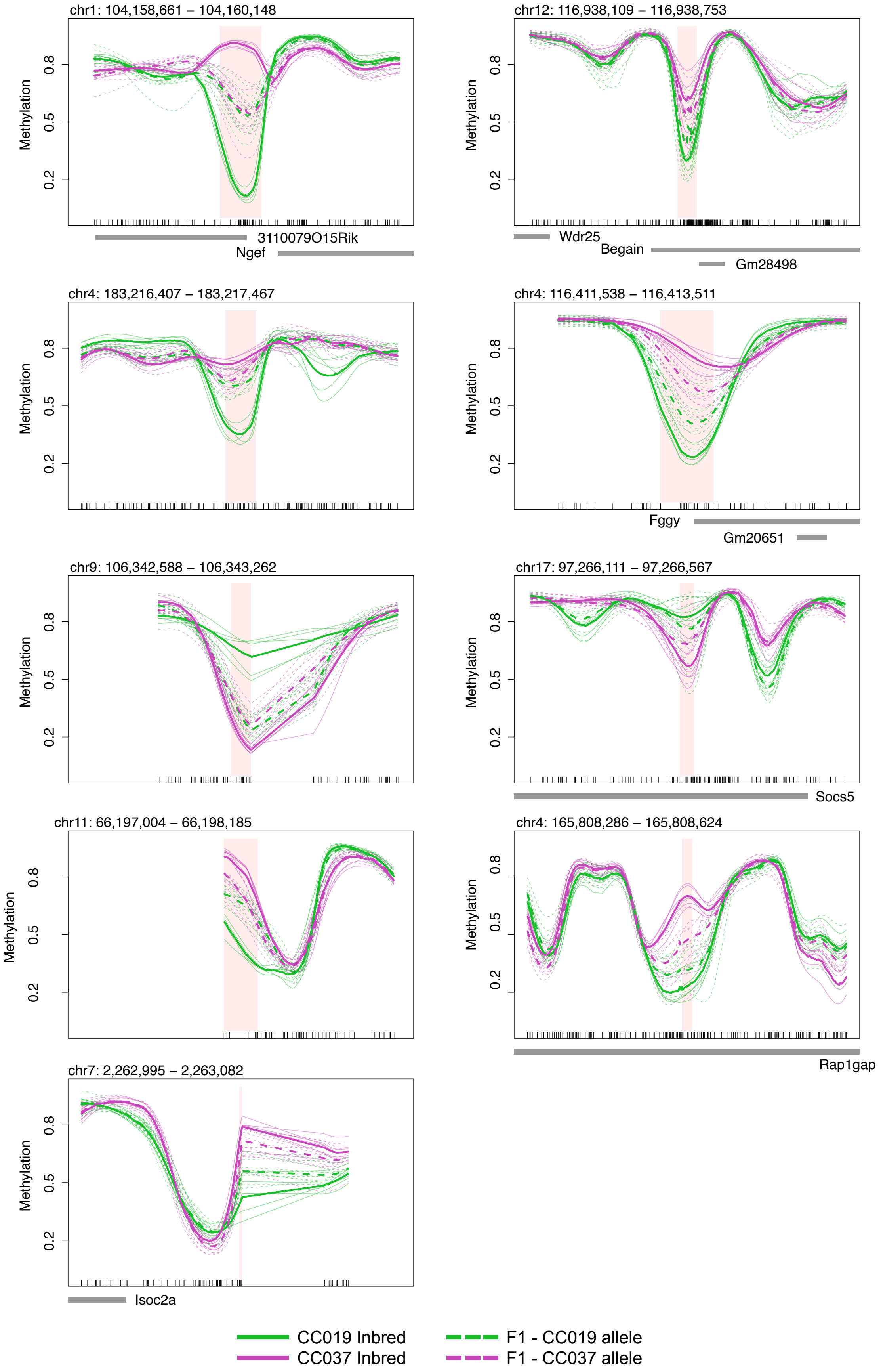

Supplementary Fig. 4. Muscle autosomal non-dominant *trans*-acting meQTL DMRs. Muscle autosomal DMRs categorized as non-dominant *trans*-acting meQTLs. Bold lines represent coverage-weighted mean methylation of the respective group and CpG sites included in the final analysis are denoted by tick marks on the x-axis.

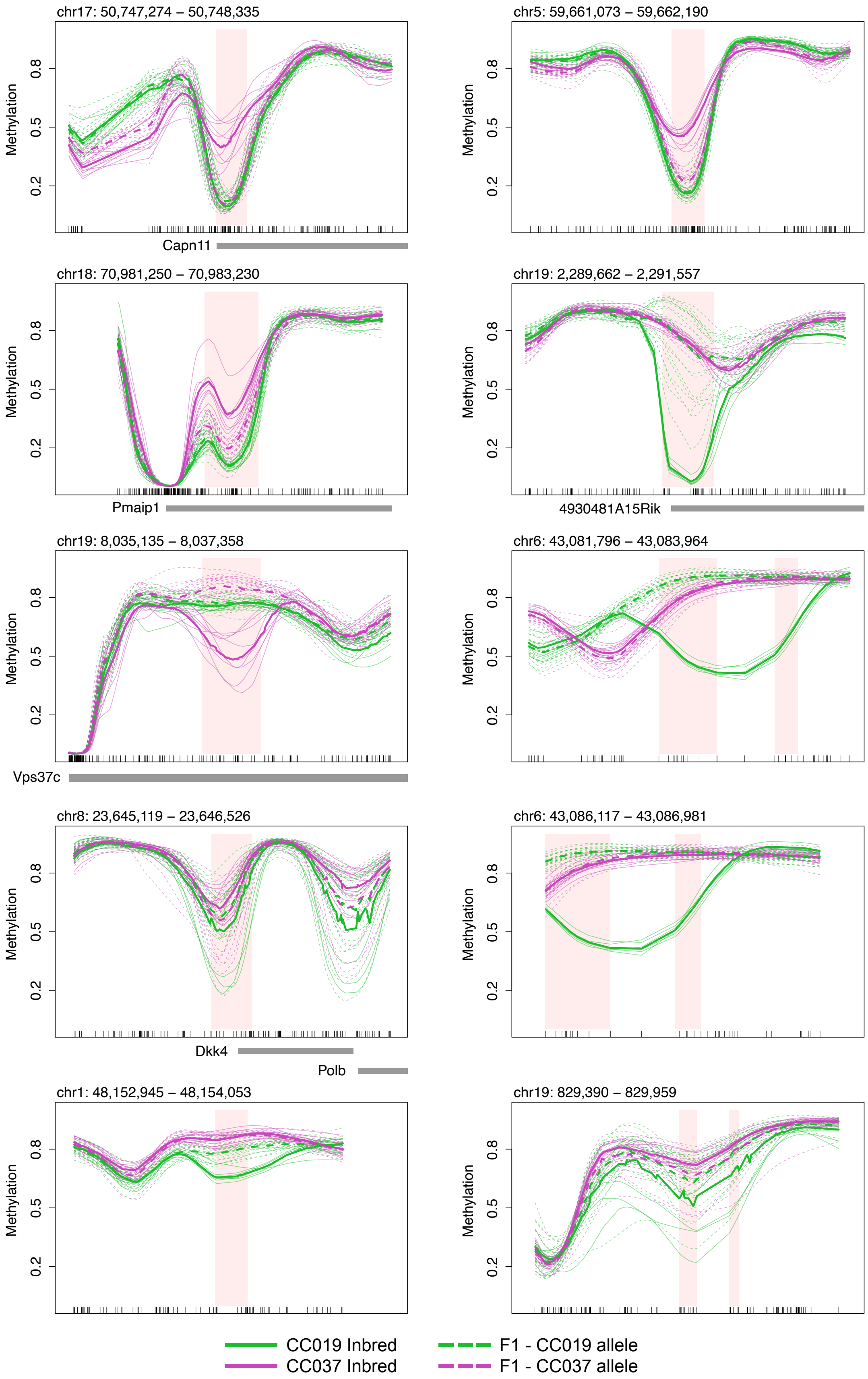

Supplementary Fig. 5. Top liver autosomal dominant *trans*-acting meQTL, transvection, and paramutation DMRs. Top 10 liver autosomal DMRs categorized as dominant *trans*-acting meQTLs, transvection, or paramutation. Bold lines represent coverage-weighted mean methylation of the respective group and CpG sites included in the final analysis are denoted by tick marks on the x-axis. Note that the region overlapping *Dkk4* and the final region (chr19: 829,390-829,959) exhibit potential batch specificity (as defined in the Methods).

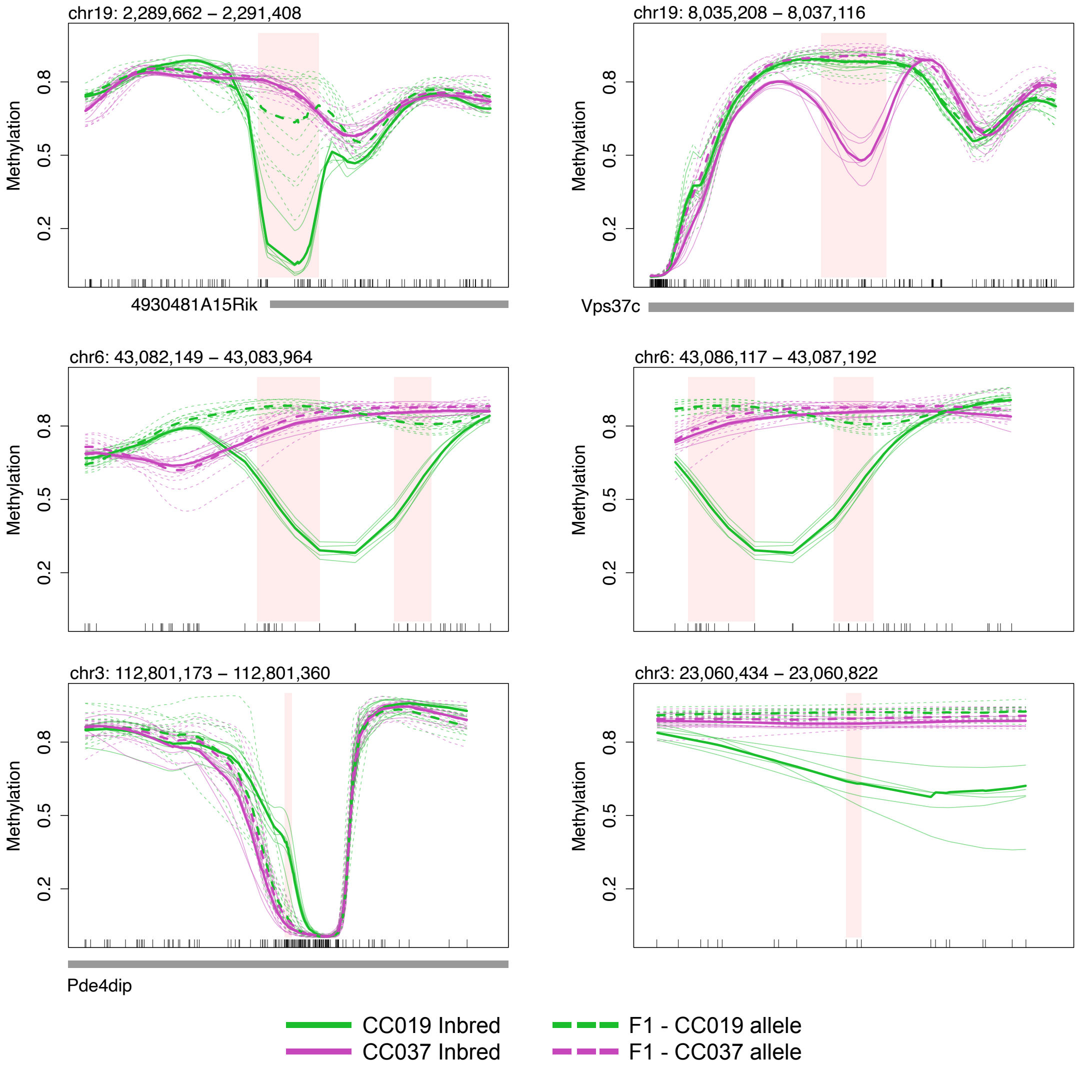

Supplementary Fig. 6. Muscle autosomal dominant *trans*-acting meQTL, transvection, and paramutation DMRs. Muscle autosomal DMRs categorized as dominant *trans*-acting meQTLs, transvection, or paramutation. Bold lines represent coverage-weighted mean methylation of the respective group and CpG sites included in the final analysis are denoted by tick marks on the x-axis.

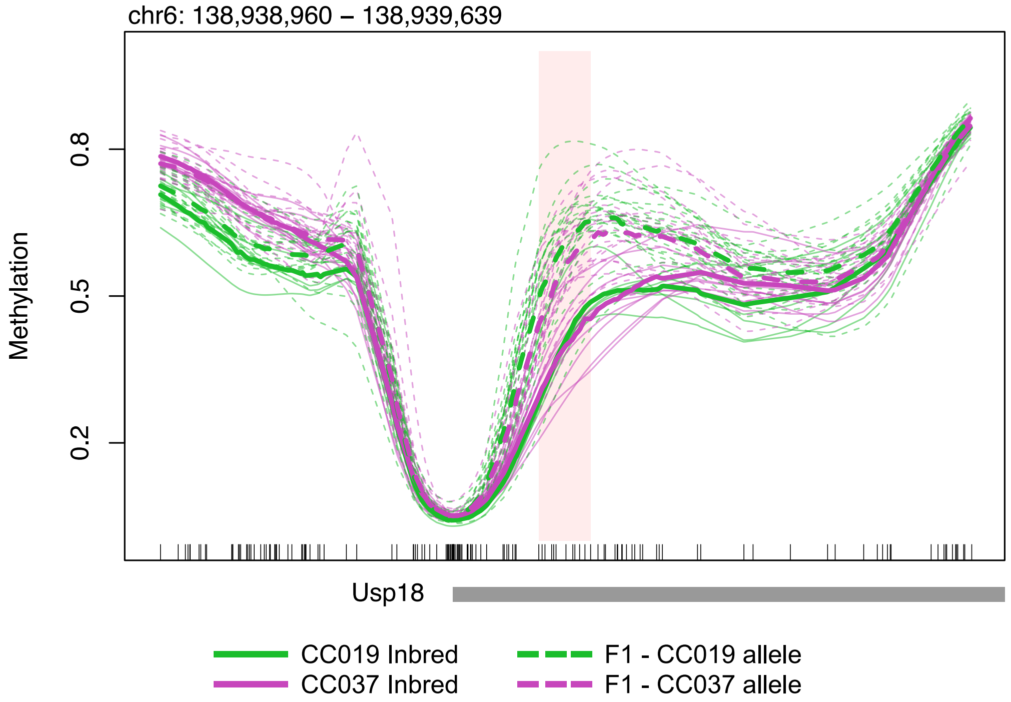

Supplementary Fig. 7. Liver autosomal overdominance and underdominance DMRs. Liver autosomal DMRs categorized as overdominance or underdominance. Bold lines represent coverage-weighted mean methylation of the respective group and CpG sites included in the final analysis are denoted by tick marks on the x-axis.

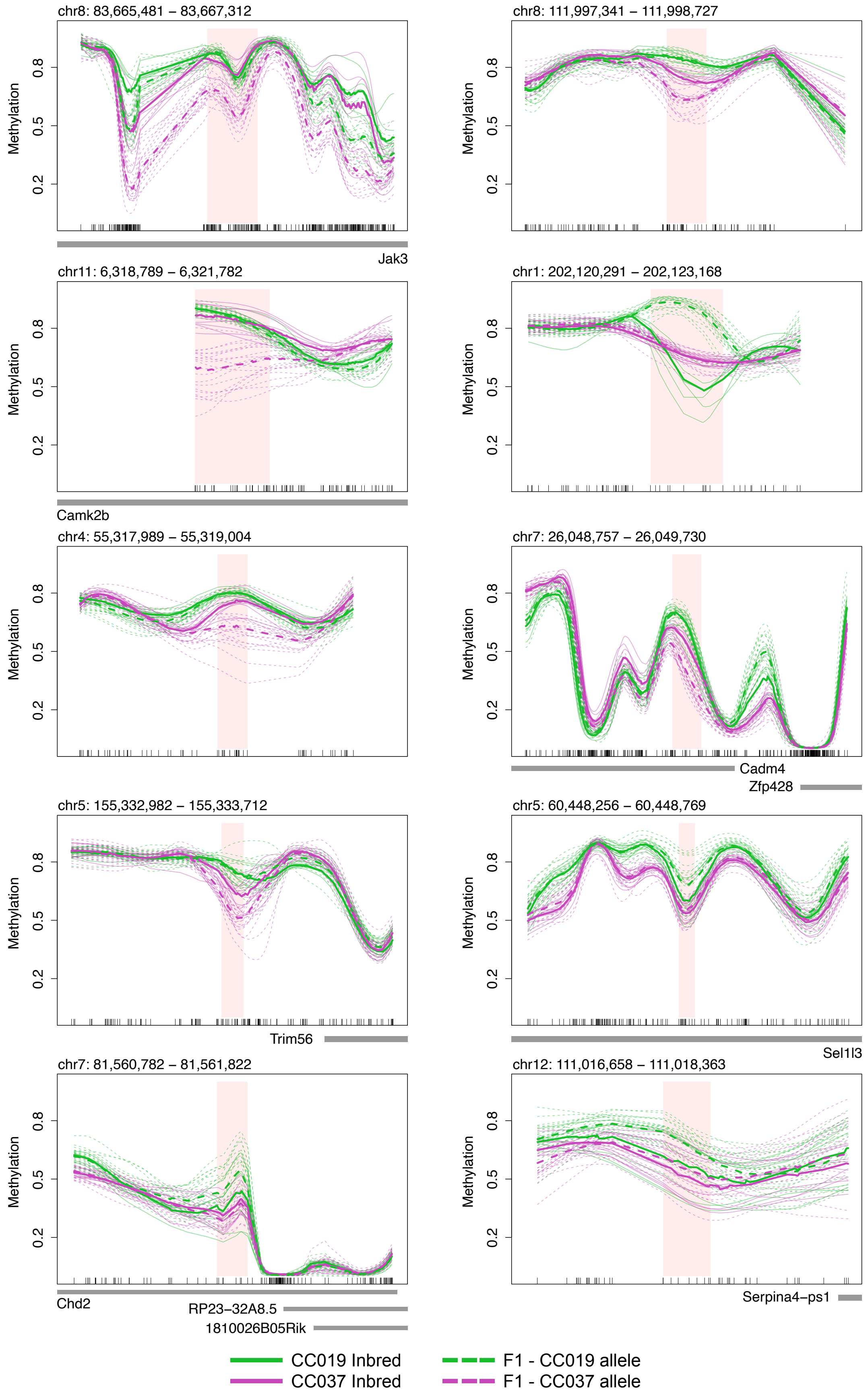

Supplementary Fig. 8. Top liver autosomal allele-specific overdominance and allele-specific underdominance DMRs. Top 10 liver autosomal DMRs categorized as allele-specific overdominance or allele-specific underdominance. Bold lines represent coverage-weighted mean methylation of the respective group and CpG sites included in the final analysis are denoted by tick marks on the x-axis.

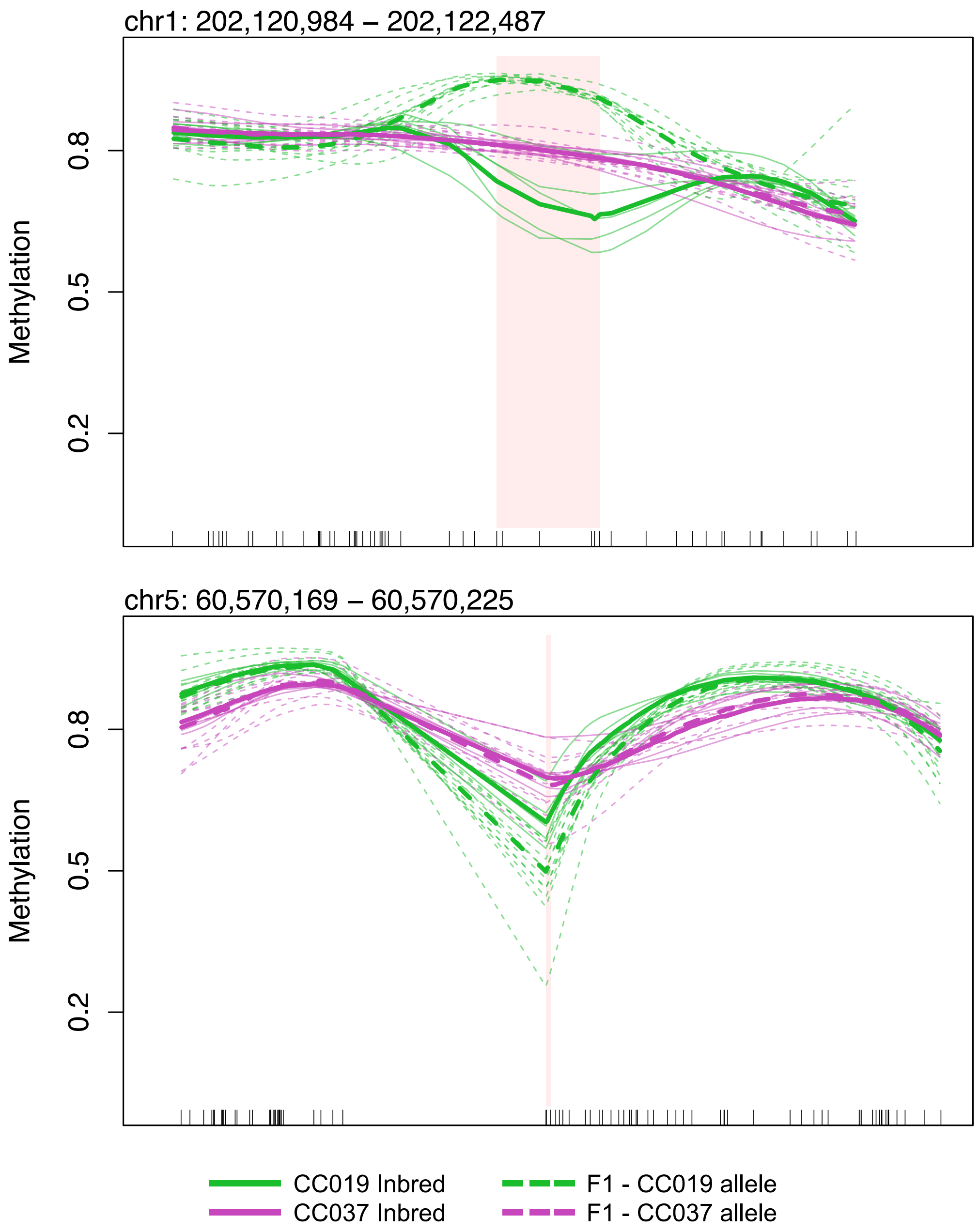

Supplementary Fig. 9. Muscle autosomal allele-specific overdominance and allele-specific underdominance DMRs. Muscle autosomal DMRs categorized as allele-specific overdominance or allele-specific underdominance. Bold lines represent coverage-weighted mean methylation of the respective group and CpG sites included in the final analysis are denoted by tick marks on the x-axis.

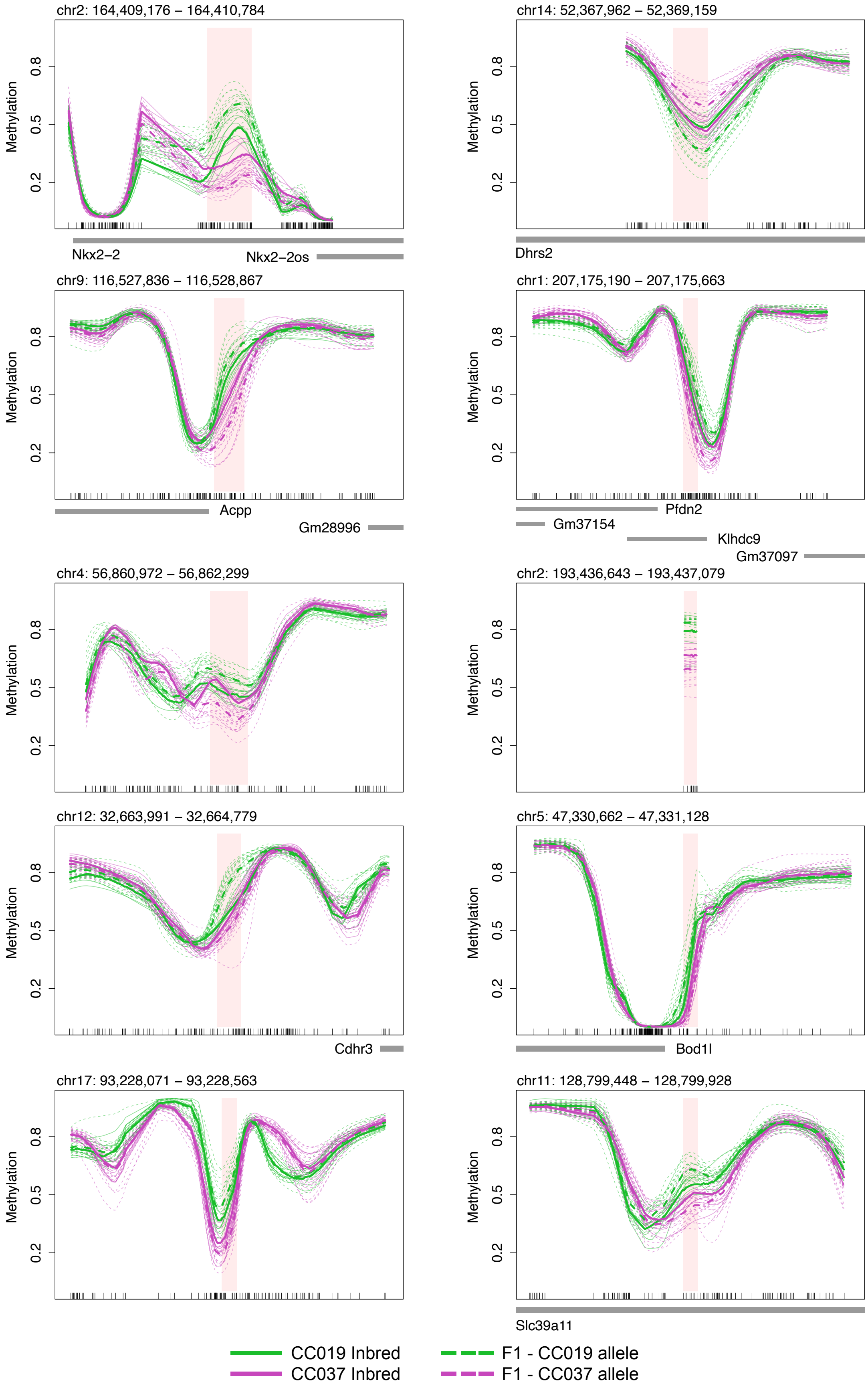

Supplementary Fig. 10. Top liver autosomal biallelic dominance DMRs. Top 10 liver autosomal DMRs categorized as biallelic dominance. Bold lines represent coverage-weighted mean methylation of the respective group and CpG sites included in the final analysis are denoted by tick marks on the x-axis.

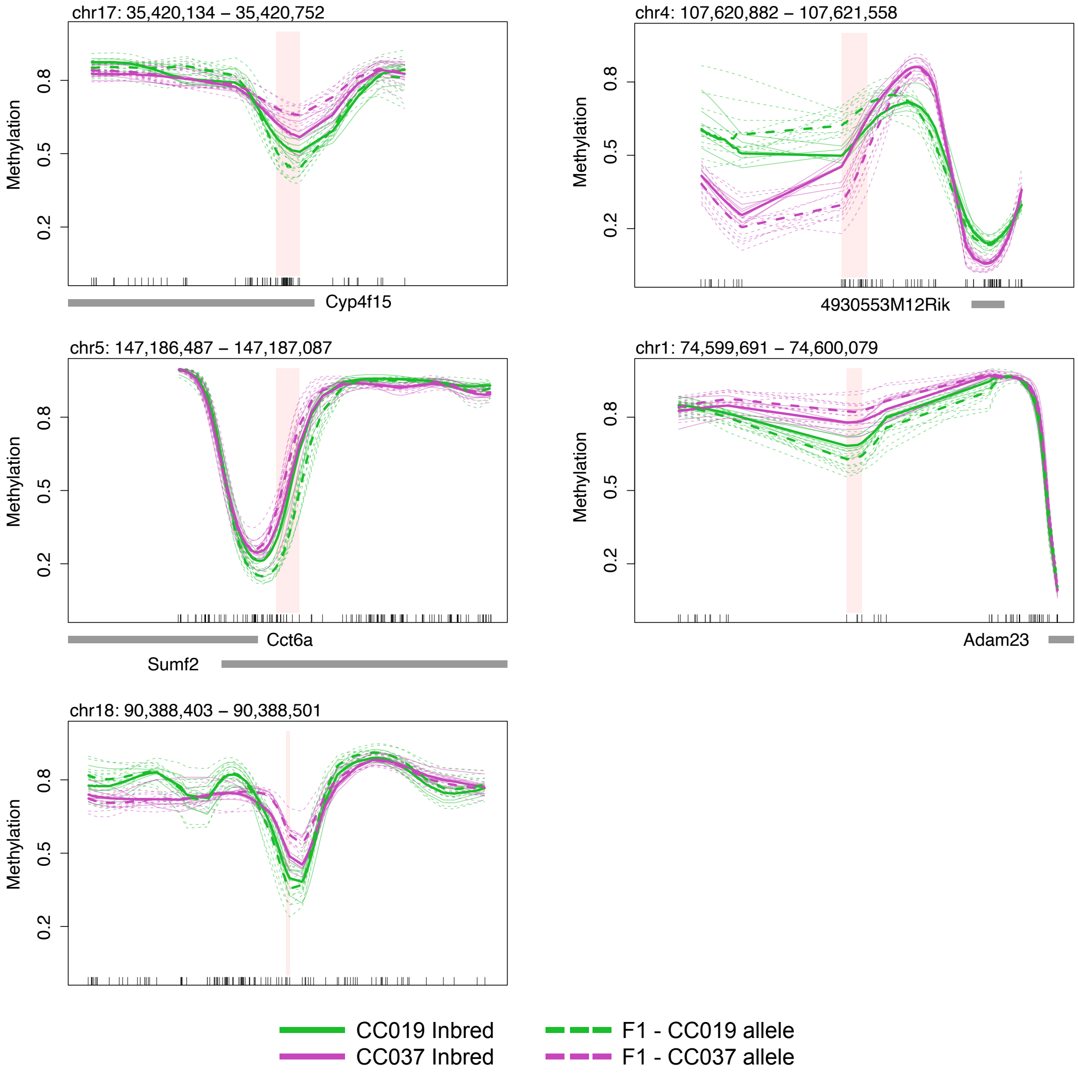

Supplementary Fig. 11. Muscle autosomal biallelic dominance DMRs. Muscle autosomal DMRs categorized as biallelic dominance. Bold lines represent coverage-weighted mean methylation of the respective group and CpG sites included in the final analysis are denoted by tick marks on the x-axis.

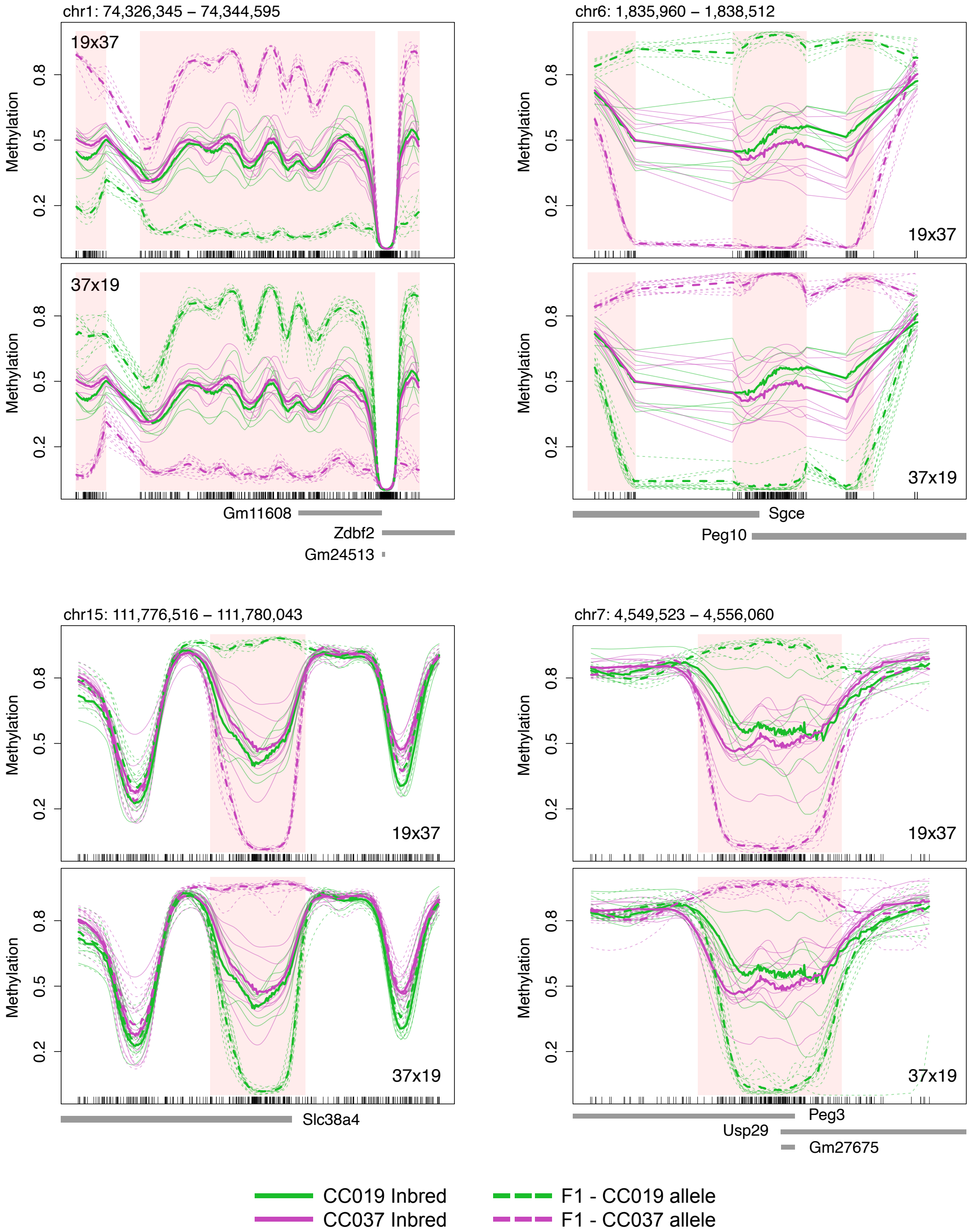

Supplementary Fig. 12. Top liver autosomal imprinted DMRs. Top 10 liver autosomal DMRs categorized as genomic imprinting from the inbred samples and 19x37 F1s (top) and inbred samples and 37x19 F1s (bottom). Bold lines represent coverage-weighted mean methylation of the respective group and CpG sites included in the final analysis are denoted by tick marks on the x-axis.

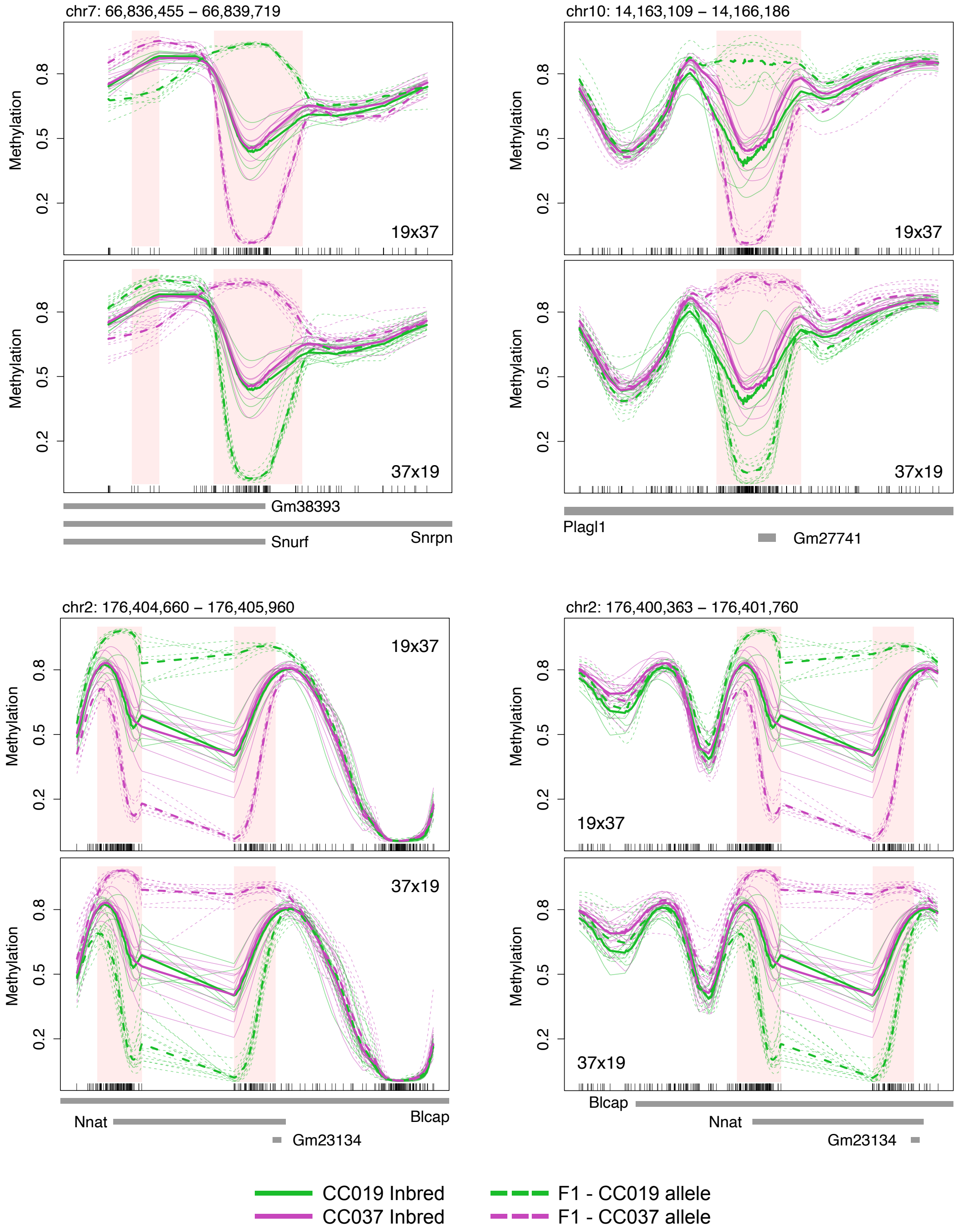

Supplementary Fig. 12. Continuation from previous page.

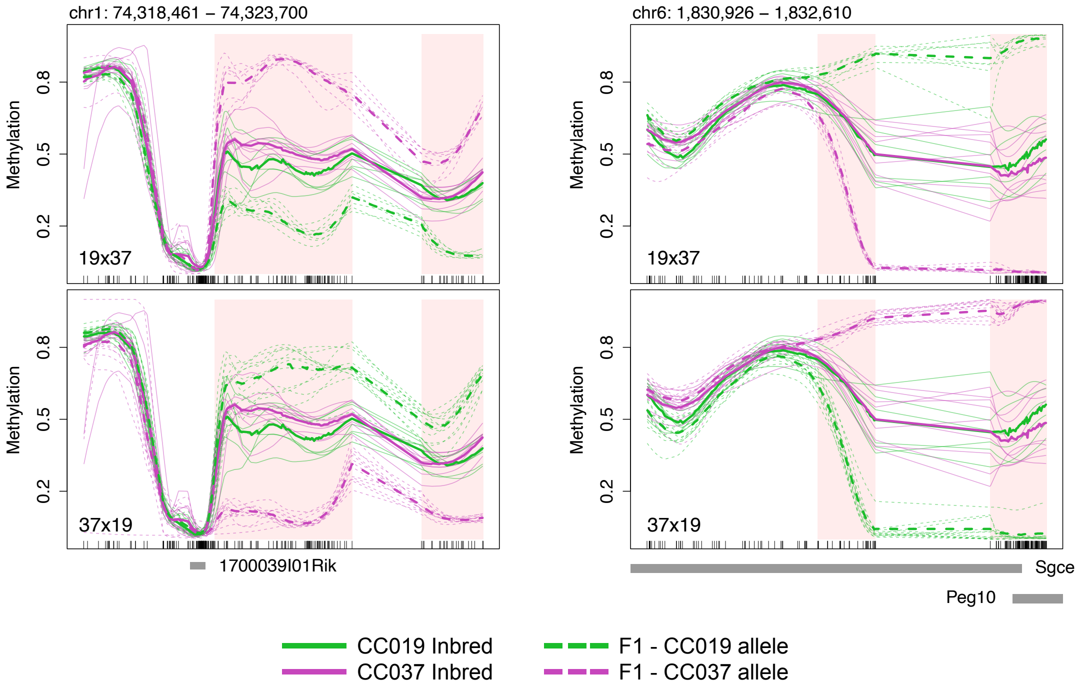

Supplementary Fig. 12. Continuation from previous page.

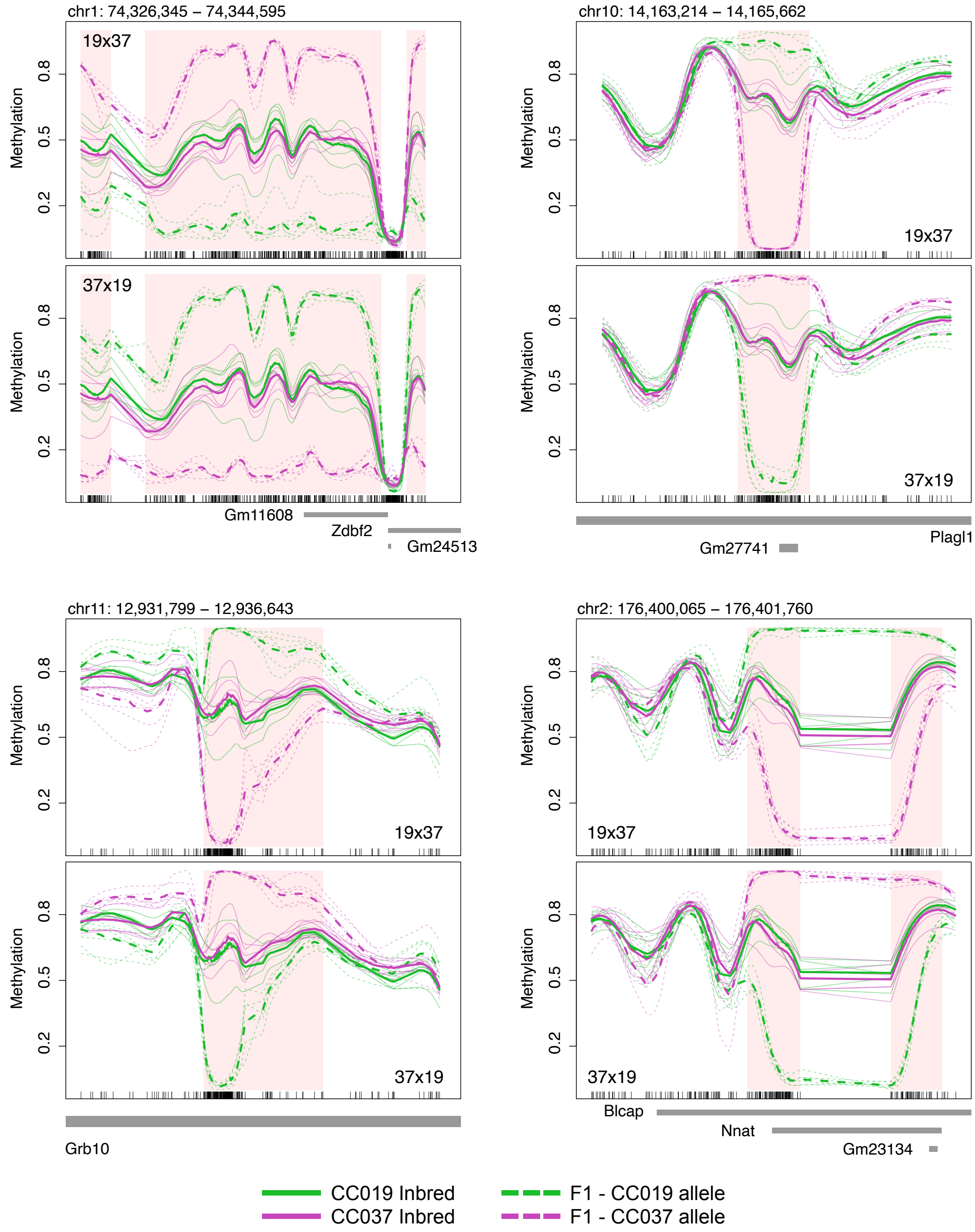

Supplementary Fig. 13. Top muscle autosomal imprinted DMRs. Top 10 muscle autosomal DMRs categorized as genomic imprinting from the inbred samples and 19x37 F1s (top) and inbred samples and 37x19 F1s (bottom). Bold lines represent coverage-weighted mean methylation of the respective group and CpG sites included in the final analysis are denoted by tick marks on the x-axis.

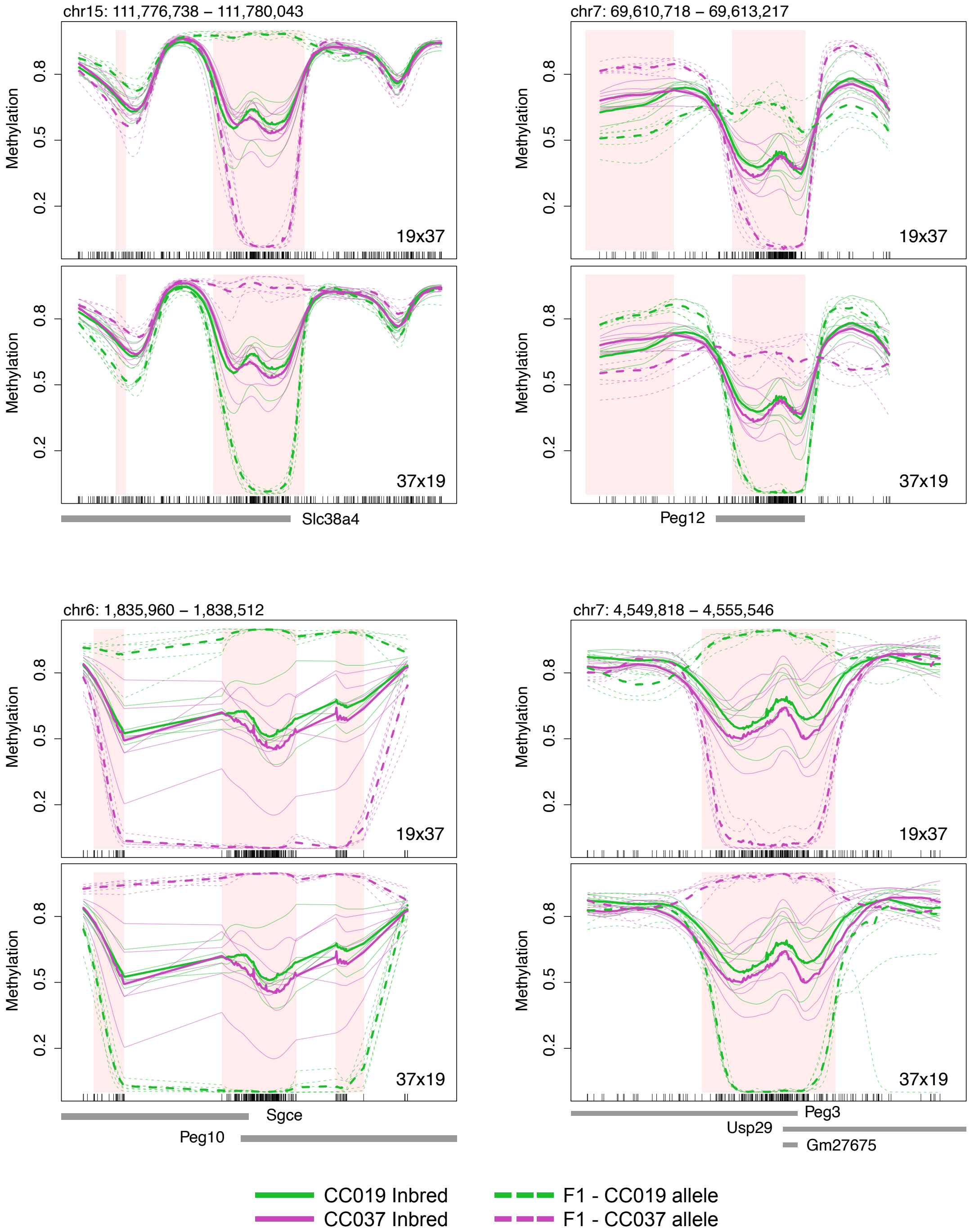

Supplementary Fig. 13. Continuation from previous page.

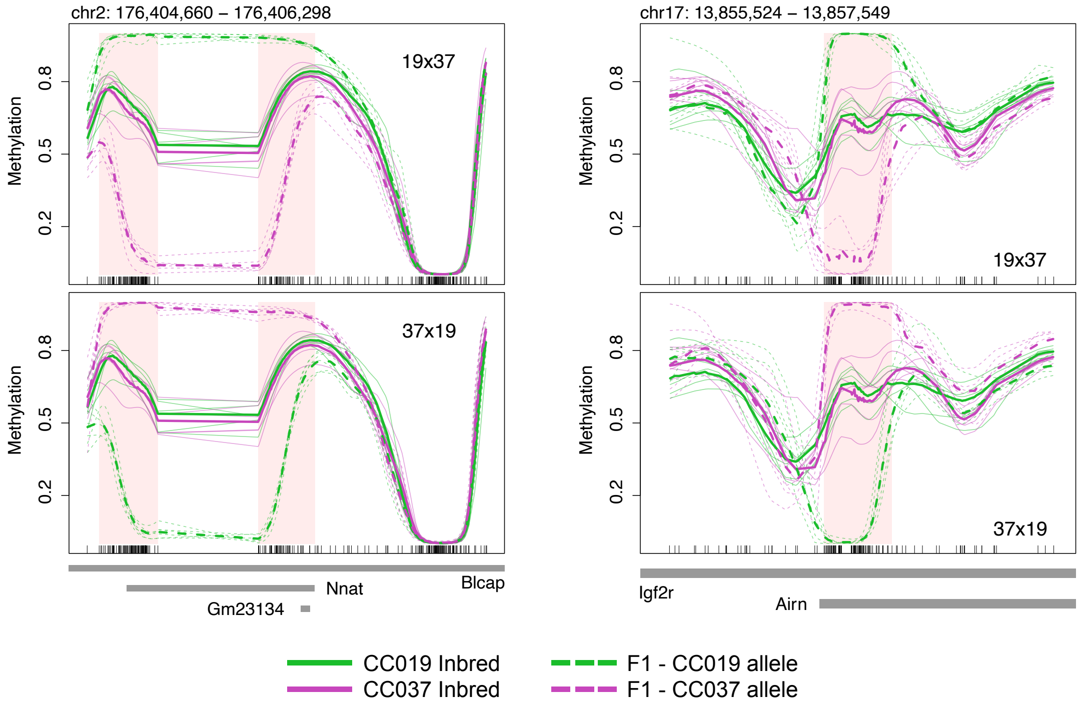

Supplementary Fig. 13. Continuation from previous page.

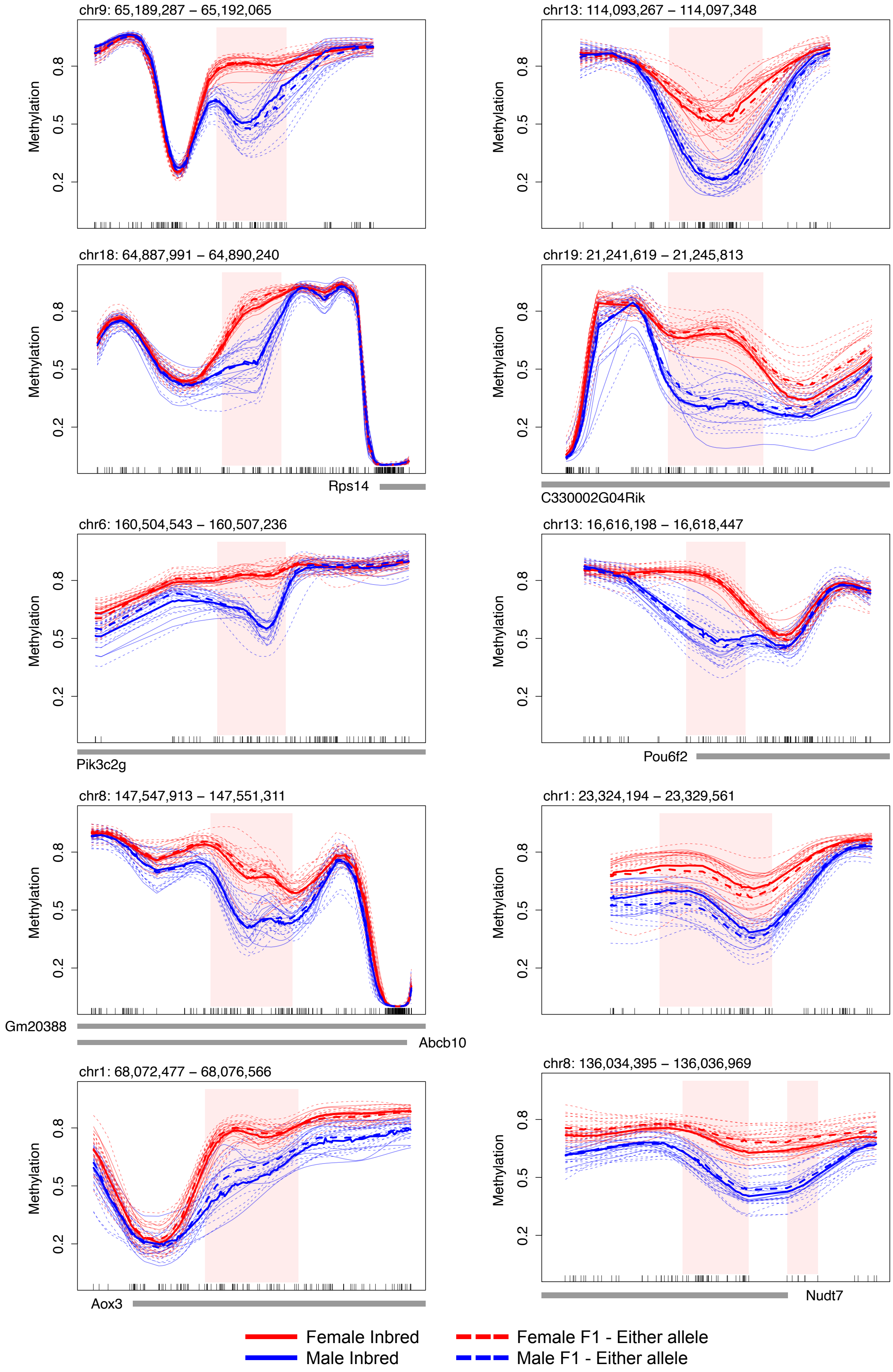

Supplementary Fig. 14. Top liver autosomal sex-specific DMRs. Top 10 liver autosomal DMRs categorized as sex-specific. Bold lines represent coverage-weighted mean methylation of the respective group and CpG sites included in the final analysis are denoted by tick marks on the x-axis.

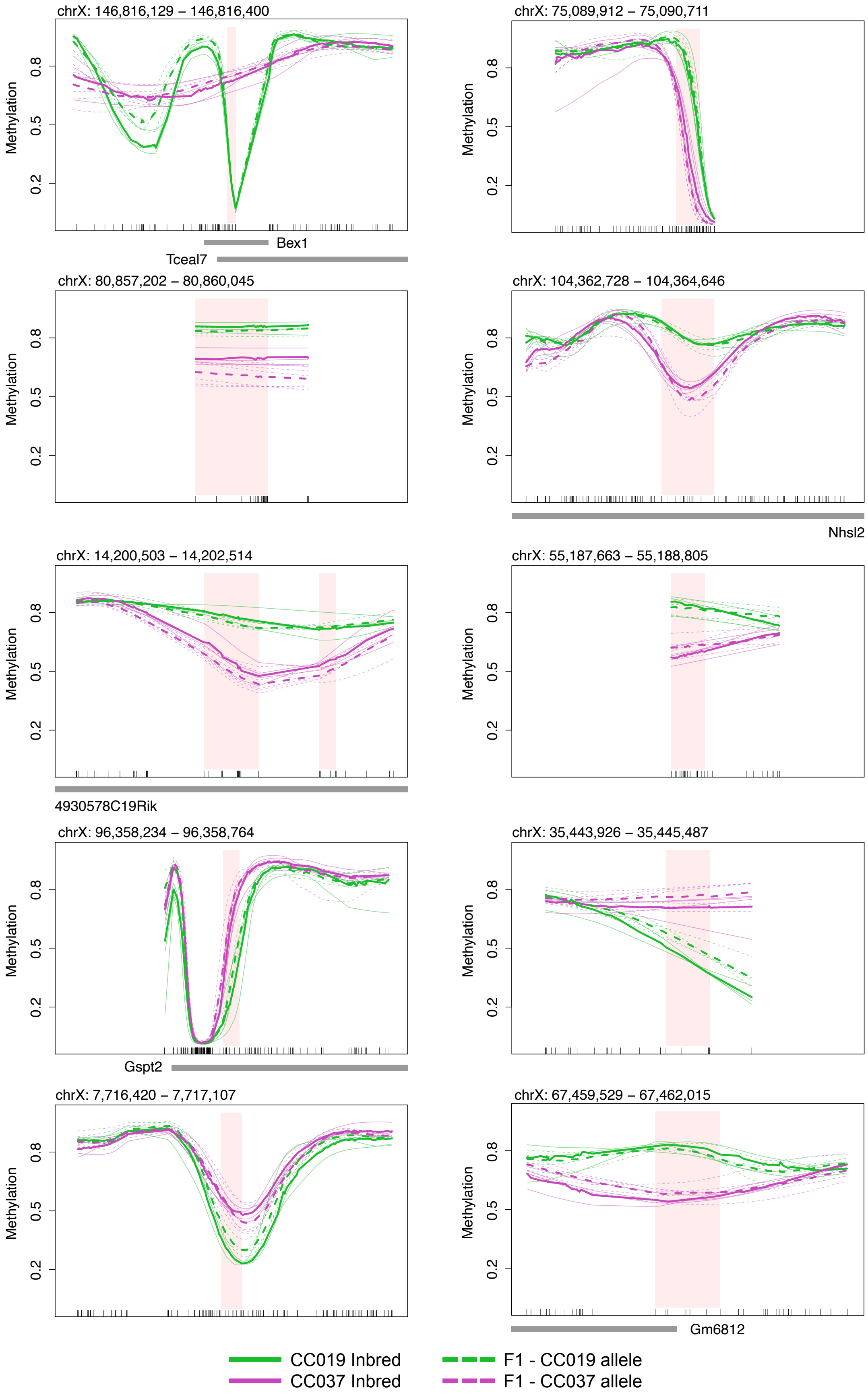

Supplementary Fig. 15. Top liver male maternal X chromosome *cis*-acting meQTL DMRs. Top 10 X chromosomal DMRs from the male maternal allele comparison in liver categorized as *cis*-acting meQTLs. Bold lines represent coverage-weighted mean methylation of the respective group and CpG sites included in the final analysis are denoted by tick marks on the x-axis.

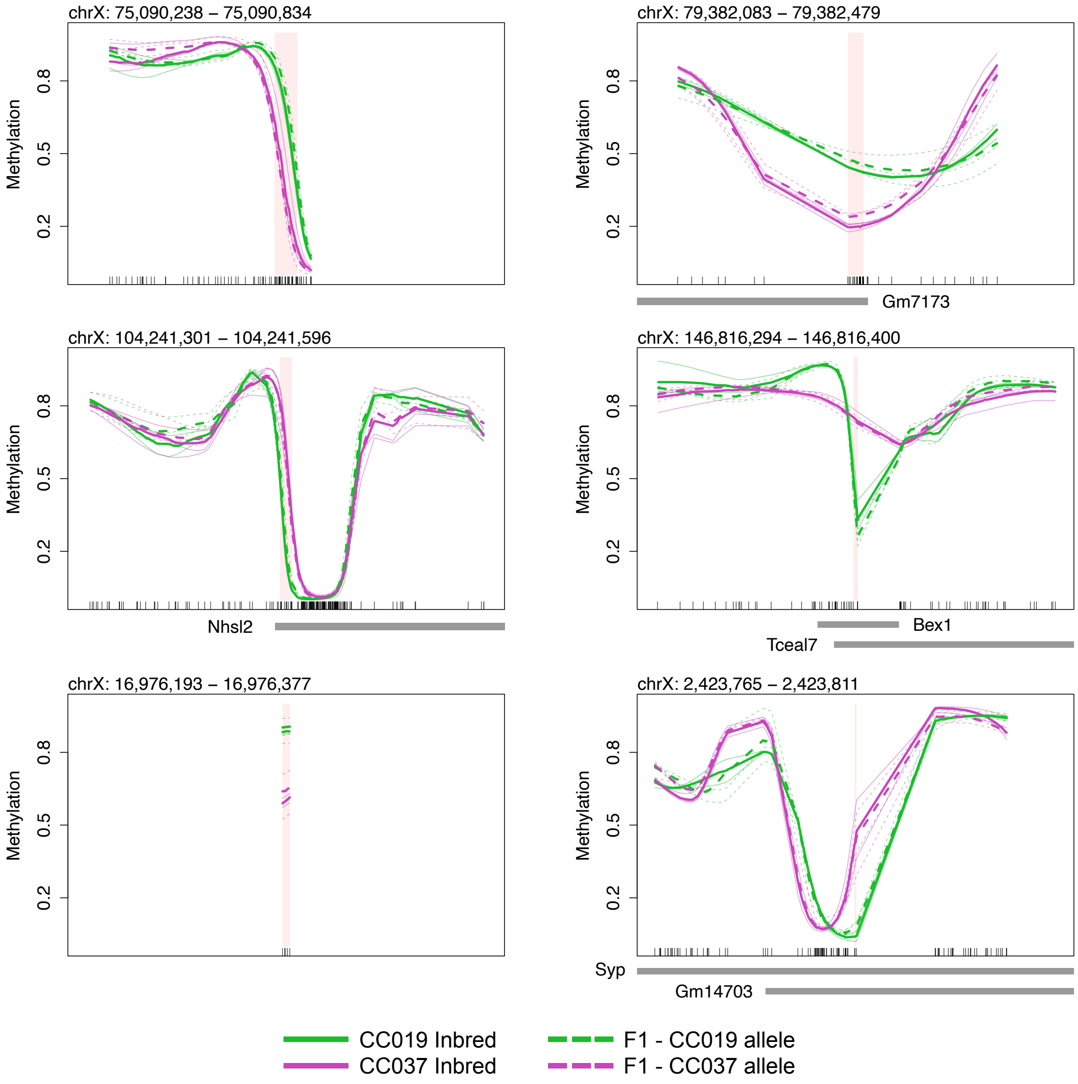

Supplementary Fig. 16. Muscle male maternal X chromosome *cis*-acting meQTL DMRs. X chromosomal DMRs from the male maternal allele comparison in muscle categorized as *cis*-acting meQTLs. Bold lines represent coverage-weighted mean methylation of the respective group and CpG sites included in the final analysis are denoted by tick marks on the x-axis.

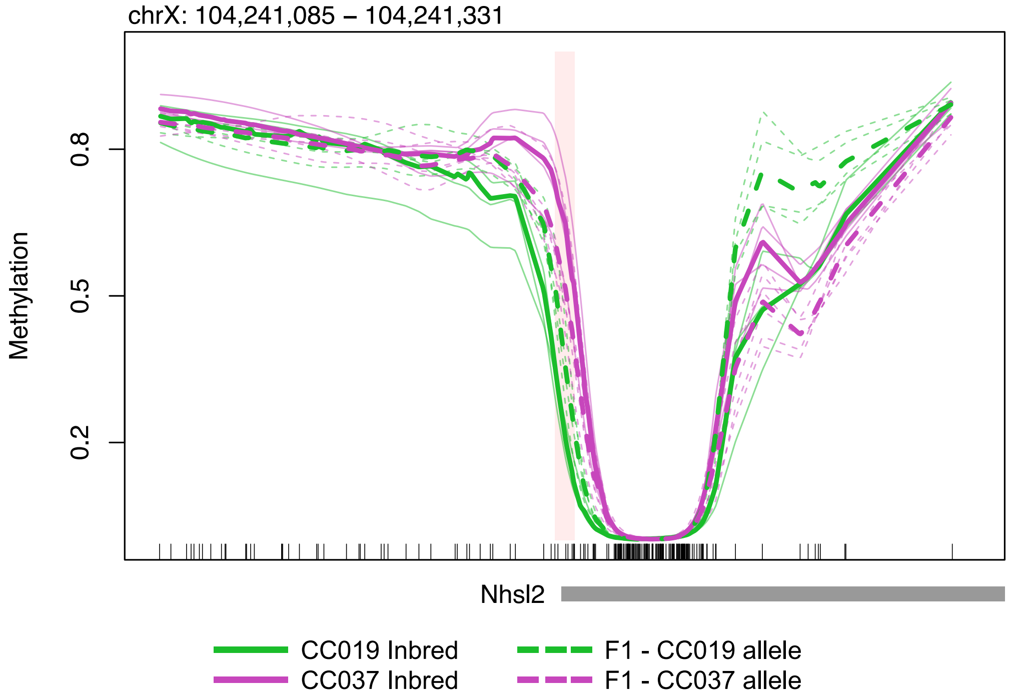

Supplementary Fig. 17. Liver male maternal X chromosome non-dominant *trans*-acting meQTL DMR. X chromosomal DMR from the male maternal allele comparison in liver categorized as non-dominant *trans*-acting meQTLs. Bold lines represent coverage-weighted mean methylation of the respective group and CpG sites included in the final analysis are denoted by tick marks on the x-axis.

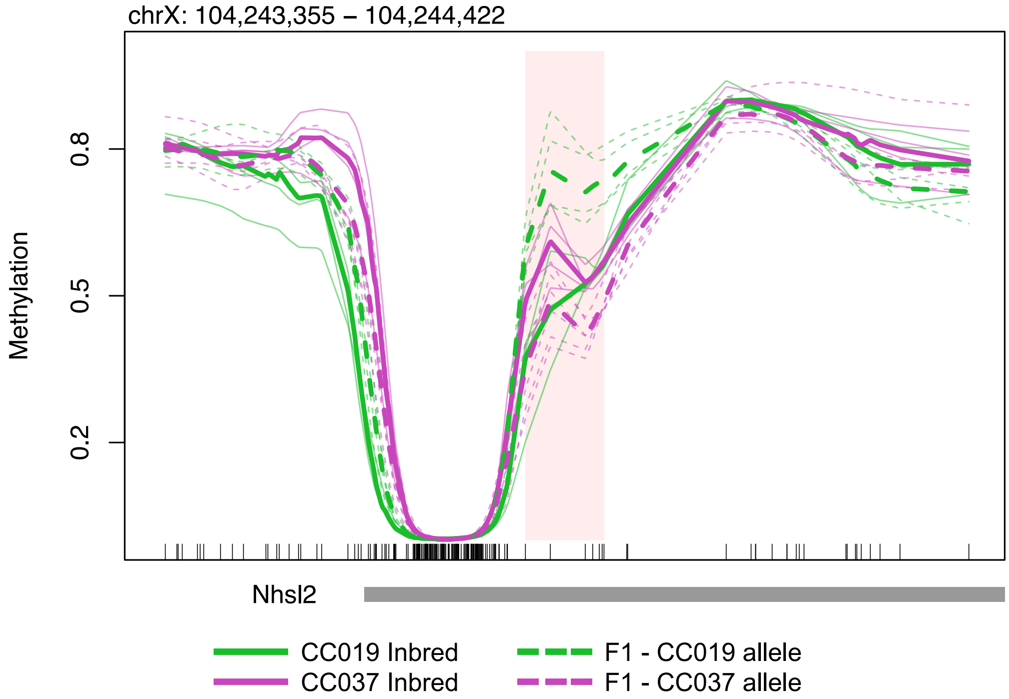

Supplementary Fig. 18. Liver male maternal X chromosome biallelic dominance DMR. X chromosomal DMR from the male maternal allele comparison in liver categorized as biallelic dominance. Bold lines represent coverage-weighted mean methylation of the respective group and CpG sites included in the final analysis are denoted by tick marks on the x-axis.

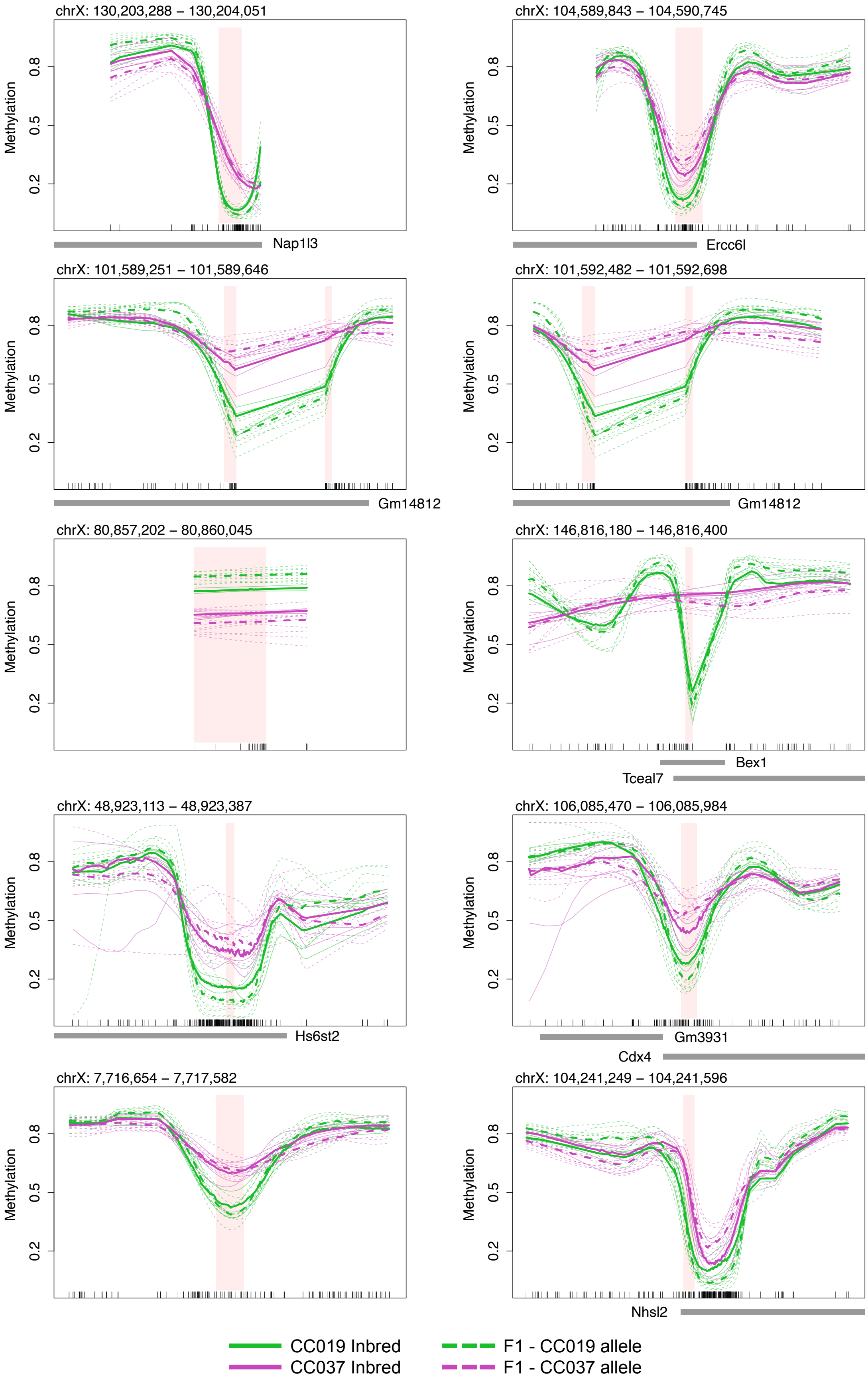

Supplementary Fig. 19. Top liver female X chromosome *cis*-acting meQTL DMRs. Top 10 X chromosomal DMRs from the female comparison in liver categorized as *cis*-acting meQTLs. Bold lines represent coverage-weighted mean methylation of the respective group and CpG sites included in the final analysis are denoted by tick marks on the x-axis.

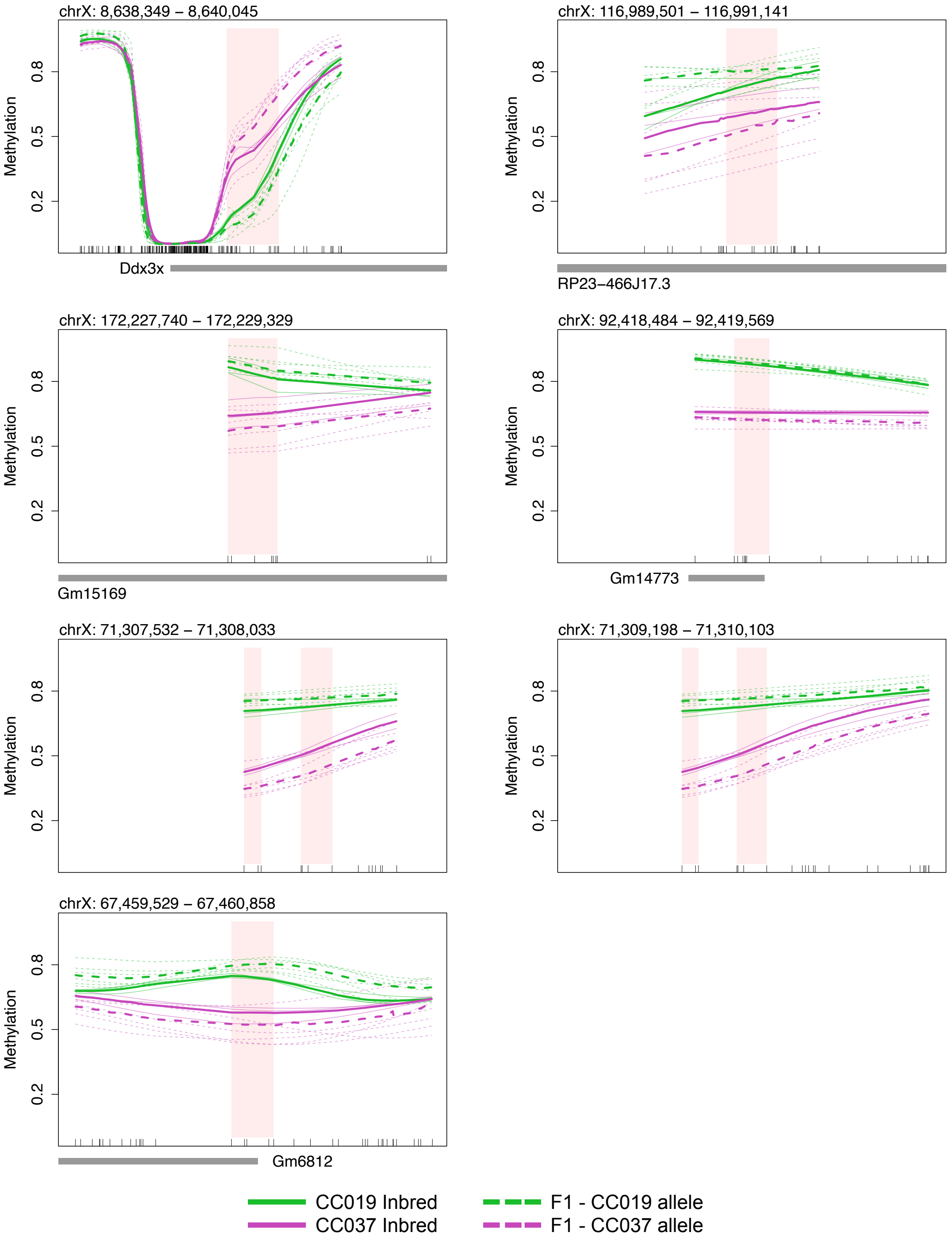

Supplementary Fig. 20. Muscle female X chromosome *cis*-acting meQTL DMRs. X chromosomal DMRs from the female comparison in muscle categorized as *cis*-acting meQTLs. Bold lines represent coverage-weighted mean methylation of the respective group and CpG sites included in the final analysis are denoted by tick marks on the x-axis.

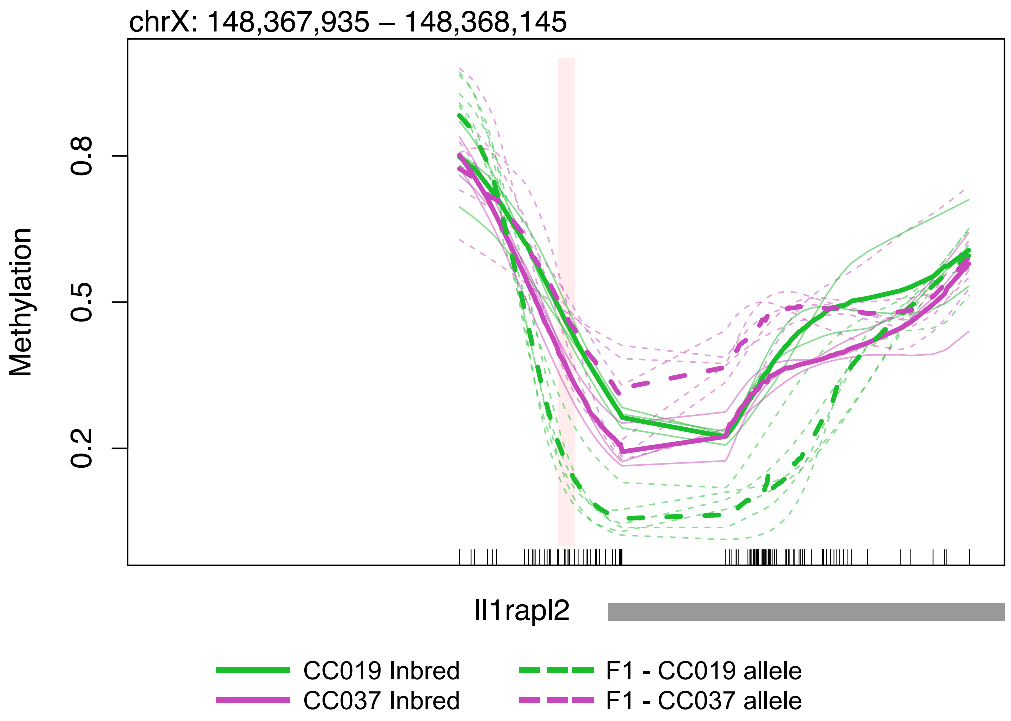

Supplementary Fig. 21. Muscle female X chromosome allele-specific underdominance DMR. X chromosomal DMR from the female comparison in muscle categorized as allele-specific overdominance or allele-specific underdominance. Bold lines represent coverage-weighted mean methylation of the respective group and CpG sites included in the final analysis are denoted by tick marks on the x-axis.

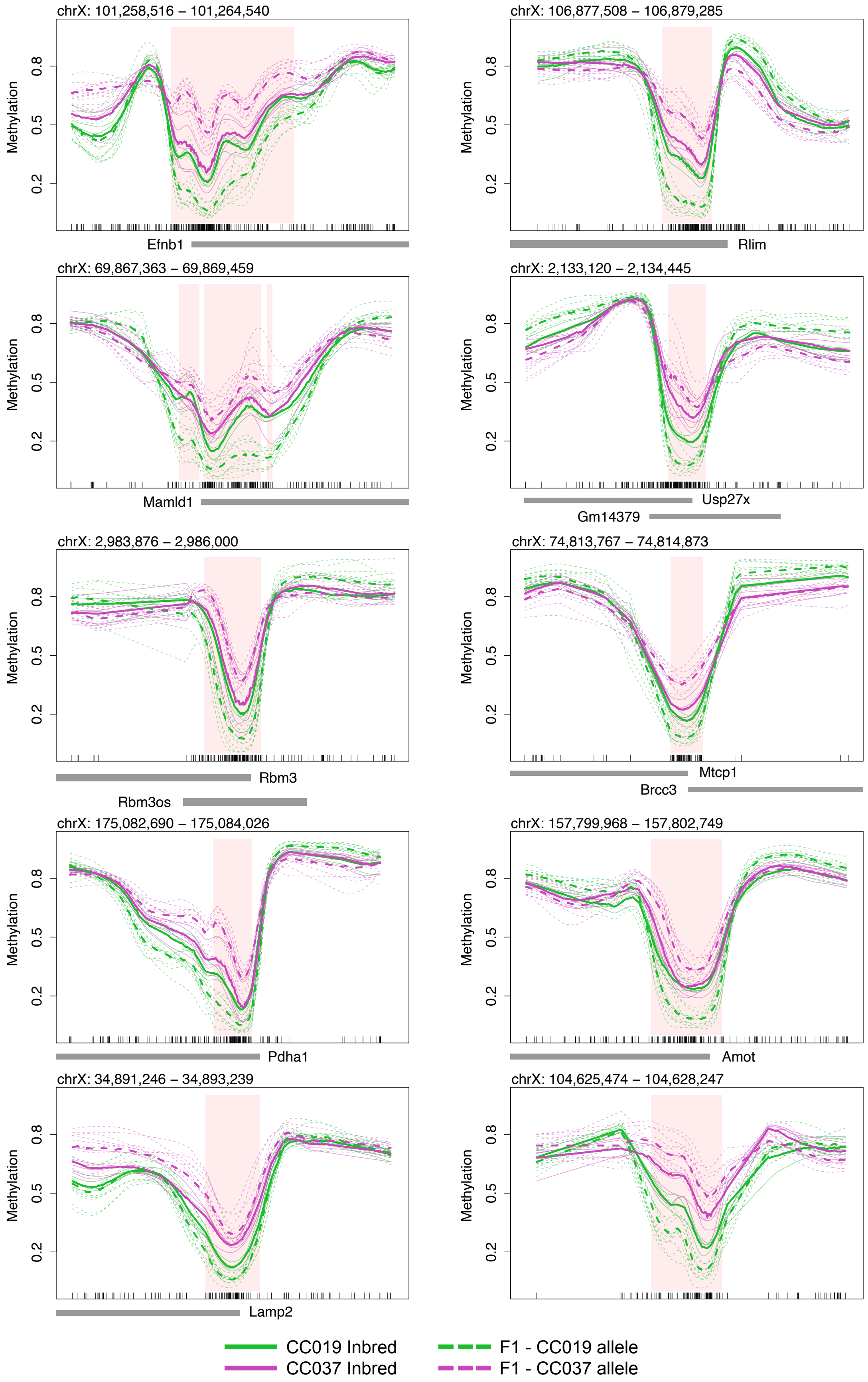

Supplementary Fig. 22. Top liver female X chromosome skewed XCI DMRs. Top 10 X chromosomal DMRs from the female comparison in liver categorized as skewed XCI. Bold lines represent coverage-weighted mean methylation of the respective group and CpG sites included in the final analysis are denoted by tick marks on the x-axis.

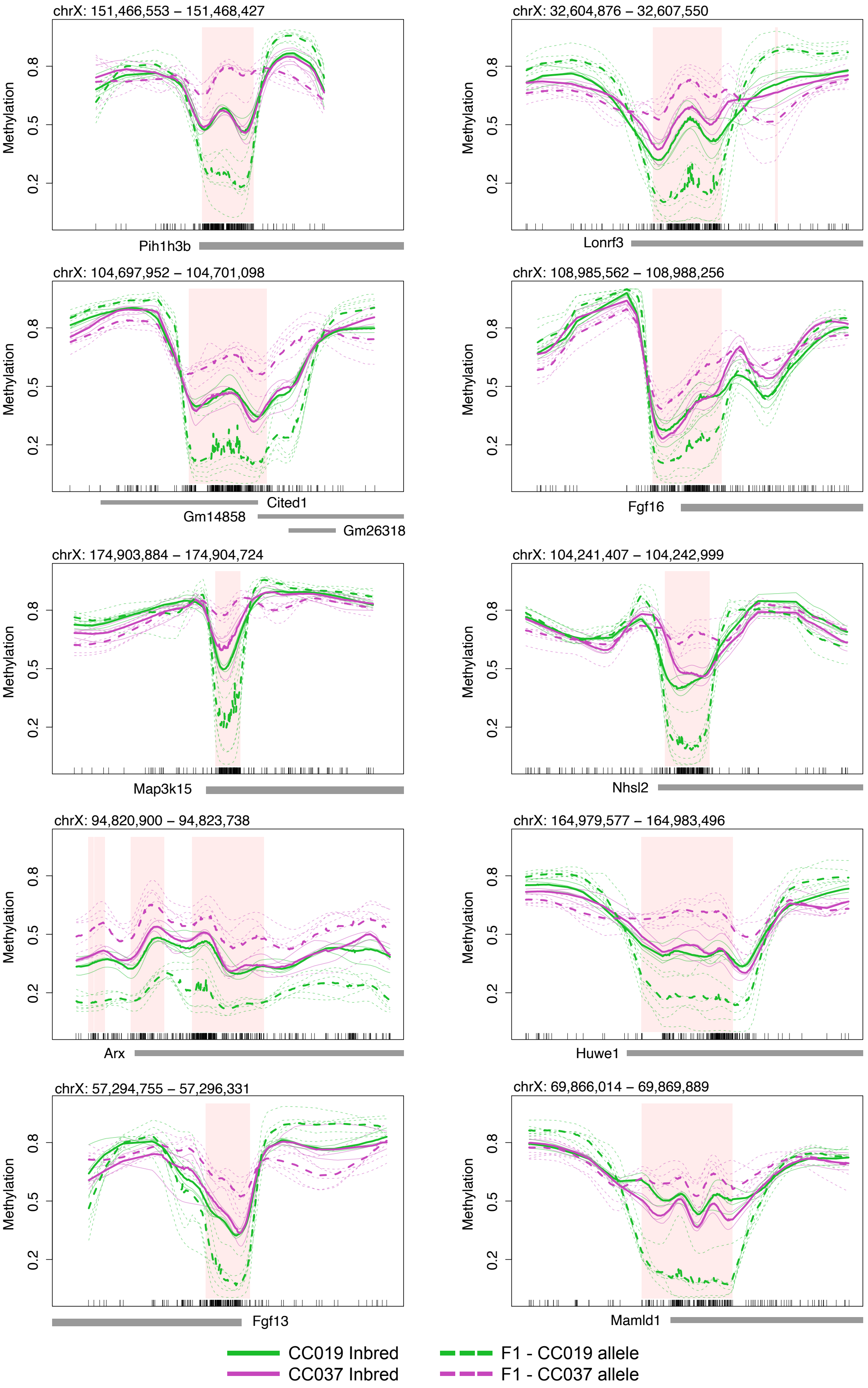

Supplementary Fig. 23. Top muscle female X chromosome skewed XCI DMRs. Top 10 X chromosomal DMRs from the female comparison in muscle categorized as skewed XCI. Bold lines represent coverage-weighted mean methylation of the respective group and CpG sites included in the final analysis are denoted by tick marks on the x-axis.

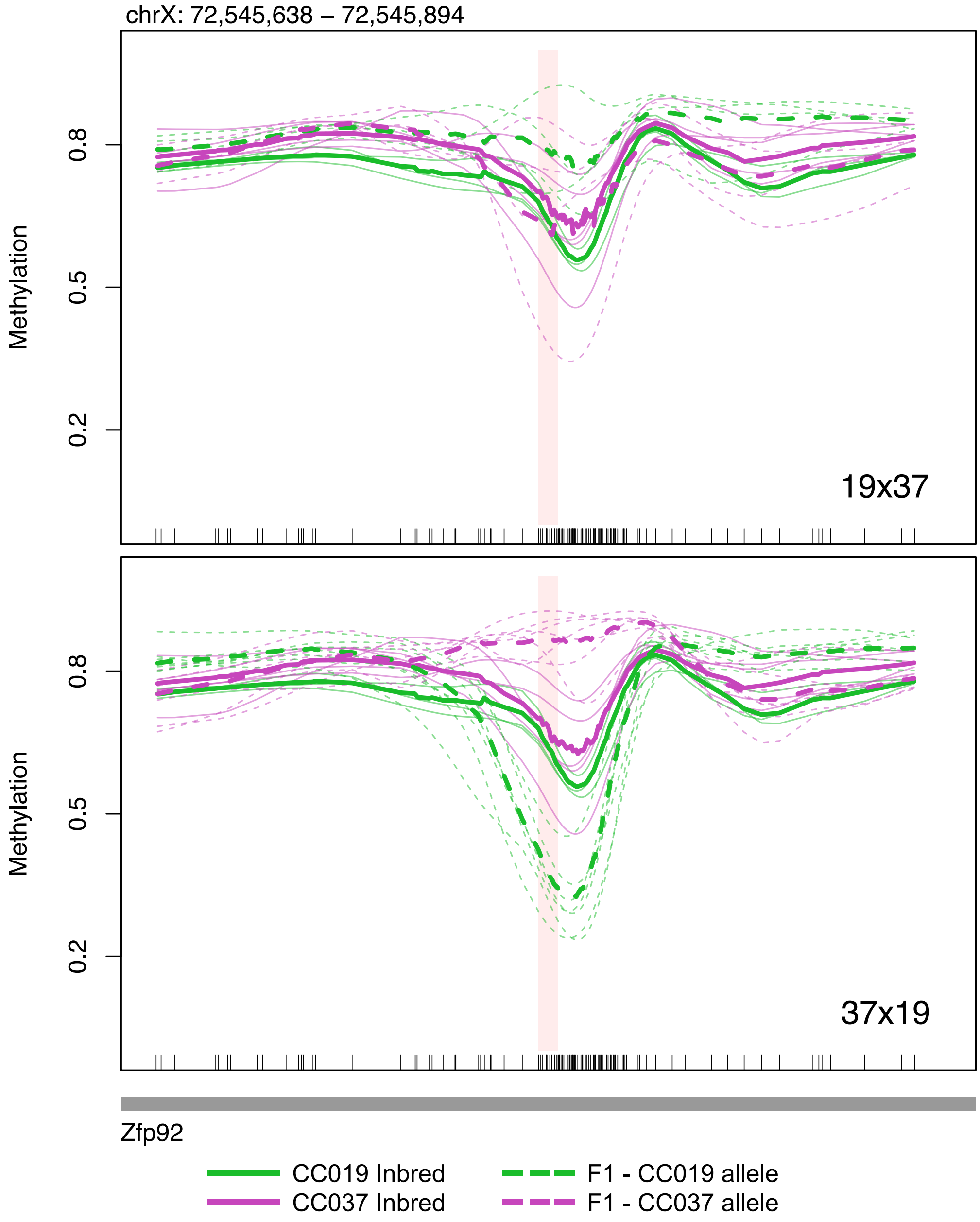

Supplementary Fig. 24. Liver female X chromosome imprinted DMR. X chromosomal DMR from the female comparison in liver categorized as genomic imprinting from the inbred samples and 19x37 F1s (top) and inbred samples and 37x19 F1s (bottom). Bold lines represent coverage-weighted mean methylation of the respective group and CpG sites included in the final analysis are denoted by tick marks on the x-axis.

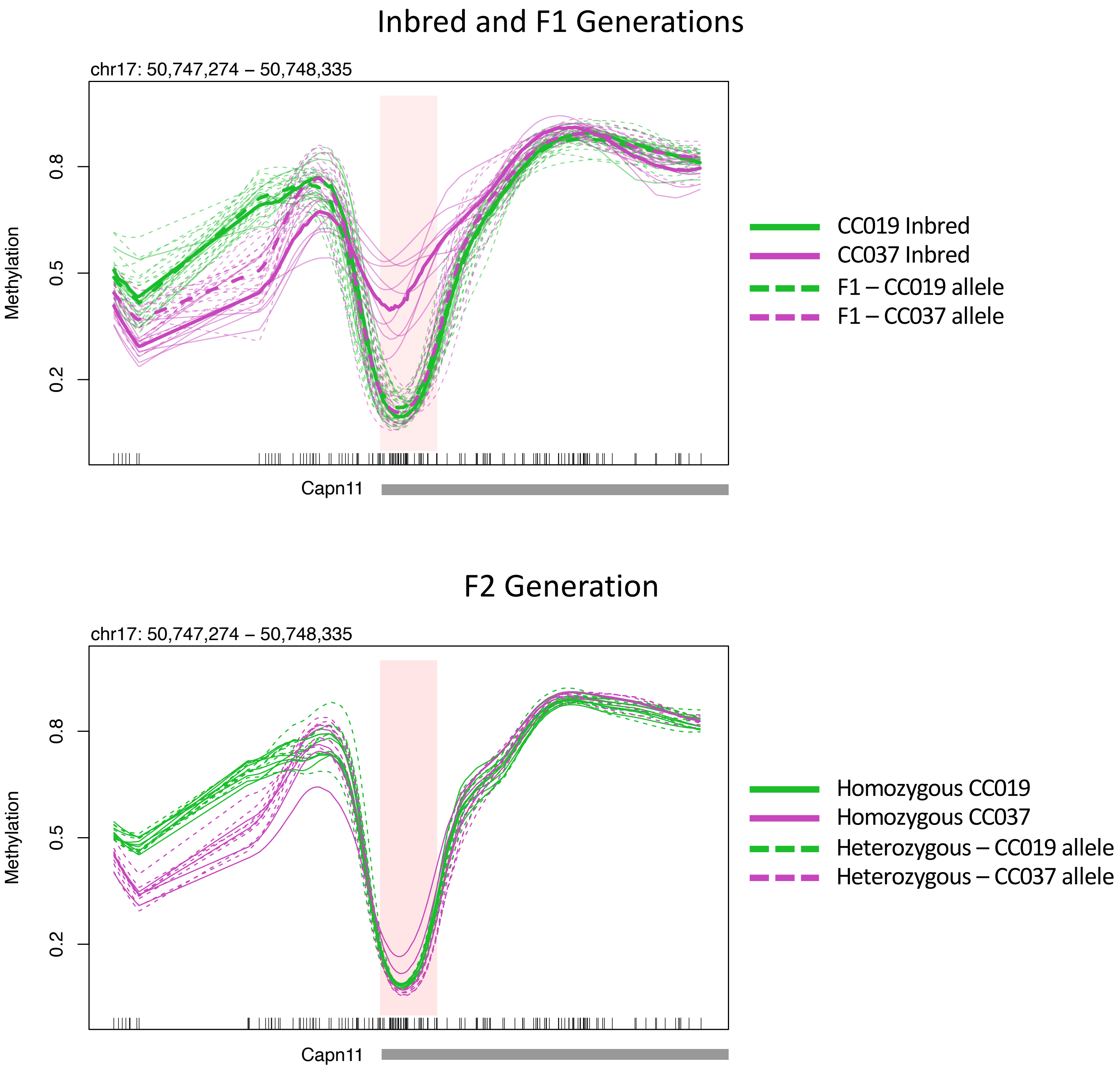

Supplementary Fig. 25. F2 candidate region methylation. Liver methylation from the F2 generation over the dominant *trans*-acting meQTL/transvection/paramutation DMRs chosen for targeted analysis in the F2s. Bold lines represent coverage-weighted mean methylation of the respective group and CpG sites included in the final analysis are denoted by tick marks on the x-axis.

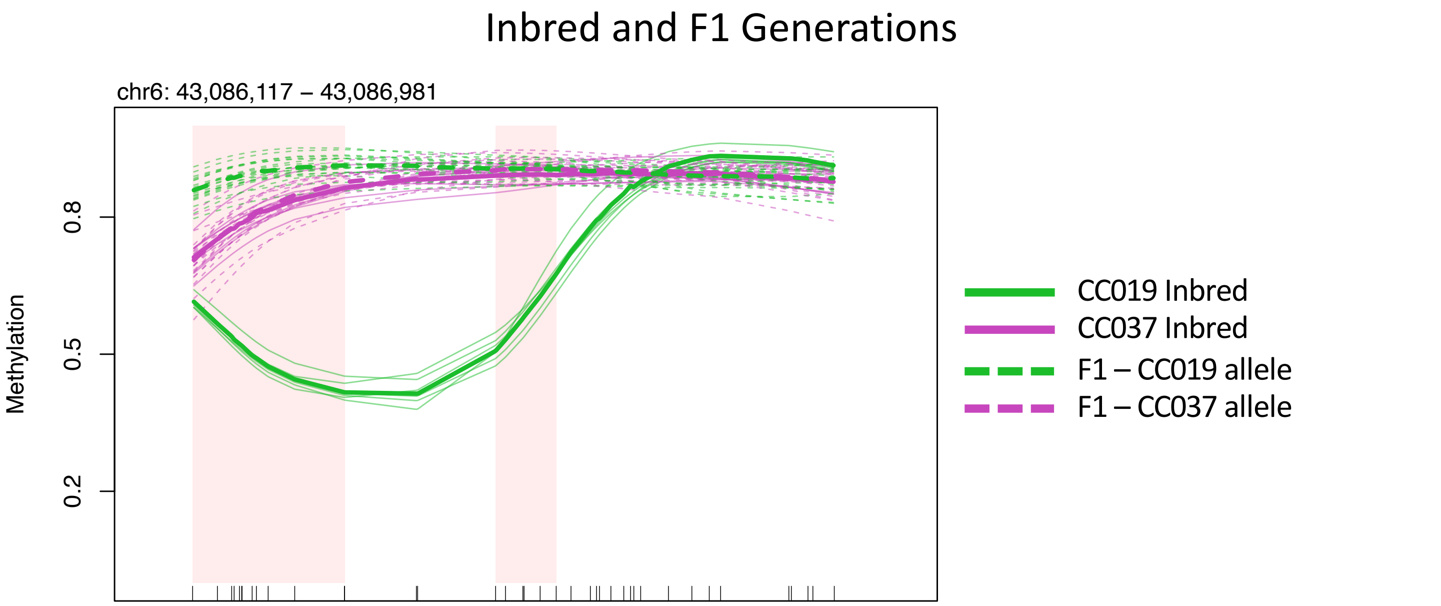

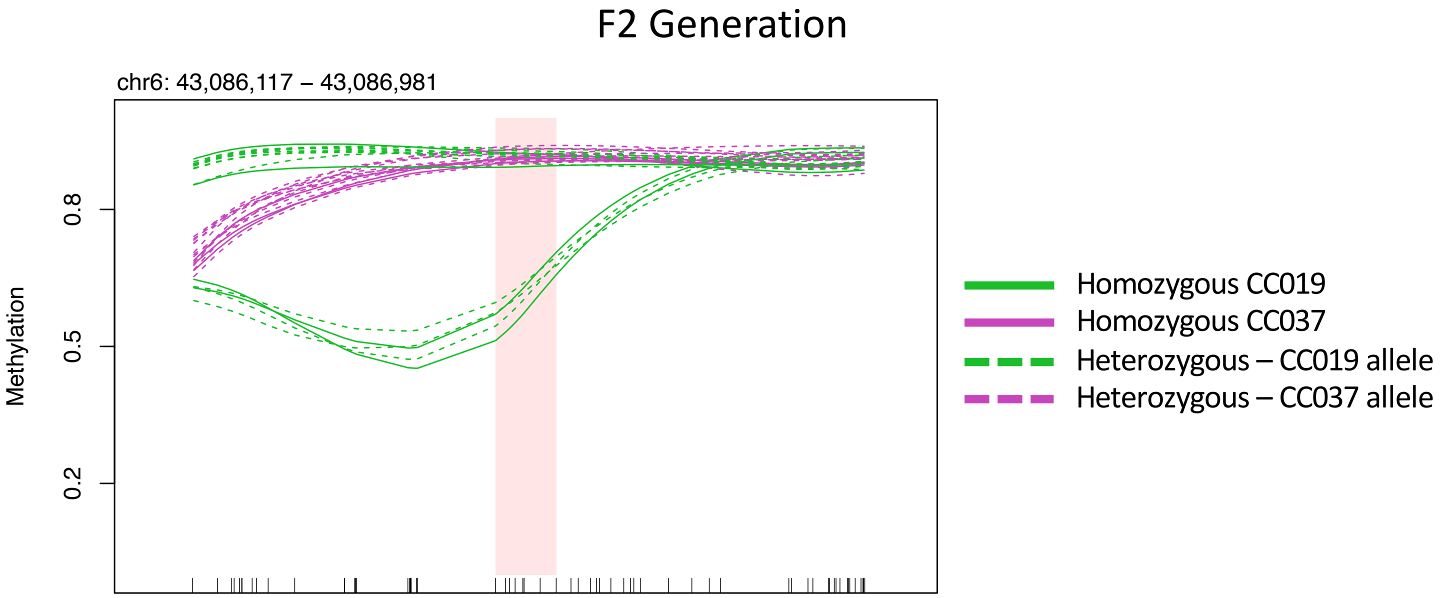

Supplementary Fig. 25. Continuation from previous page.

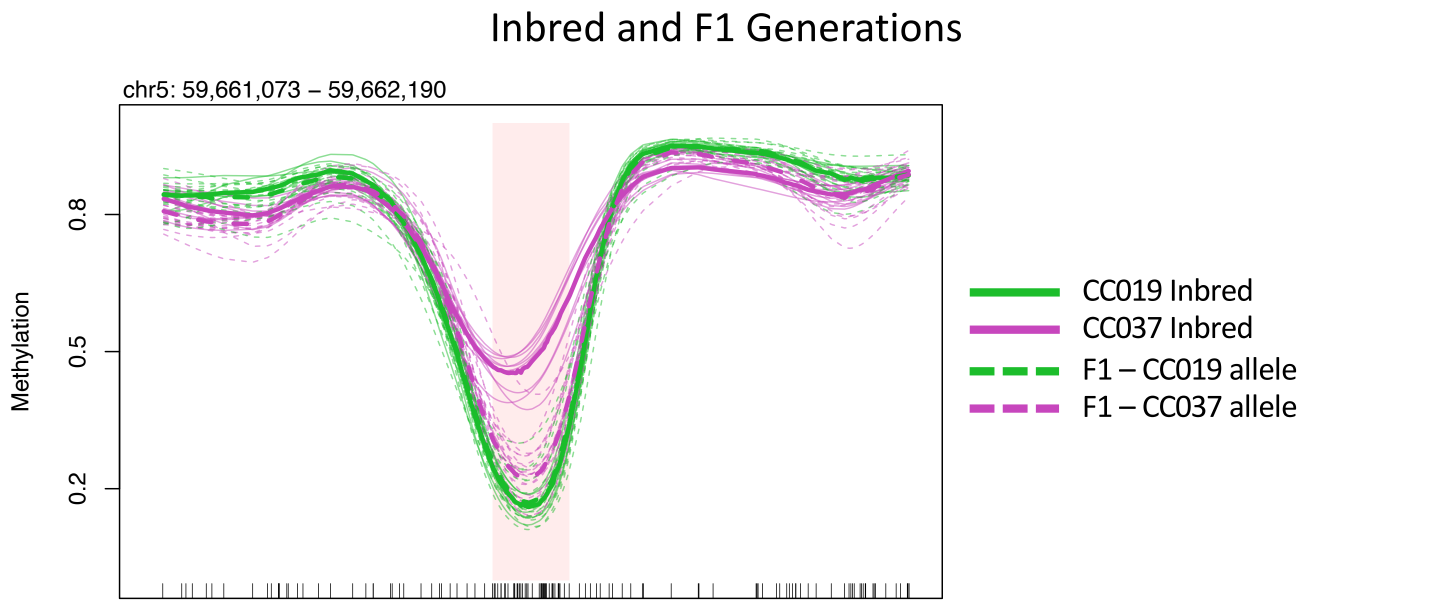

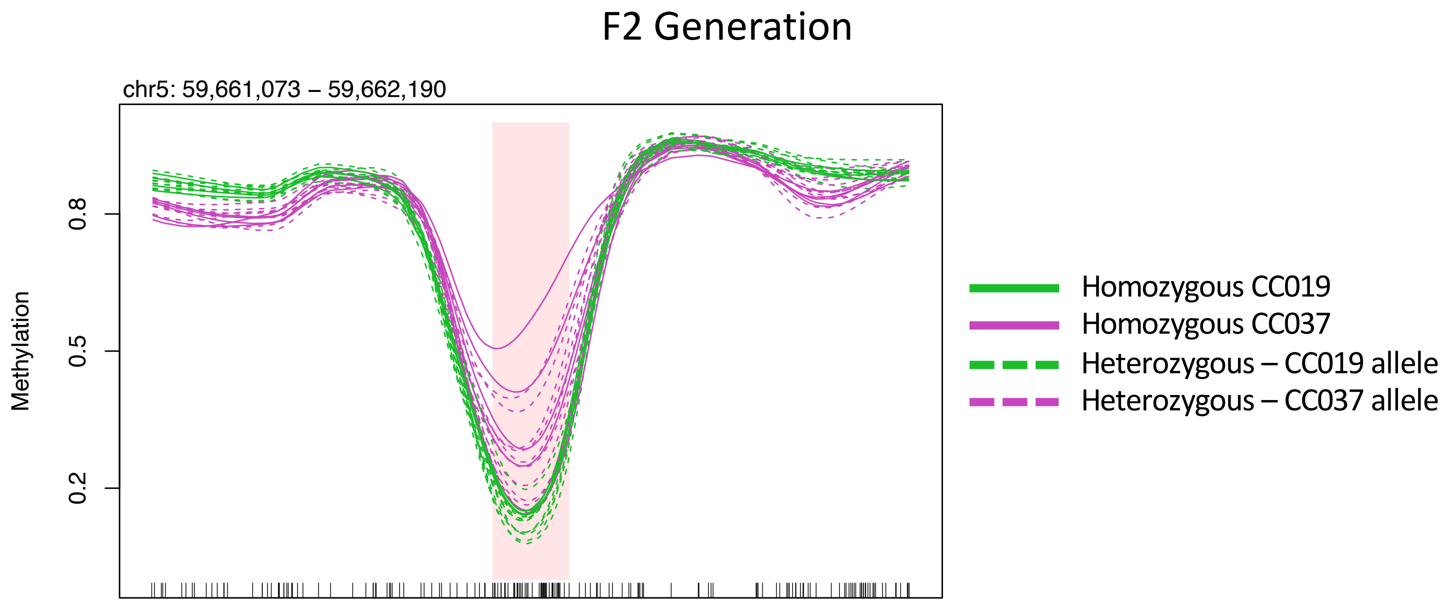

Supplementary Fig. 25. Continuation from previous page.

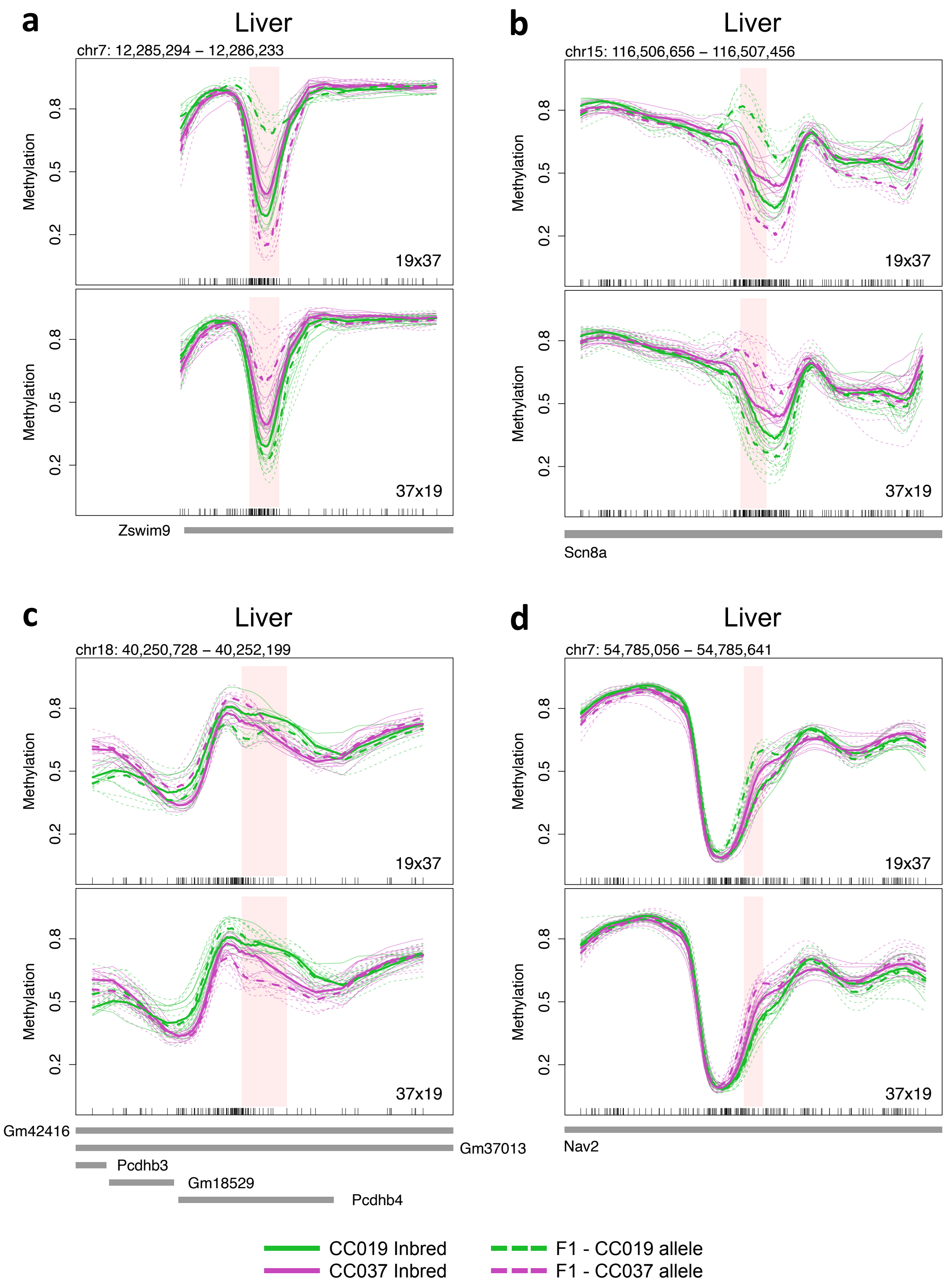

Supplementary Fig. 26. Novel and variably reported imprinted genes. DMRs categorized as genomic imprinting located nearby genes that have not been previously reported as imprinted or are variably reported as imprinted in the literature: **(a)** *Zswim9*, **(b)** *Scn8a*, **(c)** *Pcdhb4*, **(d)** *Nav2*, **(e)** *Casc1*, **(f)** *Fry*, **(g)** *Zfp92*, **(h)** *Cntnap1*, and **(i)** *Socs5*. For each region, the tissue in which the DMR was identified is indicated. Inbred samples and F1s from the 19x37 (top) and 37x19 (bottom) cross directions are shown. Bold lines represent coverage-weighted mean methylation of the respective group and CpG sites included in the final analysis are denoted by tick marks on the x-axis.

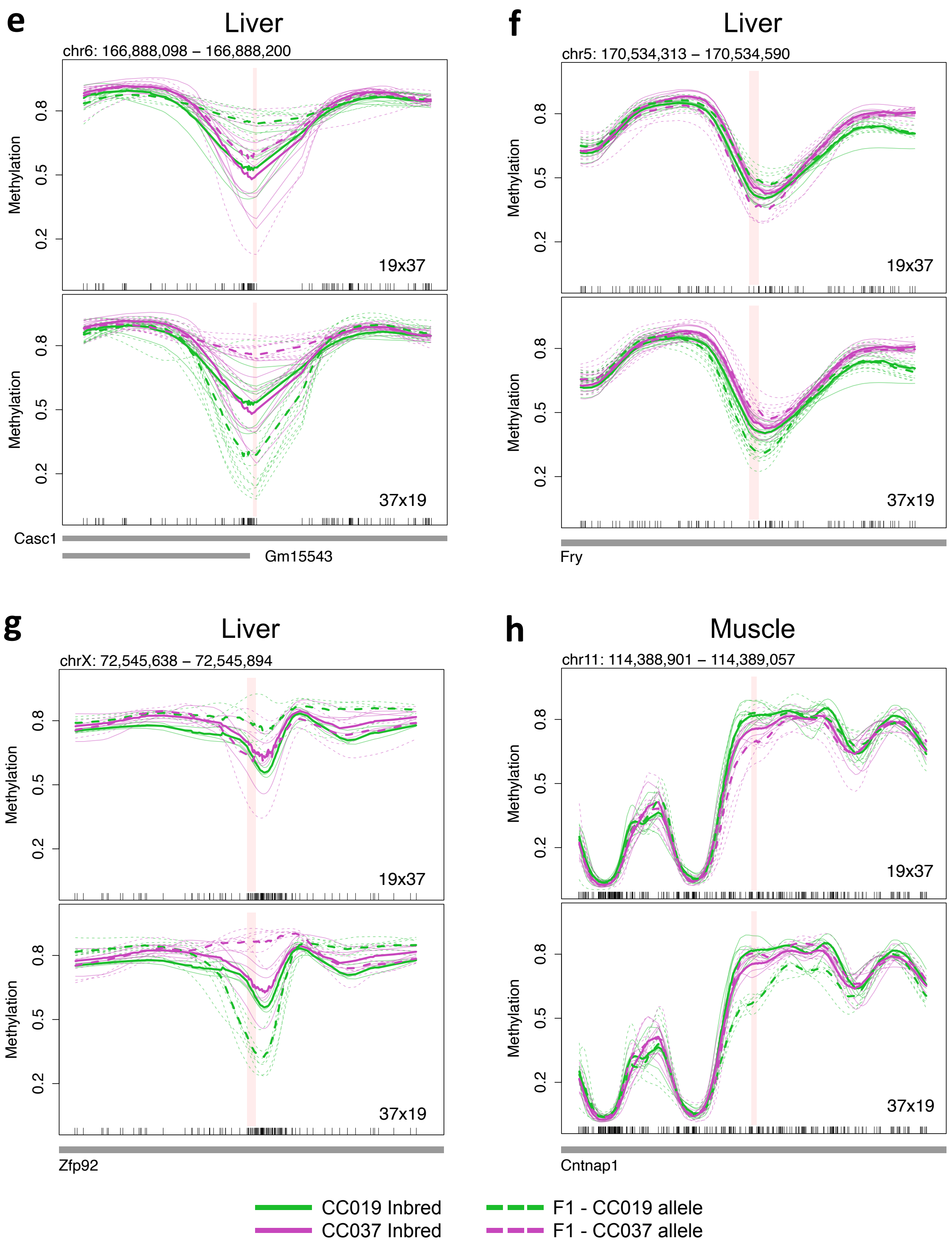

Supplementary Fig. 26. Continuation from previous page.

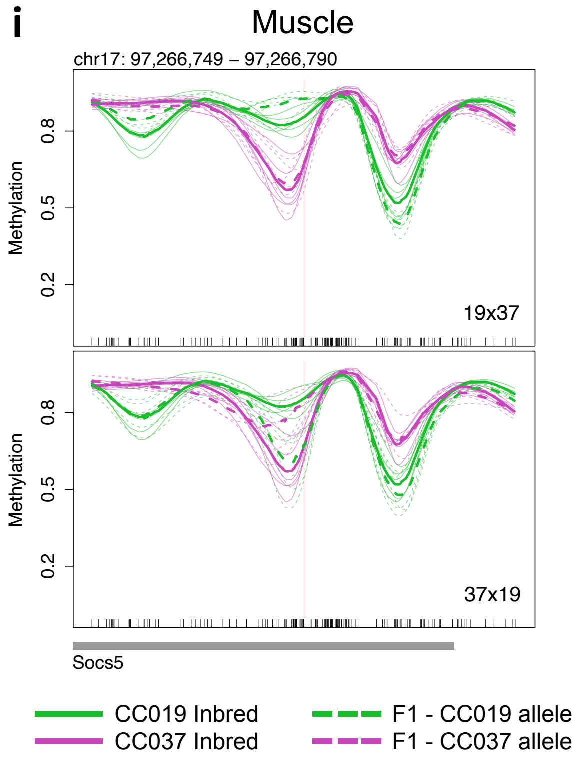

Supplementary Fig. 26. Continuation from previous page.

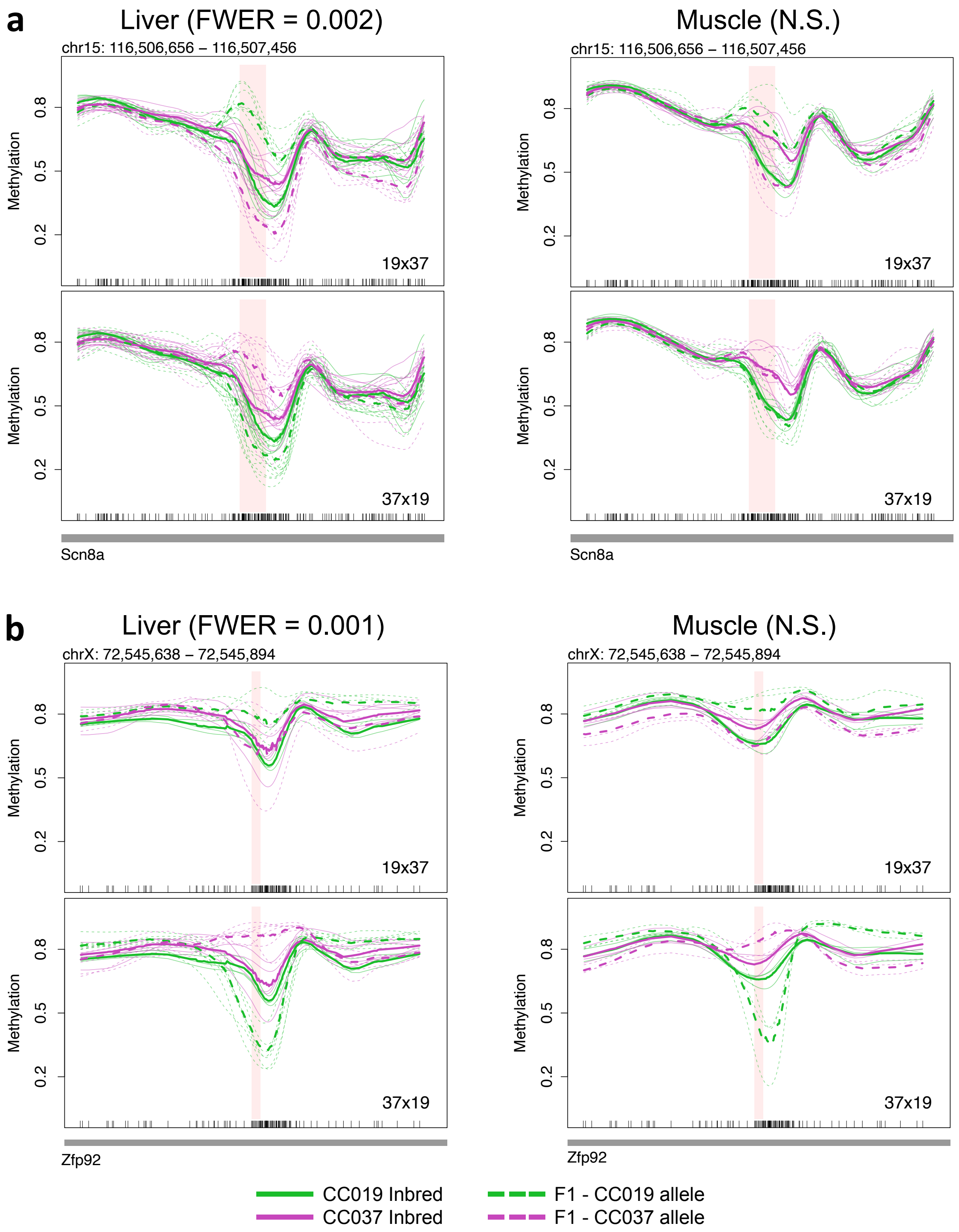

Supplementary Fig. 27. Methylation in the liver and muscle over novel imprinted genes. Methylation in both the liver (left) and the muscle (right) for DMRs categorized as genomic imprinting that are located nearby genes that have not been previously reported as imprinted in the literature and exhibit parent-of-origin-specific methylation patterns in both tissues: **(a)** *Scn8a*, **(b)** *Zfp92*, and **(c)** *Socs5*. For each region, the FWER value is provided for the tissue in which the DMR is significant while the other tissue is noted as not significant (N.S.). Inbred samples and F1s from the 19x37 (top) and 37x19 (bottom) cross directions are shown. Bold lines represent coverage-weighted mean methylation of the respective group and CpG sites included in the final analysis are denoted by tick marks on the x-axis.

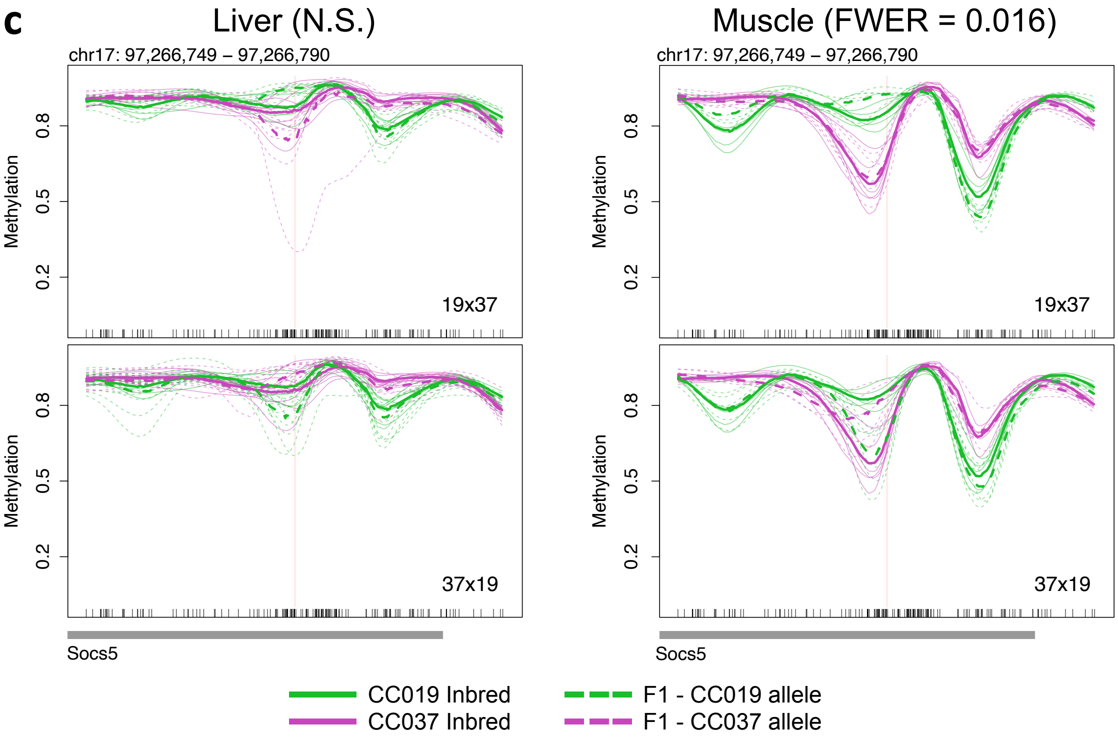

Supplementary Fig. 27. Continuation from previous page.

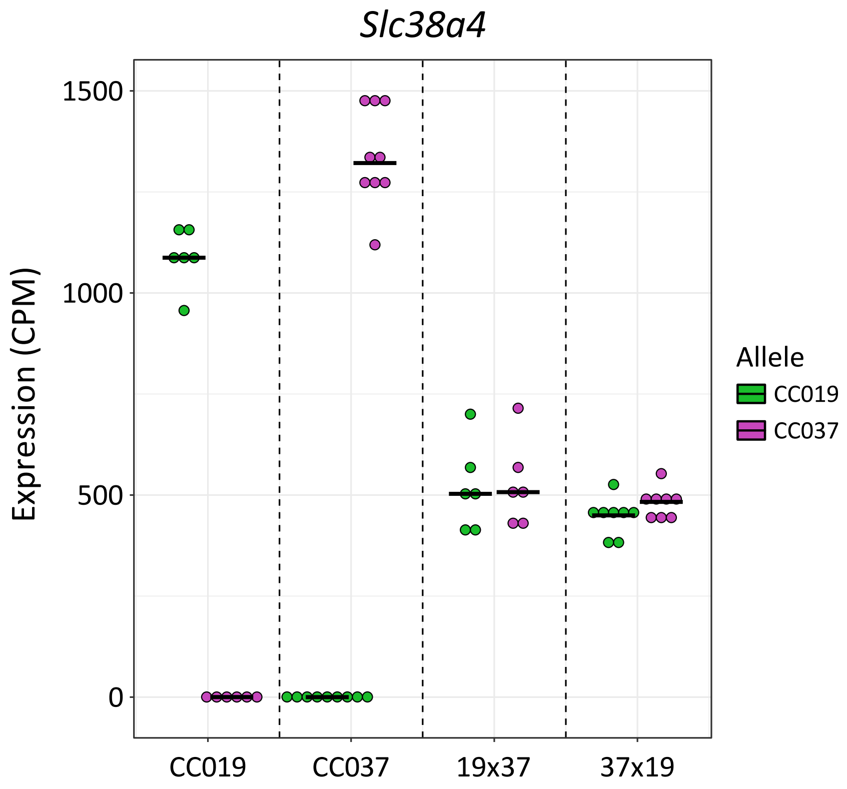

Supplementary Fig. 28. ***Slc38a4*** **allele-specific expression.** Liver allele-specific expression of the well-documented imprinted gene *Slc38a4* which does not exhibit parent-of-origin-specific expression despite having parent-of-origin-specific methylation over its ICR in the liver. Expression was analyzed in 6 CC019 inbred, 9 CC037 inbred, 6 CC019xCC037 F1, and 8 CC037xCC019 F1 mice. For the inbred samples (CC019 and CC037), the expression levels of the absent alleles are provided as technical controls confirming accurate allelic assignment.

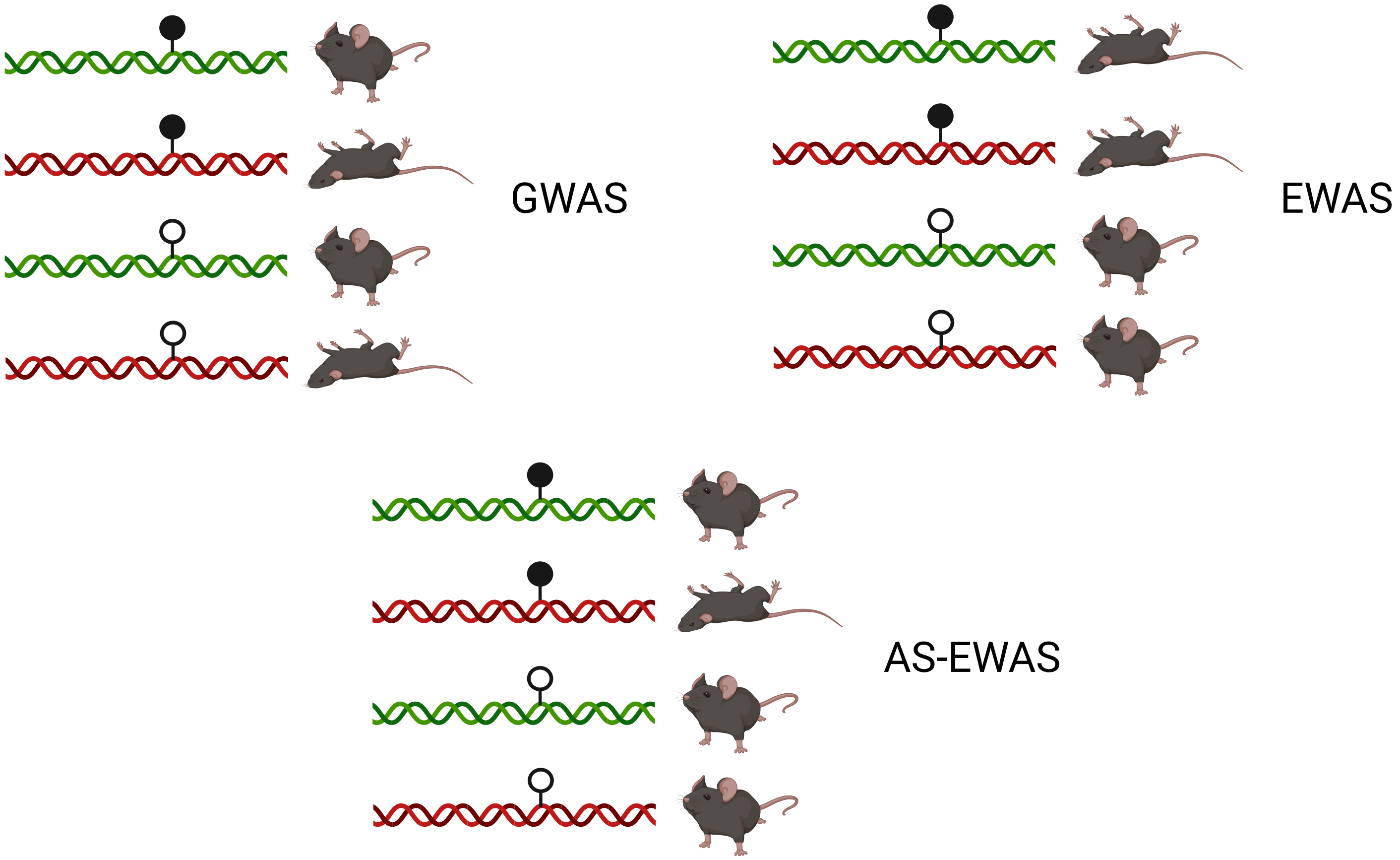

Supplementary Fig. 29. Allele-specific epigenome-wide association studies (AS-EWAS). We suggest an approach to identify disease or phenotype associations using a hybrid of GWAS and EWAS which exploits the ability to phase large portions of the genome using long-read sequencing. Shown for each type of analysis are the same four possible combined genetic and epigenetic alleles. The risk-associated genetic allele is in red, and the non-risk allele in green. The risk-associated methylation state is shown in black and the non-risk state is shown in white. In GWAS, the association is between the disease and the risk allele (both risk alleles are associated with the ailing mouse). In EWAS, the association is between the disease and the risk methylation state (both methylated alleles are associated with the ailing mouse). In AS-EWAS, the association is between the disease and phased allele-specific DNA methylation patterns (only the methylated allele in conjunction with the risk allele is associated with the ailing mouse).

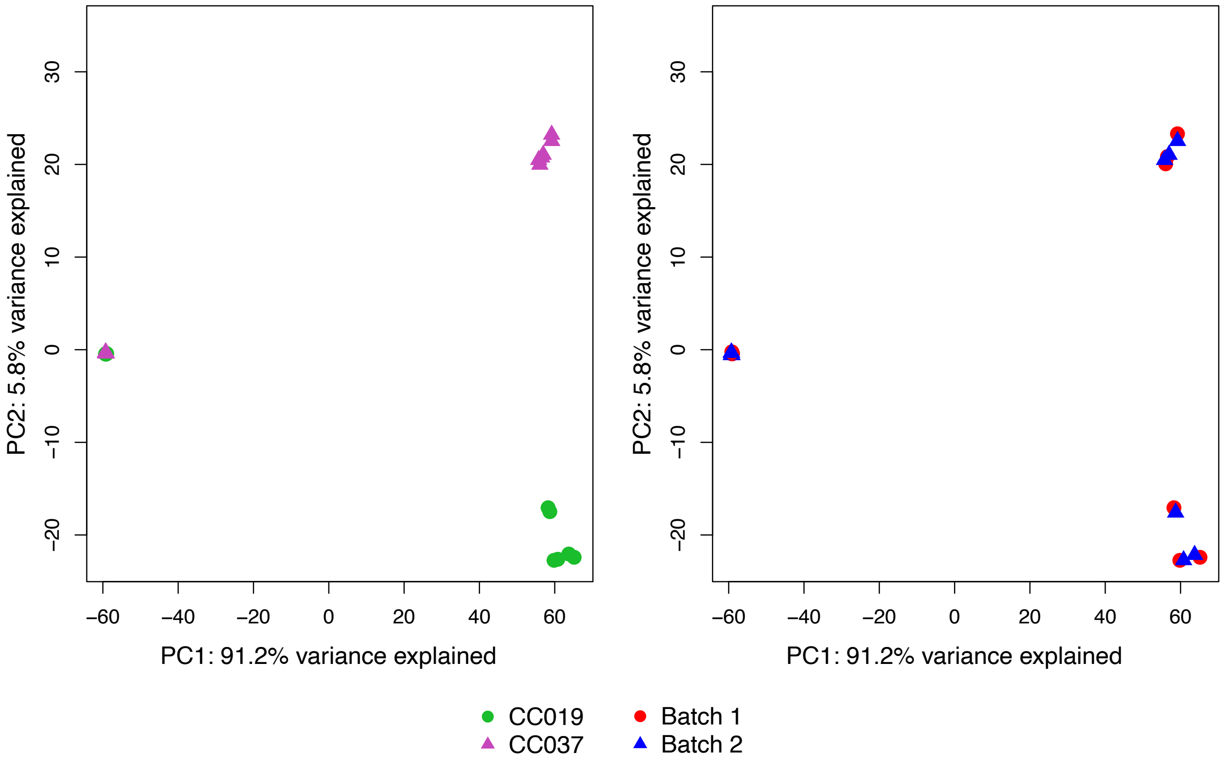

Supplementary Fig. 30. RNA sequencing batch PCA. PCA plots of the six samples included in both RNA-seq batches highlighted by allele (left) and batch (right). Note that the cluster of samples with negative values in PC1 are the 'incorrect' alleles, i.e. reads assigned to the CC037 allele from a CC019 inbred and reads assigned to the CC019 allele from a CC037 inbred.
