# Supplementary material for: Non-Mendelian inheritance of DNA methylation patterns in mice
Source: Nat Genet. 2026 May 20;58(6):1409–22. doi: 10.1038/s41588-026-02604-z (PMC13263155; doi:10.1038/s41588-026-02604-z)
Supplement: Supplementary file 2 — Reporting Summary [file 41588_2026_2604_MOESM2_ESM.pdf]

Corresponding author(s): Andrew P. Feinberg, David W. Threadgill, Kasper D. Hansen

Last updated by author(s): 11/26/2025

Reporting Summary

Nature Portfolio wishes to improve the reproducibility of the work that we publish. This form provides structure for consistency and transparency in reporting. For further information on Nature Portfolio policies, see our Editorial Policies and the Editorial Policy Checklist.

Statistics

For all statistical analyses, confirm that the following items are present in the figure legend, table legend, main text, or Methods section.

n/a	Confirmed	
☐	☒	The exact sample size (*n*) for each experimental group/condition, given as a discrete number and unit of measurement
☐	☒	A statement on whether measurements were taken from distinct samples or whether the same sample was measured repeatedly
☐	☒	The statistical test(s) used AND whether they are one- or two-sided<br>*Only common tests should be described solely by name; describe more complex techniques in the Methods section.*
☐	☒	A description of all covariates tested
☐	☒	A description of any assumptions or corrections, such as tests of normality and adjustment for multiple comparisons
☐	☒	A full description of the statistical parameters including central tendency (e.g. means) or other basic estimates (e.g. regression coefficient) AND variation (e.g. standard deviation) or associated estimates of uncertainty (e.g. confidence intervals)
☐	☒	For null hypothesis testing, the test statistic (e.g. *F*, *t*, *r*) with confidence intervals, effect sizes, degrees of freedom and *P* value noted<br>*Give P values as exact values whenever suitable.*
☒	☐	For Bayesian analysis, information on the choice of priors and Markov chain Monte Carlo settings
☒	☐	For hierarchical and complex designs, identification of the appropriate level for tests and full reporting of outcomes
☒	☐	Estimates of effect sizes (e.g. Cohen's *d*, Pearson's *r*), indicating how they were calculated

Our web collection on statistics for biologists contains articles on many of the points above.

Software and code

Policy information about availability of computer code

Data collection

No software was used.

Data analysis

Genome sequence and anchor information for CC019 and CC037 were extracted from the Collaborative Cross Graphical Genome (v2.0).

Two distinct analytical pipelines were used for the analysis of ONT data, one for each tissue (liver and muscle). For liver, basecalling was performed using Guppy (v6.1.2). Alignment was performed using MiniMap2 (v2.24). Prior to methylation calling, the FASTQ file was indexed alongside the sequencing summary file from Guppy super accurate basecalling using the Nanopolish (v0.14.0) index function. Methylation was called using the Nanopolish call-methylation function. Methylation calls were converted to methylation frequency values for each CpG using the Nanopolish calculate_methylation_frequency.py script. Heterozygous genetic variants were identified using the nucmer command from MUMmer (v4.0.0rc1). Haplotype phasing of aligned reads was performed using the WhatsHap (v1.6) haplotag function. We have created tools which are capable of mapping coordinates between the CCGG genomes and the intermediate genome, as well as between mm10 and the CCGG genomes. Methylation data was processed in R (v4.2.1) using the bsseq package (v1.38.0).

Muscle samples were processed as described above, with the following exceptions: (1) basecalling and methylation calling were performed using Dorado (v0.8.3), (2) the resulting unaligned, modified BAM file was then converted to FASTQ format using the samtools (v1.19.2) fastq command, (3) alignment was performed using MiniMap2 (v2.28), (4) reads with a MAPQ quality score of less than 20 were filtered out after alignment using the samtools view command, (5) haplotype phasing was performed using the WhatsHap (v2.3) haplotag function, and (6) methylation data was extracted from the final BAM files using the modkit (v0.4.1) pileup command. Samtools was also used for FASTQ and BAM indexing, sorting, and merging. The read.modkit function from the bsseq package (v1.42.0) was utilized to import methylation data from the modkit output files into R (v4.4.0). The resulting BS objects with combined 5mC and 5hmC calls were utilized for subsequent analyses in R (v4.3.1) using the bsseq package (v1.38.0).

For the analysis of RNA sequencing data, strain-specific transcriptomes for CC019 and CC037 were constructed using the gffread (v0.12.7) function. The diploid transcriptome was indexed using Salmon (v1.9.0). Sequenced reads were aligned to the diploid transcriptomes and allelic expression levels were quantified using the Salmon quant function. Allelic expression values were then calculated in R using the fishpond (v2.8.0) package. The transcript to gene mapping file was constructed using the BiomaRt (v2.58.0) package. Expression data was analyzed using DESeq2 (v1.42.0) package. Read-level methylation plots were generated for non-dominant trans-acting meQTLs and skewed XCI DMRs using methylartist (v1.2.10).

The processing and analysis code used in this manuscript is available through Zenodo using the following link: https://zenodo.org/records/11099825?token=eyJhbGciOiJIUzUxMiJ9.eyJpZCI6ImZhODI1ZmYxLTM5OTItNDFjYy1iODBjLTc1YTlmMTVhYzA0MiIsImRhdGEiOnt9LCJyYW5kb20iOiI4MTg5Mzk4NGNmNmM2N2JhYWVlNGQ4NmQwMzkyNzc4ZSJ9.ISBPDeQ4sSQuUOFiAKQX_ylNcKo9hNU_5qY-JN-WTqgtLPxy5FKvr3rNhNvxZl-jPlAdZvj3KHU6BahAtg8Wzw

For manuscripts utilizing custom algorithms or software that are central to the research but not yet described in published literature, software must be made available to editors and reviewers. We strongly encourage code deposition in a community repository (e.g. GitHub). See the Nature Portfolio guidelines for submitting code & software for further information.

Data

Policy information about availability of data

All manuscripts must include a data availability statement. This statement should provide the following information, where applicable:

- Accession codes, unique identifiers, or web links for publicly available datasets
- A description of any restrictions on data availability
- For clinical datasets or third party data, please ensure that the statement adheres to our policy

All ONT and RNA-seq FASTQ files have been deposited to the NIH Sequence Read Archive (BioProject: PRJNA1107693, reviewer link: https://dataview.ncbi.nlm.nih.gov/object/PRJNA1107693?reviewer=oad1eaicl7kfqum3h07imh46gs). Methylation frequency files, Guppy sequencing summary files, Salmon quantification files, and allele-specific gene counts have been deposited to the NCBI Gene Expression Omnibus (accession code: GSE266668, reviewer token: spuvykoctlkjnsb). F2 genotyping data has been deposited to Zenodo and is available using the following link: https://zenodo.org/records/17148323?token=eyJhbGciOiJIUzUxMiJ9.eyJpZCI6ImYyMGFlYzc4LWI4YzUtNDhjYy04YjJkLTIyZGYwY2UxOGQwZCIsImRhdGEiOnt9LCJyYW5kb20iOiI0YWJhMWRiZDc4MzQ2NWNiOWMyZWM3YzJiMGU5ZDJlYyJ9.slzgk2PKFXzKjxD7YpjSt7djZUreDSo_Rdnw1FLqDAfk_Y6_lakpLlatla7NXQEsRJOGiDfsV7u-aaTl3nZIHA

Research involving human participants, their data, or biological material

Policy information about studies with human participants or human data. See also policy information about sex, gender (identity/presentation), and sexual orientation and race, ethnicity and racism.

Reporting on sex and gender	N/A
Reporting on race, ethnicity, or other socially relevant groupings	N/A
Population characteristics	N/A
Recruitment	N/A
Ethics oversight	N/A

Note that full information on the approval of the study protocol must also be provided in the manuscript.

Field-specific reporting

Please select the one below that is the best fit for your research. If you are not sure, read the appropriate sections before making your selection.

☒ Life sciences ☐ Behavioural & social sciences ☐ Ecological, evolutionary & environmental sciences

For a reference copy of the document with all sections, see nature.com/documents/nr-reporting-summary-flat.pdf

Life sciences study design

All studies must disclose on these points even when the disclosure is negative.

Sample size	A sufficient number of mice for each tissue of the inbred and F1 generations was chosen such that there would be a minimum sample size of three per strain/cross/sex/tissue for all comparisons. Additionally, a minimal set of F2s was chosen for targeted ONT sequencing which satisfied the following two conditions: (1) for each of the candidate dominant trans-acting meQTL/transvection/paramutation regions to be targeted there are more than five heterozygous samples and three homozygous samples for each parental allele, CC019 and CC037; and (2) for each region in the genome there is at least one sample which is homozygous for each parental strain, i.e. there are no contiguous three SNPs in the genome which harbor at least one copy of the same genetic variants in all selected mice.

Data exclusions	One female CC019xCC037 F1 liver sample was removed from subsequent methylation and expression analyses as phasing of the X chromosome revealed only one parental allele, indicating monosomy of the X chromosome (X0). One male CC019xCC037 was removed from subsequent expression analyses due to incorrect sequencing read depth (~360M compared to 50M targeted). One female CC019xCC037 F1 sample was removed from subsequent expression analyses as an outlier (total expression of the outlier was more than 3 standard deviations away from the mean in 6,927/12,894 expressed genes). One male CC037xCC019 F1 sample was removed from subsequent expression analyses due to poor mapping to the diploid transcriptome (68.8% mapping rate).
Replication	After genome-wide identification of our diverse set of epigenetic inheritance patterns in the liver of the inbred and F1 generations, we performed subsequent targeted ONT sequencing on DNA extracted from the liver of 19 mice from the F2 generation. For this experiment, we targeted three dominant trans-acting meQTL/ transvection/paramutation DMRs, 20 cis-acting meQTL DMRs, 5 non-dominant trans-acting meQTL DMRs, 20 sex-specific DMRs, 5 imprinted DMRs, 10 skewed XCI DMRs, and 21 regions which were included based on a preliminary analysis but are no longer of interest and have thus been removed from subsequent analysis. This data was used to further investigate the genetic and non-genetic factors regulating the identified epigenetic inheritance patterns as well as to validate epigenetic inheritance patterns mediated by cis- and trans-acting regulatory factors.<br><br>In addition to sequencing these F2 mice, we also performed an additional analysis of these intergenerational epigenetic inheritance patterns on DNA extracted from the muscle of the inbred and F1 generations of distinct mice from the same strains as analyzed in the liver.
Randomization	This study involved no exposures or treatments that would require randomization. Samples included in each ONT sequencing run were randomized with considerations for an even distribution of samples from each strain/cross/sex included in each run.
Blinding	This study involved no exposures or treatments that would require blinding.

Reporting for specific materials, systems and methods

We require information from authors about some types of materials, experimental systems and methods used in many studies. Here, indicate whether each material, system or method listed is relevant to your study. If you are not sure if a list item applies to your research, read the appropriate section before selecting a response.

Materials & experimental systems

n/a	Involved in the study
☒	☐ Antibodies
☒	☐ Eukaryotic cell lines
☒	☐ Palaeontology and archaeology
☐	☒ Animals and other organisms
☒	☐ Clinical data
☒	☐ Dual use research of concern
☒	☐ Plants

Methods

n/a	Involved in the study
☒	☐ ChIP-seq
☒	☐ Flow cytometry
☒	☐ MRI-based neuroimaging

Animals and other research organisms

Policy information about studies involving animals; ARRIVE guidelines recommended for reporting animal research, and Sex and Gender in Research

Laboratory animals	The Collaborative Cross lines, specifically CC019/TauUnc and CC037/TauUnc were sourced from the Systems Genetics Core Facility at the University of North Carolina at Chapel Hill and underwent breeding and maintenance at Texas A&M University. F1 mice were generated by crossing CC019/TauUnc females with CC037/TauUnc males (CC019 x CC037) and CC037/TauUnc females with CC019/TauUnc males (CC037 x CC019). The F1 mice were subsequently intercrossed to produce three distinct F2 populations: [(CC019 x CC037) x (CC019 x CC037)], [(CC019 x CC037) x (CC037 x CC019)], and [(CC037 x CC019) x (CC037 x CC019)]. Samples were collected from all mice at approximately 4 months of age.
Wild animals	The study did not involve wild animals.
Reporting on sex	Sex has been considered in thus study, including the identification of autosomal sex-specific methylation patterns, parent-of-origin-specific methylation, and tests performed on the X chromosome separately for each sex. Furthermore, a roughly even mixture of both sexes has been included in all sample sets analyzed. Sex was assigned using morphological characteristics and confirmed by genotyping.
Field-collected samples	The study did not involve samples collected from the field.
Ethics oversight	All experiments were performed in accordance and approval by Texas A&M University Institution of Animal Care and Use Committee (IACUC 2022-0273).

Note that full information on the approval of the study protocol must also be provided in the manuscript.

Plants

Seed stocks	N/A
Novel plant genotypes	N/A
Authentication	N/A
