# Supplementary material for: Non-Mendelian inheritance of DNA methylation patterns in mice
Source: Nat Genet. 2026 May 20;58(6):1409–22. doi: 10.1038/s41588-026-02604-z (PMC13263155; doi:10.1038/s41588-026-02604-z)

Dominant *trans*-acting meQTL/Transvection/Paramutation

Inbred and F1 Generations

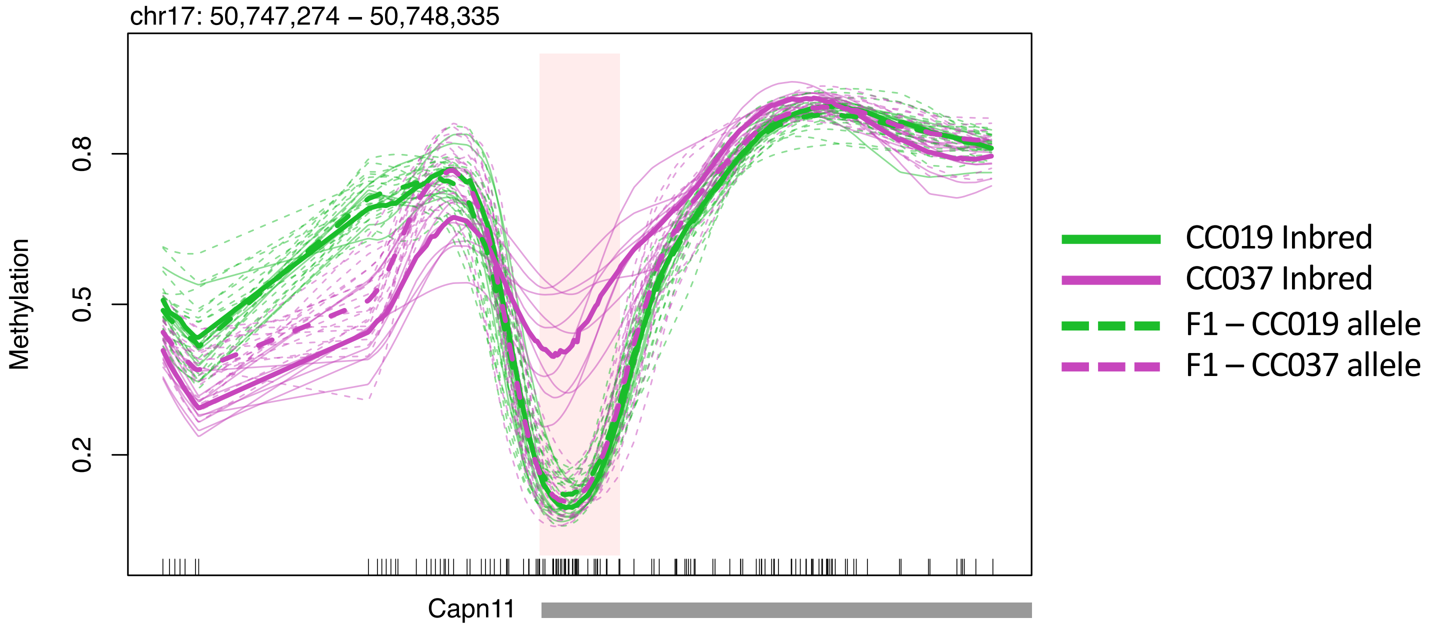

F2 Generation

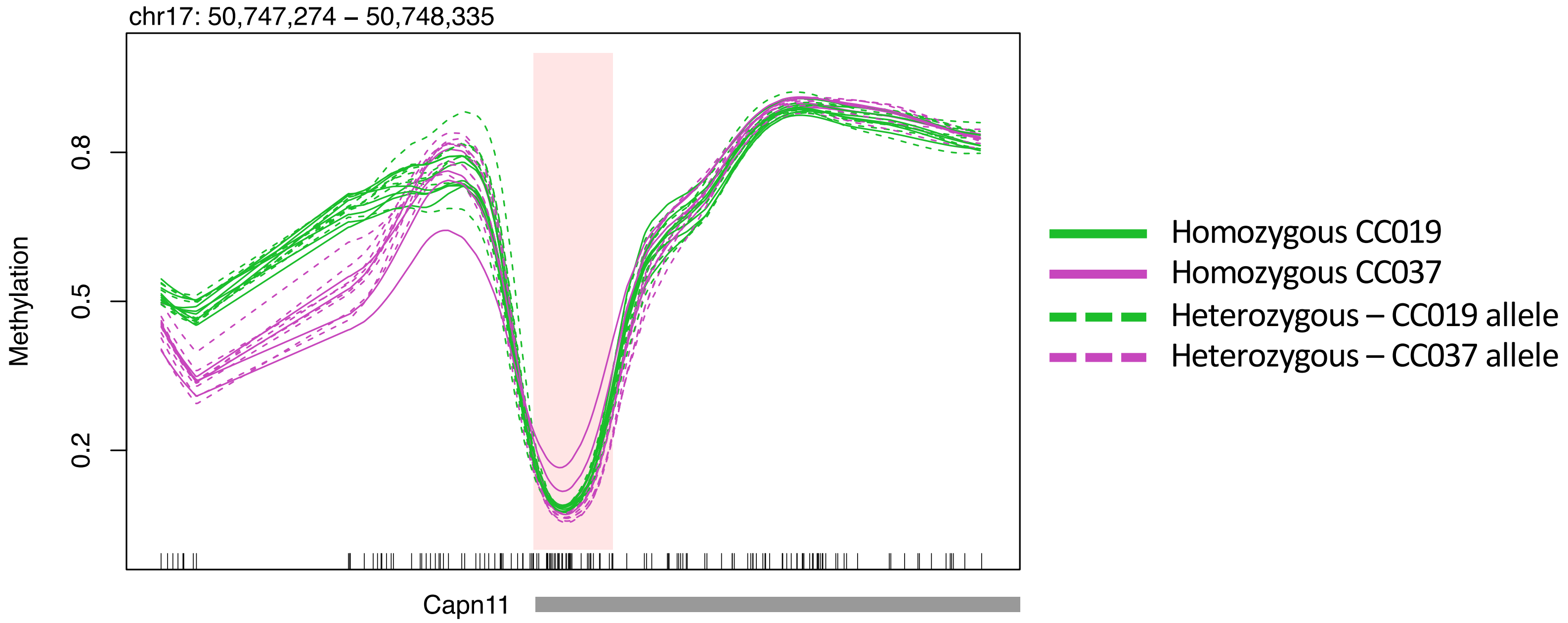

Dominant *trans*-acting meQTL/Transvection/Paramutation

Inbred and F1 Generations

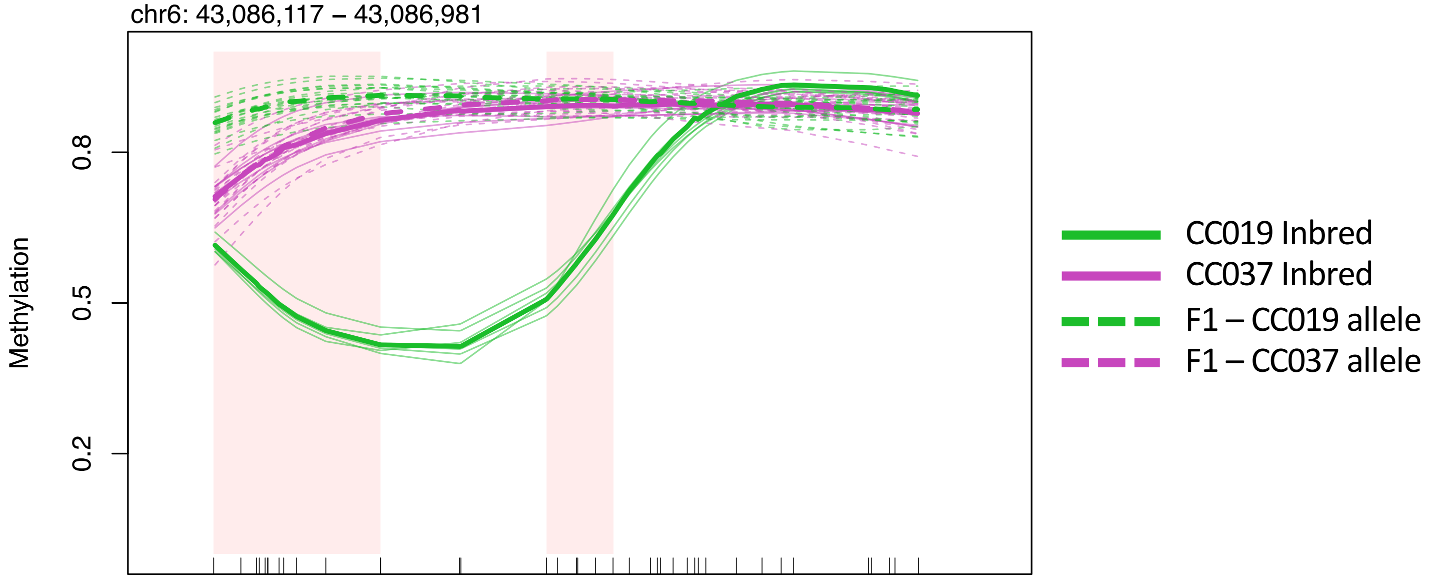

F2 Generation

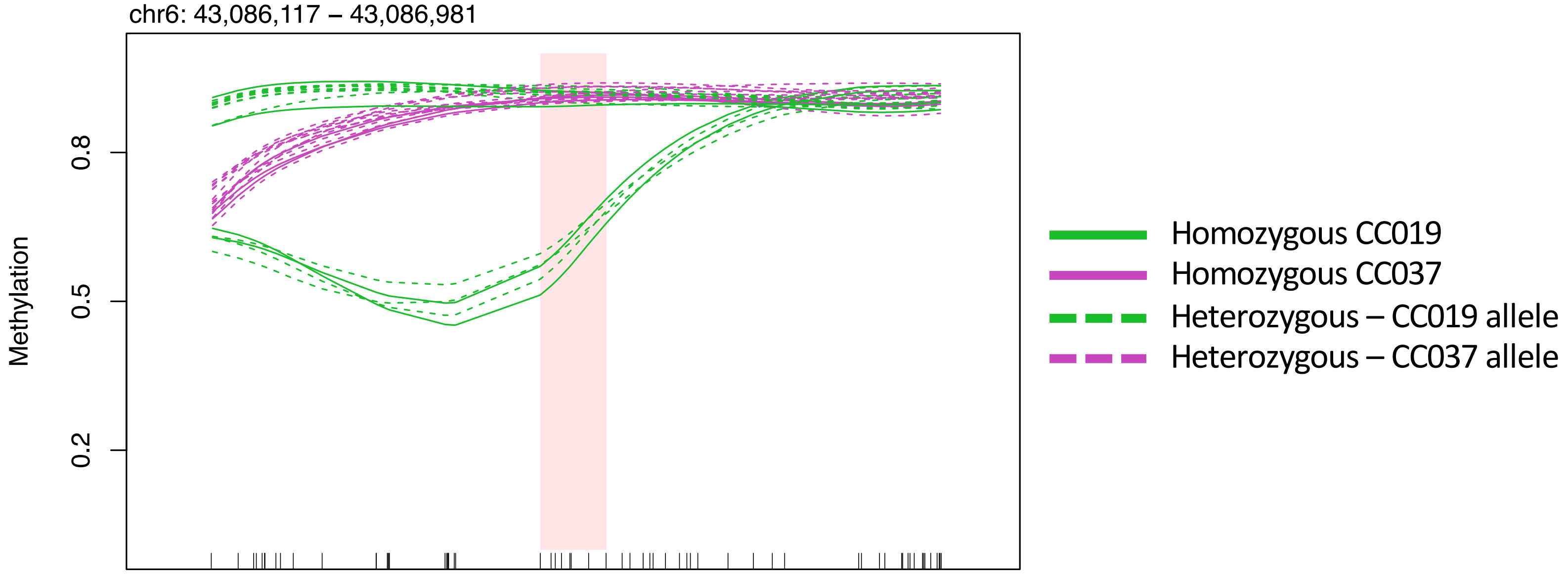

Dominant *trans*-acting meQTL/Transvection/Paramutation

Inbred and F1 Generations

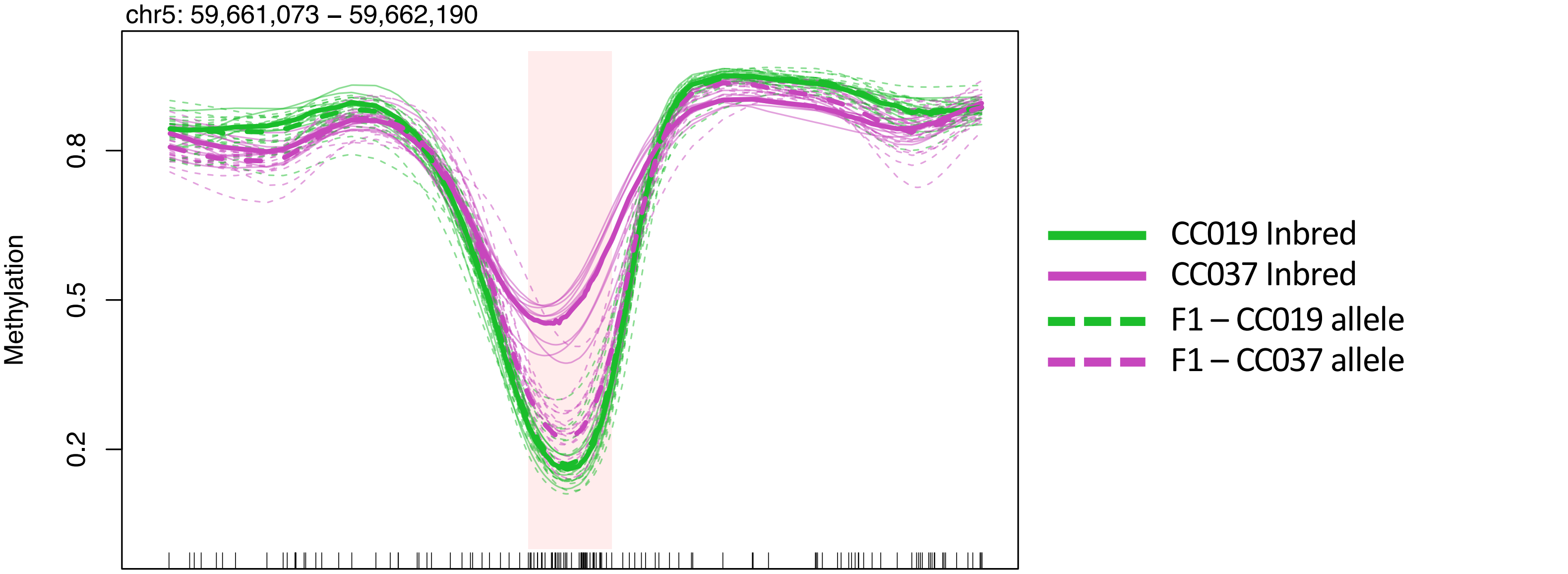

F2 Generation

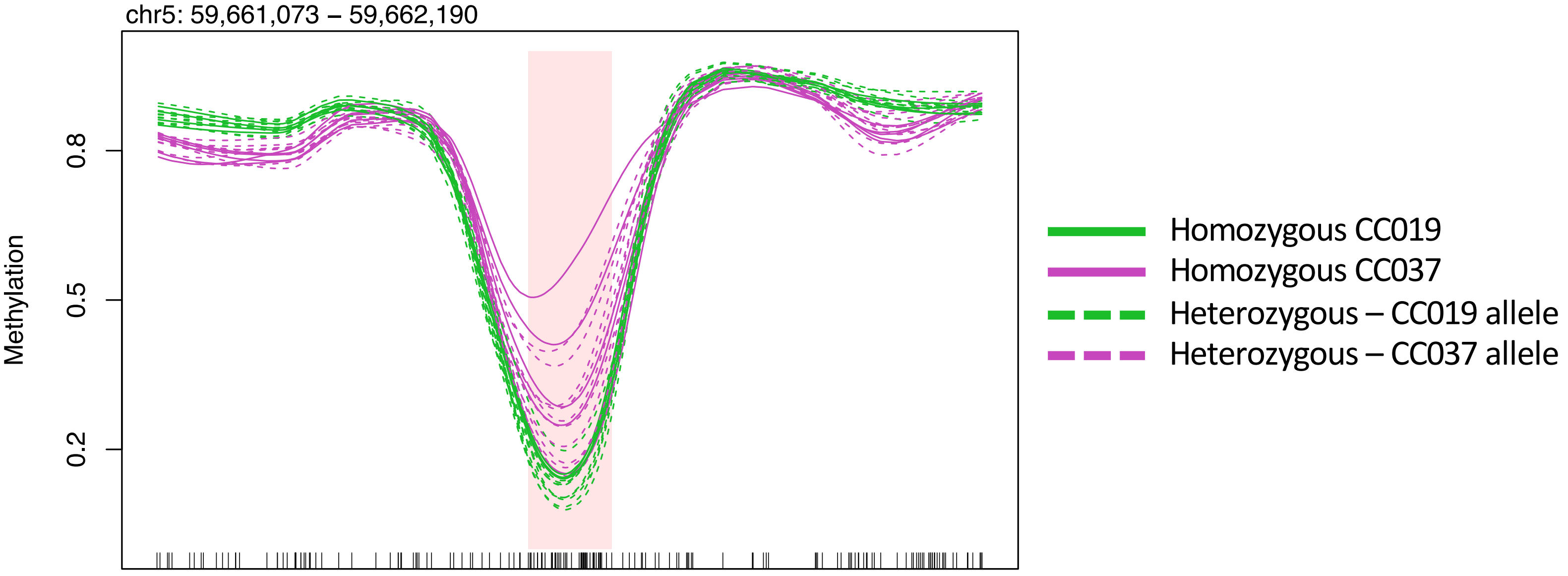

Cis-acting meQTL

Inbred and F1 Generations

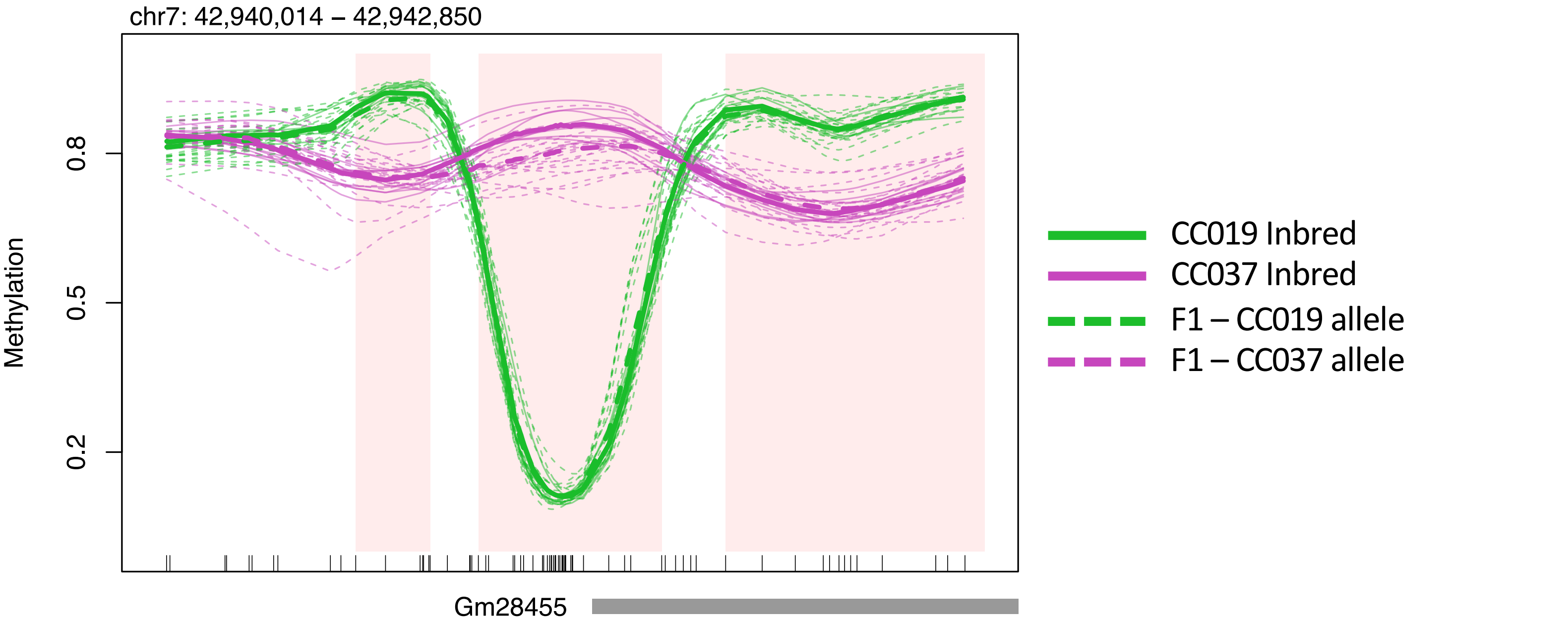

F2 Generation

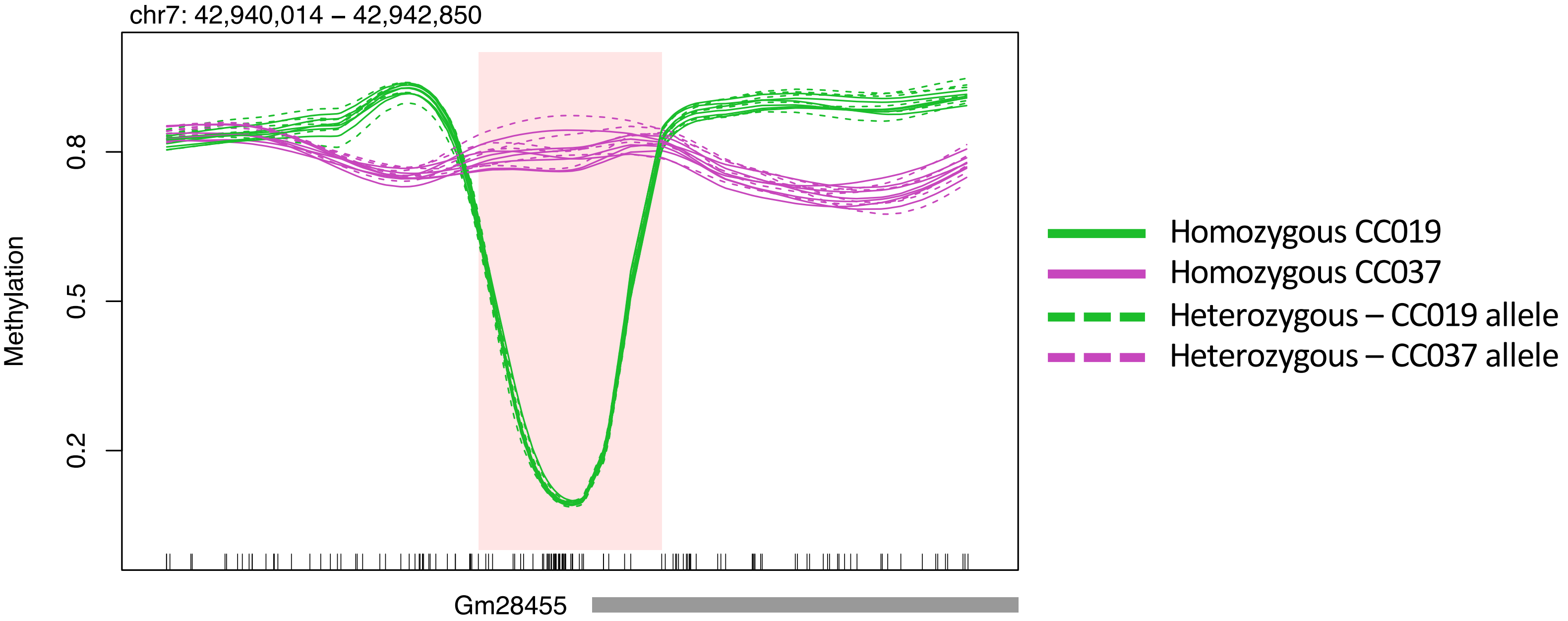

Cis-acting meQTL

Inbred and F1 Generations

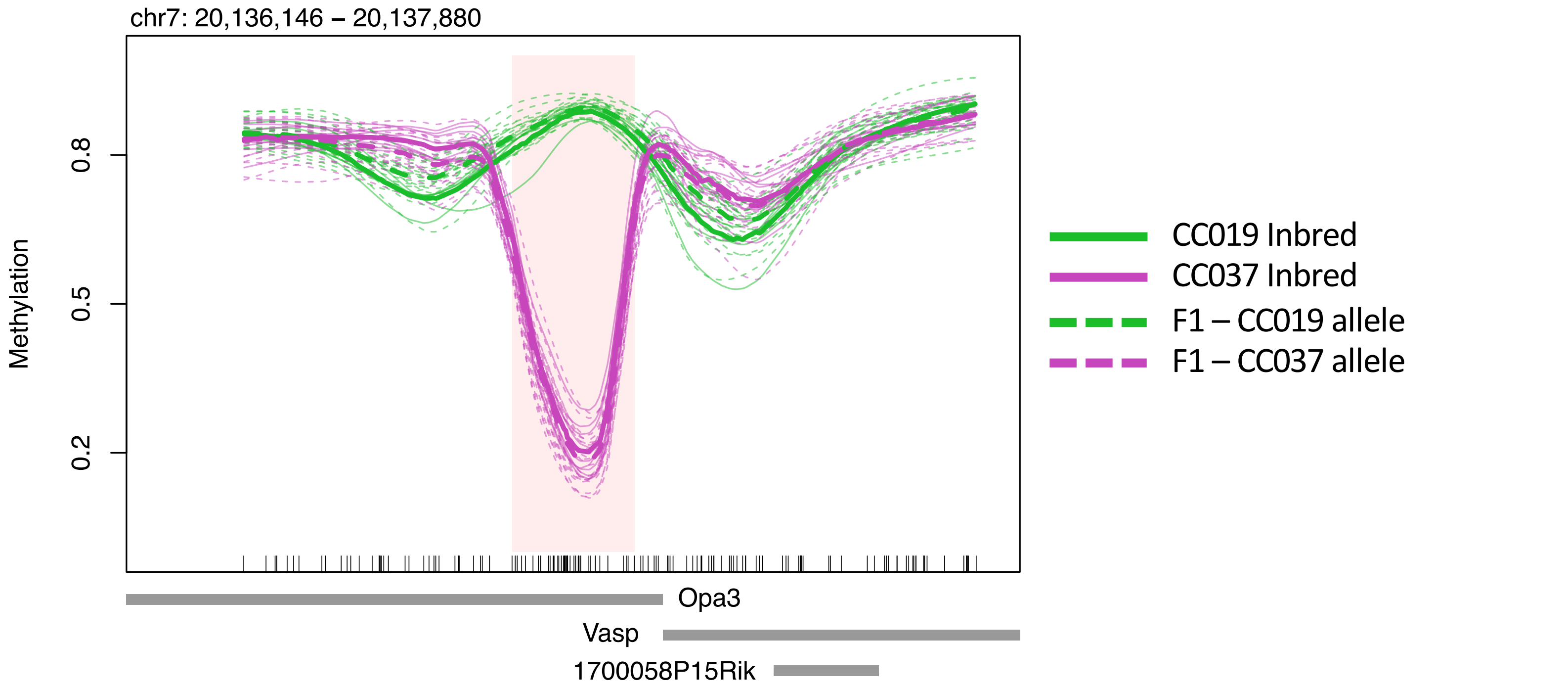

F2 Generation

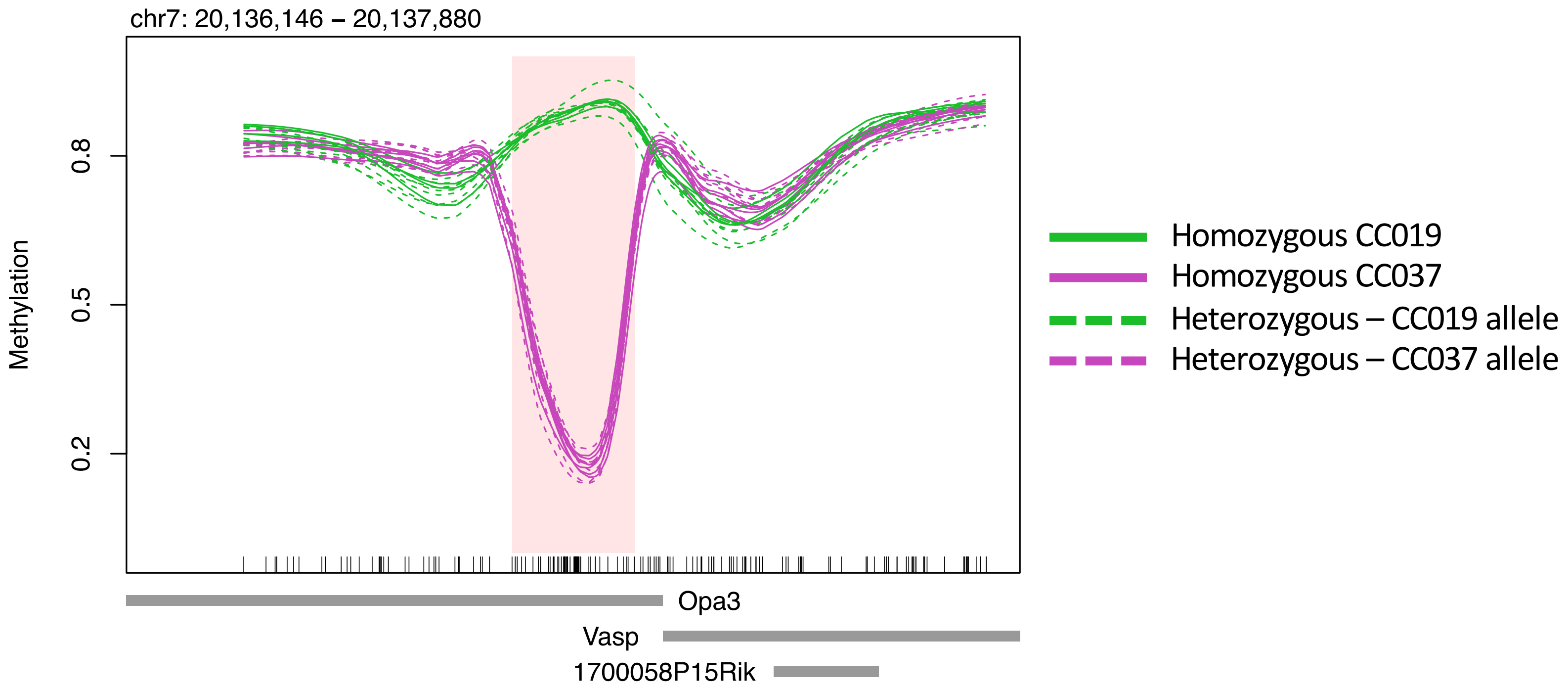

Cis-acting meQTL

Inbred and F1 Generations

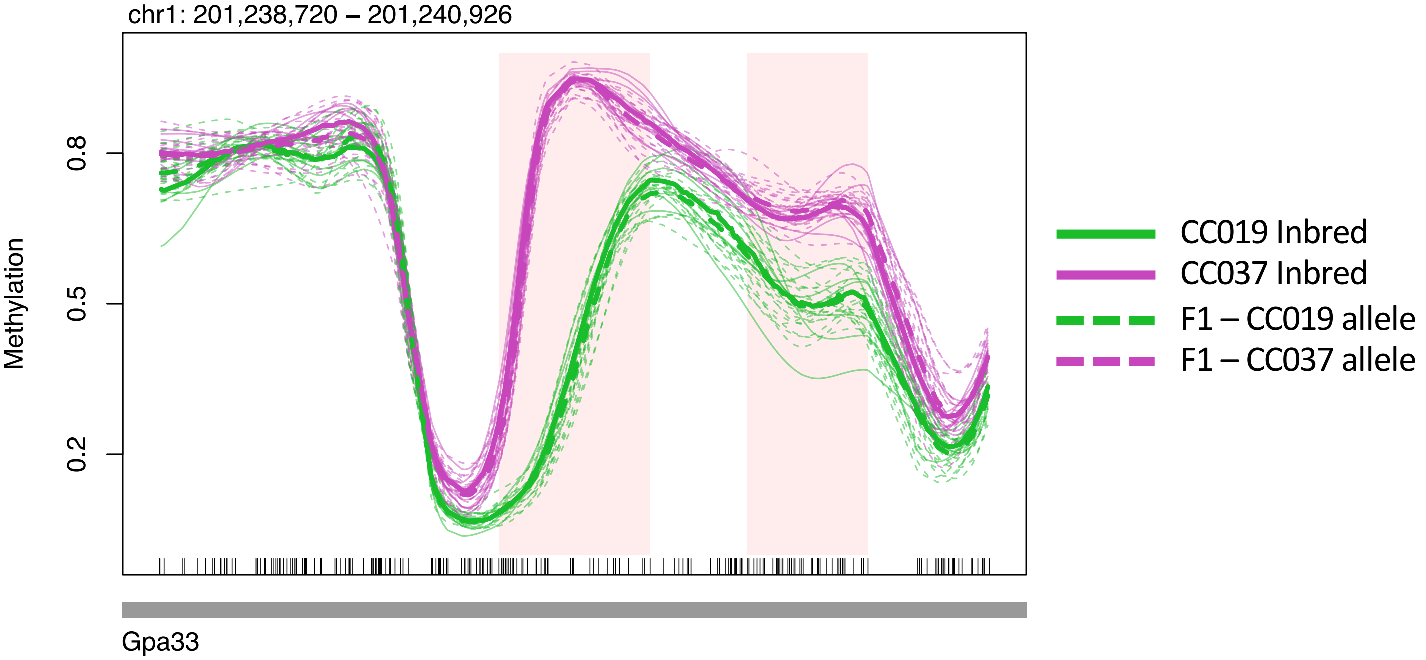

F2 Generation

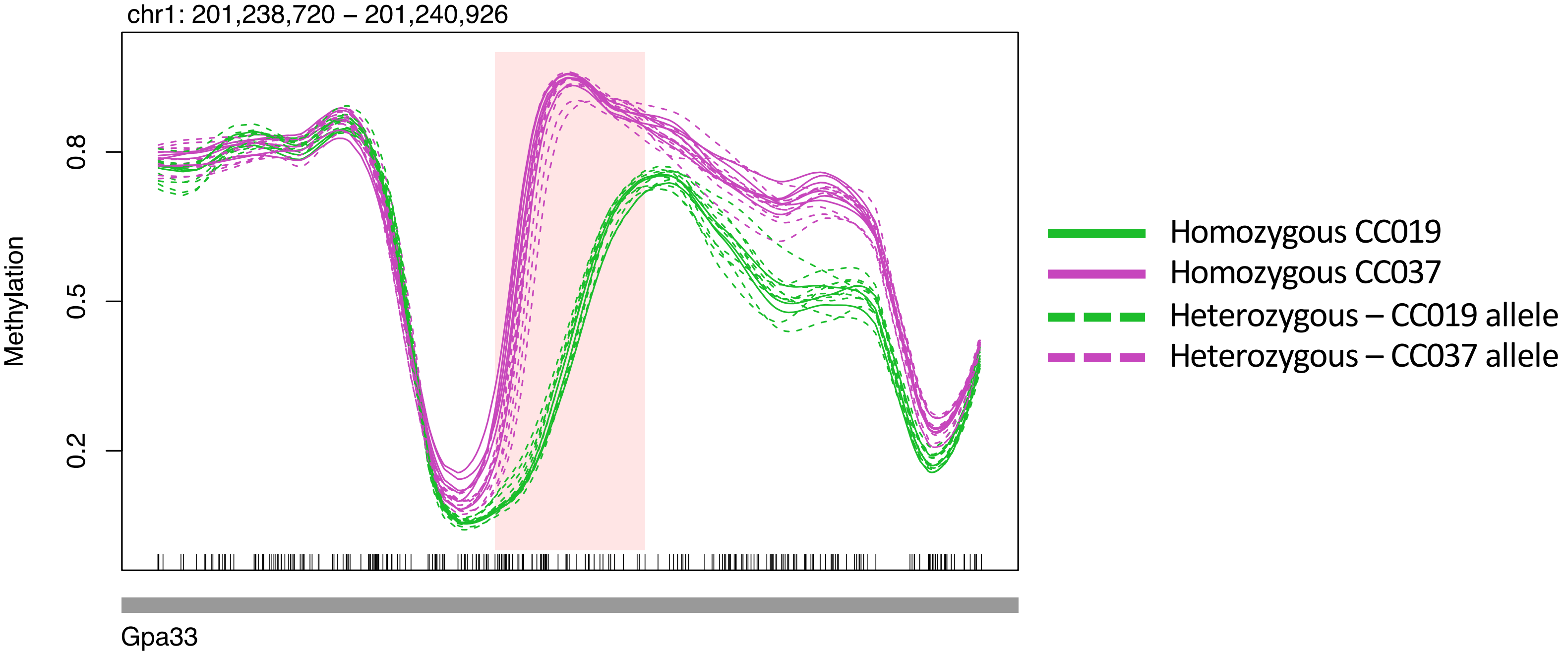

Cis-acting meQTL

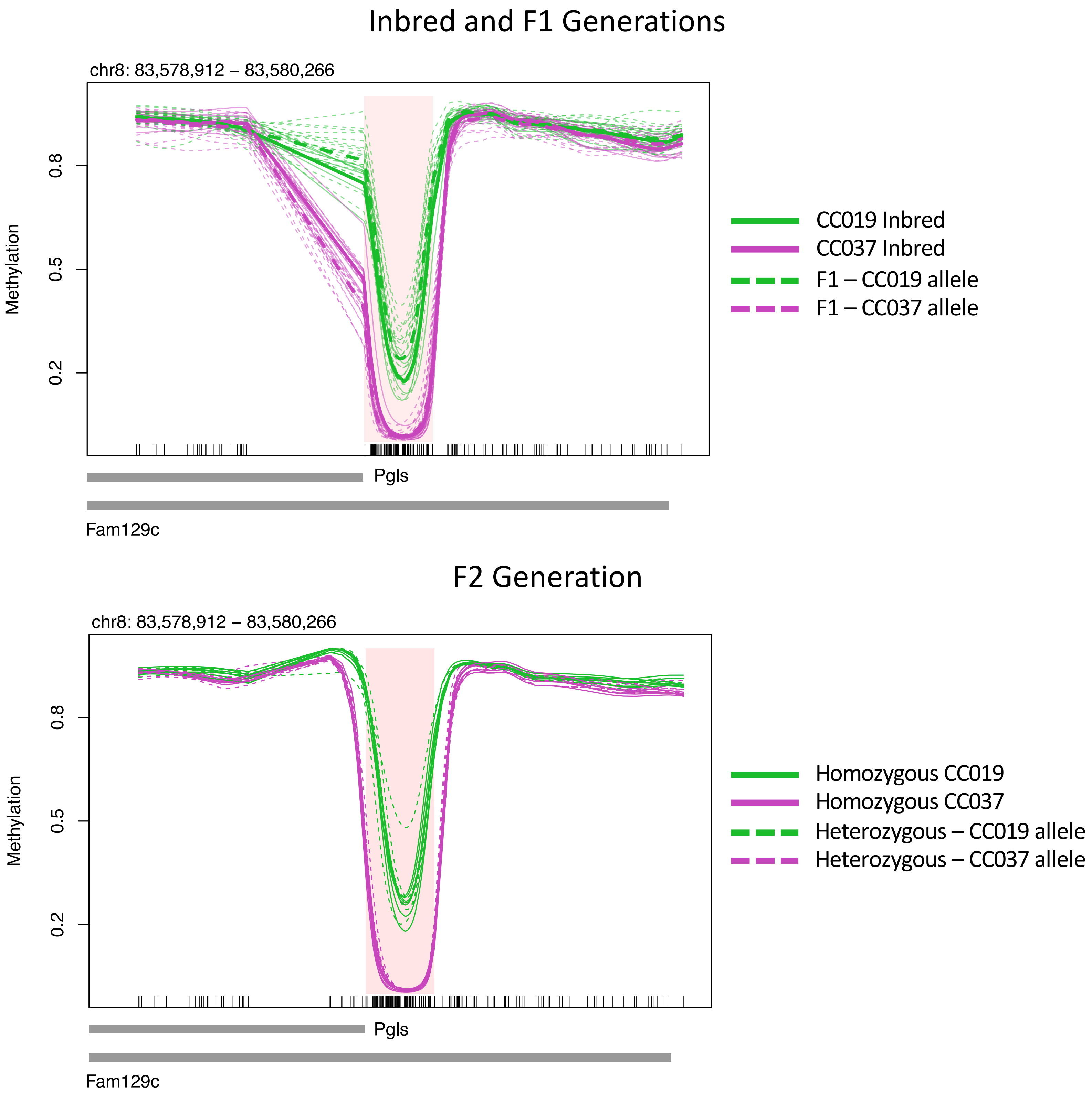

Cis-acting meQTL

Inbred and F1 Generations

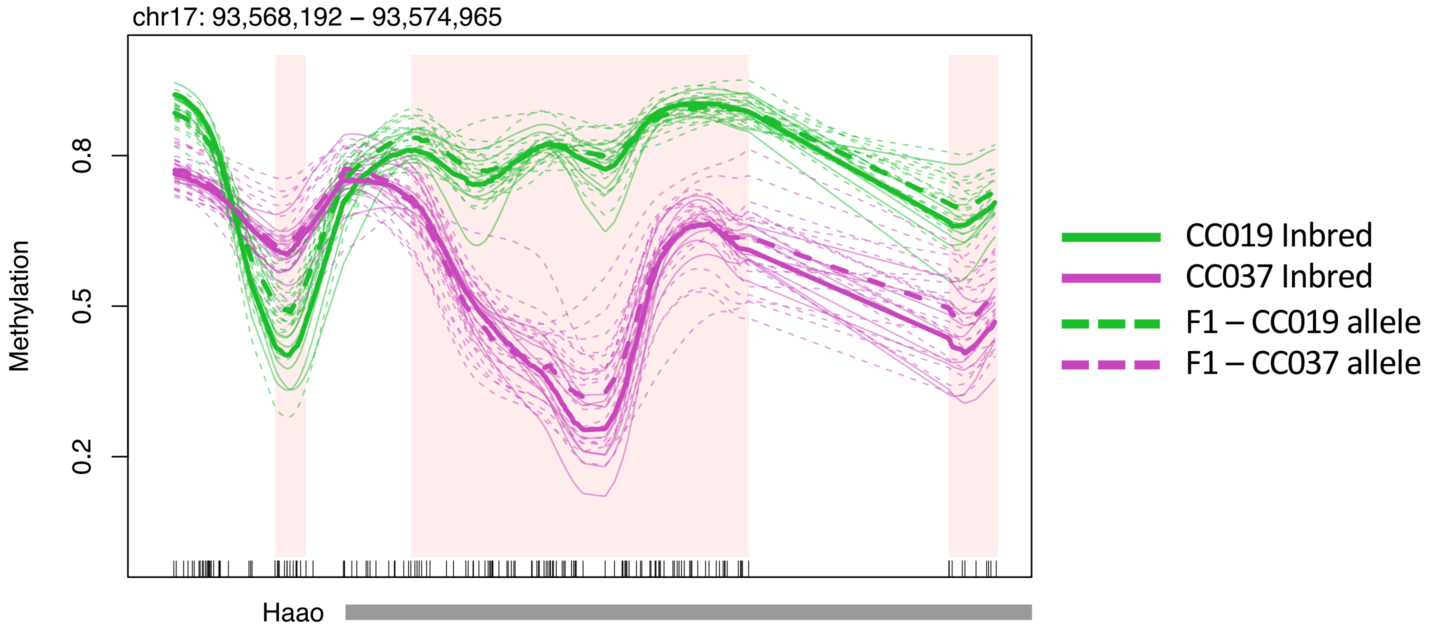

F2 Generation

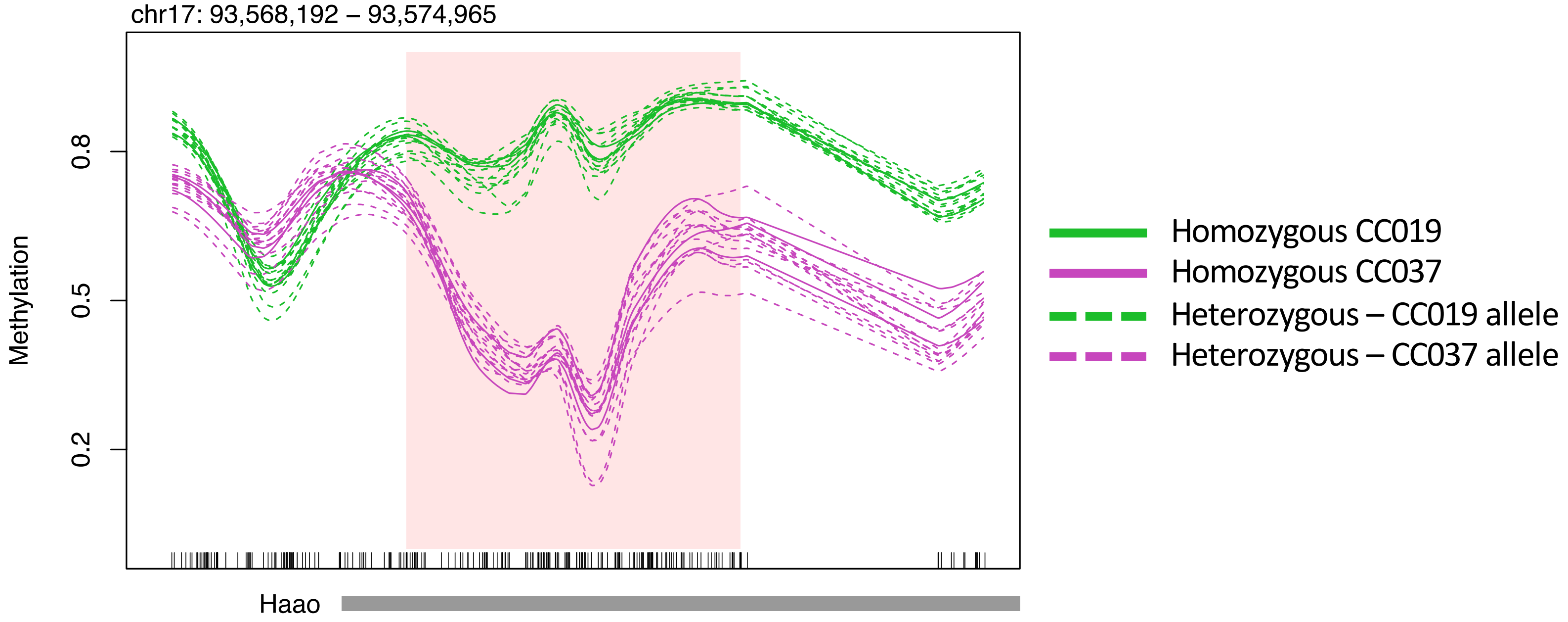

Cis-acting meQTL

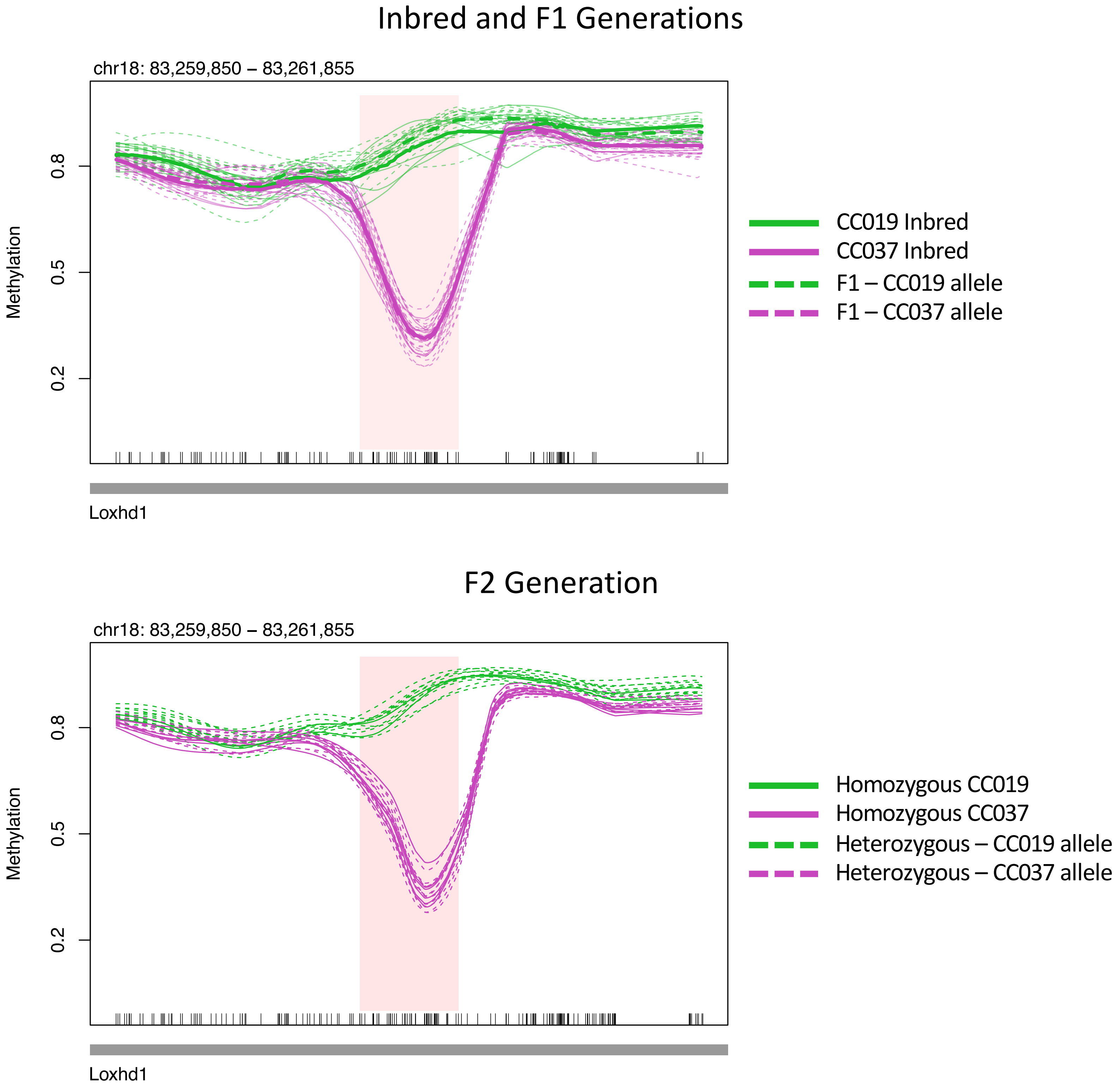

Cis-acting meQTL

Inbred and F1 Generations

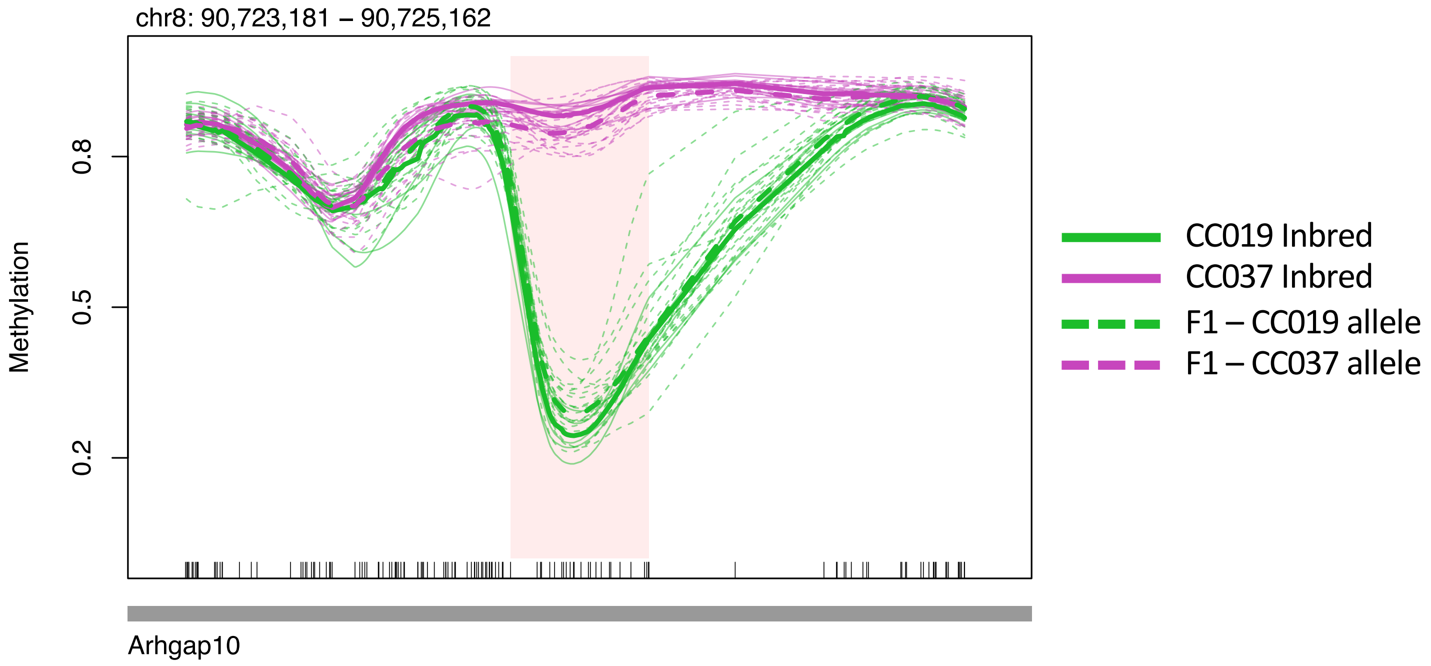

F2 Generation

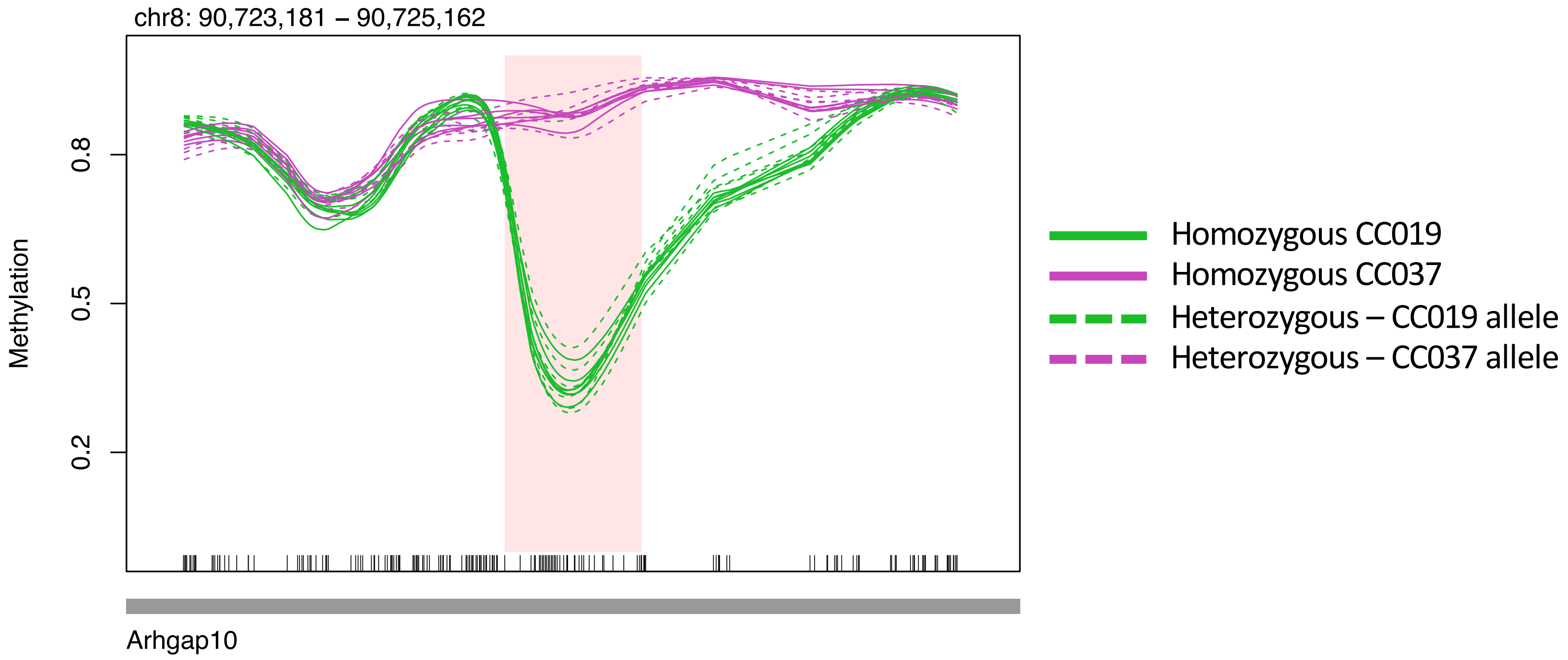

Cis-acting meQTL

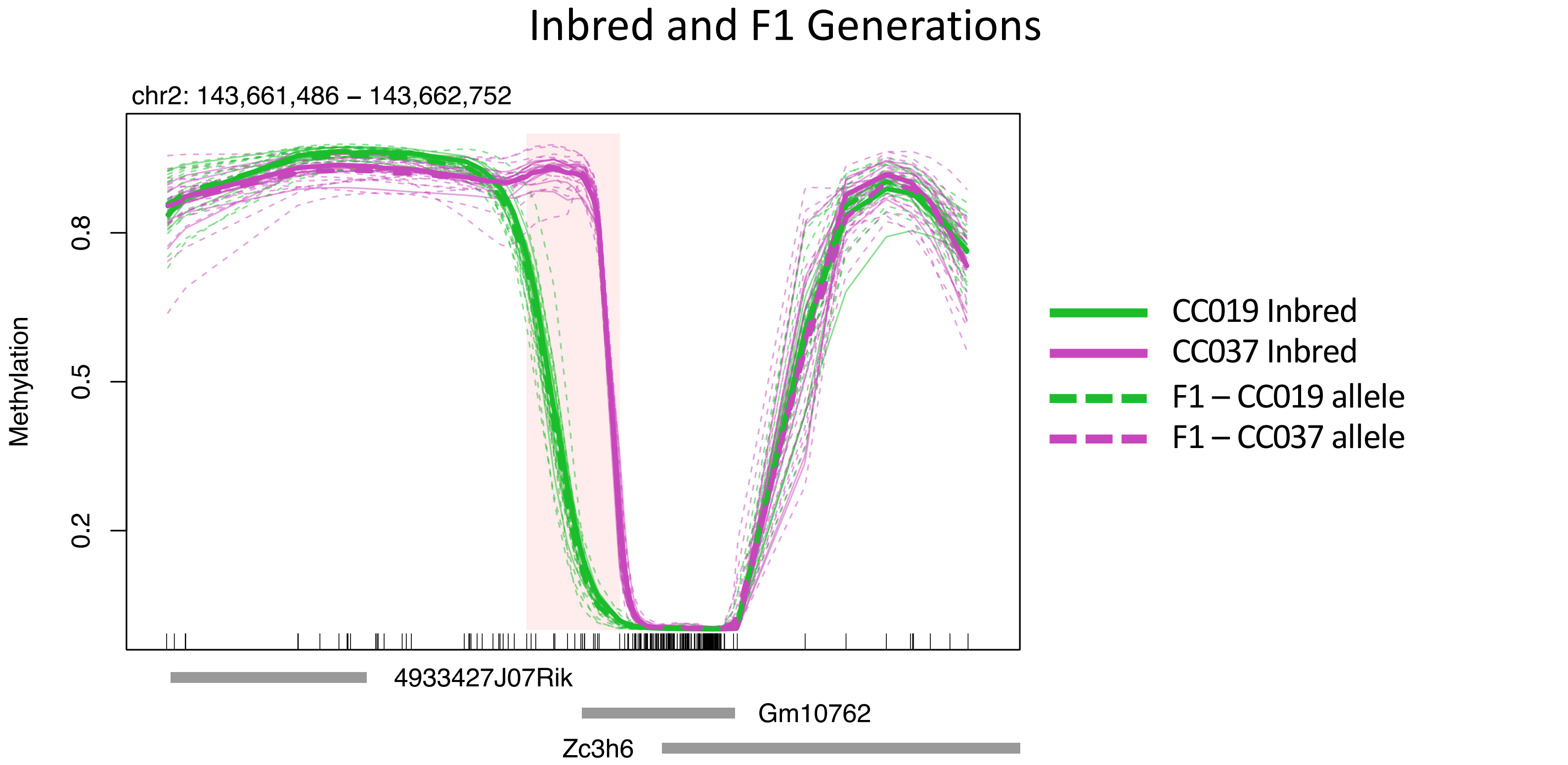

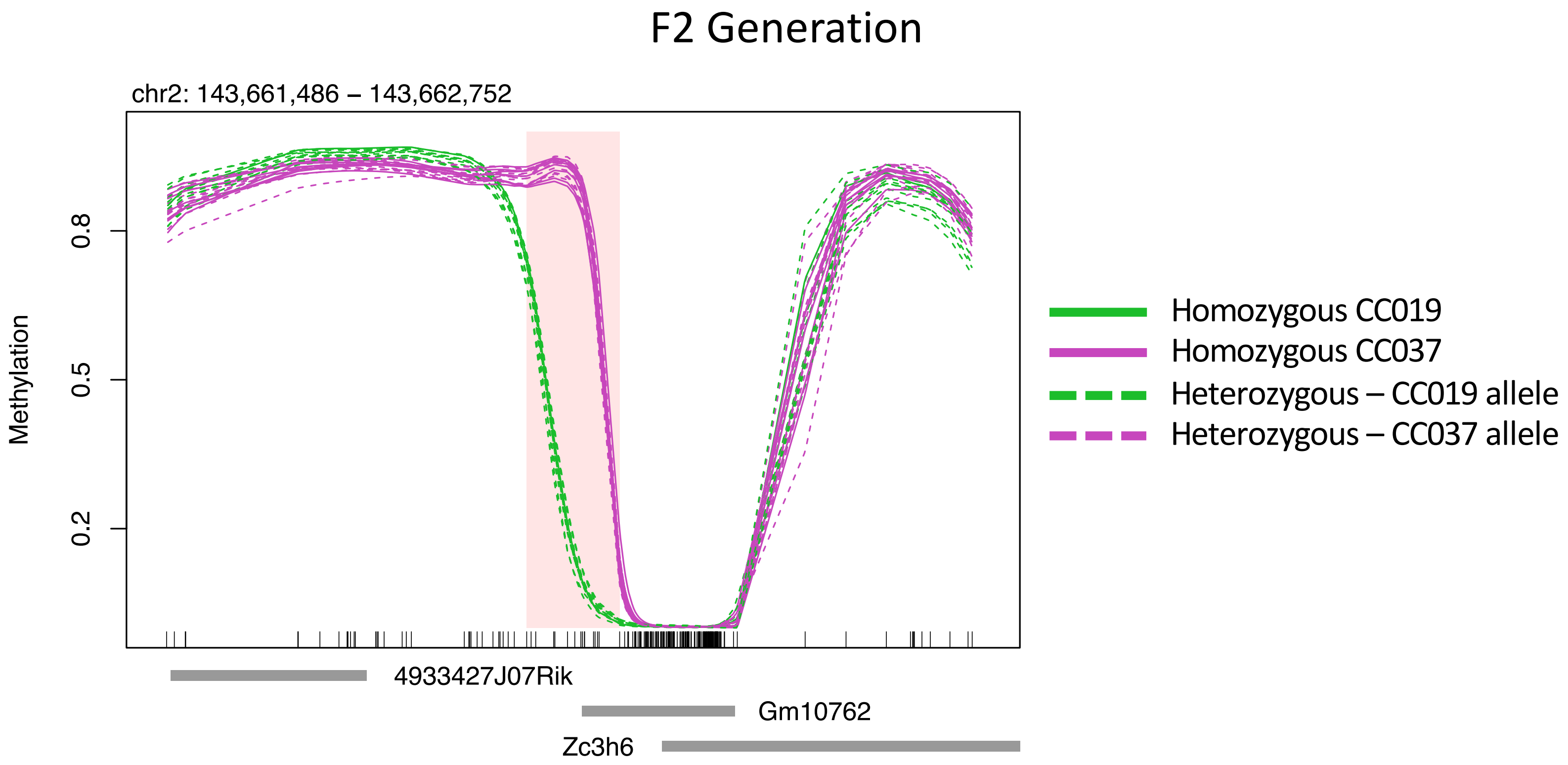

Cis-acting meQTL

Inbred and F1 Generations

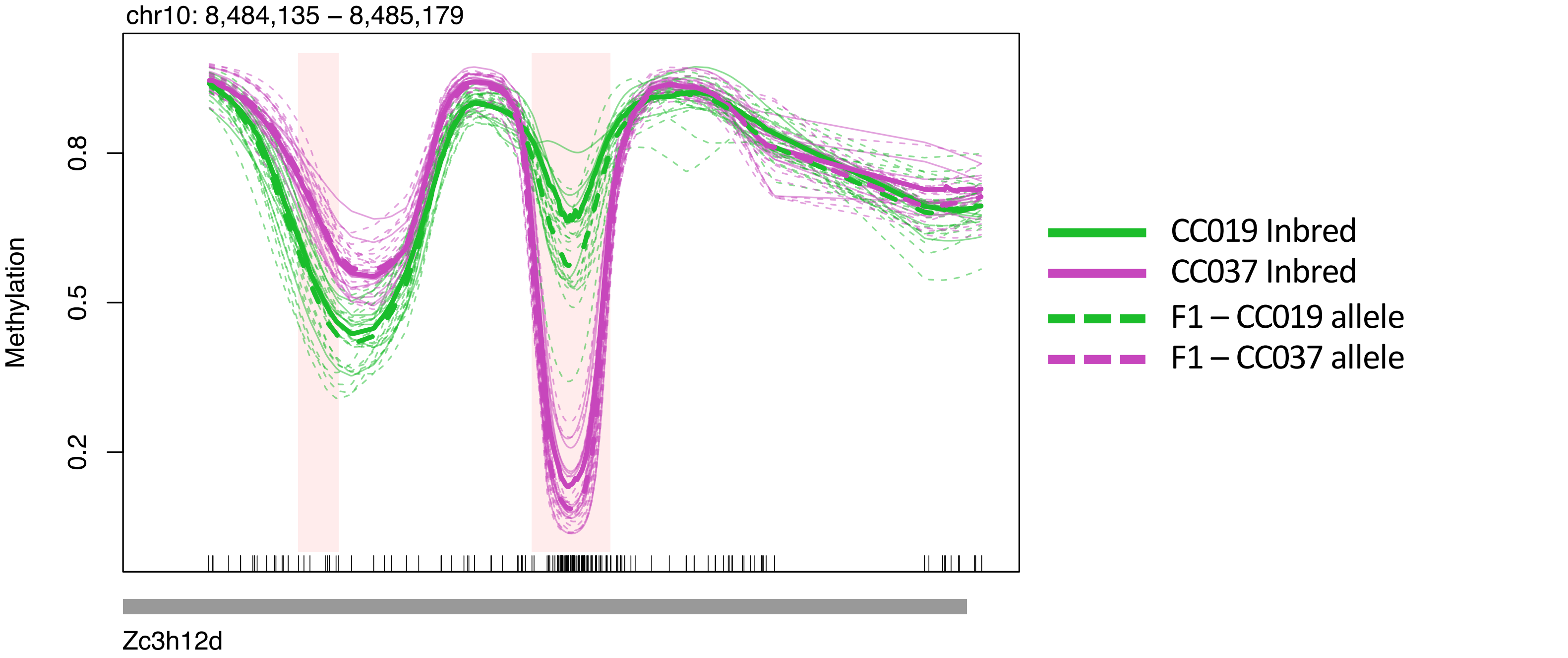

F2 Generation

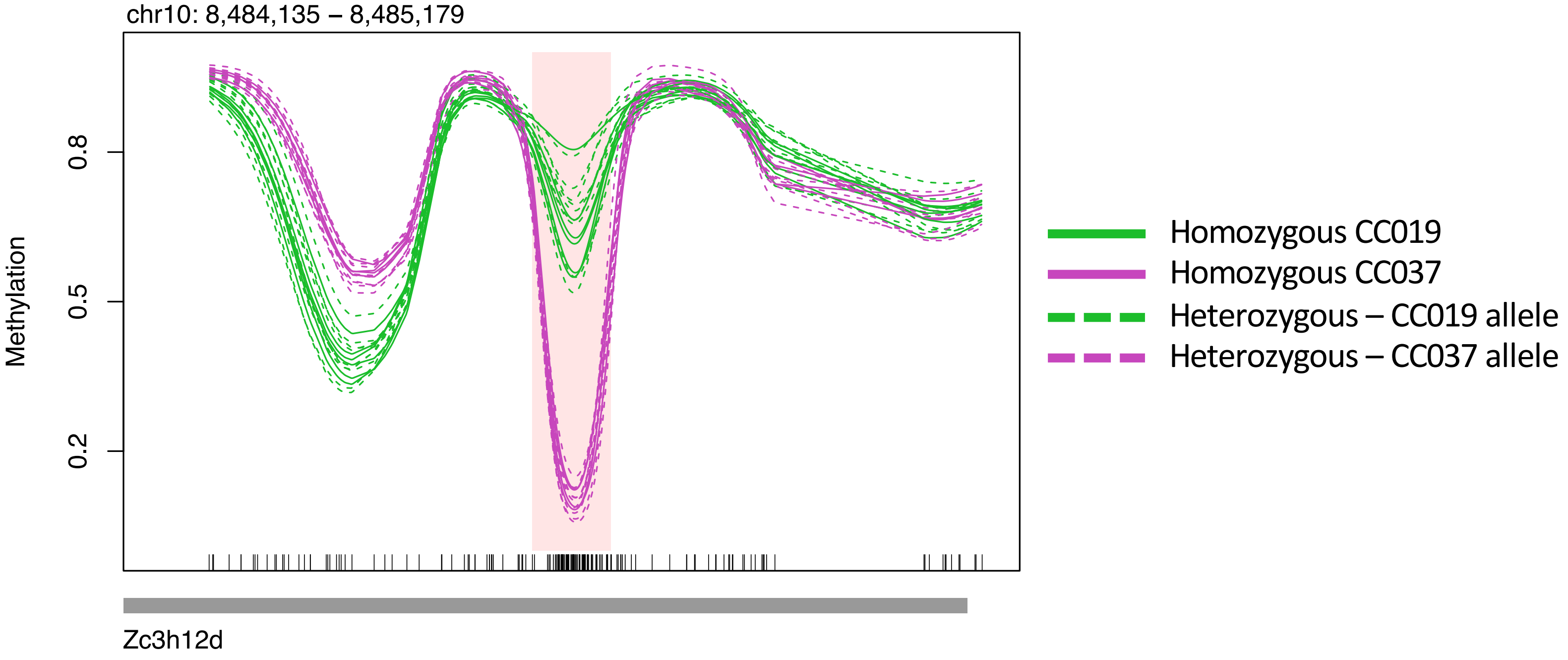

Cis-acting meQTL

Inbred and F1 Generations

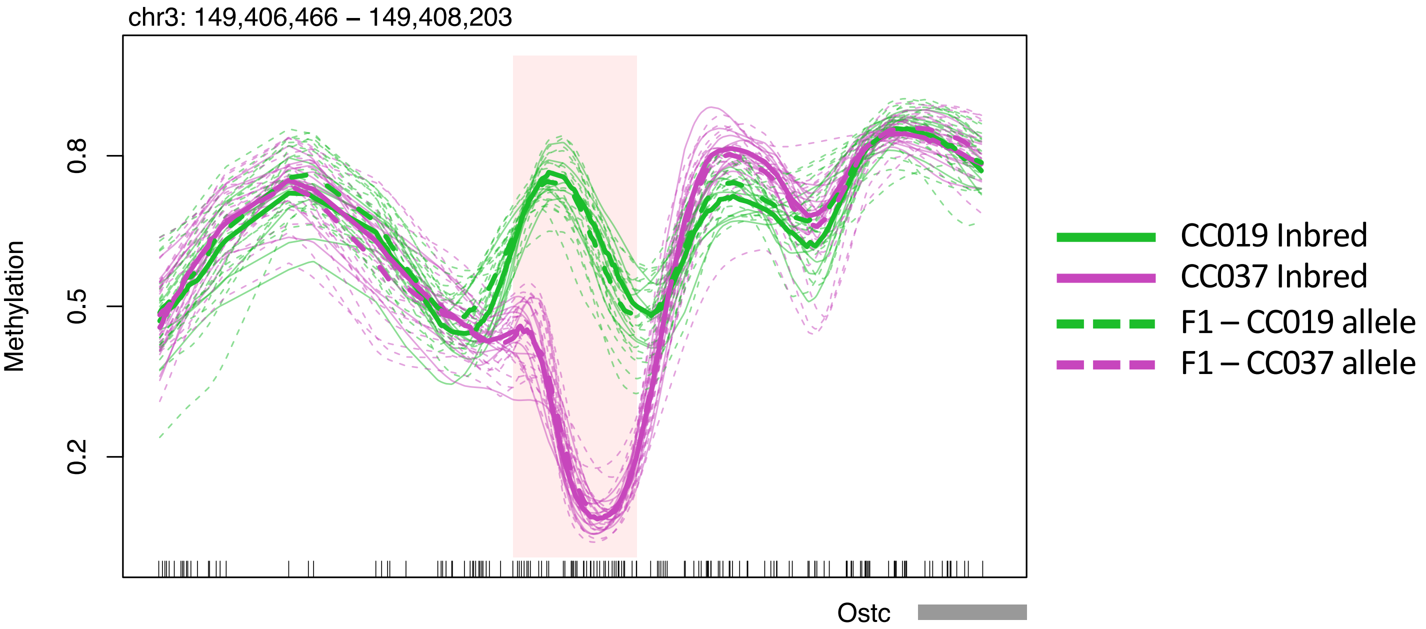

F2 Generation

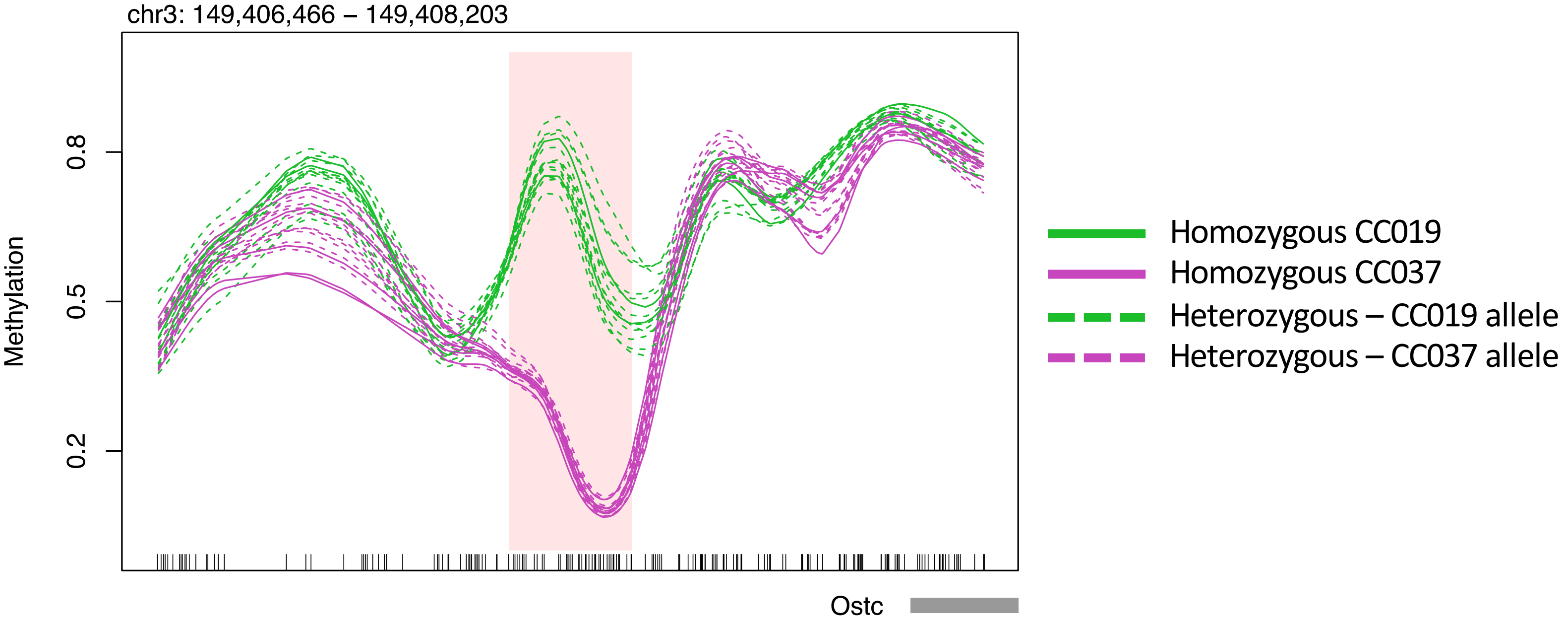

Cis-acting meQTL

Inbred and F1 Generations

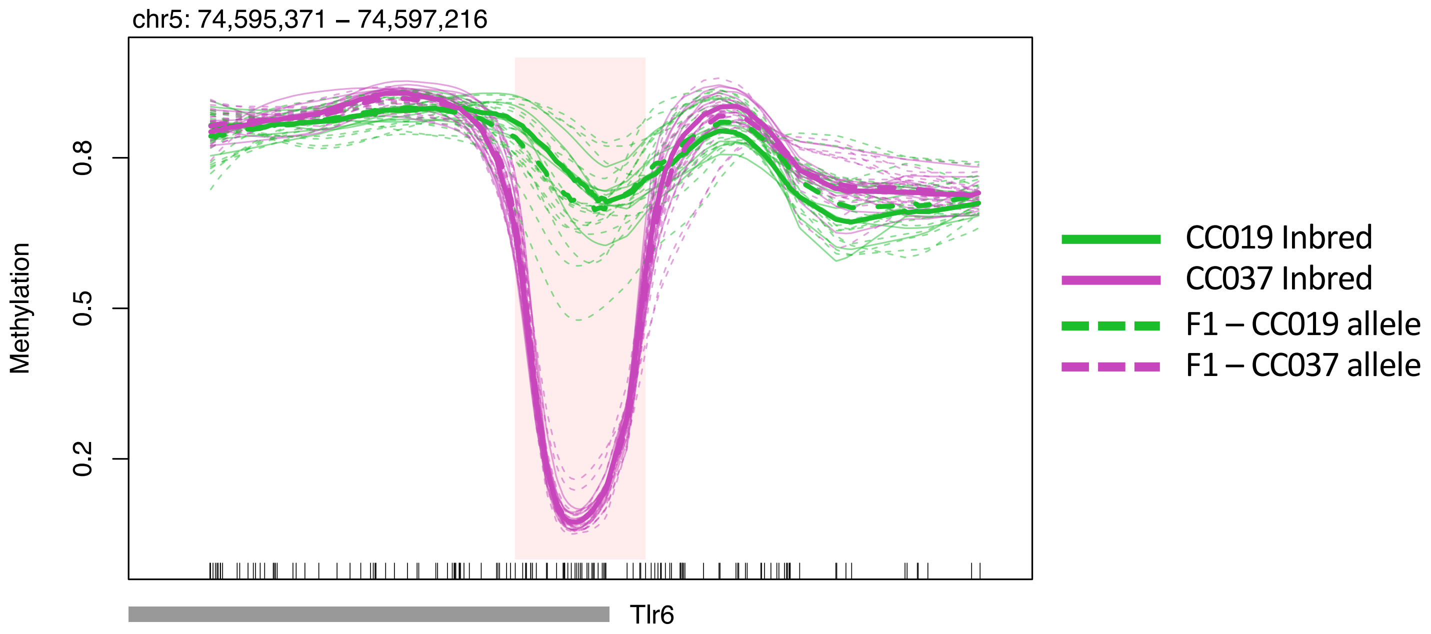

F2 Generation

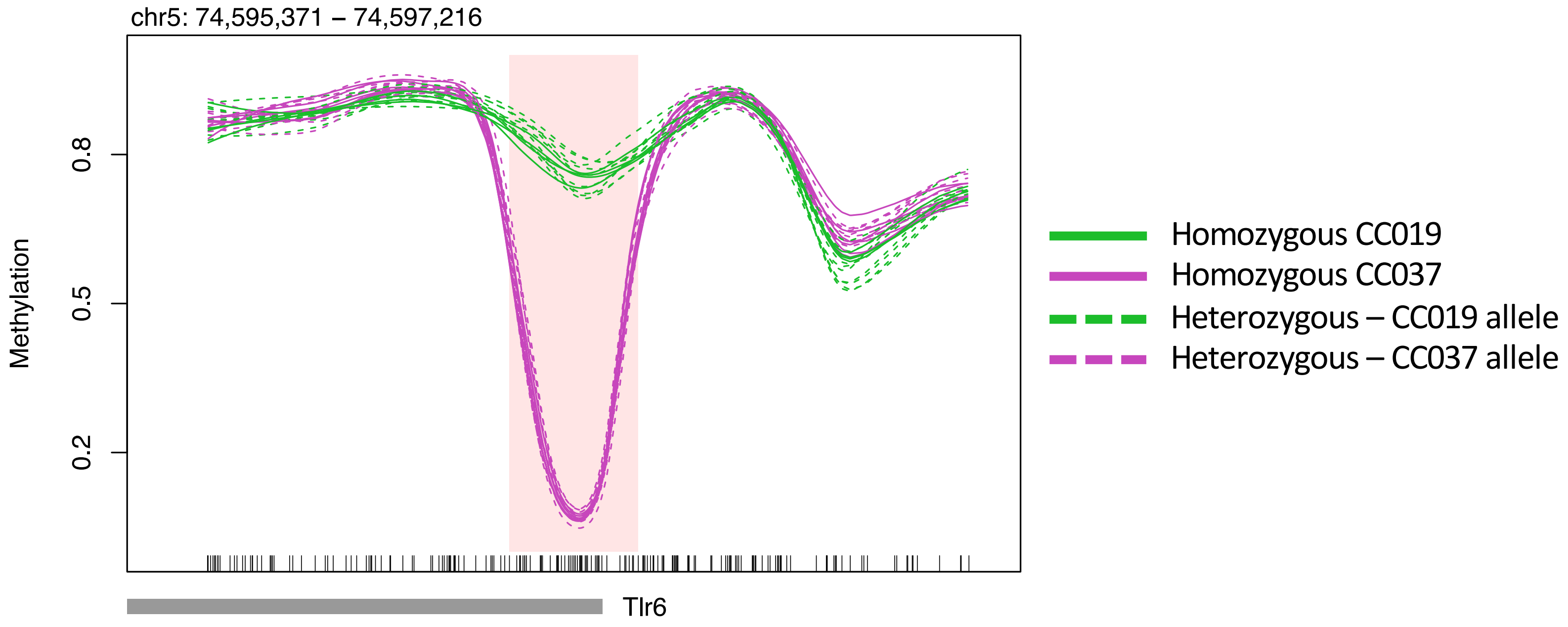

Cis-acting meQTL

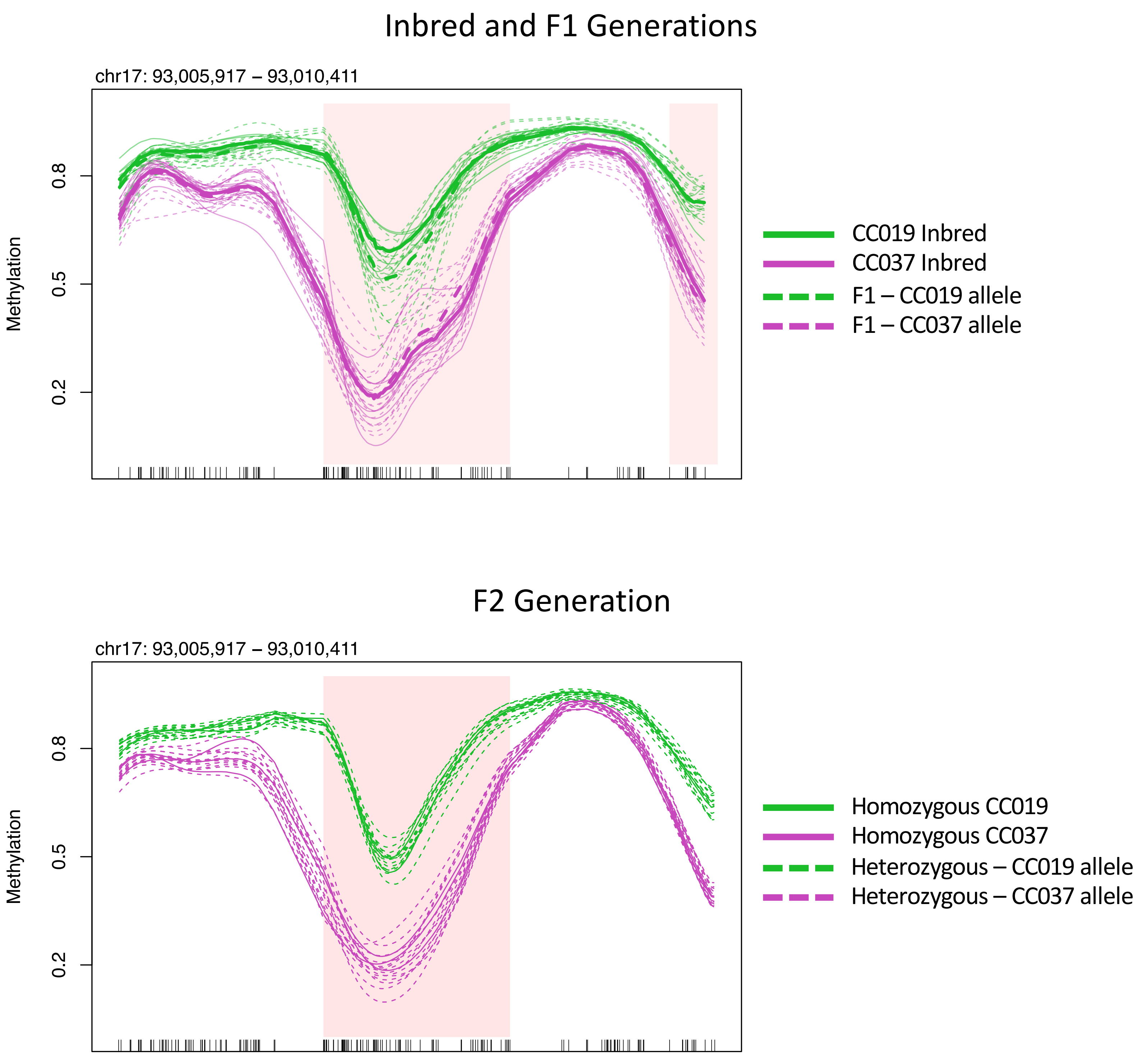

Cis-acting meQTL

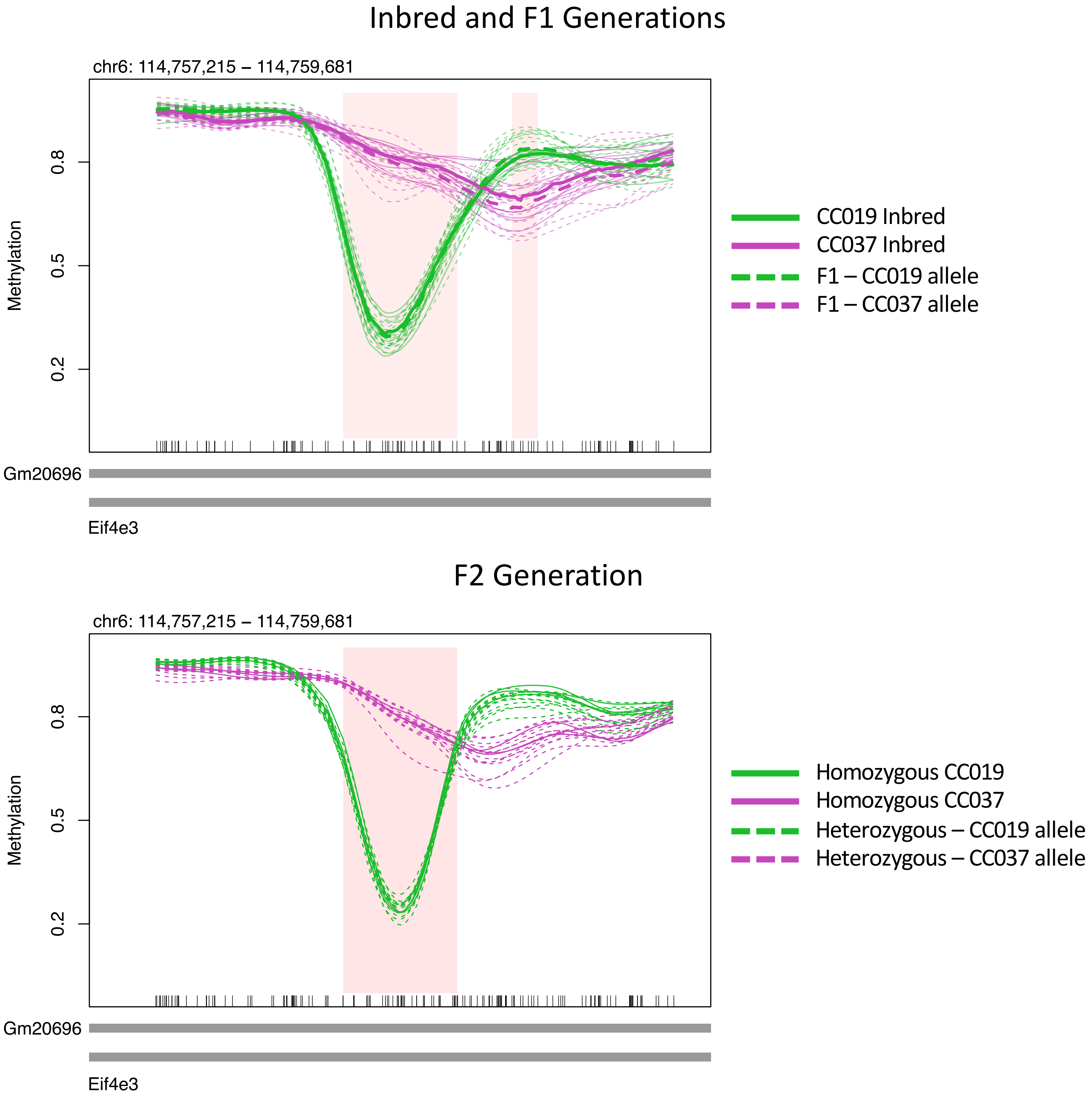

Cis-acting meQTL

Inbred and F1 Generations

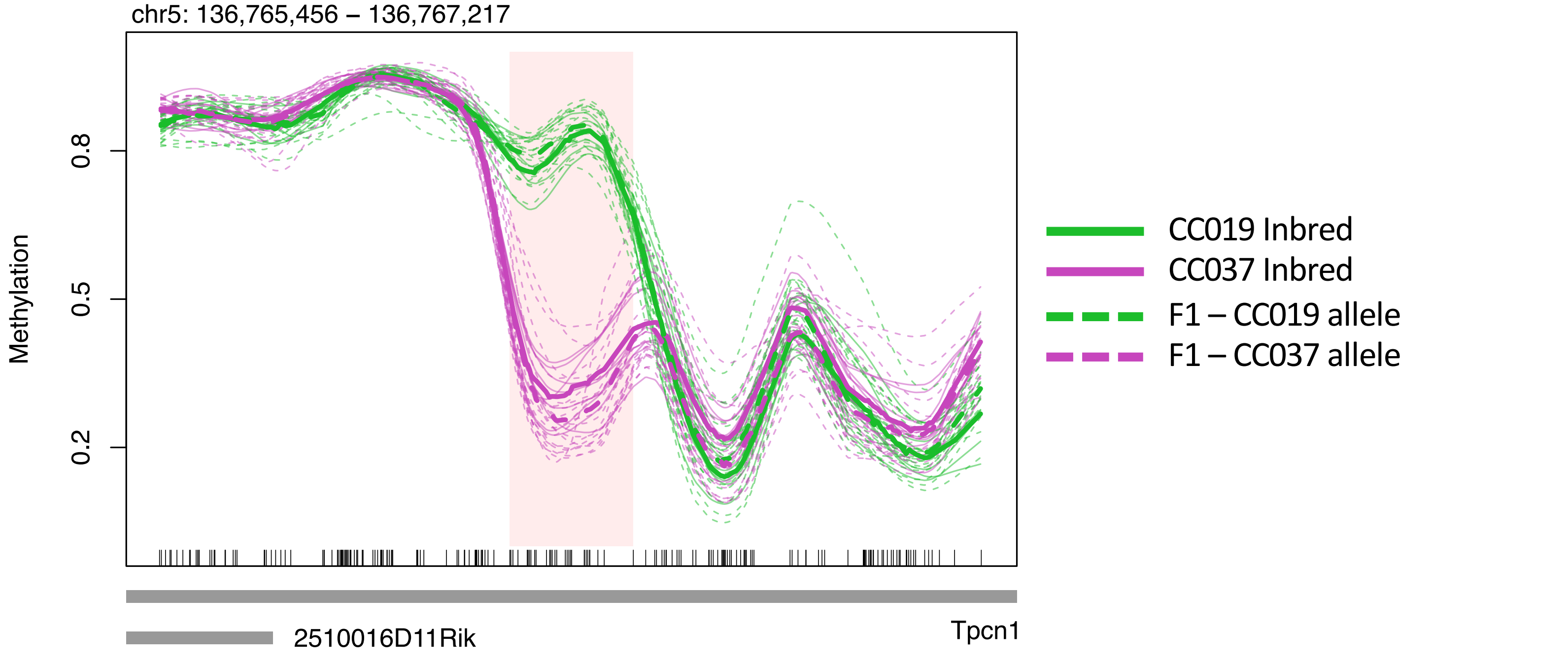

F2 Generation

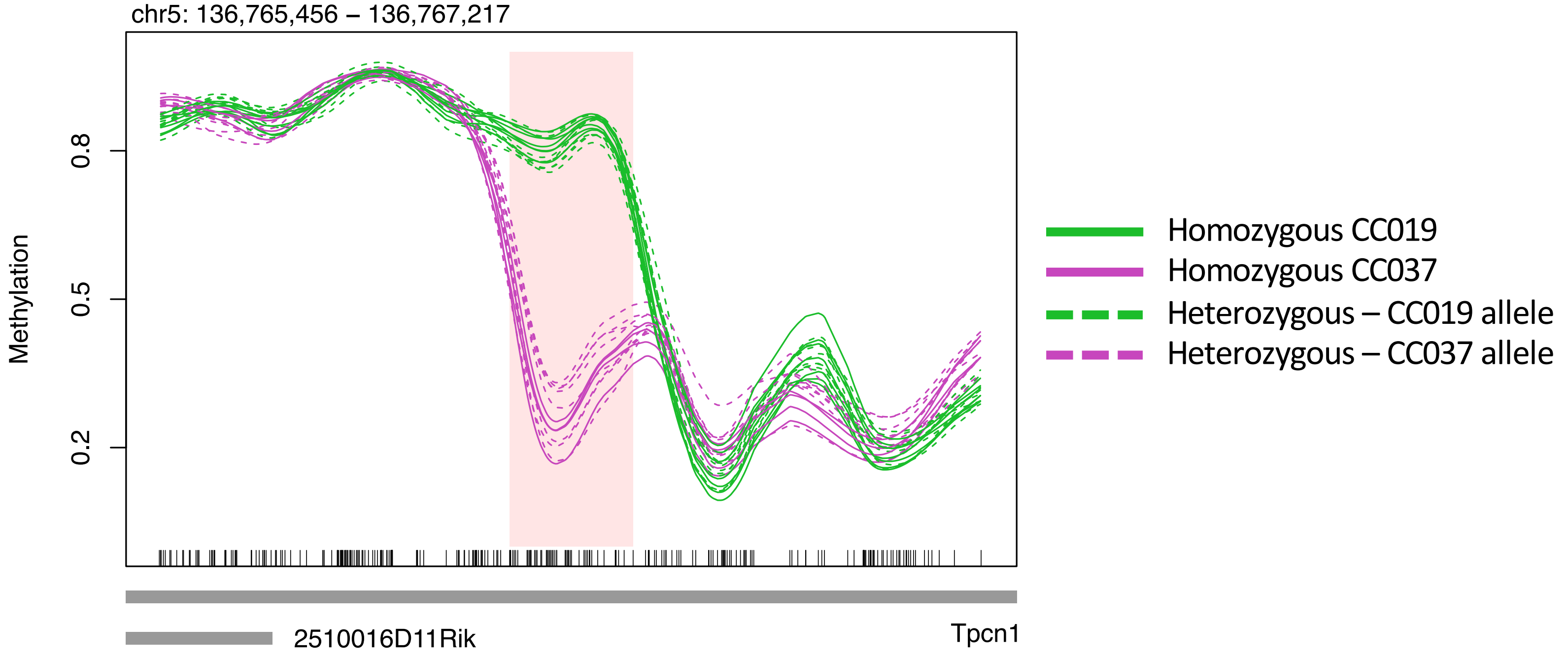

Cis-acting meQTL

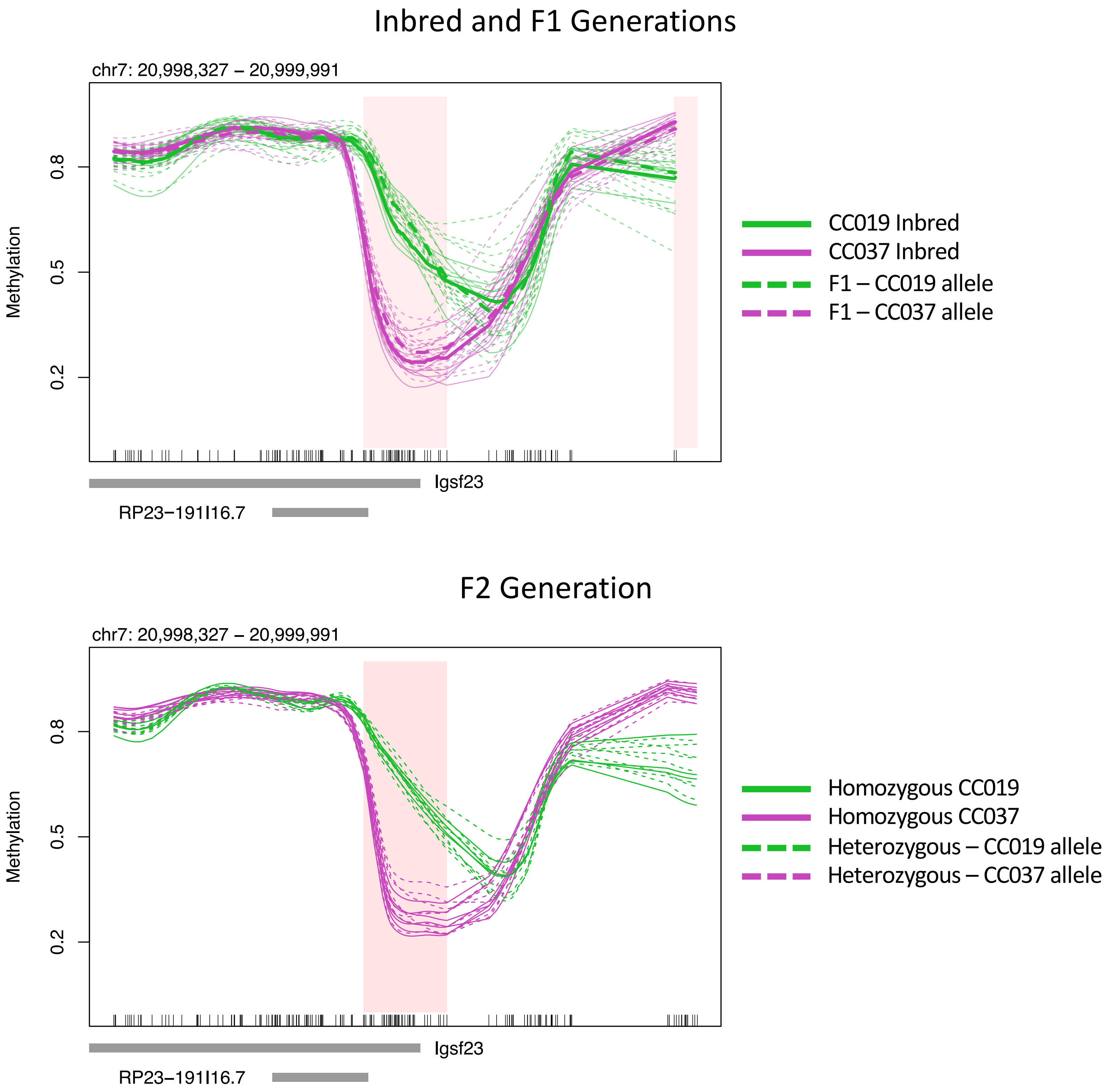

Cis-acting meQTL

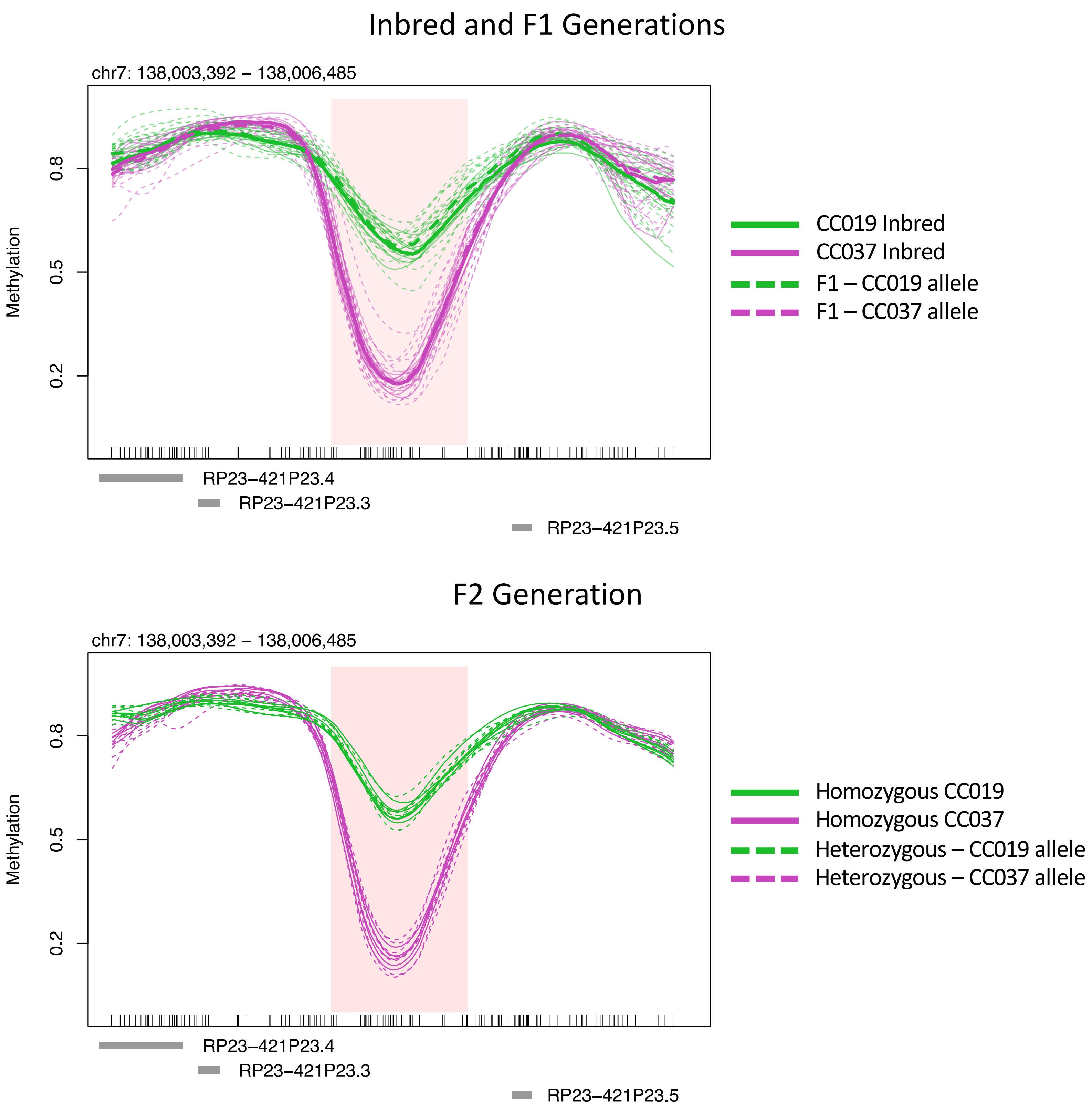

Cis-acting meQTL

Inbred and F1 Generations

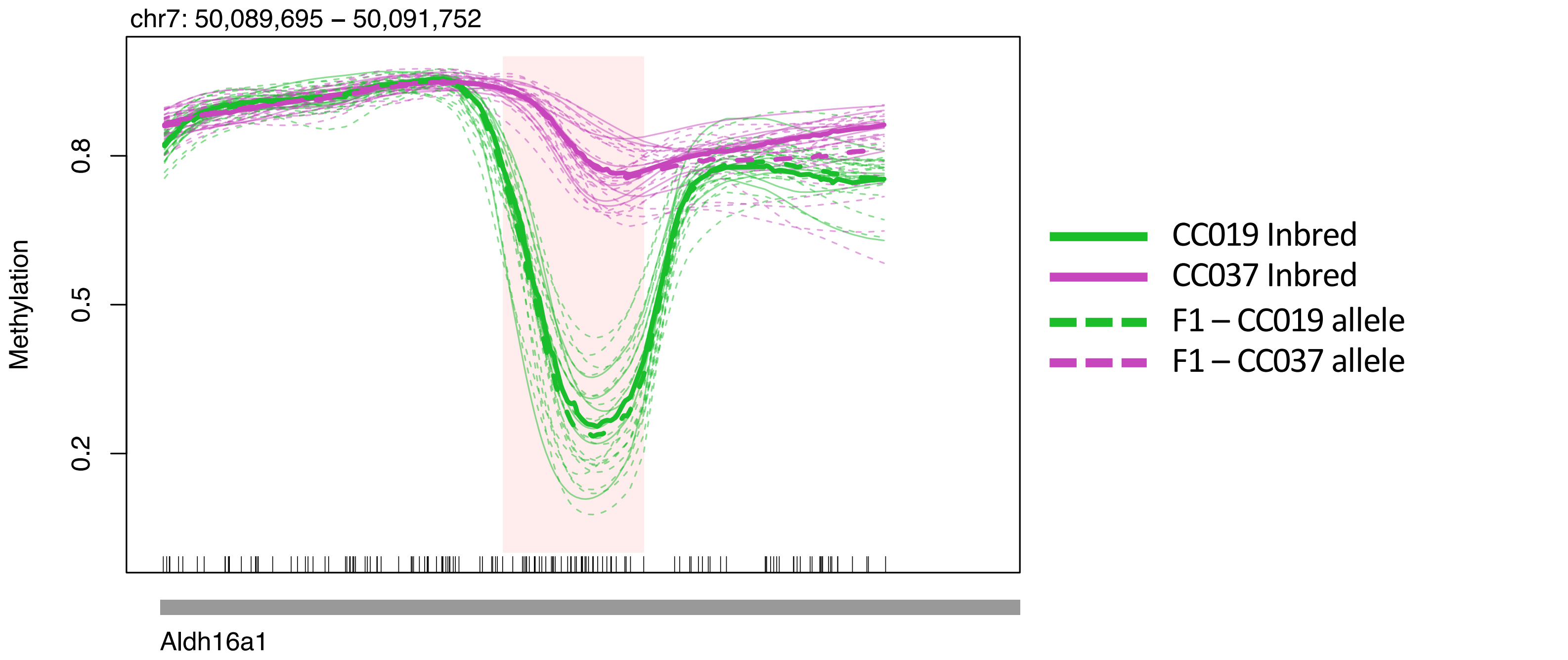

F2 Generation

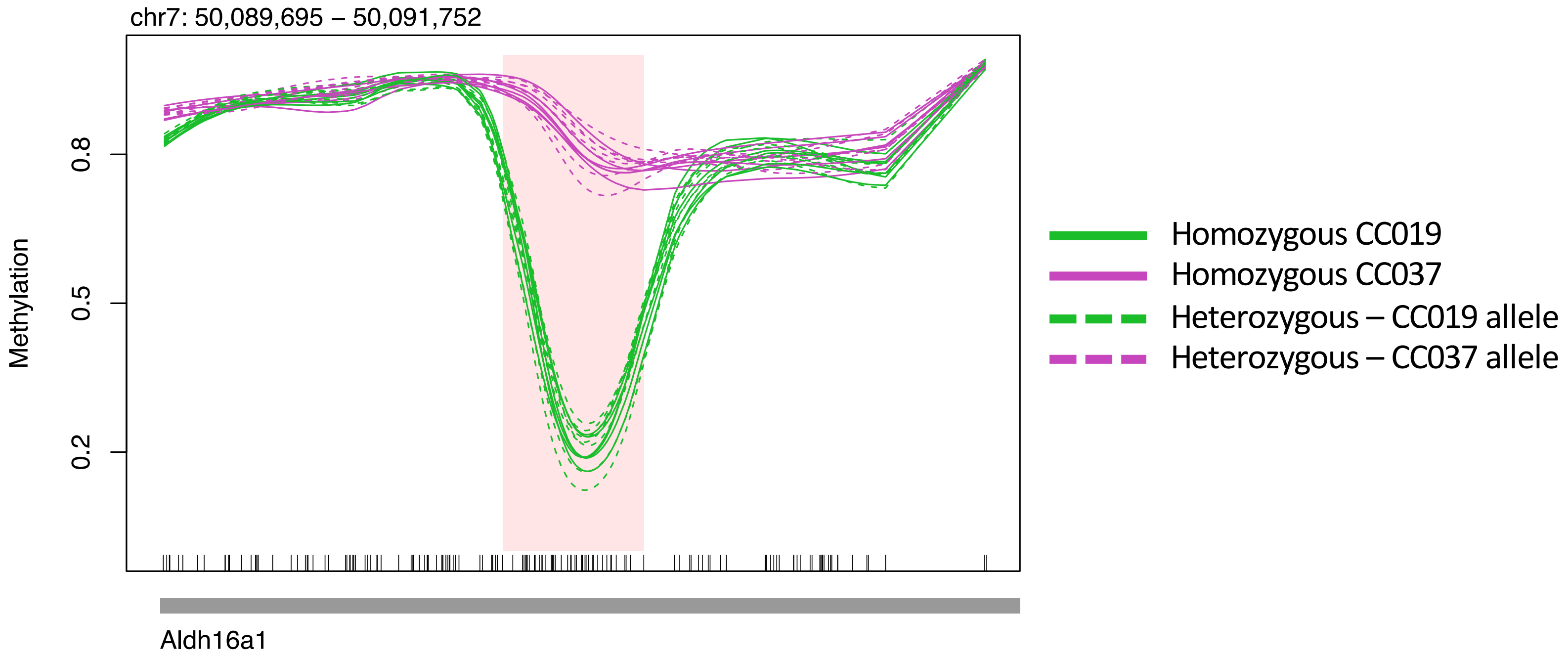

Cis-acting meQTL

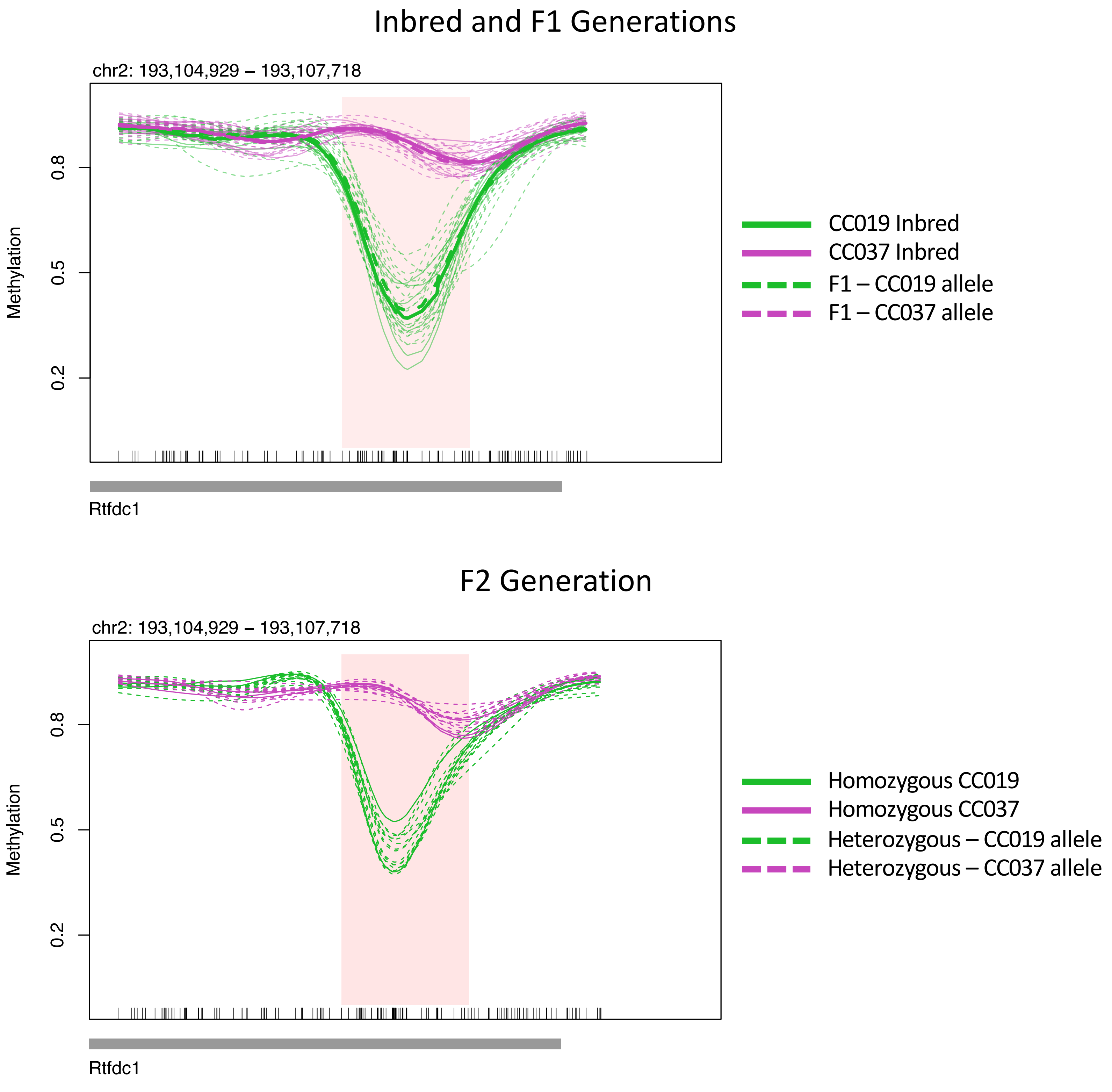

Cis-acting meQTL

Inbred and F1 Generations

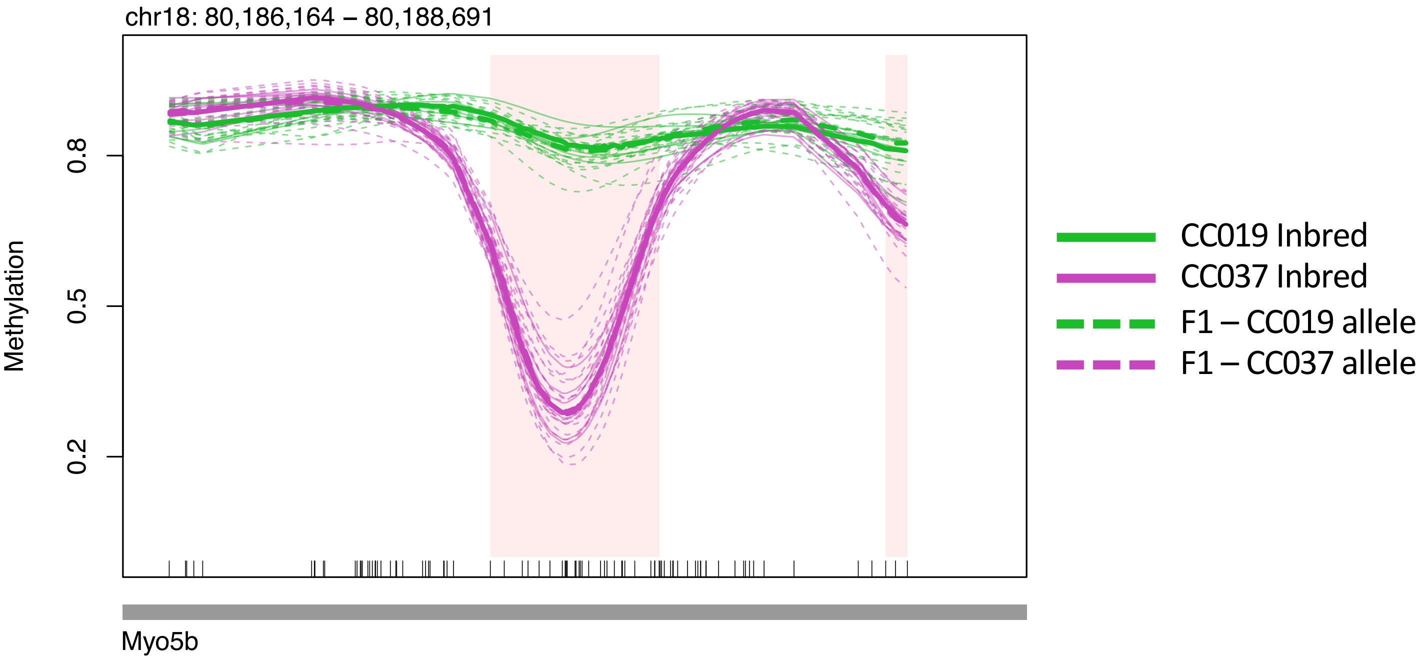

F2 Generation

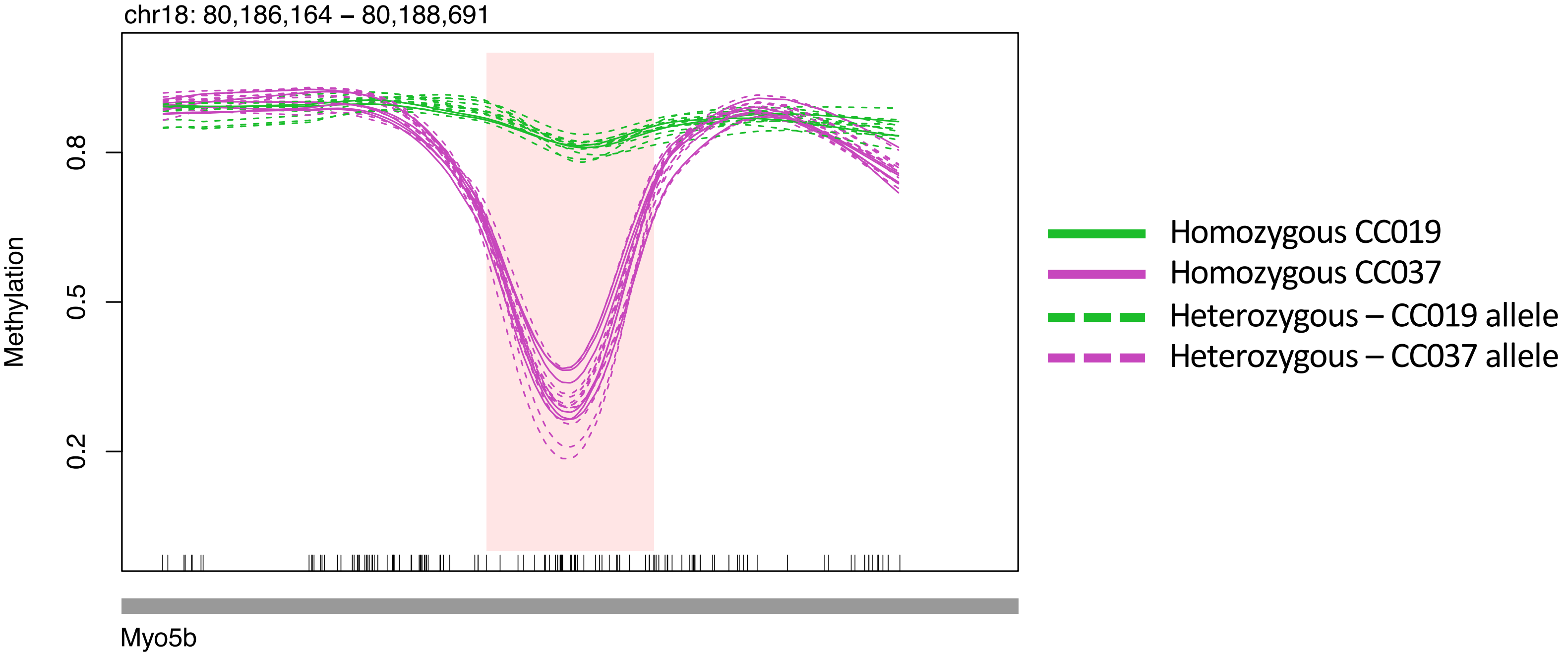

Cis-acting meQTL

Inbred and F1 Generations

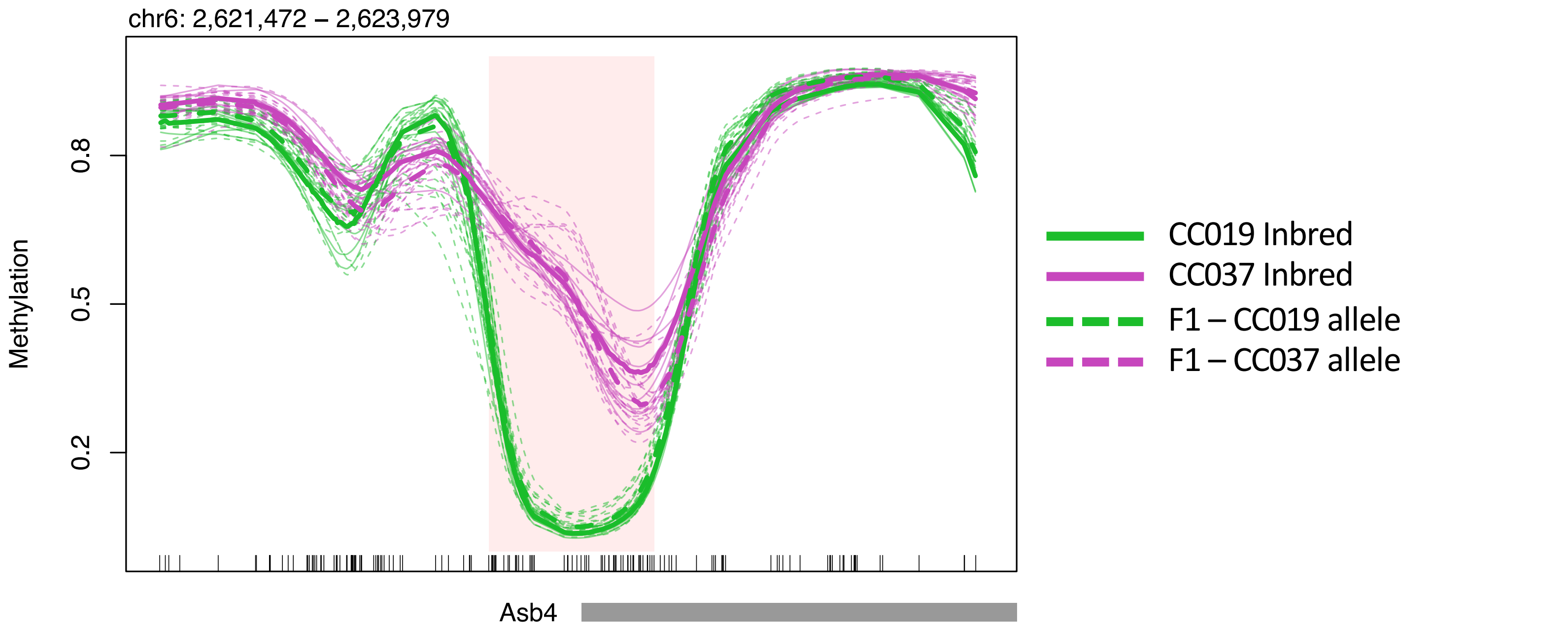

F2 Generation

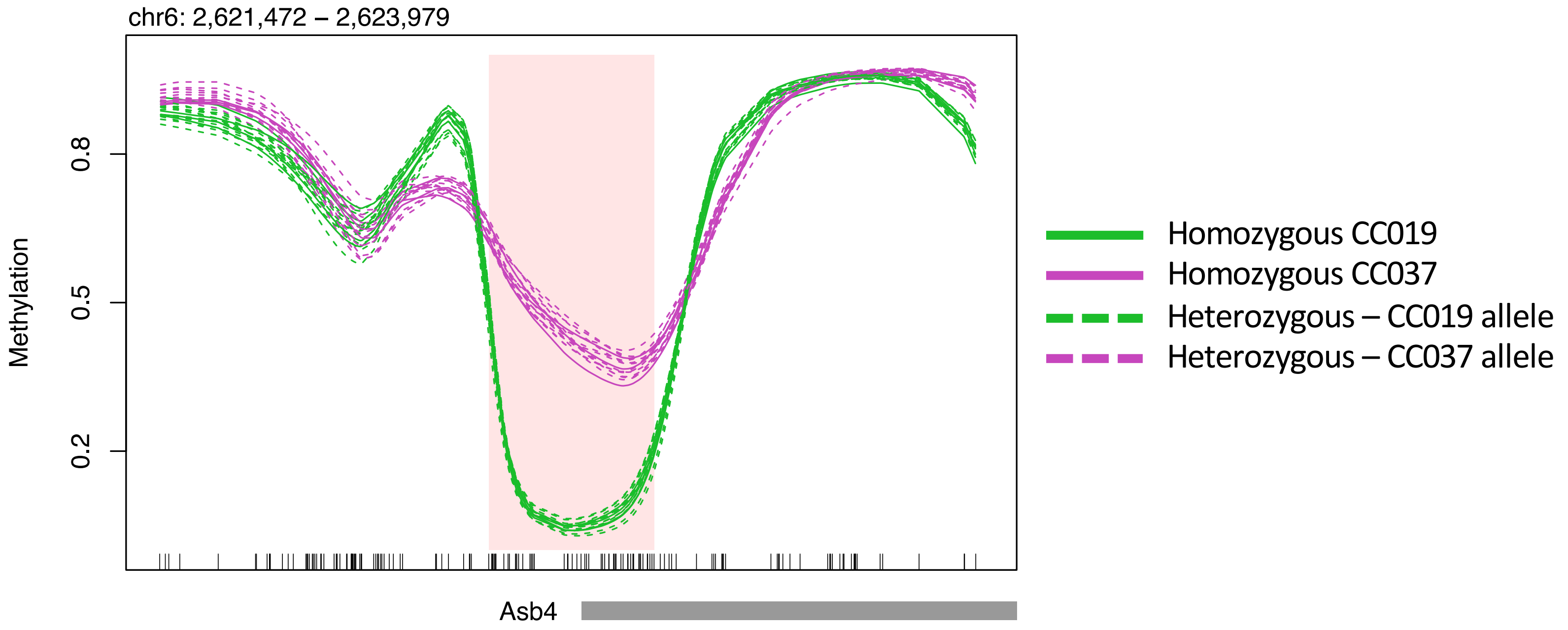

Non-dominant *trans*-acting meQTL

Inbred and F1 Generations

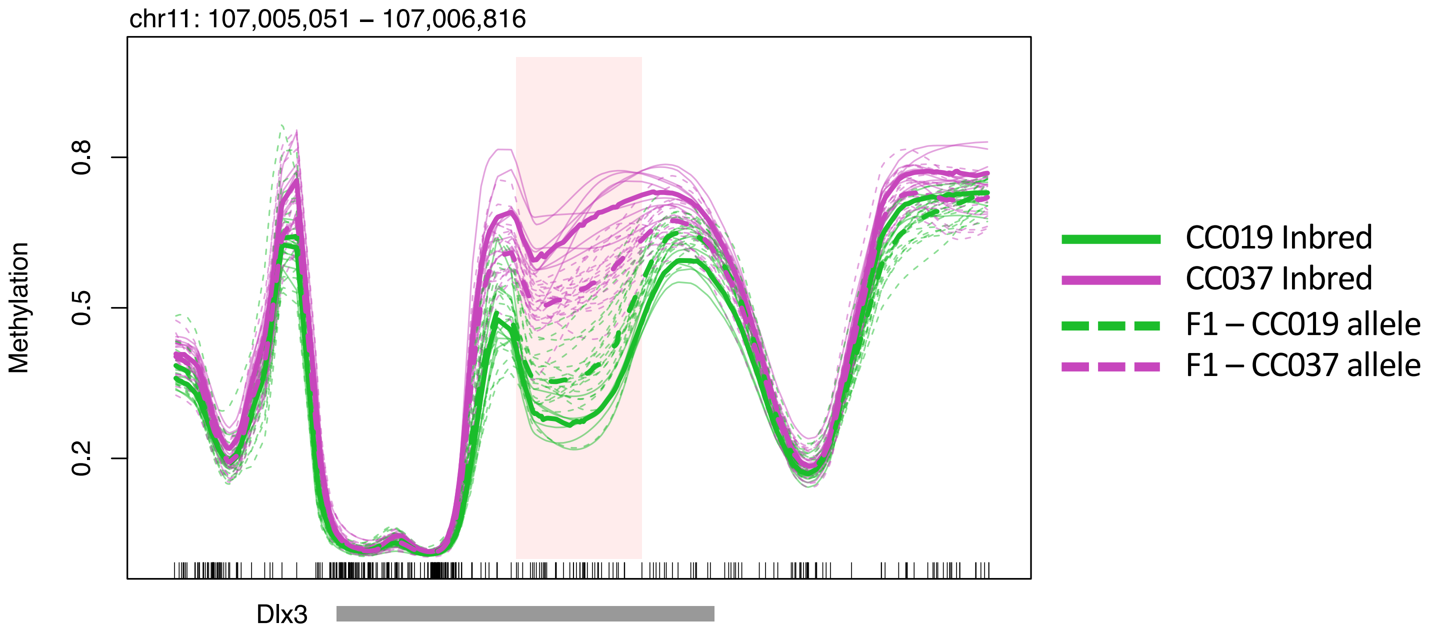

F2 Generation

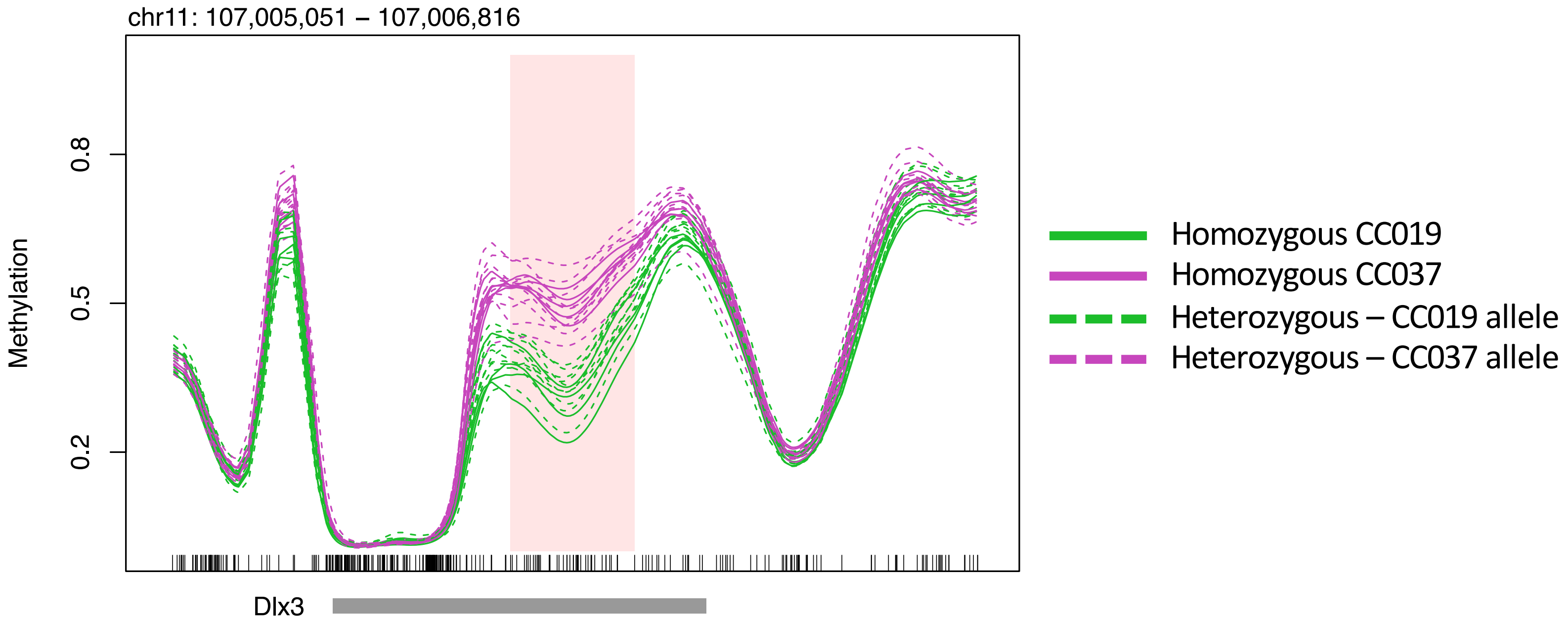

Non-dominant *trans*-acting meQTL

Inbred and F1 Generations

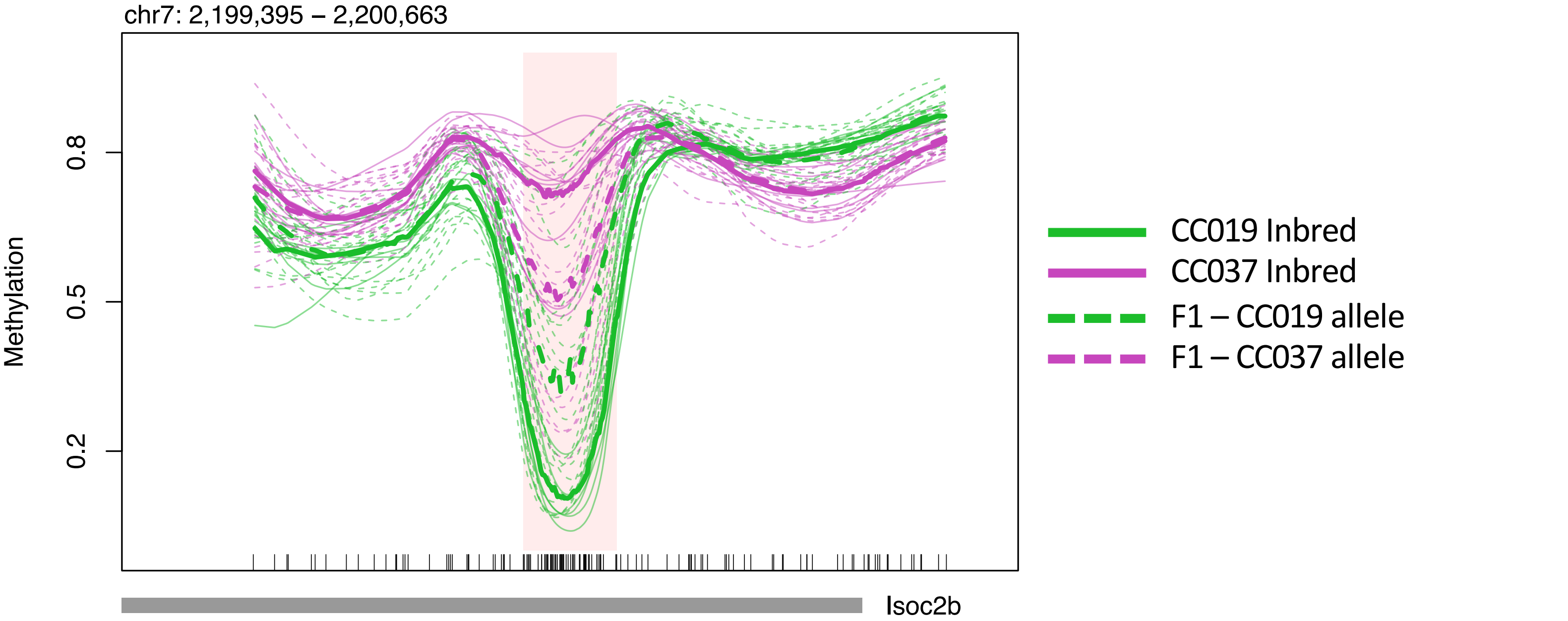

F2 Generation

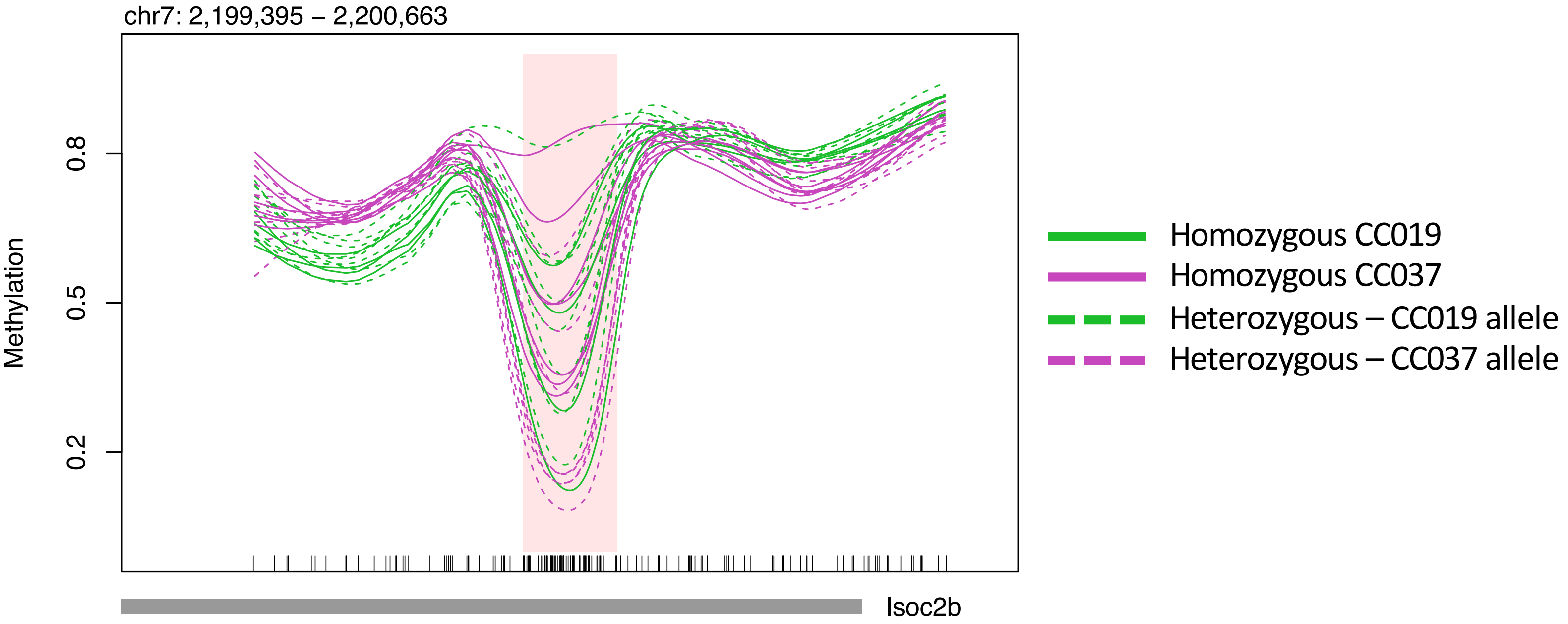

Non-dominant *trans*-acting meQTL

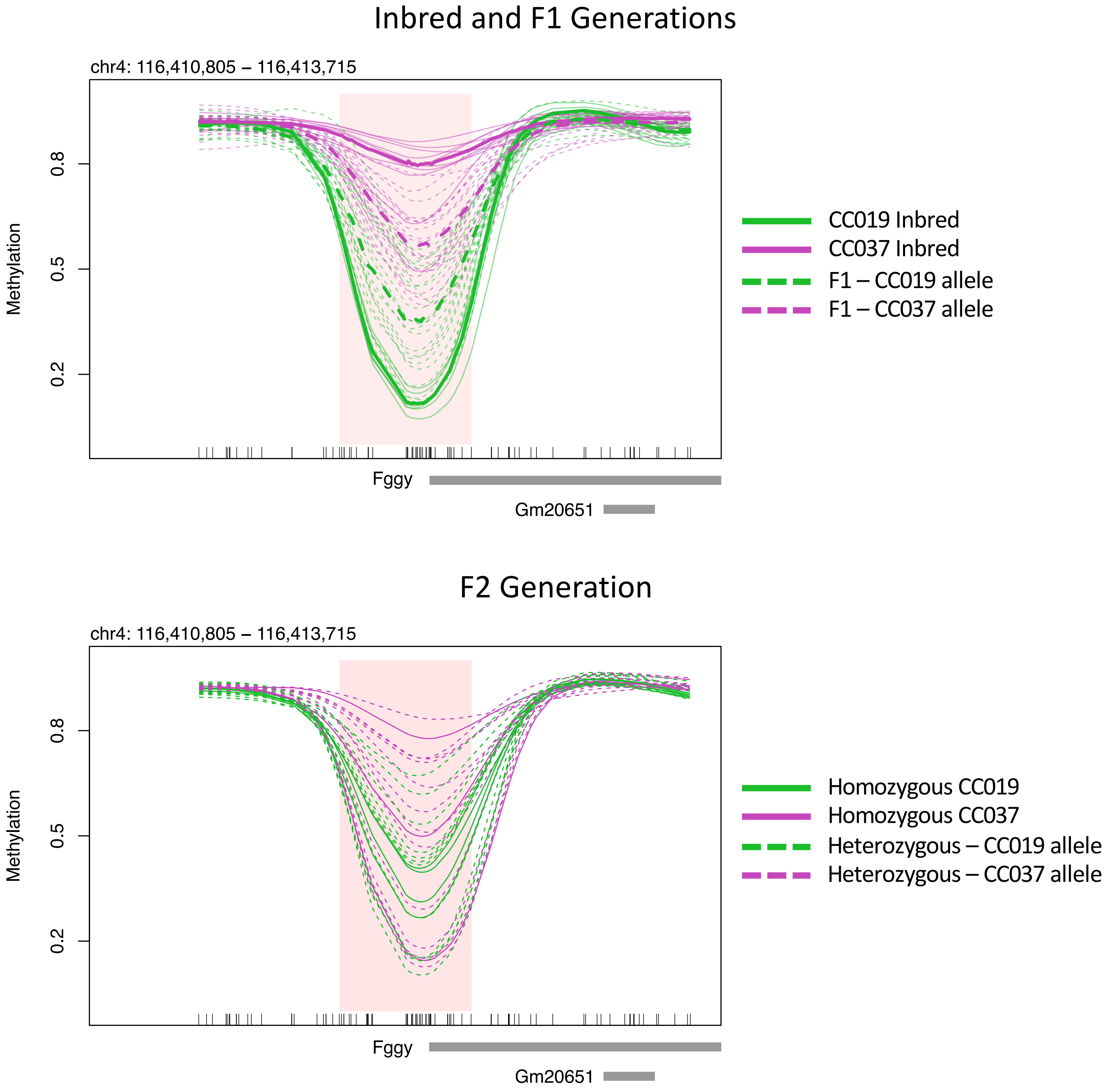

Non-dominant *trans*-acting meQTL

Inbred and F1 Generations

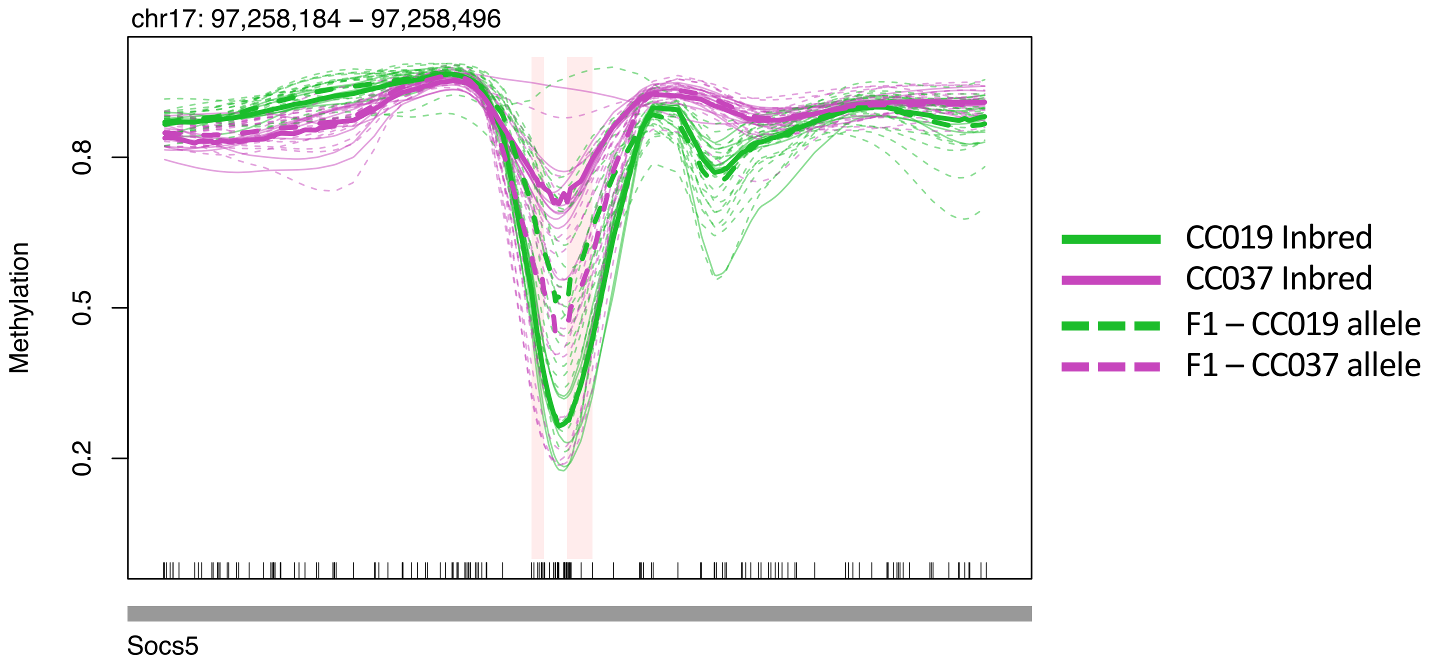

F2 Generation

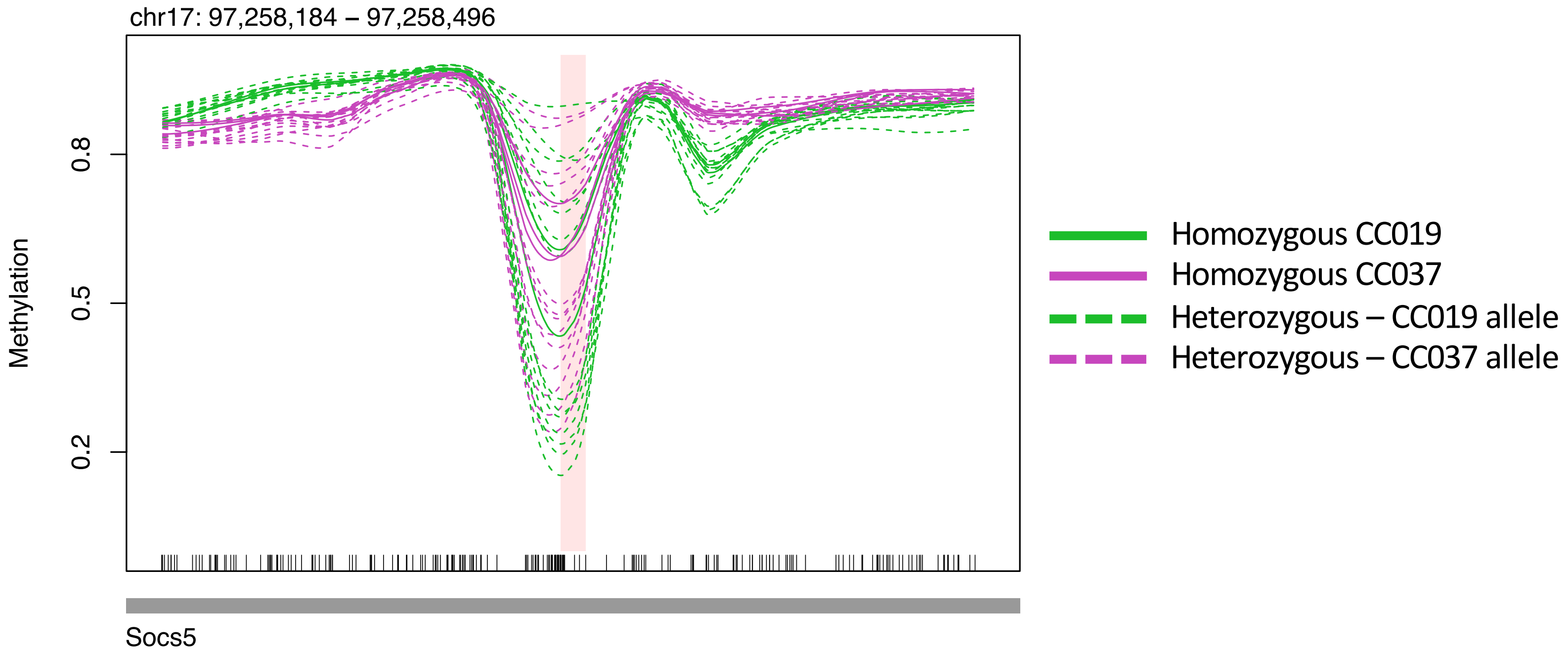

Non-dominant *trans*-acting meQTL

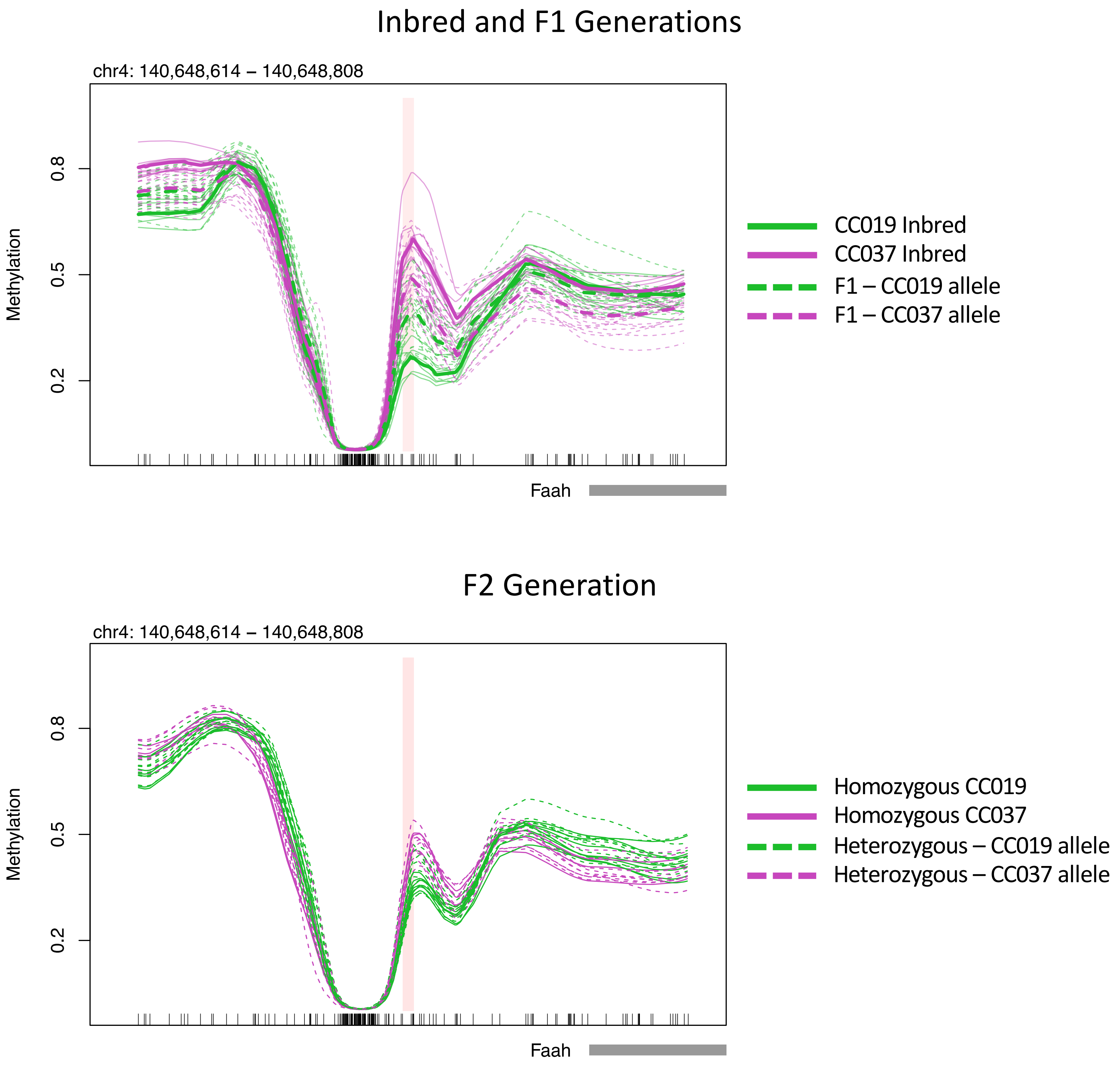

Sex-specific methylation

Inbred and F1 Generations

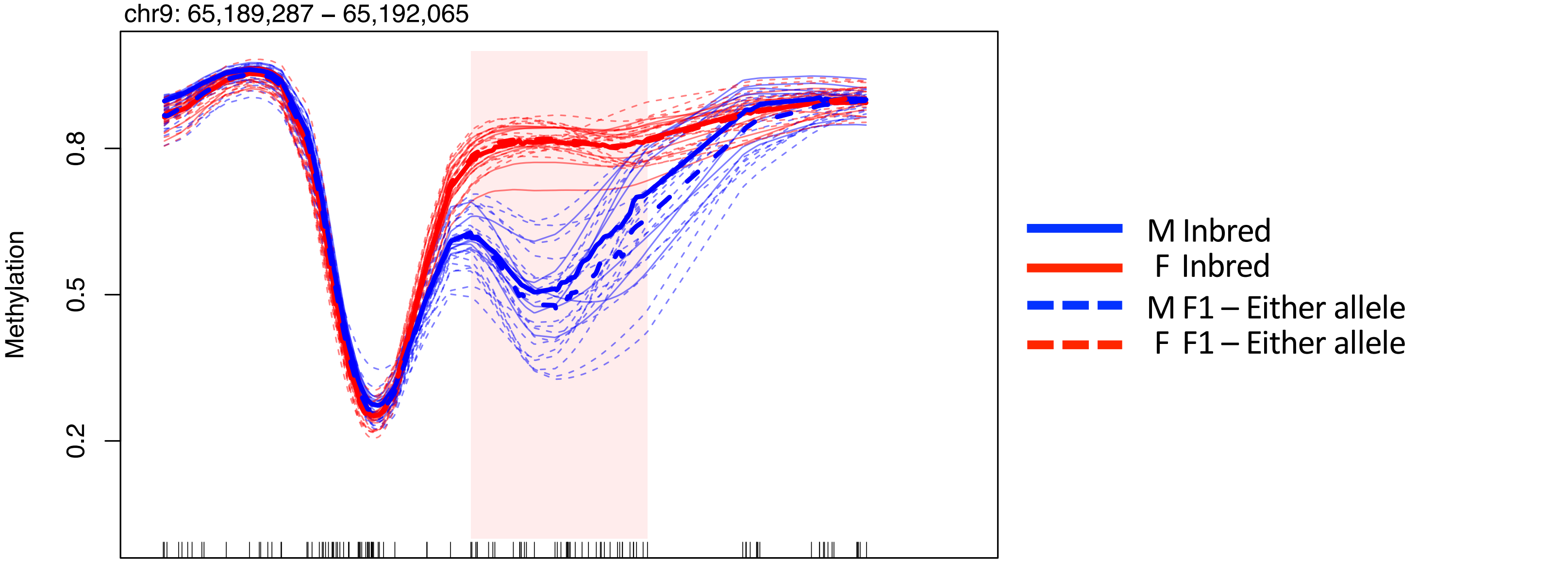

F2 Generation

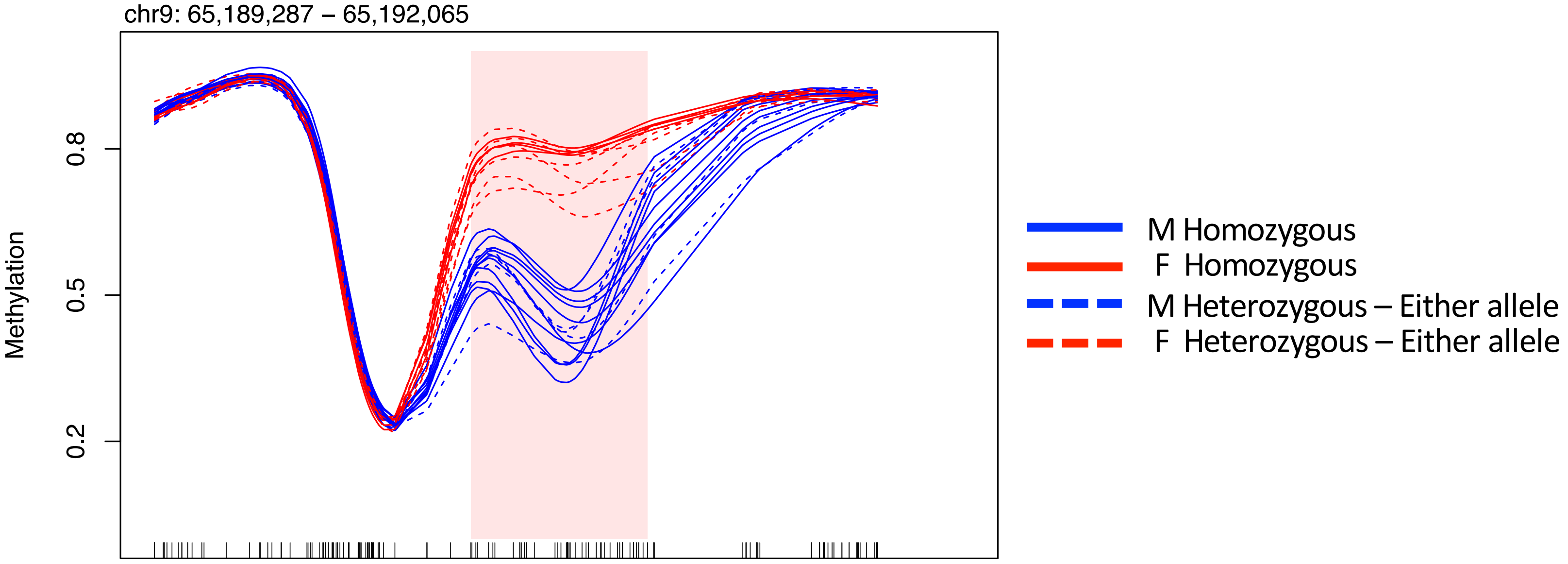

Sex-specific methylation

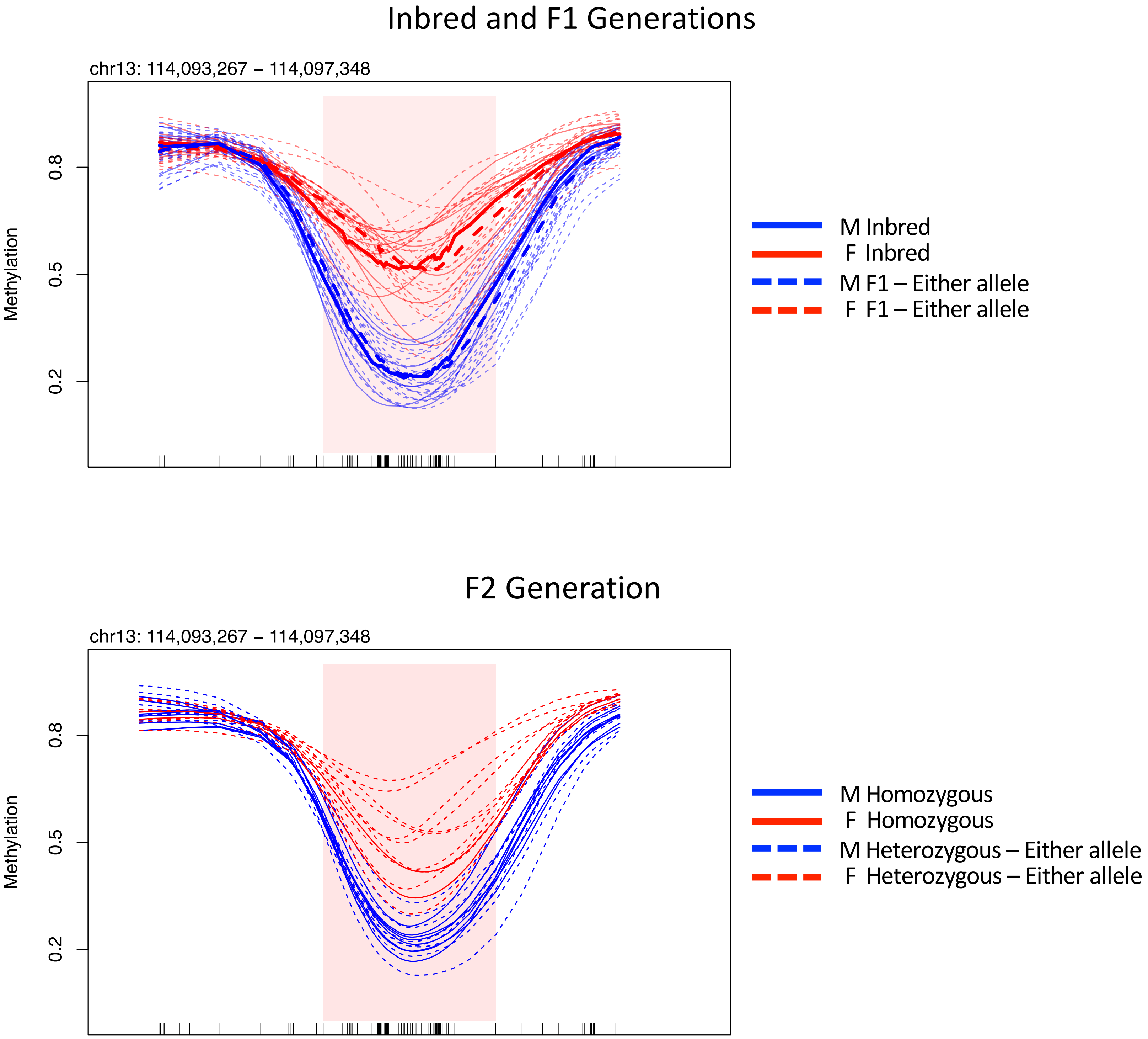

Sex-specific methylation

Inbred and F1 Generations

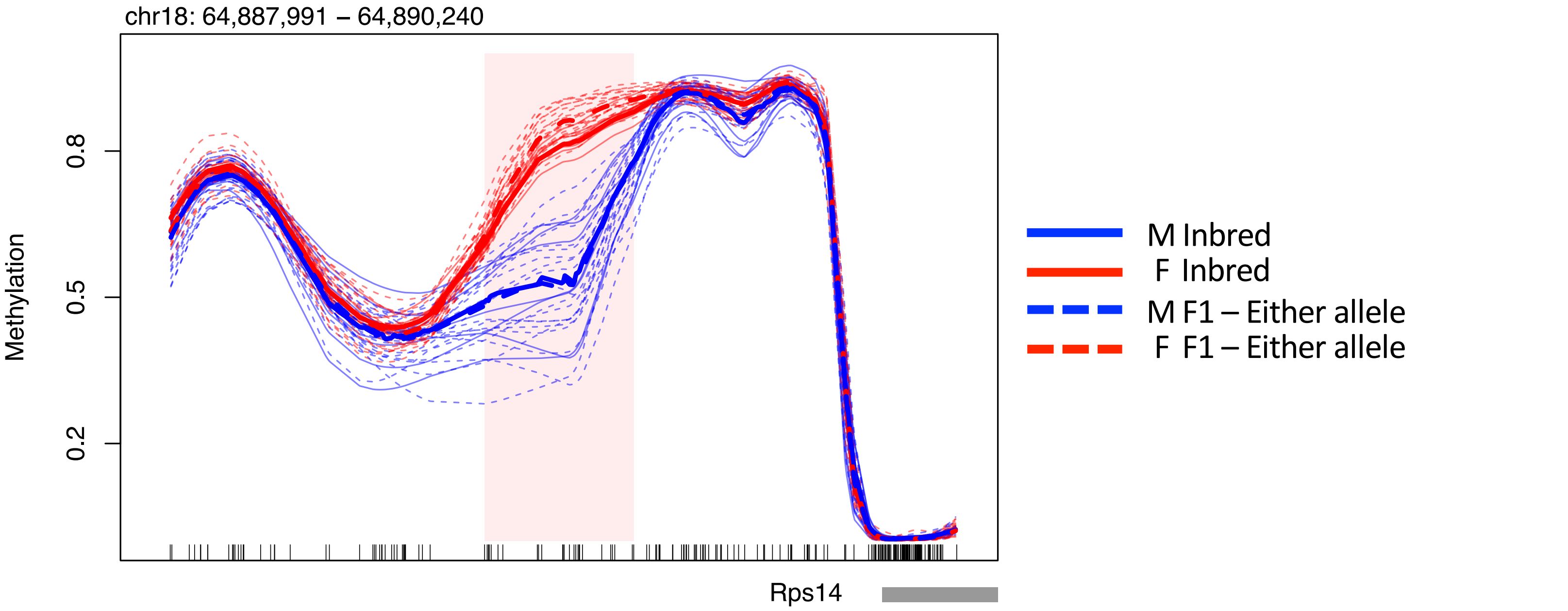

F2 Generation

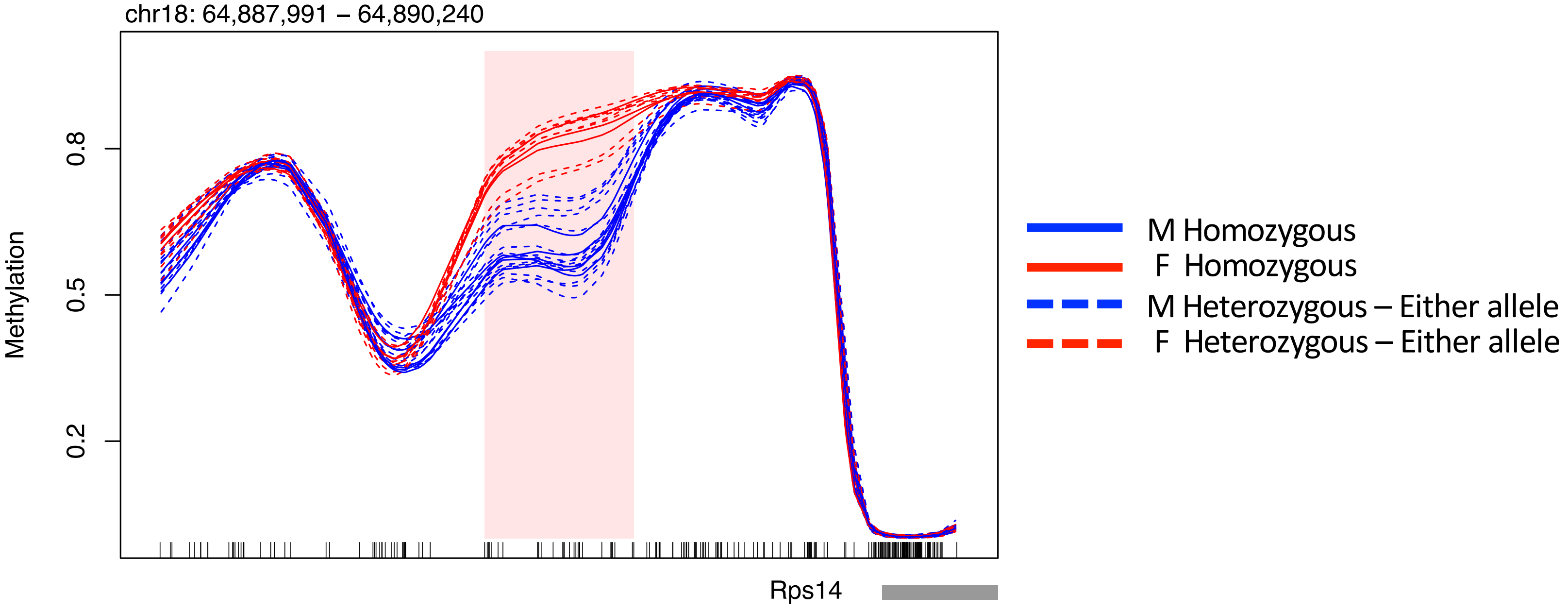

Sex-specific methylation

Inbred and F1 Generations

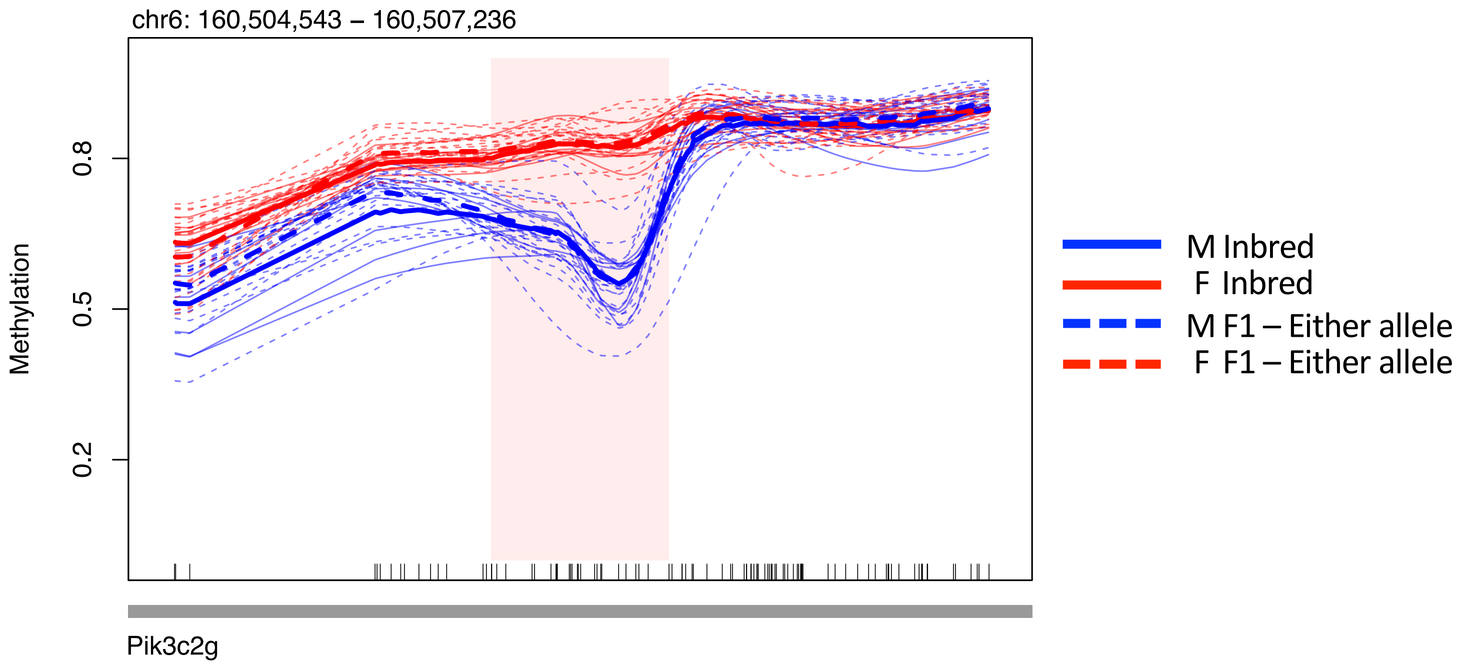

F2 Generation

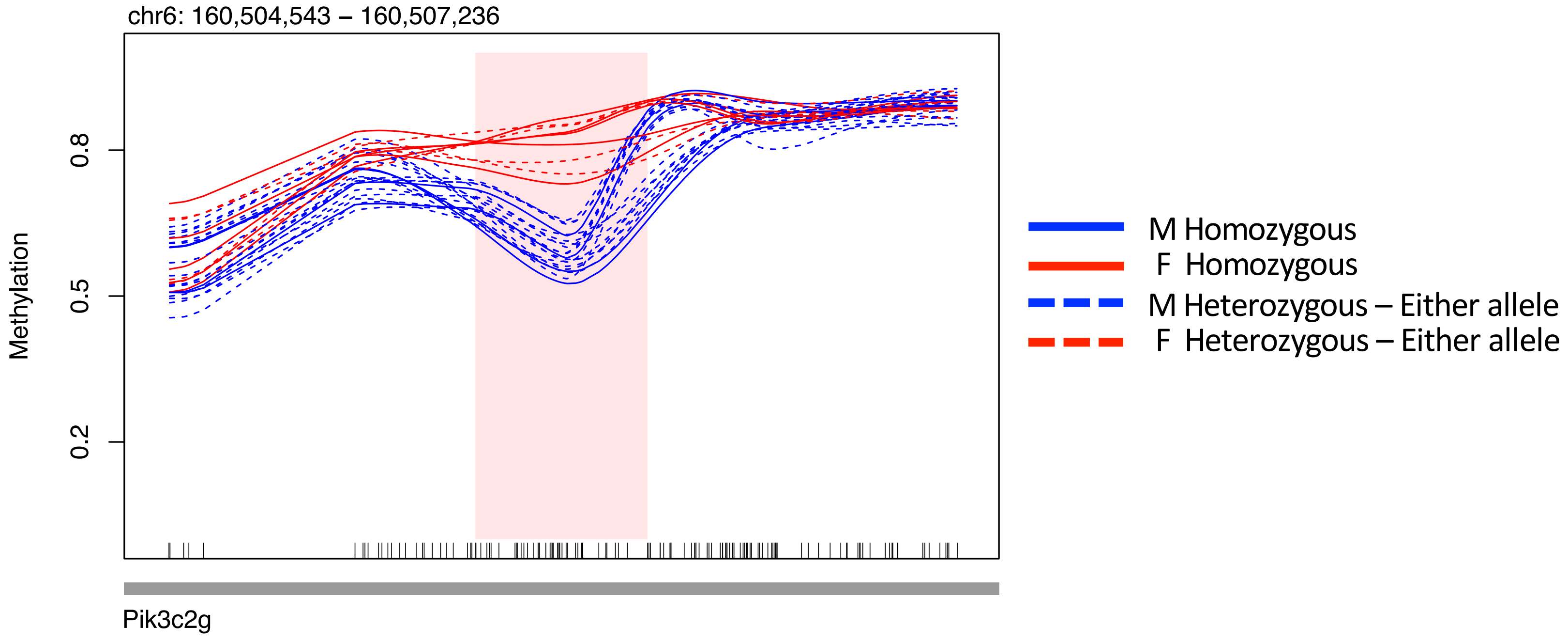

Sex-specific methylation

Inbred and F1 Generations

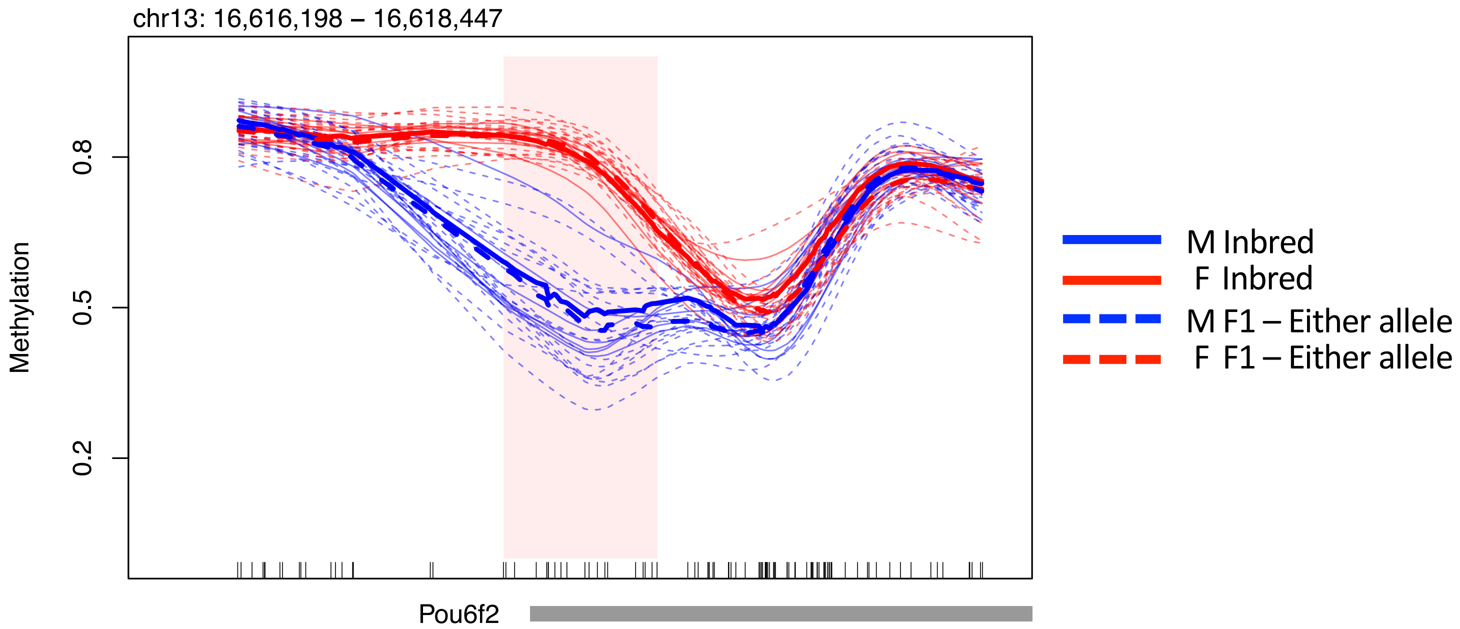

F2 Generation

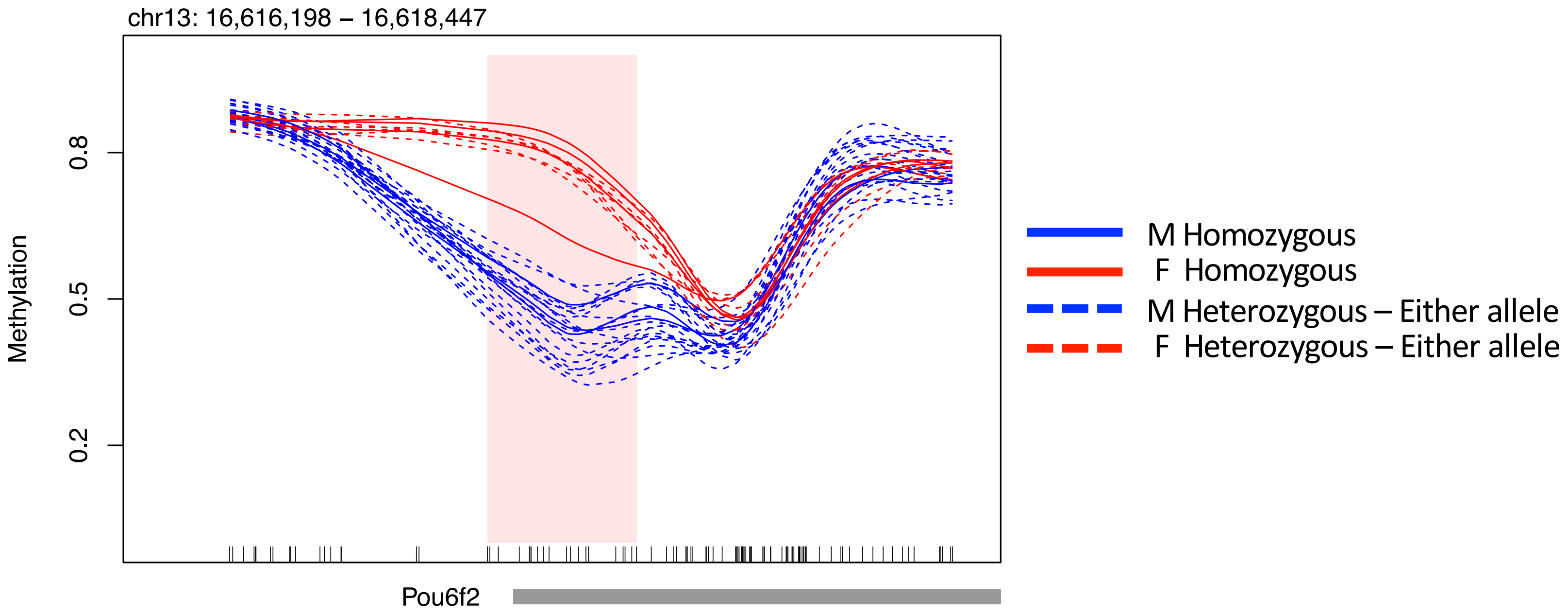

Sex-specific methylation

Inbred and F1 Generations

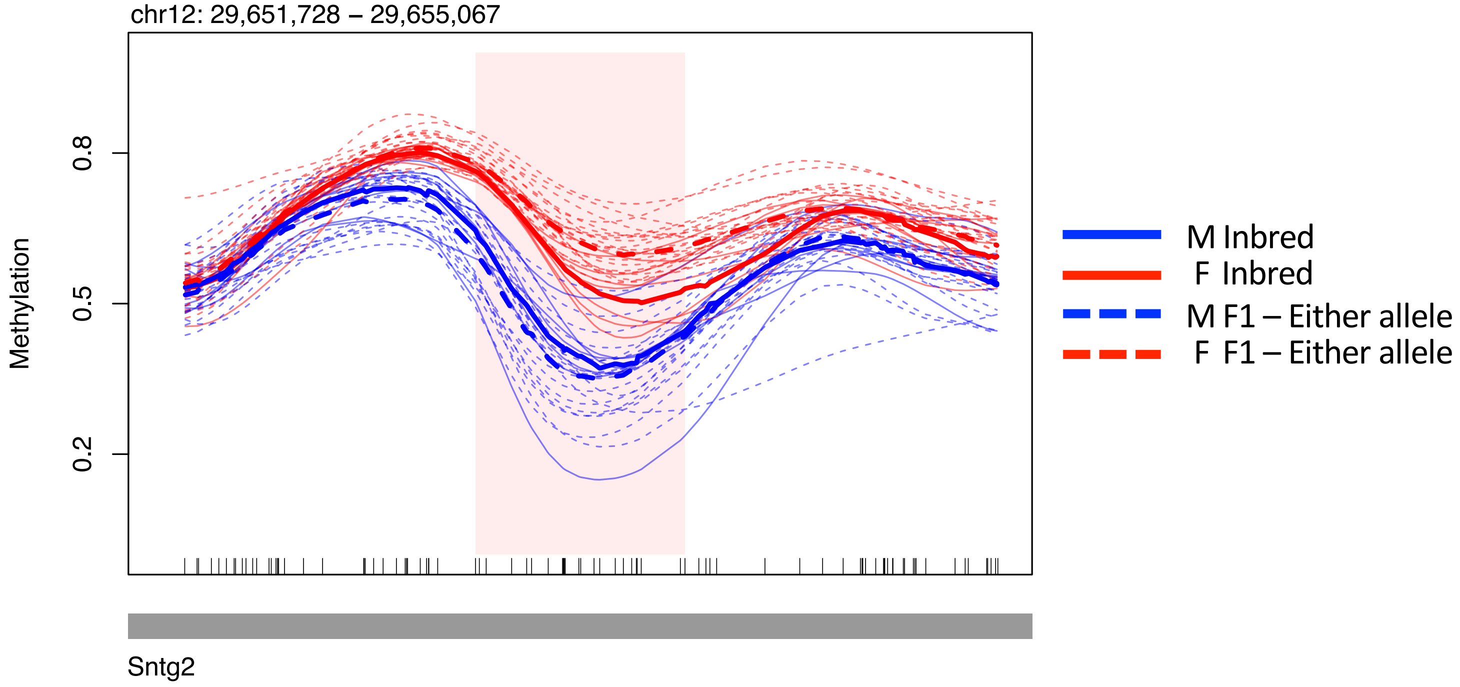

F2 Generation

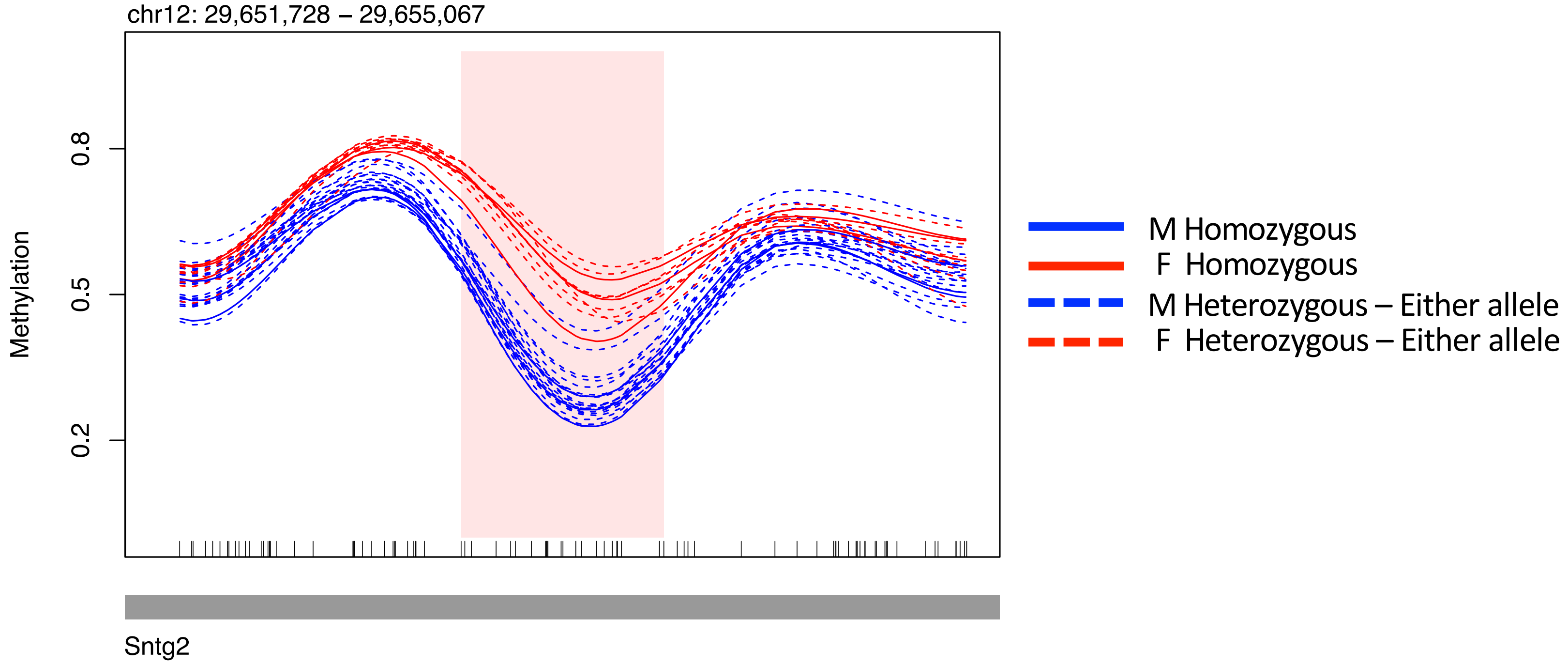

Sex-specific methylation

Inbred and F1 Generations

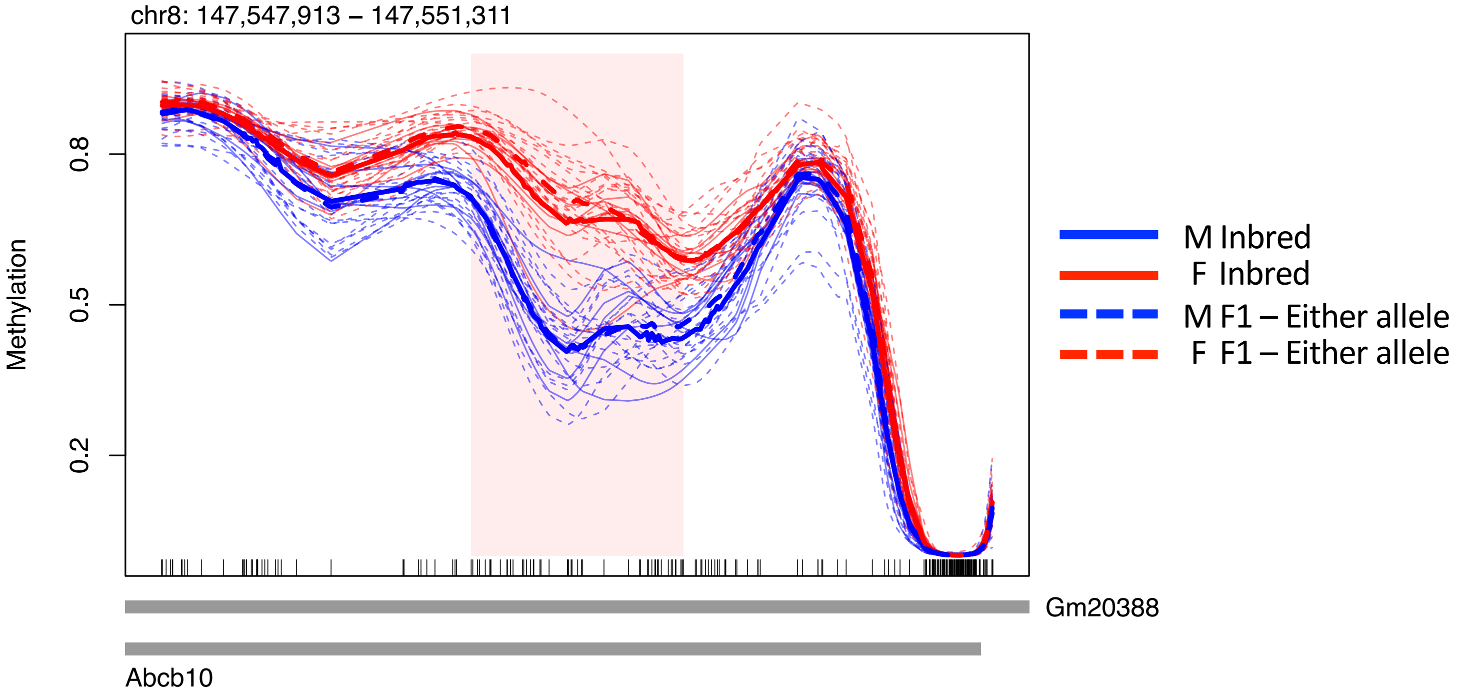

F2 Generation

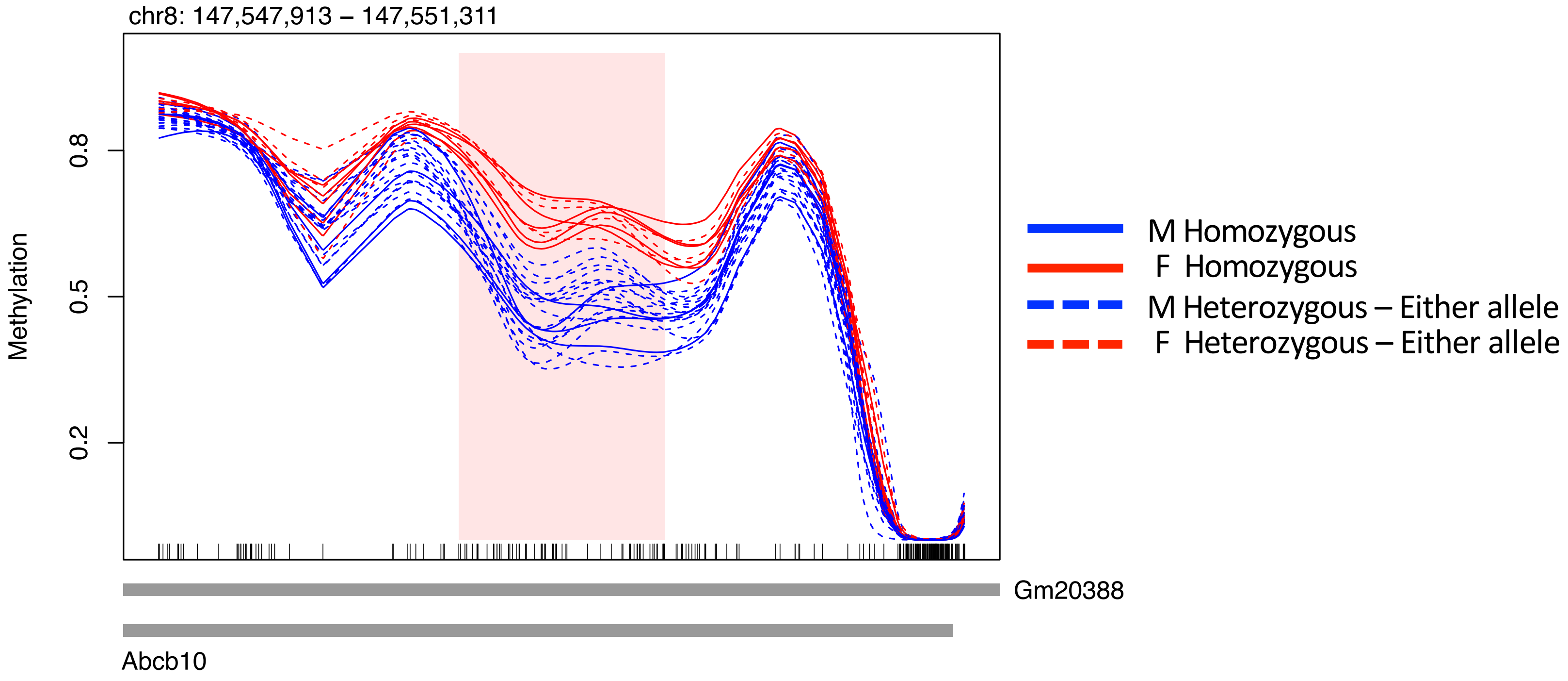

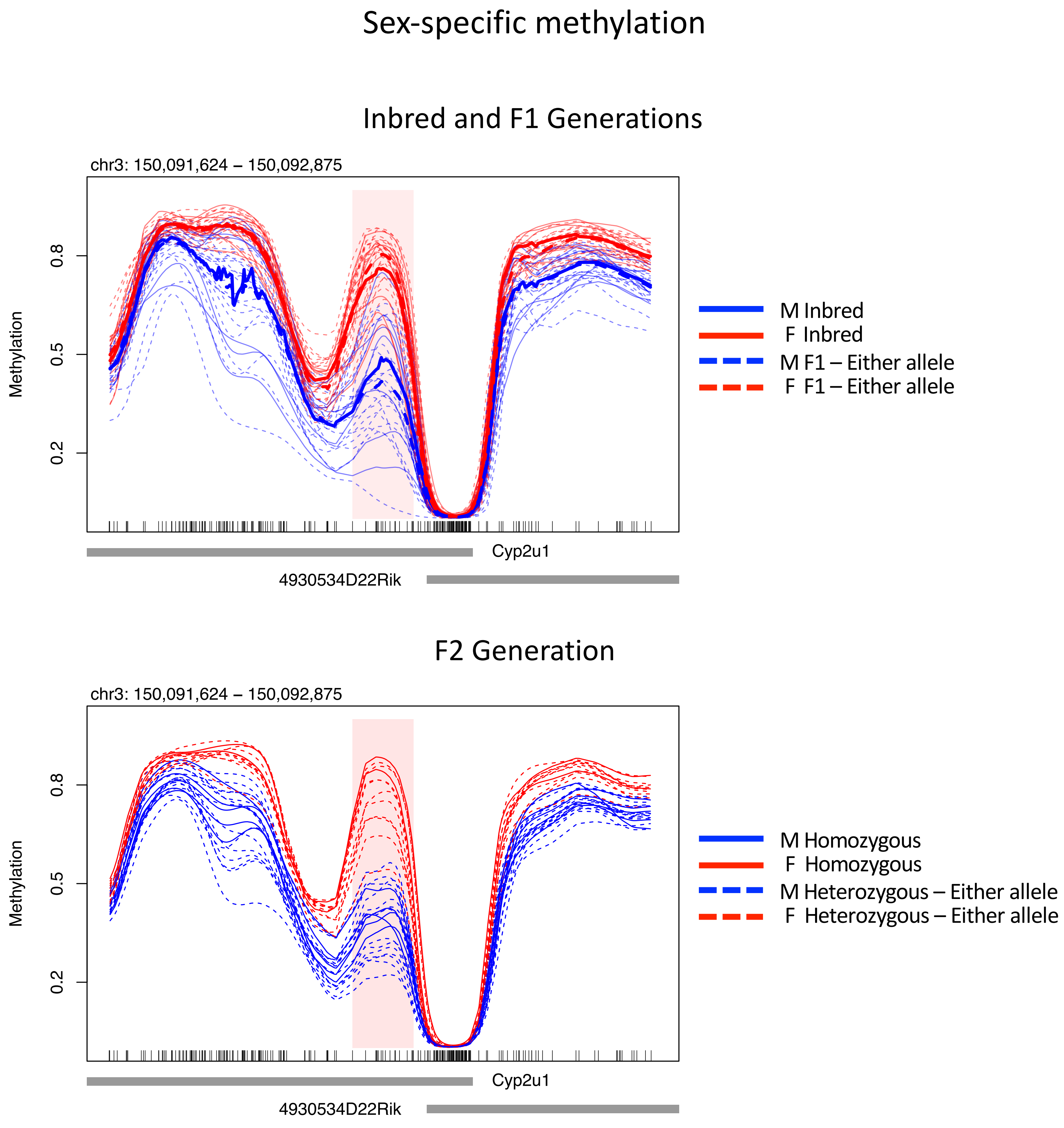

Sex-specific methylation
Inbred and F1 Generations
chr3: 150,091,624 – 150,092,875
Methylation
0.8
0.5
0.2
Cyp2u1
4930534D22Rik
M Inbred
F Inbred
M F1 – Either allele
F F1 – Either allele
F2 Generation
chr3: 150,091,624 – 150,092,875
Methylation
0.8
0.5
0.2
Cyp2u1
4930534D22Rik
M Homozygous
F Homozygous
M Heterozygous – Either allele
F Heterozygous – Either allele

Sex-specific methylation

Inbred and F1 Generations

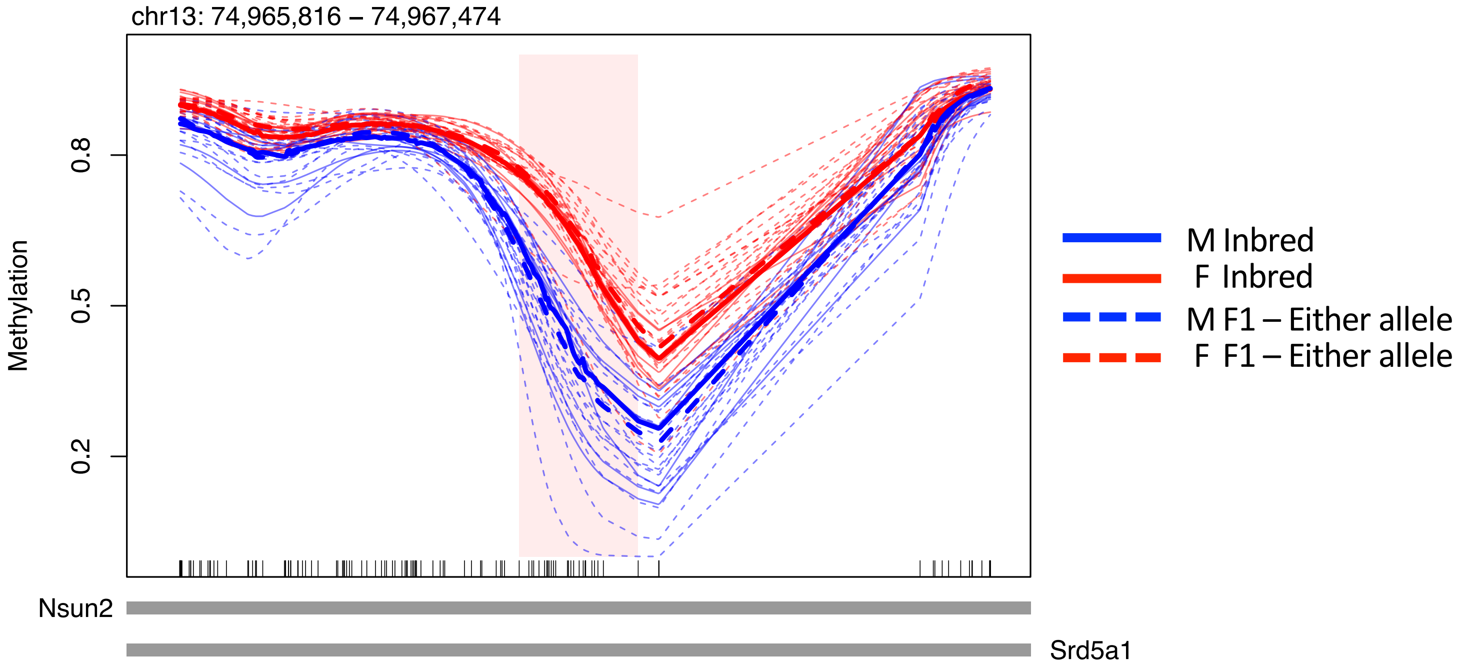

F2 Generation

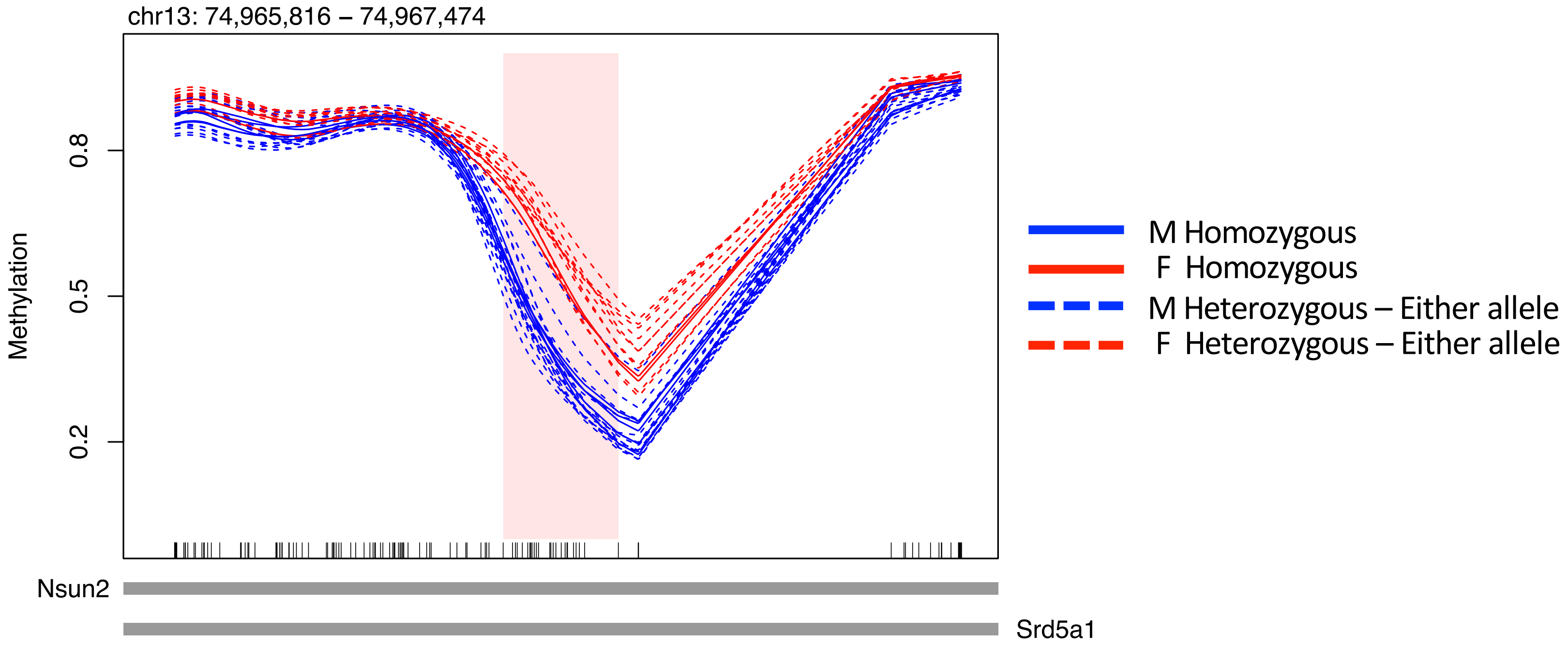

Sex-specific methylation

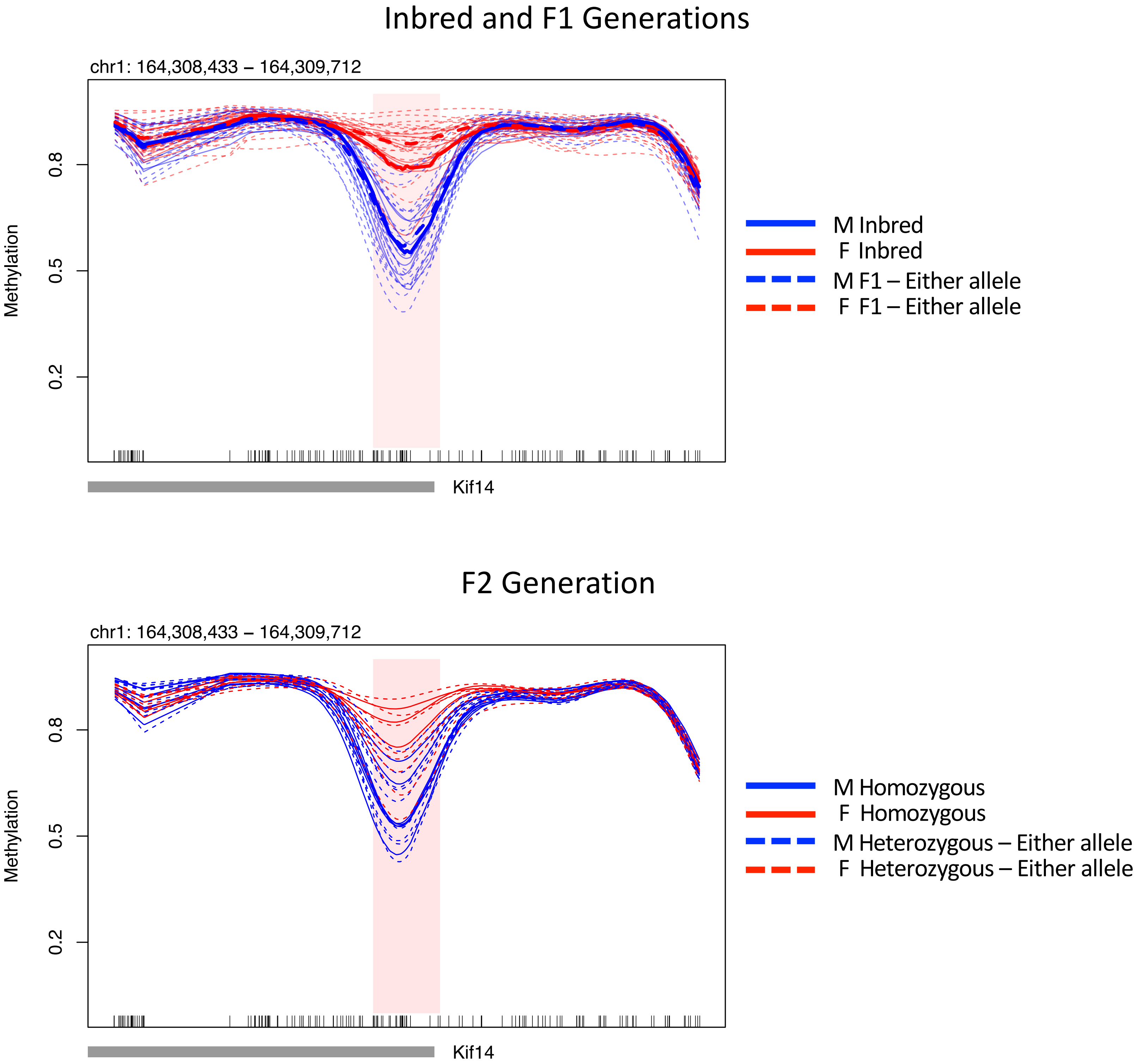

Sex-specific methylation

Inbred and F1 Generations

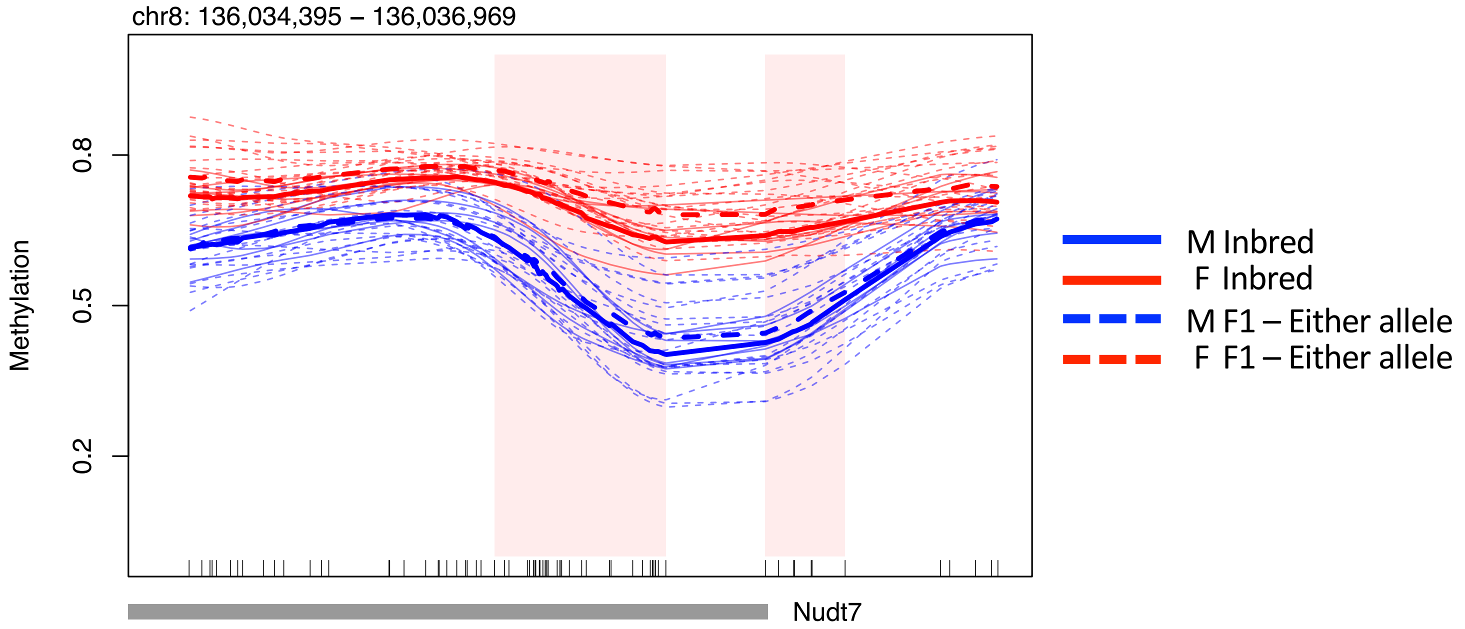

F2 Generation

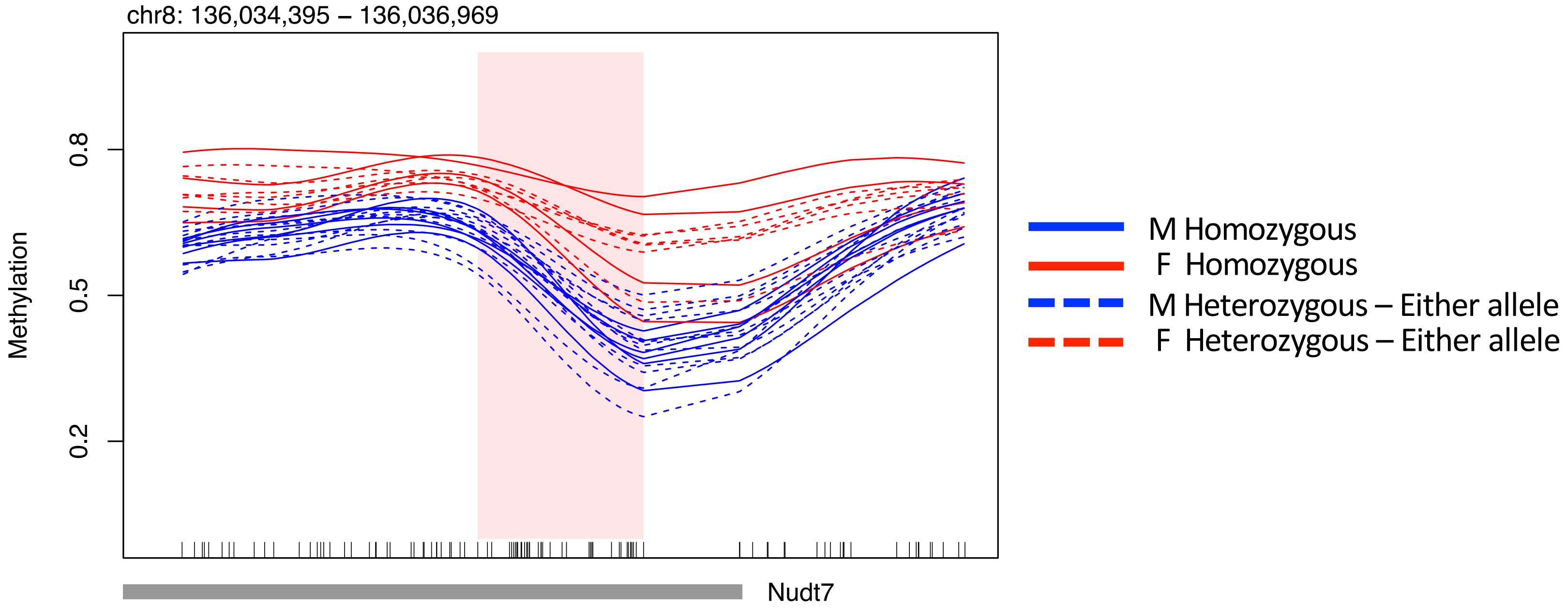

Sex-specific methylation

Inbred and F1 Generations

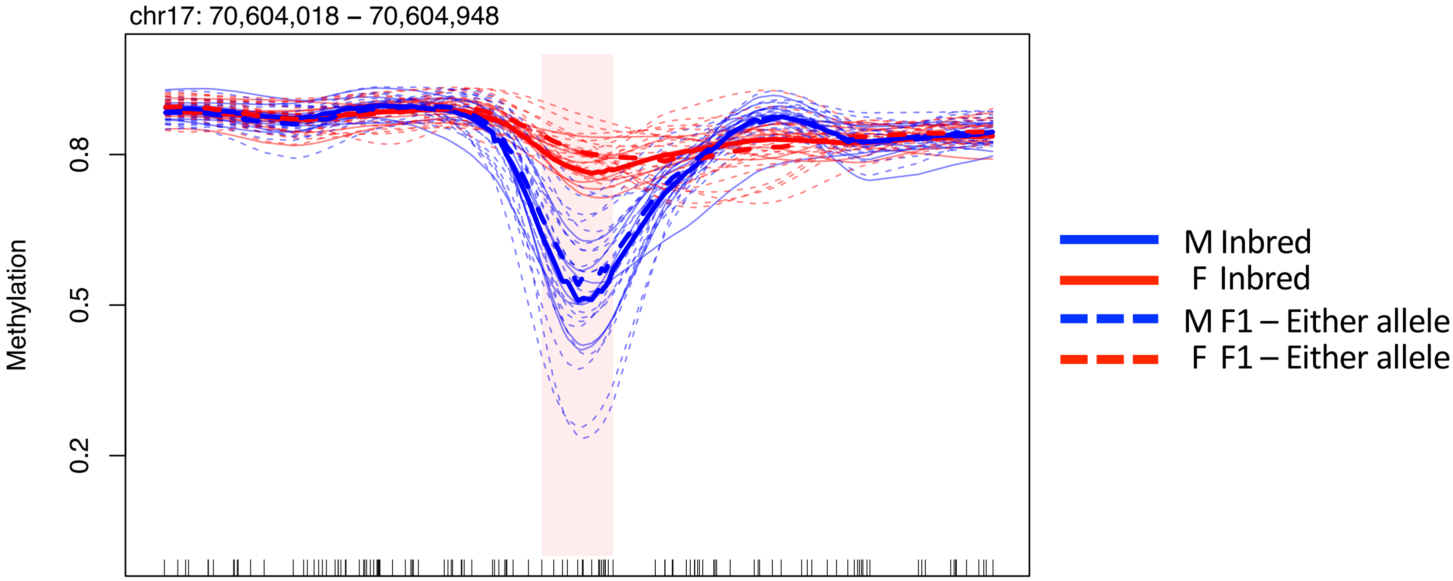

F2 Generation

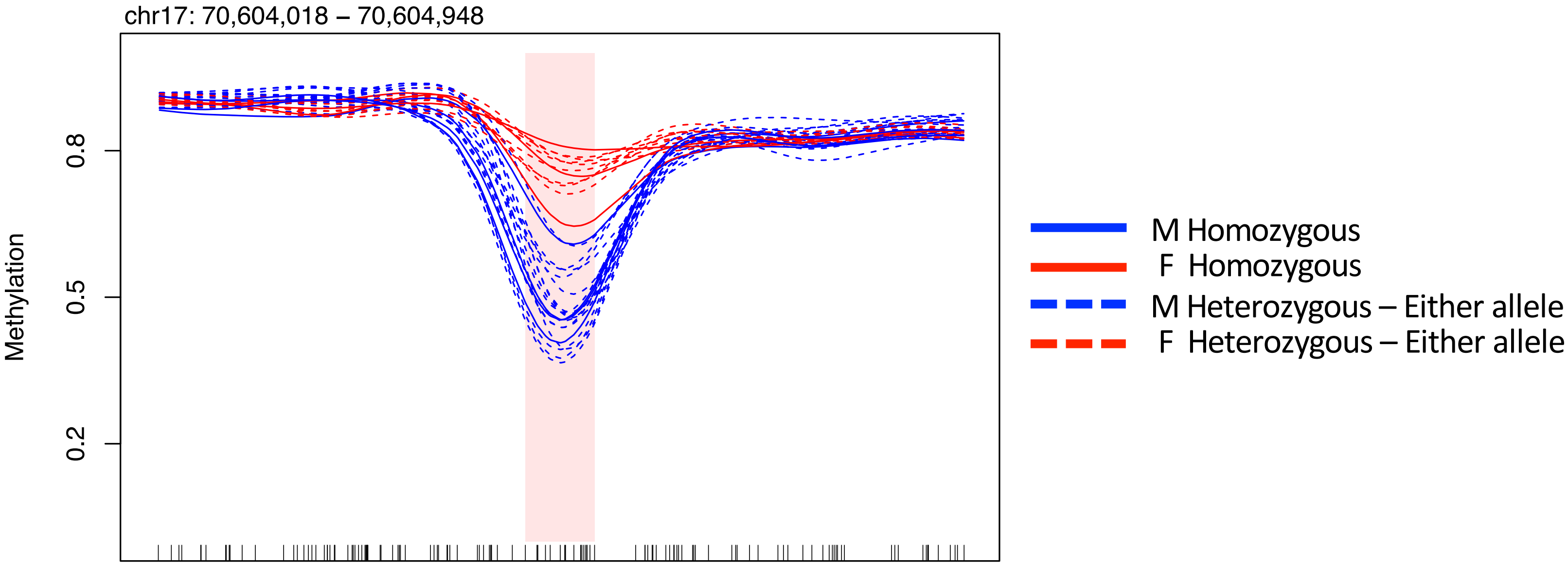

Sex-specific methylation

Inbred and F1 Generations

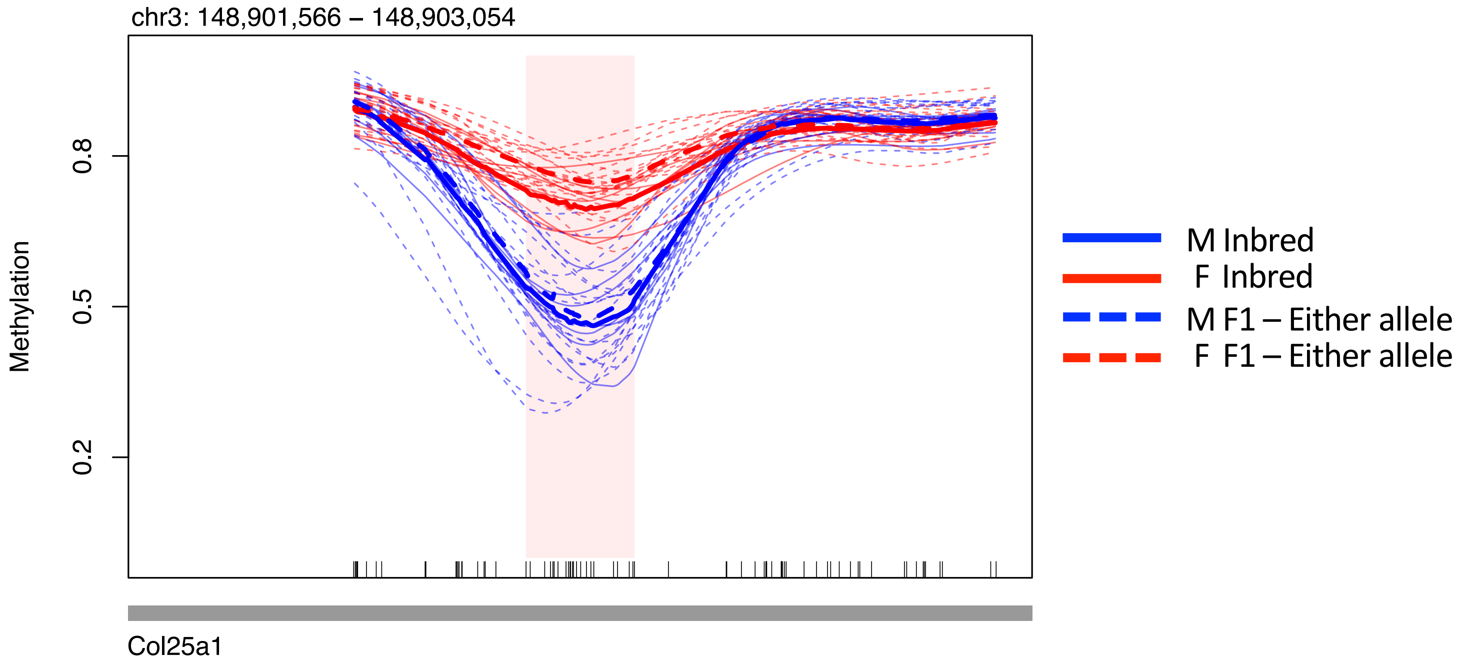

F2 Generation

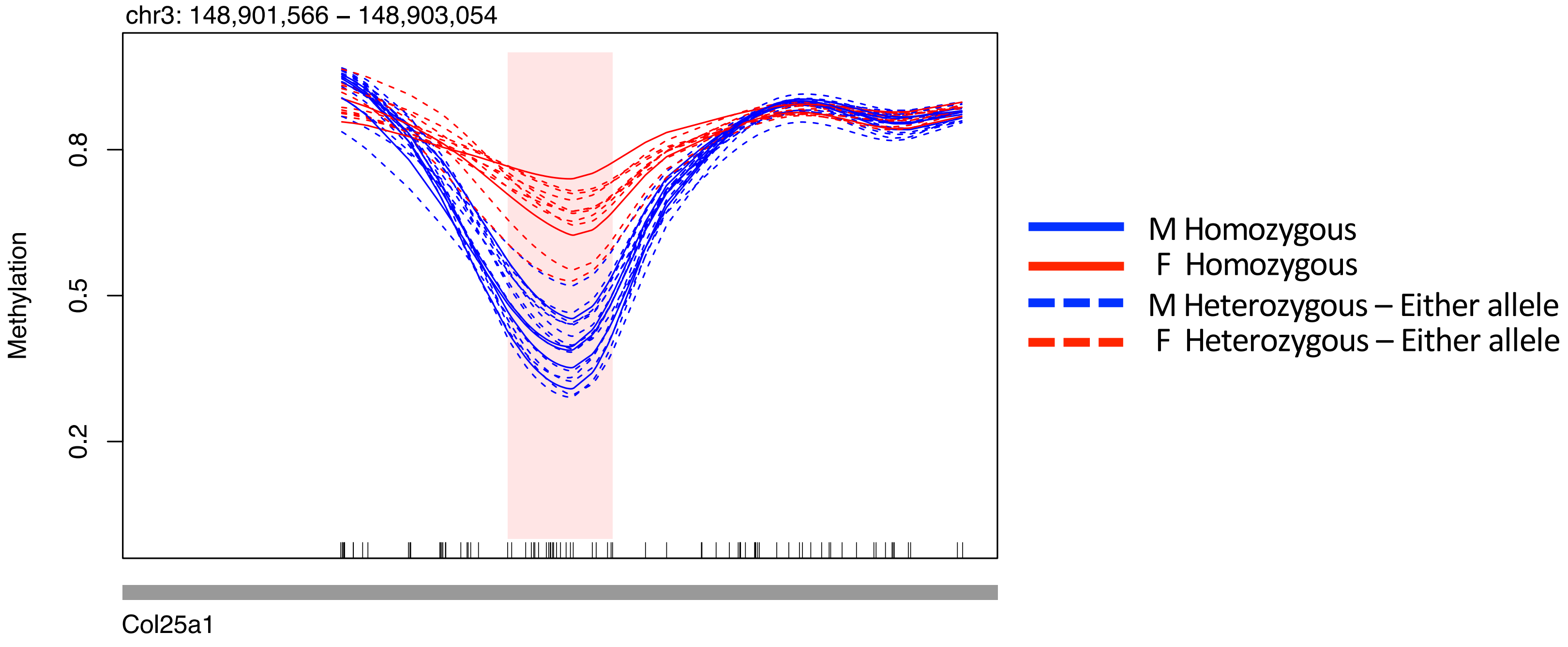

Sex-specific methylation

Inbred and F1 Generations

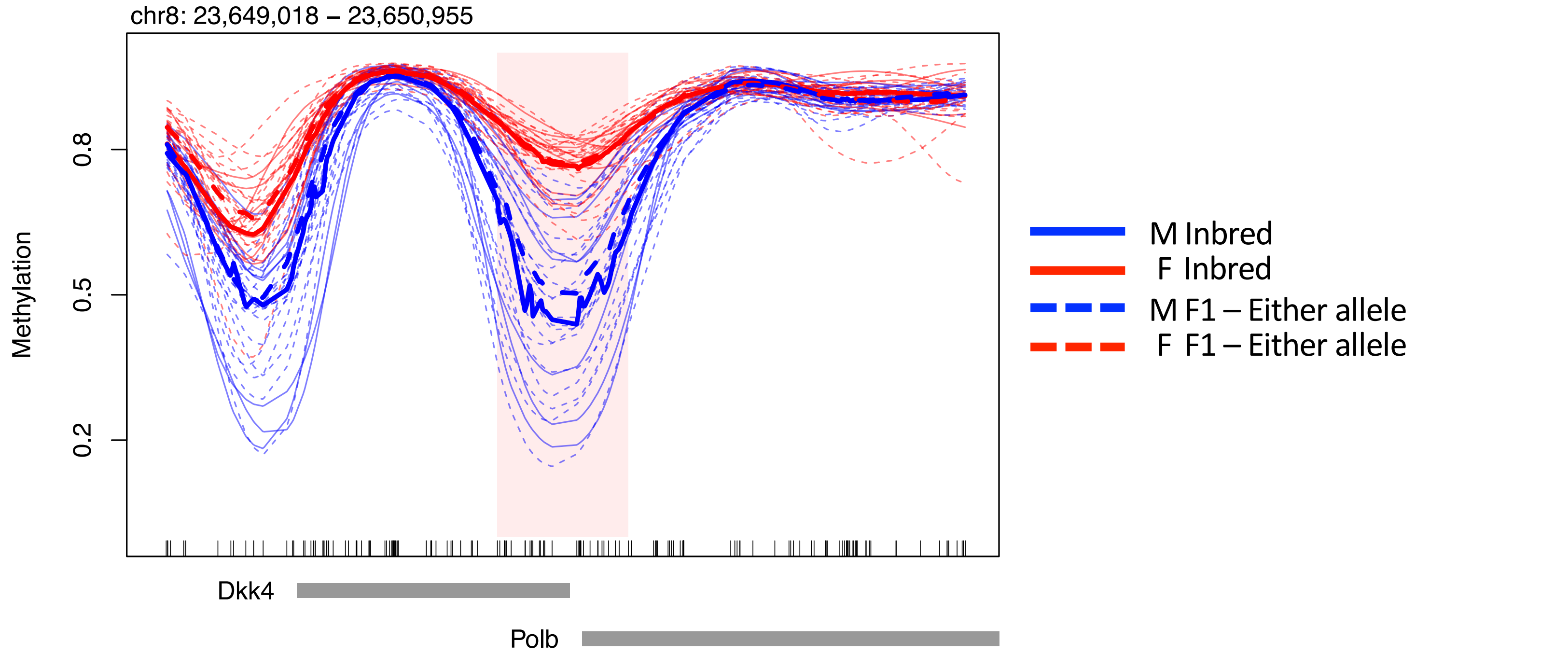

F2 Generation

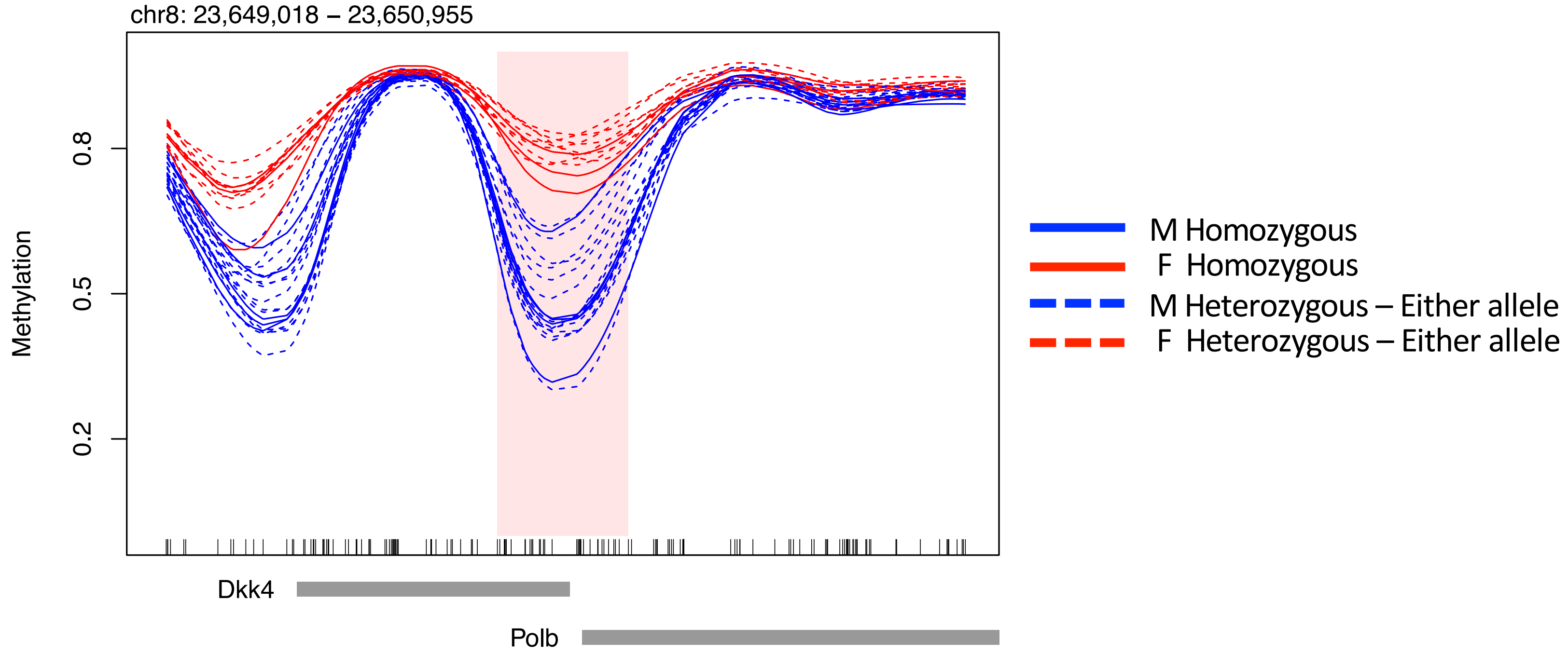

Sex-specific methylation

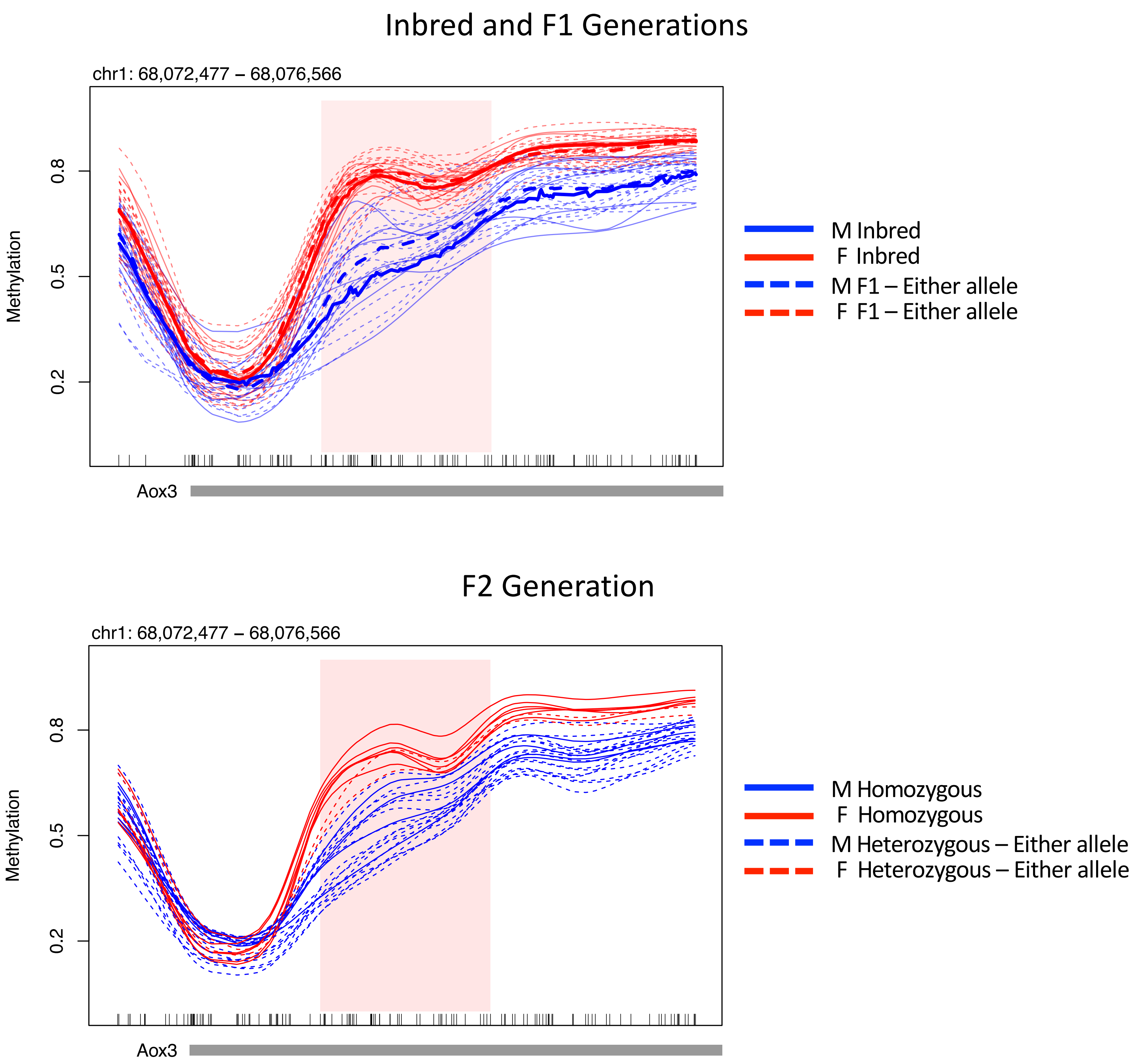

Sex-specific methylation

Inbred and F1 Generations

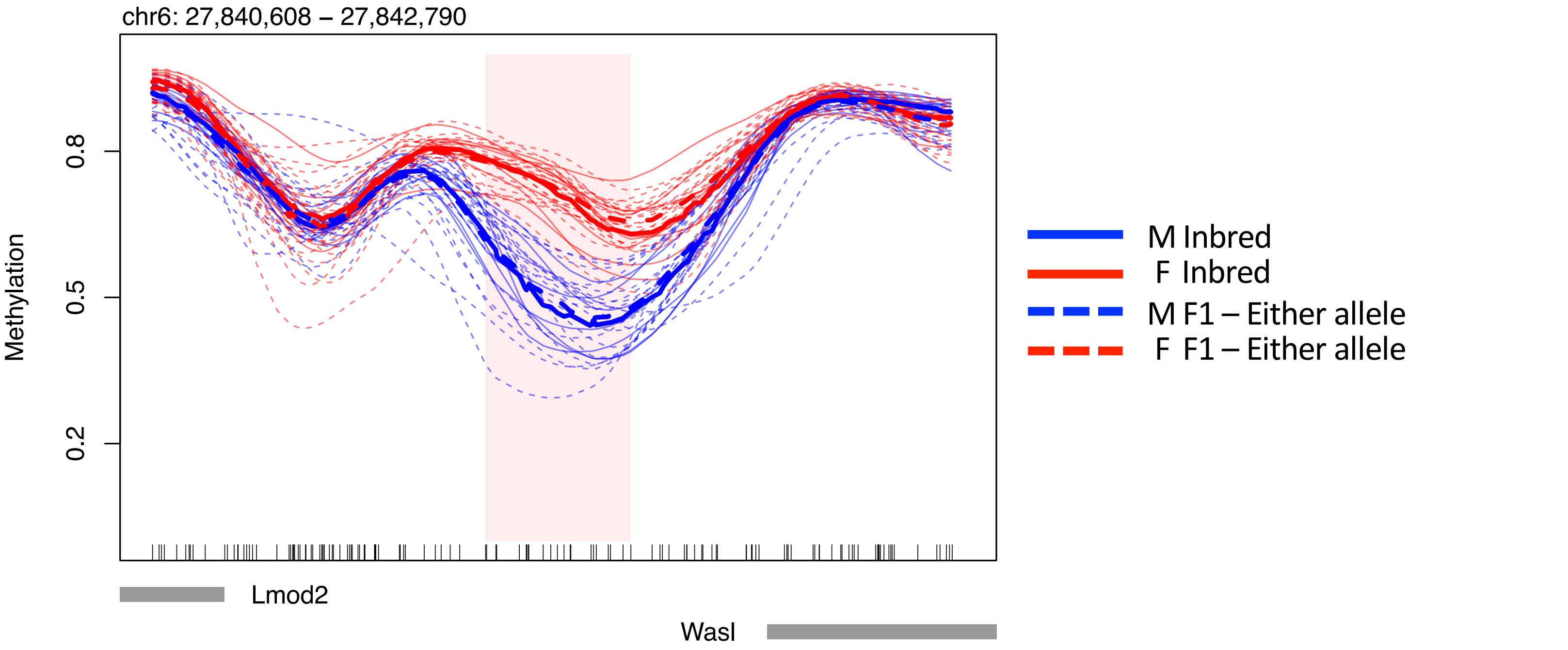

F2 Generation

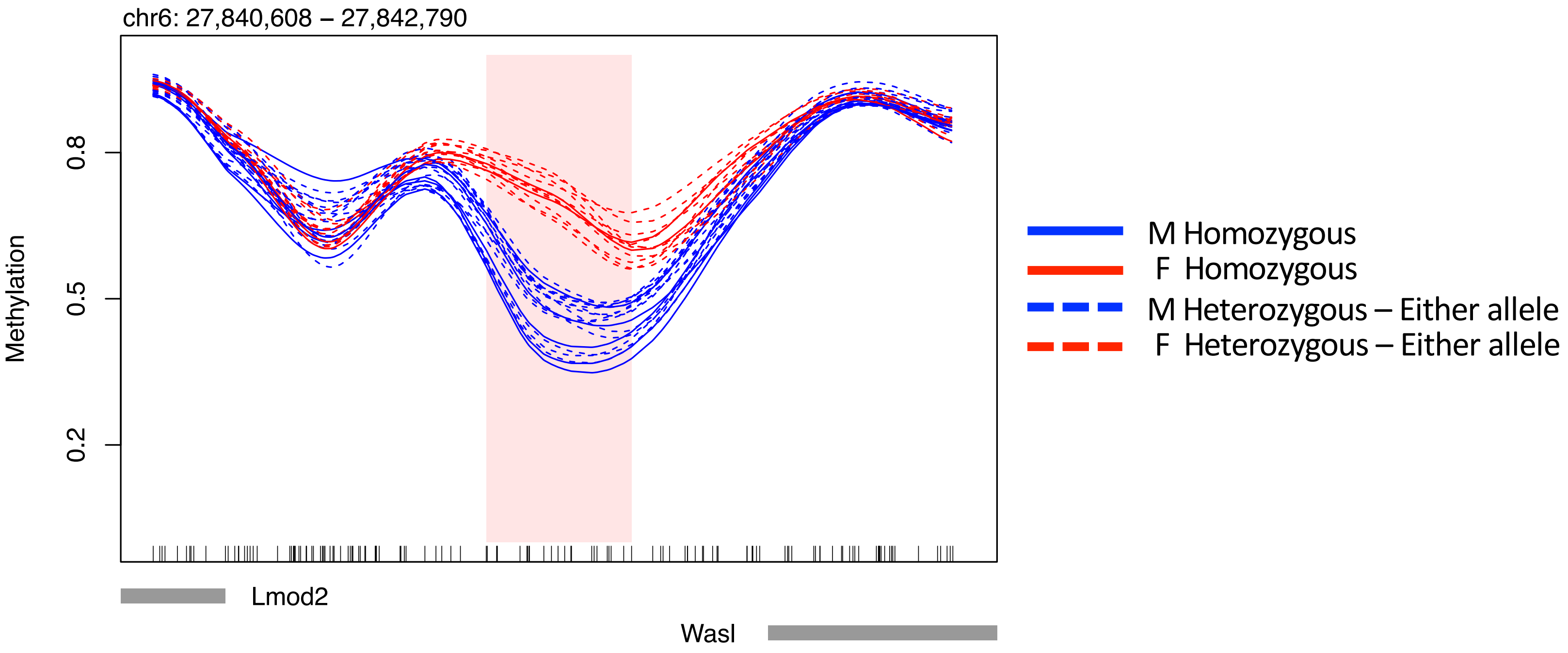

Sex-specific methylation

Inbred and F1 Generations

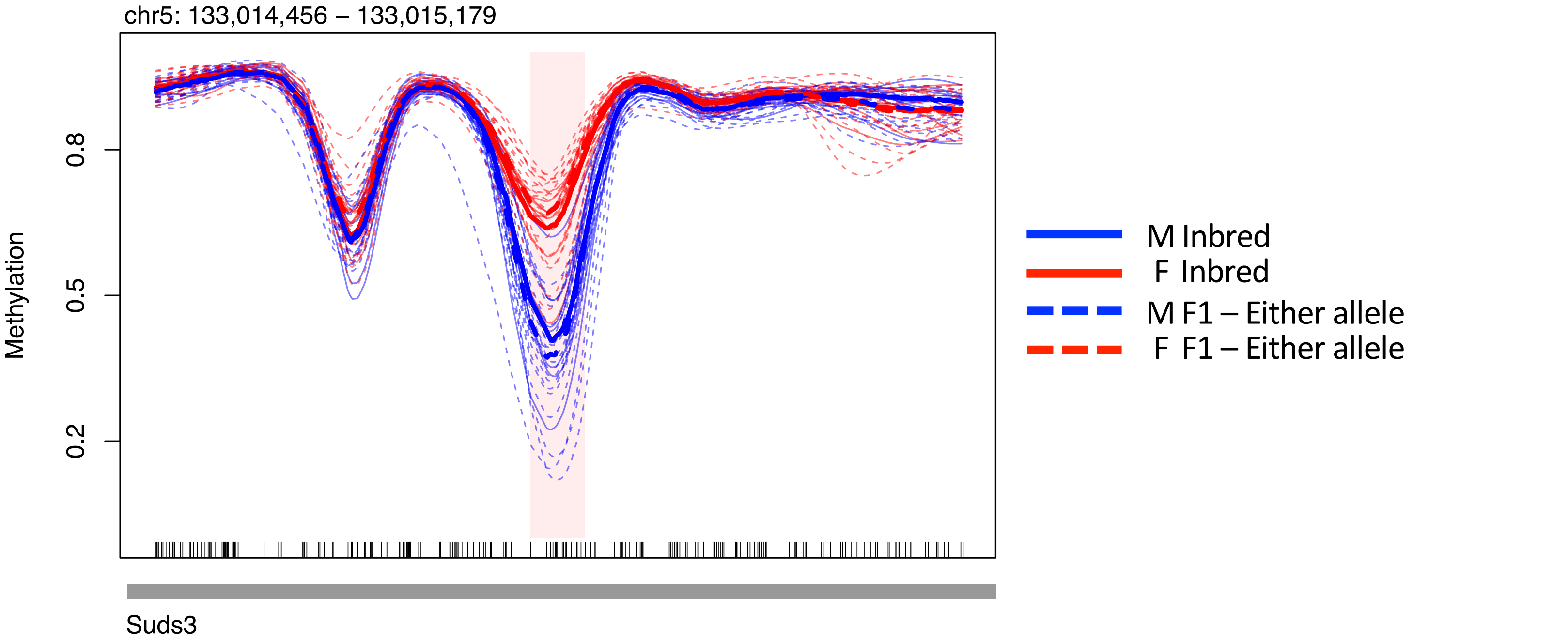

F2 Generation

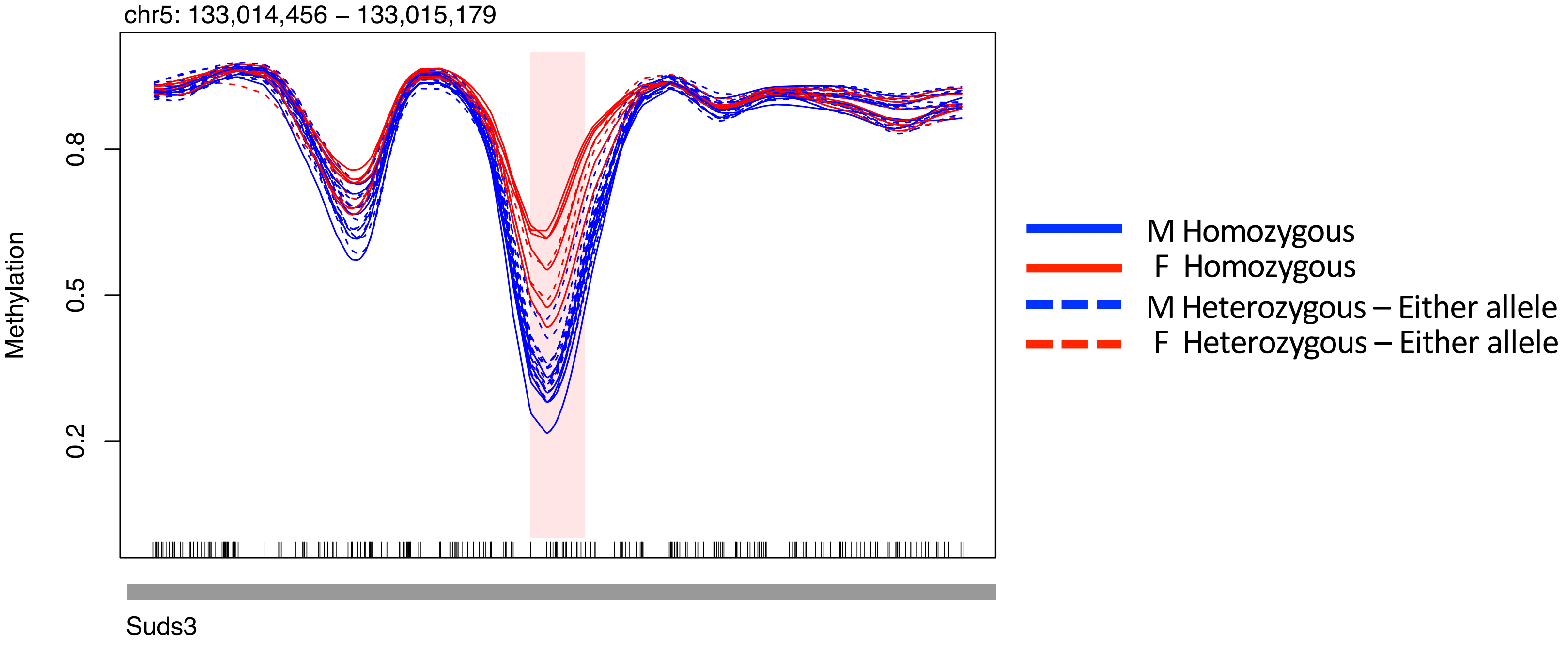

Sex-specific methylation

Inbred and F1 Generations

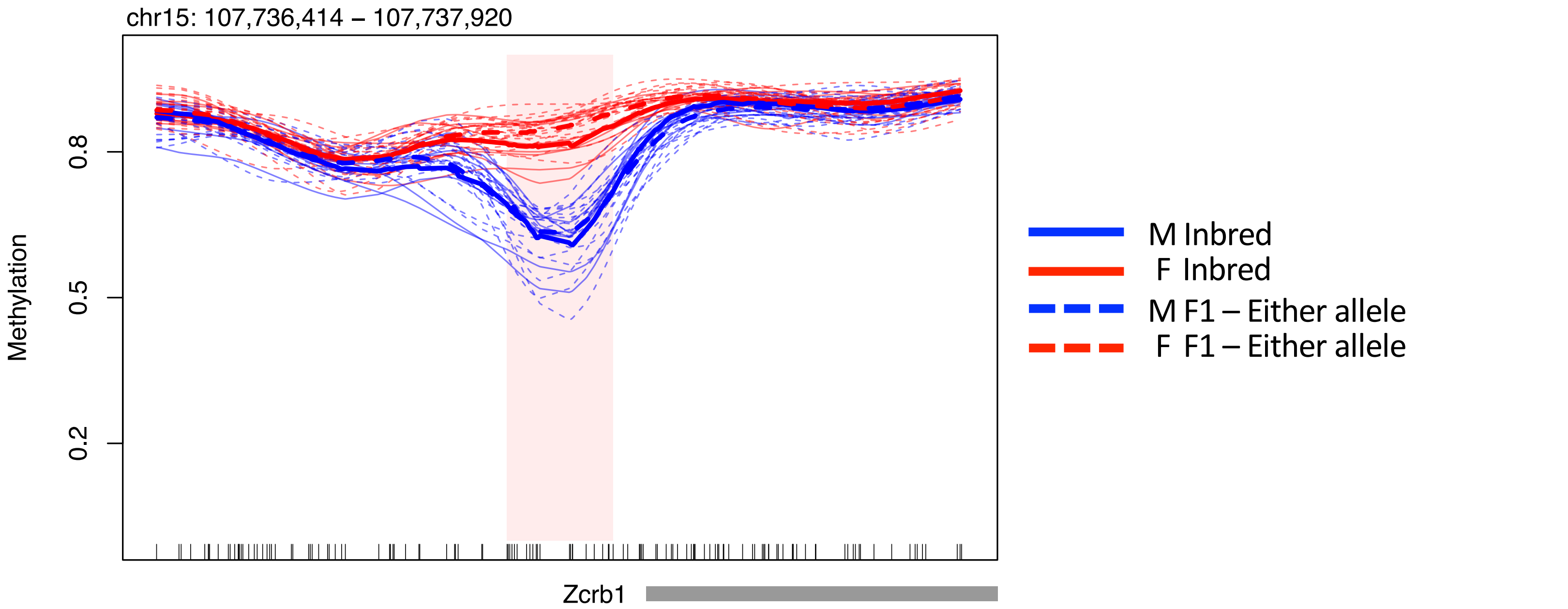

F2 Generation

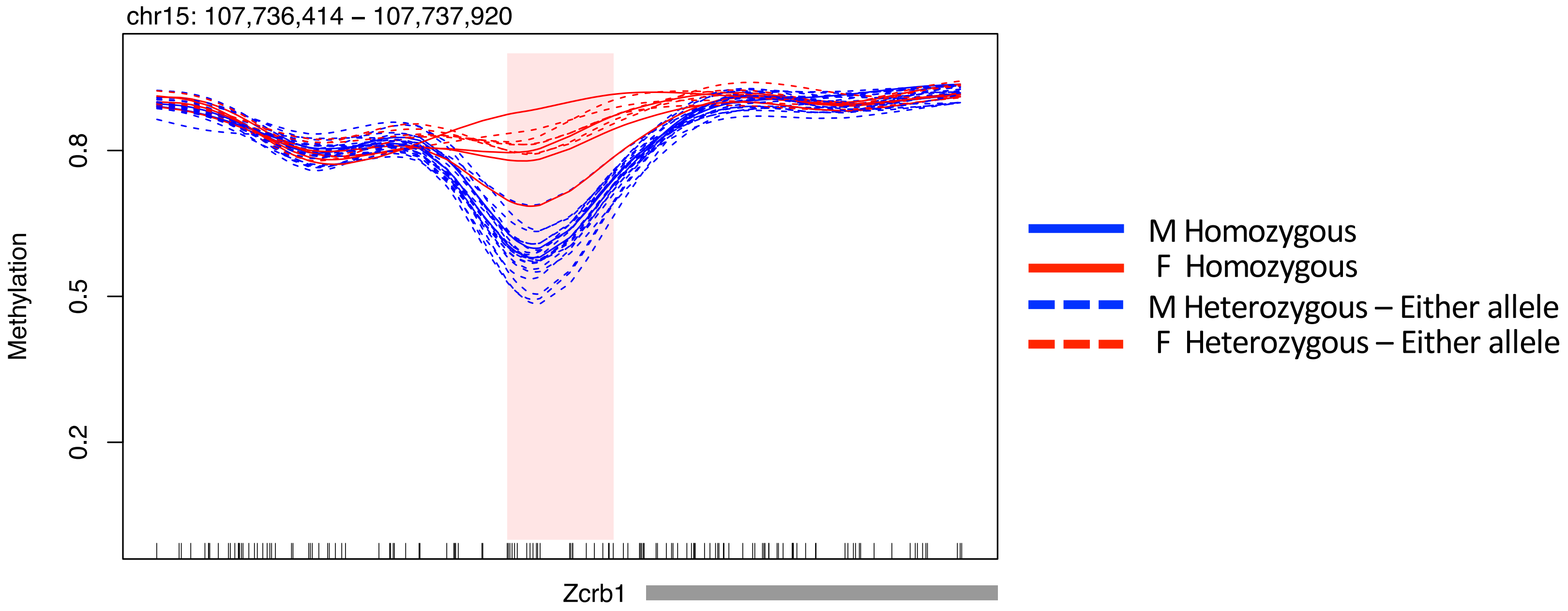

Sex-specific methylation

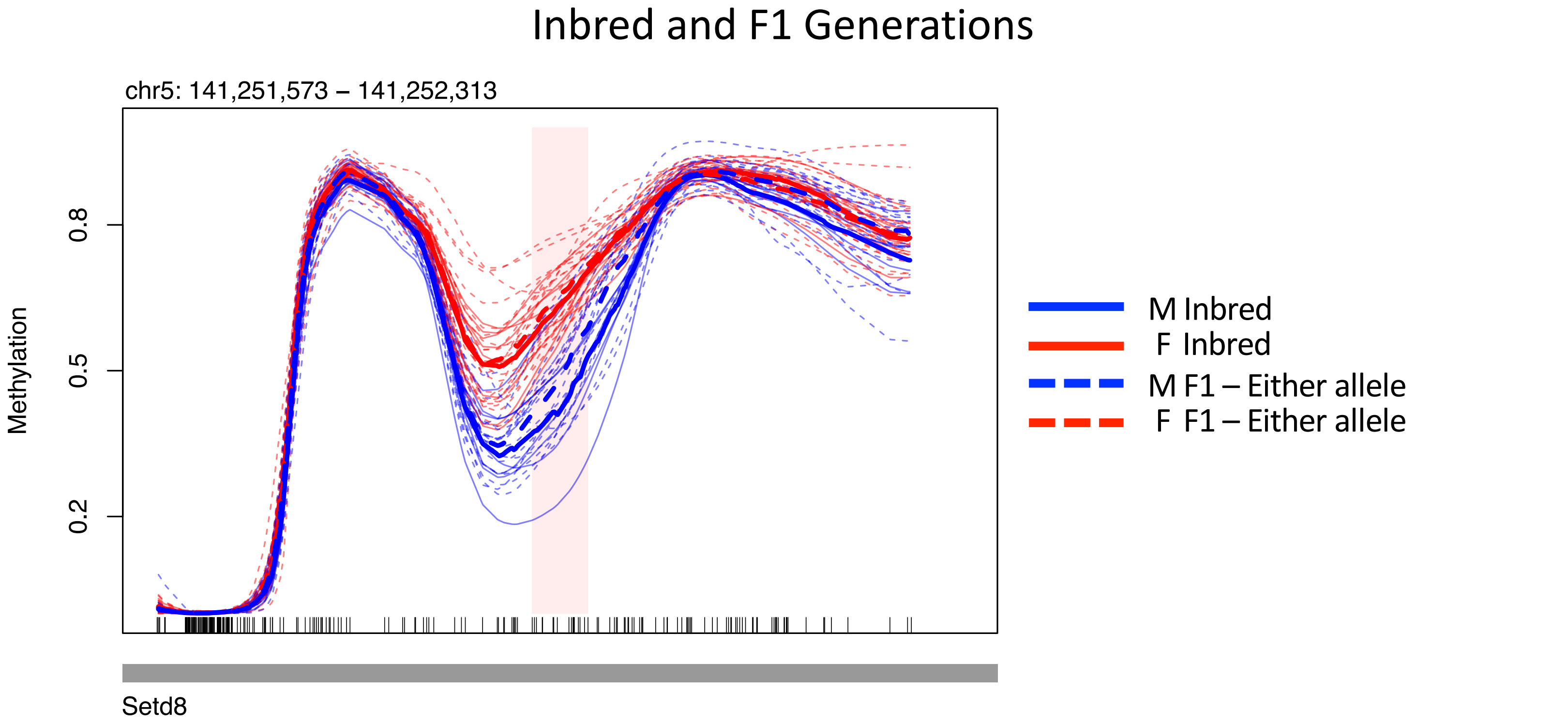

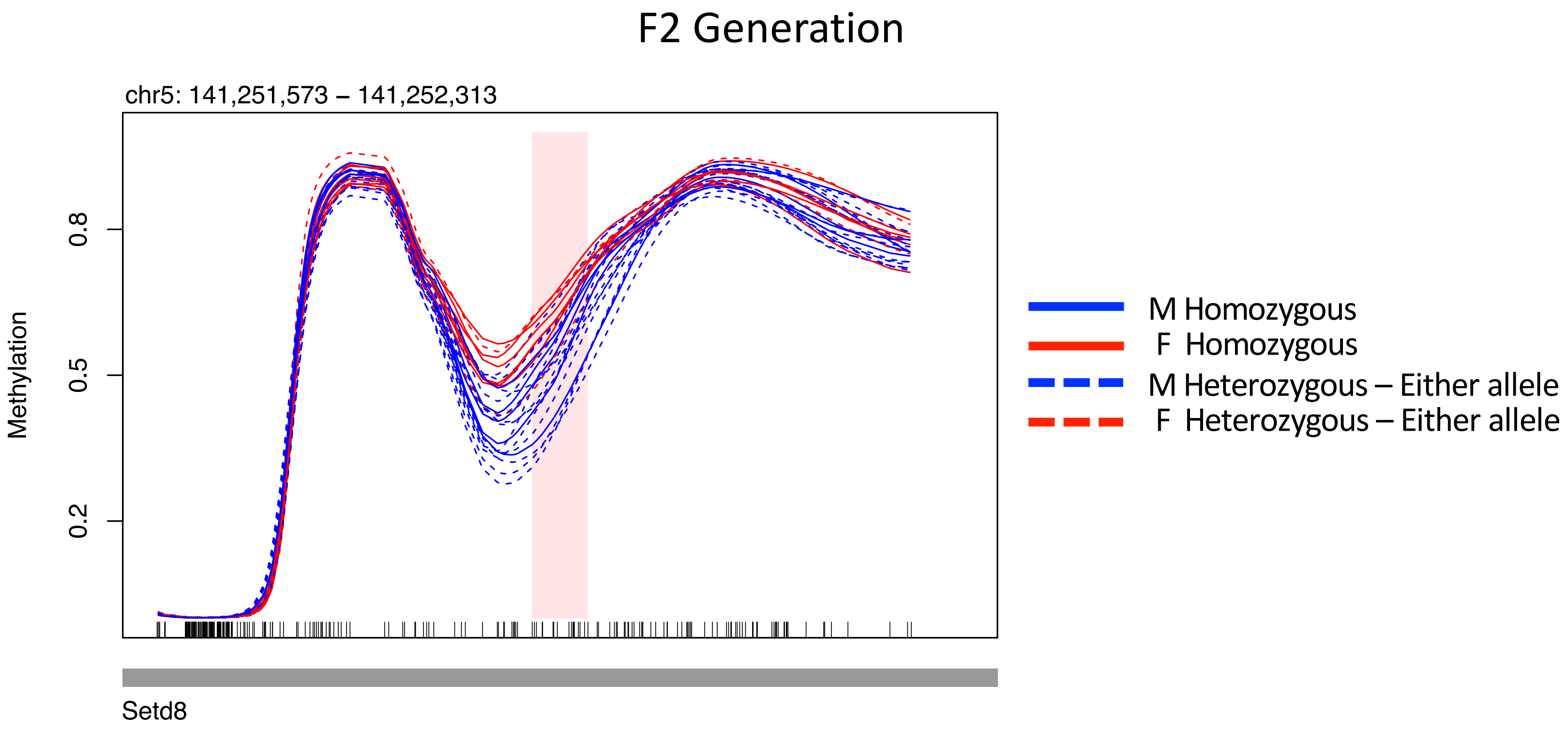

Sex-specific methylation

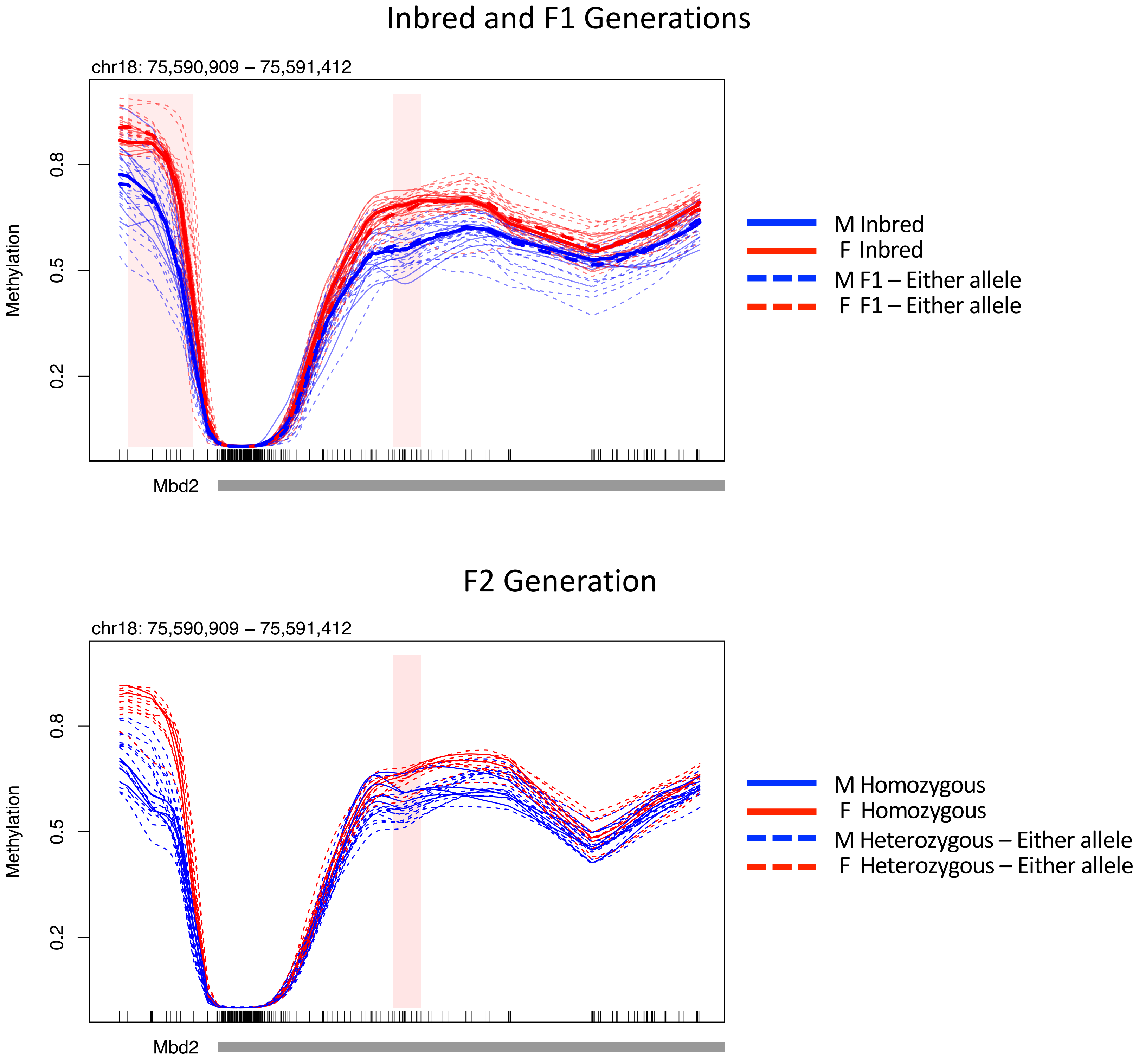

Supplement: Supplementary file 7 — F2 candidate region methylation. Liver methylation from the F2 generation over the dominant trans-acting meQTL/transvection/paramutation DMRs, cis-acting meQTL DMRs, nondominant trans-acting meQTL DMRs, and sex-specific DMRs chosen for targeted analysis in the F2s. Bold lines represent coverage-weighted mean methylation of the respective group and CpG sites included in the final analysis are denoted by tick marks on the x-axis. [file 41588_2026_2604_MOESM7_ESM.pdf]
